# Rh(iii)-catalyzed sp^3^/sp^2^–C–H heteroarylations *via* cascade C–H activation and cyclization†

**DOI:** 10.1039/d3sc06955a

**Published:** 2024-03-27

**Authors:** Atul K. Chaturvedi, Rahul K. Shukla, Chandra M. R. Volla

**Affiliations:** a Department of Chemistry, Indian Institute of Technology Bombay Powai Mumbai 400076 India Chandra.volla@chem.iitb.ac.in

## Abstract

The development of an efficient strategy for facile access to quinoline-based bis-heterocycles holds paramount importance in medicinal chemistry. Herein, we describe a unified approach for accessing 8-(indol-3-yl)methyl-quinolines by integrating Cp*Rh(iii)-catalyzed C(sp^3^)–H bond activation of 8-methylquinolines followed by nucleophilic cyclization with *o*-ethynylaniline derivatives. Remarkably, methoxybiaryl ynones under similar catalytic conditions delivered quinoline tethered spiro[5.5]enone scaffolds *via* a dearomative 6*-endo-dig* C-cyclization. Moreover, leveraging this method for C8(sp^2^)–H bond activation of quinoline-*N*-oxide furnished biologically relevant oxindolyl-quinolines. This reaction proceeds *via* C(sp^2^)–H bond activation, regioselective alkyne insertion, oxygen-atom-transfer (OAT) and intramolecular nucleophilic cyclization in a cascade manner. One C–C, one C–N and one C=O bond were created with concomitant formation of a quaternary center.

## Abstract 2

A Rh(iii)-catalyzed C–H indolation of 8-methylquinolines was illustrated with 2-alkynylanilines *via* sp^3^ C–H activation to achieve indole-tethered quinoline derivatives. Extension of this methodology to quinoline-*N*-oxide led to biologically relevant oxindolyl-quinolines.

## Graphical abstract

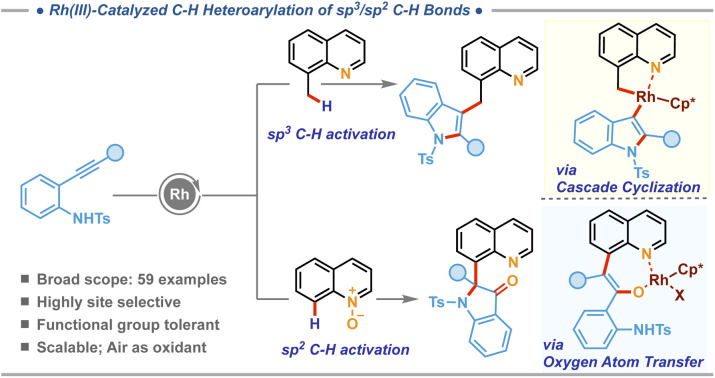

## Introduction

**Scheme 1:**
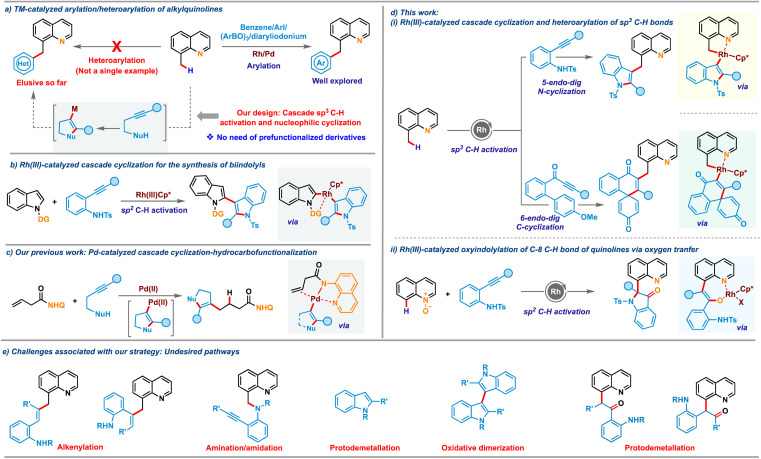
Overview of the work.

Atom-economical approaches that allow selective incorporation of one privileged heterocycle into another are highly attractive due to their ability to provide access to bis-heterocycles having significant commercial interest.^1,2^ The resulting hybrid molecules often display enhanced potential applications compared to their discrete units. Quinoline is one of the most frequently encountered heterocyclic cores in natural products,^3^ pharmaceuticals^4^ and functional materials.^5^ Consequently, the site selective modification of the quinoline nucleus has emerged as one of the most heavily studied in the realm of chemical synthesis.^6^ In particular, the substitution pattern at the C-8 position of quinoline plays a crucial role in the structure–activity-relationship (SAR) of many anti-malarial and anti-Alzheimer's drugs, as well as natural products.^7^ In this regard, conjugation of a highly celebrated heterocycle such as indole or oxindole with quinolines would unlock a library of novel conjugates having potential application as ligands, pharmaceuticals and OLEDs. Transition metal-catalyzed sp^2^ C–H activation of quinoline derivatives or sp^3^ C–H activation of 8-alkylquinolines has garnered significant attention for accessing diverse C8-functionalized quinoline frameworks.^8,9^ Along these, elegant strategies for incorporating an aryl moiety onto quinoline and its analogues have been developed in the recent past engaging functionalized precursors such as haloarenes, arylboroxines or aryldiazonium salts (Scheme 1a, right).^10^ While these arylations continue to expand, the catalyst systems exhibit limitations that restrict their scope and utility, particularly concerning the use of heterocyclic substrates. Installation of valuable heterocycles such as indole or oxindole has remained elusive with existing sp^2^/sp^3^ C–H activation of quinoline derivatives, likely due to their competing metal coordination ability and the requirement of pre-functionalized starting materials, which are difficult to access. Furthermore, the need for oxidants in cross-dehydrogenative-coupling (CDC) with C–H heterocycles often leads to side reactions such as homocoupling, which is a major concern that needs to be addressed.^11^ To overcome these limitations, we postulated a new protocol for the indolation of 8-methylquinoline by assimilating a symbiotic combination of two key areas of research in organometallic chemistry: C–H activation and nucleophilic cyclization (Scheme 1a, left), thereby bypassing the need for functionalized heterocyclic precursors.

Cascade coupling reactions involving intramolecular cyclization of nucleophile-tethered alkynyl substrates have emerged as powerful platforms for achieving complex heterocycles in a single stroke.^12^ 2-Alkynylanilines, in particular, have gained significant interest as versatile building blocks in these cascade cyclizations.^13^ Despite their widespread exploration in various cascade cyclizations, their use for synthesizing bis-heterocyclic derivatives *via* cascade C–H activation and cyclization has been scarce due to several associated challenges. For instance, alkynes are prone to alkenylation reactions, as reported by Wang and co-workers.^14^ Additionally, undesired pathways such as amidation/amination with aniline derivatives^8*d*,9*b*^ compound these challenges. To date, only a single report exists wherein directing group assisted C–H activation was integrated with nucleophilic cyclization of 2-alkynylanilines. In a seminal study, Li and co-workers in 2019 disclosed a cascade involving Rh(iii)-catalyzed C2(sp^2^)–H activation of *N*-pyrimidylindoles and intramolecular aminometallation to access 2,3′-biindole derivatives (Scheme 1b).^15^ A similar strategy *via* C(sp^3^)–H activation would be even more attractive and challenging. Building on our recent work on merging nucleophilic cyclization with hydrofunctionalization^16^ (Scheme 1c) and transition-metal catalyzed C–H activations,^17^ we envisioned that the metallacycle generated after C(sp^3^)–H or C(sp^2^)–H bond activation of 8-methylquinoline or quinoline-*N*-oxide could promote the nucleometallation of 2-alkynylaniline derivatives, providing access to a range of complex heterocyclic embedded quinolines (Scheme 1d(i) and (ii)). The key challenges (Scheme 1e) associated with the envisioned cascade are manifold. These include the possible migratory insertion of alkyne before intramolecular cyclization with the rhodacycle, which could result in alkenylation. Additionally, direct nucleophilic addition of amines to C–H activated rhodacycle may lead to C–H amination, while protonolysis of the indolyl intermediate formed after aminometalation can result in unfunctionalized indoles. Moreover, C-3 metalated indoles may undergo homocoupling to form bis-indoles, and protodemetalation after oxygen-atom-transfer (OAT) with quinoline-*N*-oxides may provide ketones, all of which could compromise reaction efficiency. In this regard, we now report C(sp^3^)–H indolation of 8-methylquinolines by merging C(sp^3^)–H activation and nucleophilic cyclization of 2-alkynylanilines, proceeding *via* 5*-endo-dig* N-cyclization addressing these challenges. Interestingly, engaging methoxybiaryl ynones instead of 2-alkynylanilines led to 6*-endo-dig* C-cyclization to realize quinoline-tethered spirocyclic cores. Leveraging this reactivity mode for C8(sp^2^)–H activation of quinoline-*N*-oxides resulted in another appealing class of bis-heterocycles: oxindole-substituted quinolines (Scheme 1d(ii)). Notably, both these oxidative processes were carried out with air/O_2_ as the terminal oxidant, adding substantial value to the protocols in terms of sustainability.

## Results and discussion

### Optimization of the reaction

We commenced our study by examining the reaction of 8-methylquinoline 1 with *N*-tosyl-2-(phenylethynyl)aniline 2 using various metal catalysts (see the ESI† for screening of different metal catalysts). Our initial screening along with the literature precedent revealed the catalytic ability of Cp*Rh(iii)-salts in facilitating the merging of C–H activation and nucleophilic cyclization for realizing sp^3^ C–H indolation. It is worth mentioning that *N*-tosyl protection was found to be crucial for the success of the transformation, as free NH_2_ or *N*-triflyl protection led to a complex mixture of products (alkenylation or C–H-amidation) instead of the desired cyclization. After extensive optimization, we identified the optimal conditions comprising 2 mol% of [Cp*Rh(iii)Cl_2_]_2_, 10 mol% of AgOTf as an additive, and 30 mol% of Cu(OAc)_2_ in DCE at room temperature to afford the desired product 3 in 80% isolated yield (Table 1, entry 1). Remarkably, the reaction proceeded with air as the terminal oxidant. The structure of 3 was unambiguously characterized by spectroscopic as well as single crystal X-ray diffraction analysis (CCDC: 2259444). While the reaction provided synthetically useful yields with as low as 1.5 equiv. 2, the yield steadily decreased with lower loadings of 2 (entries 2–3). Increasing the temperature to 70 °C had an adverse effect as the simple cyclization-protonation product (indole) was found to be more favourable over the desired C–H activation and nucleophilic cyclization (entry 4). The solvent has strongly influenced the reactivity of the cascade C–H activation and cyclization. While DCM and 1,2-dichlorobenzene produced 3 in inferior yields (entries 5–6), no product was isolated when DCE was replaced by other solvents such as CH_3_CN, THF, 1,4-dioxane or MeOH (entry 7). The use of 1.0 equiv. of either AgOAc or AgOPiv instead of Cu(OAc)_2_ proved to be less effective (entries 8 & 9). Control experiments revealed that 3 was not formed in the absence of either AgOTf or Cu(OAc)_2_ (entries 10 and 11). Similar yields were observed when the reaction was performed with either 1 equiv. of Cu(OAc)_2_ or using O_2_ balloon (entries 12–13). However, the reaction under N_2_ yielded a poor amount of 3 indicating the importance of air as the terminal oxidant (entry 14).

**Table 1:** Optimization of the reaction parameters

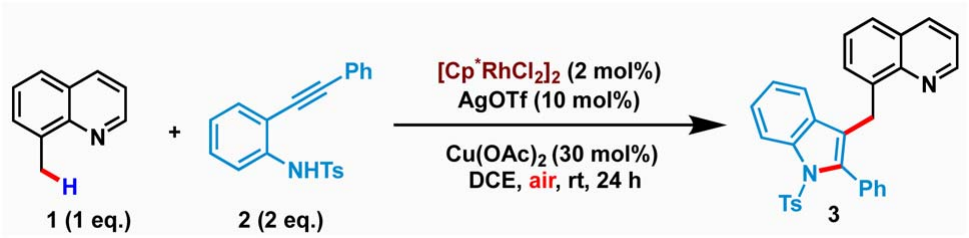		
Entry	Deviation from standard conditions	Yield (%)^a^
1	None	82 (80)^b^
2	1.5 equiv. of 2	74
3	1.1 equiv. of 2	63
4	Reaction at 70 °C	54
5	DCM instead of DCE	32
6	1,2-Dichlorobenzene instead of DCE	25
7	CH_3_CN, THF, 1,4-dioxane, and MeOH instead of DCE	n.r.
8	AgOAc (1 equiv.) instead of Cu(OAc)_2_	44
9	AgOPiv (1 equiv.) instead of Cu(OAc)_2_	42
10	Without AgOTf	n.r.
11	Without Cu(OAc)_2_	n.r.
12	1 equiv. of Cu(OAc)_2_	80
13	Reaction under O_2_	82
14	Reaction under N_2_	20

a ^1^H NMR of the crude reaction mixture was obtained with 1,3,5-trimethoxybenzene as an internal standard.

b Isolated yield.

### Substrate scope of sp^3^–C–H indolation

**Scheme 2:**
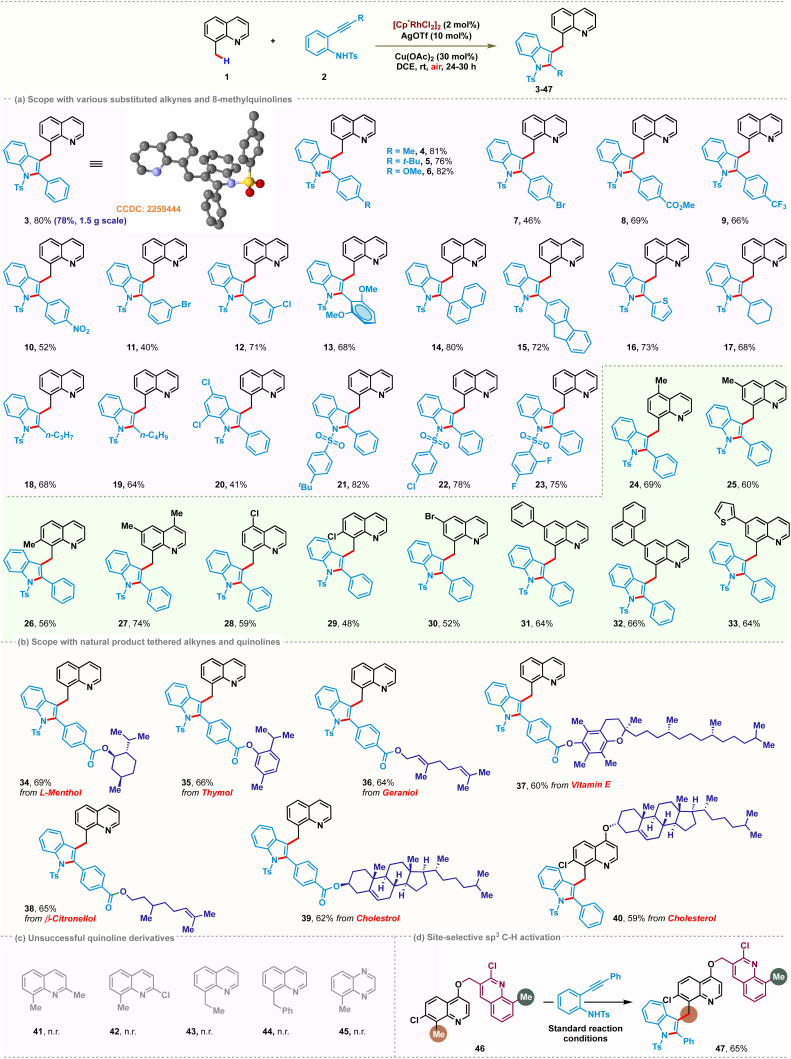
Scope of the C(sp^3^)–H indolation of 8-methylquinolines.

After establishing the standard reaction conditions, the scope of the Rh-catalyzed C(sp^3^)–H indolation was demonstrated with a series of 2-alkynylanilines (Scheme 2a). The presence of electron-donating or -withdrawing substituents at the *para*- or *meta*-position of the aryl ring afforded the desired products 4–12 with yields ranging from 40–82%. *ortho*-Dimethoxy substitution on the ethynyl phenyl ring of aniline was well tolerated to give the corresponding product 13 in 68% yield. Replacing the phenyl group with naphthyl, fluorenyl or thienyl resulted in the formation of 14–16 in 80%, 72%, and 73% yields, respectively. Besides aromatic substituents, aliphatic substituents at the alkyne terminus also furnished the desired products 17–19 in moderate to good yields (64–68%). However, the reaction with substitution on the aniline ring was rather sluggish and gave a low yield of 20 (41%). Variation of the *N*-sulfonyl group afforded 21–23 in 82%, 78% and 75% yields, respectively, which confirmed the effectiveness of different types of *N*-sulfonyl protecting groups.

Next, we investigated the generality with respect to different 8-methylquinolines. Methyl and halogen substituents at various positions of 8-methylquinoline delivered the desired products 24–30 in good yields ranging from 48% to 74%. Phenyl, naphthyl or thienyl substitution at the 6-position of 8-methylquinoline was also found to be compatible, providing the corresponding products 31–33 with yields up to 66%. Our developed cascade strategy set the stage for the late-stage modification with a range of natural products (Scheme 2b). Alkynes and 8-methylquinolines tethered with various natural products such as (*L*)-menthol, thymol, geraniol, vitamin E, citronellol and cholesterol successfully followed the reaction pathway and provided the corresponding bio conjugates 34–40 in good yields up to 69%. Several limitations were also identified. C2-Substituted 8-methylquinolines failed to show any reactivity, probably due to increased steric hindrance for Rh-coordination with the nitrogen center (Scheme 2c). Taking advantage of this, we next investigated substrate 46 as a model substrate having two different potential reaction sites (C2–Cl and C7–Cl). We envisioned that this could be exploited in a sterically selective manner (Scheme 2d). Indeed, when 46 was subjected to the optimized reaction conditions, pleasingly monoindolated product 47 was obtained in 65% yield, with the quinoline scaffold having 2-Cl substitution unperturbed.

**Scheme 3:**
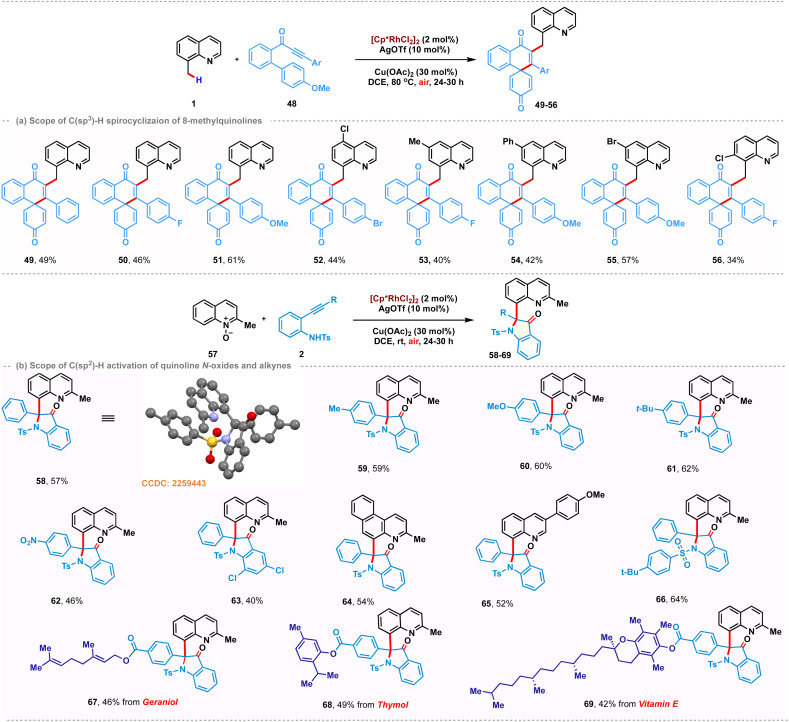
Scope of the reaction with biaryl-ynones and quinoline-*N*-oxides.

Due to the appealing biological properties associated with functionalized spirocycles,^18^ the installation of spirocycles *via* cascade dearomative spirocyclization on the C(sp^3^)–H bond of 8-methylquinoline was also examined. We hypothesized that metal coordination of methoxybiaryl-ynone might promote dearomative 6*-endo-dig* C-cyclization to access the desired quinoline tethered spiro[5.5]enone scaffolds in a tandem manner (Scheme 3a). Although there are a few examples of radical dearomative spirocyclization of methoxybiaryl-ynones,^19^ to our knowledge, there is no report on metal-catalyzed cascade C(sp^3^)–H activation/spirocyclization with methoxybiaryl-ynone as a coupling partner. Thus, we initiated our investigation with the model reaction between 1 and methoxybiaryl-ynone 48 under optimized reaction conditions. We were delighted to observe that the reaction afforded the desired product 49 in 49% yield at 80 °C. As depicted in Scheme 3a, the transformation proceeded well for the substrates bearing different functional groups on the aryl ring, such as –F and -OMe groups, affording the corresponding products 50 and 51 with yields of 46% and 61%, respectively. The substitutions on the 8-methylquinoline scaffold, such as halogen, methyl and phenyl, were compatible and the expected products 52–55 were observed in moderate yields. However, substitution at the 7-position had a negative effect on this transformation, resulting in 56 in 34% yield. Intrigued by our success with 8-methylquinoline derivatives, we then sought to scrutinize the reactivity of quinoline-*N*-oxide as a coupling partner under similar catalytic conditions (Scheme 3b). 2-Methylquinoline-*N*-oxide 57 was chosen as the model substrate. Interestingly, a cascade sequence consisting of C(sp^2^)–H activation, OAT followed by intramolecular cyclization, was observed to deliver (2-methylquinolin-8-yl)-2-phenyl-1-tosylindolin-3-one 58 in 57% yield.

The structure of 58 was confirmed through spectroscopic analysis and single-crystal X-ray diffraction (CCDC: 2259443). The relatively lower yield is attributed to the regiomeric insertion of electronically biased alkynes with the rhodacycle. The desired product arises from the regioselective insertion of the alkyne with rhodium towards the aniline terminus, while the alternative mode produces a by-product observed in 10%. It is noteworthy that different substitutions on the ethynyl phenyl ring of aniline 59–63 provided the desired products in good yields. However, substitution on the aniline ring led to a lower yield of 40% for 63. The reaction also exhibited compatibility with 3-methyl benzo-[*f*]quinoline-*N*-oxide and 6-(4-methoxyphenyl) quinoline-*N*-oxide, delivering 64 and 65 with yields of 54% and 52%, respectively. Fascinatingly, alkynes tethered with natural products such as thymol, geraniol and vitamin E reacted smoothly under the standard conditions for this transformation to afford 67–69 with moderate yields.

**Scheme 4:**
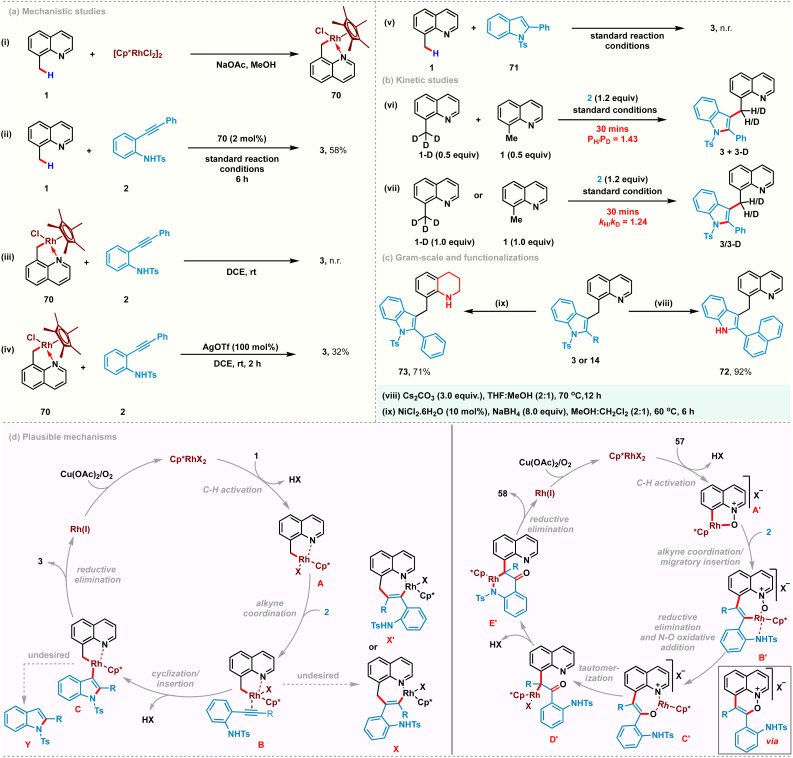
Mechanistic studies, gram-scale, post-functionalizations and plausible mechanisms.

To gain some insights into the reaction mechanism, a series of experiments were conducted. Rhodacyclic complex 70 was prepared from cyclometalation of 8-methylquinoline 1 and used as a catalyst precursor instead of [Cp*RhCl_2_]_2_ under the standard reaction conditions, yielding 3 in 58% yield. These studies indicate its catalytic competence and relevance in C–H activation (Scheme 4a(i) and (ii)). A stoichiometric reaction with complex 70 did not afford the desired product in the absence of a silver additive (Scheme 4a(iii)). However, using stoichiometric AgOTf successfully catalyzed the reaction, suggesting the plausible intermediacy of a cationic cyclometalated complex in the catalytic cycle (Scheme 4a(iv)). Furthermore, the use of 2-phenyl-1-tosyl-1H-indole 71 as a coupling partner proved to be futile, confirming that the C–H activation is the primary step and the cationic rhodacycle is the active species for this transformation (Scheme 4a(v)). Additionally, kinetic isotope experiments with 8-(methyl-*d*_3_)quinoline were performed (Scheme 4b(vi) and (vii)). Parallel and competition experiments with 1, 1-D with 2 gave kinetic isotope effect values of 1.43 and 1.24, respectively. Both these experimental KIE values suggest that the C–H bond cleavage is likely not involved in the rate-determining step. To highlight the practicality of this transformation, gram-scale synthesis of 3 was carried out and obtained 78% yield of 3, starting with 1.5 g of 1. Subsequently, the tosyl group of 3 was easily removed in nearly quantitative yield to provide 72 in 92% yield. Furthermorw, selective reduction of the quinoline ring was performed using NiCl_2_·6H_2_O (10 mol%) and NaBH_4_ (8.0 equiv.) to afford tetrahydroquinoline derivative 73 in 71% yield.

Based on our mechanistic studies, the possible mechanisms were proposed for the present catalytic transformations (Scheme 4d). The first step likely involves a C(sp^3^)–H activation process, thus affording the intermediate A. Coordination of the incoming alkyne promotes the subsequent outer-sphere intramolecular nucleometallation to give Rh(iii)-alkenyl intermediate C. Reductive elimination from C furnishes the desired coupling product 3 and Rh(i)-species, which is then reoxidized to enter a new catalytic cycle. We proposed a similar catalytic cycle for C(sp^2^)–H activation of quinoline-*N*-oxide to generate 5-membered rhodacycle A′, which upon subsequent alkyne co-ordination and regioselective migratory insertion furnishes the 7-membered rhodacycle B′. Reductive elimination of C–O bonds followed by oxidative addition of Rh(i) between N–O bonds generates the O-bound enolate C′. The oxindolated product 58 is released by tautomerization and subsequent metal promoted cyclization.

## Conclusions

In summary, we have devised a Rh(iii)-catalyzed indolation of 8-methylquinolines involving challenging C(sp^3^)–H bond activation and subsequent nucleophilic cyclization. In addition, we have demonstrated a straightforward and effective methodology for synthesizing (quinolin-8-yl)-1-tosylindolin-3-one derivatives using quinoline-*N*-oxide as a coupling partner under similar catalytic conditions. The reaction sequence encompasses C(sp^2^)–H bond activation, regioselective alkyne insertion, oxygen-atom-transfer (OAT) and intramolecular nucleophilic cyclization in a seamless domino fashion. The reactions demonstrated notable generality, accommodating a wide array of substituted 8-methylquinolines/quinoline-*N*-oxides, and *o*-alkynylanilines were successfully employed in the reaction. The utility of the developed catalytic approach was underscored by facile scale-up synthesis and late-stage modification of drugs and natural products. Mechanistic studies, including kinetic isotope effect (KIE) and competition experiments, have provided support for the proposed mechanism. Further efforts on metal-catalyzed cascade sp^3^ C–H activation/nucleophilic cyclization of other sp^3^ C–H bonds are currently underway in our laboratory.

## Data availability

Detailed synthetic procedures and complete characterization data for all new compounds can be found in the ESI.†

## Author contributions

A. K. C. and R. K. S. designed and conducted all experiments and characterized the novel compounds. A. K. C. and C. M. R. V. wrote the manuscript. C. M. R. V. directed the research.

## Conflicts of interest

There are no conflicts to declare.

## Supplementary Material

SC-015-D3SC06955A-s001

SC-015-D3SC06955A-s002
