# Supplementary material for: Rh(iii)-catalyzed sp3/sp2–C–H heteroarylations via cascade C–H activation and cyclization
Source: Chem Sci. 2024 Mar 27;15(17):6544–51. doi: 10.1039/d3sc06955a (PMC11062110; doi:10.1039/d3sc06955a)
Supplement: SC-015-D3SC06955A-s001 [file SC-015-D3SC06955A-s001.pdf]

Electronic Supplementary Material (ESI) for Chemical Science.
This journal is © The Royal Society of Chemistry 2024

Rh(III)-Catalyzed sp^3/sp^2–C-H Heteroarylations *via* Cascade C-H Activation and Cyclization

Atul K. Chaturvedi, Rahul K. Shukla, Chandra M. R. Volla*

Department of Chemistry, Indian Institute of Technology Bombay

Powai, Mumbai-400076, India.

E–mail: chandra.volla@chem.iitb.ac.in

Supporting Information

Table of Contents

1. General considerations and reagent information S2

2. General procedure for the synthesis of starting materials S2

3. Optimization details S5

4. General synthetic procedures S6

5. Gram Scale synthesis and late-stage functionalization S7

6. Control Experiments S8

7. Spectroscopic data for new compounds S12

8. References S50

9. 1H and 13C NMR spectra of the compounds S51

1) General considerations reagent information. Unless otherwise stated, all reactions were carried out under air atmosphere in screw cap reaction tubes. All the solvents were bought from Aldrich in sure-seal bottle and were used as received. $[RhCp^*Cl_2]_2$, another reagent was bought from Aldrich. For column chromatography, silica gel (100–200 mesh) from Finar Co. was used. A gradient elution using petroleum ether and ethyl acetate was performed based on Merck aluminium TLC sheets (silica gel 60F254). All starting materials were prepared according to the reported procedures in the literatures and the analytical data are in accord with the literature.

Analytical information. All isolated compounds are characterized by ^{1}H NMR, ^{13}C NMR spectroscopy. In addition, all the compounds are further characterized by HRMS Copies of ^{1}H NMR and ^{13}C NMR can be found in the supporting information. Nuclear magnetic resonance spectra were recorded either on a Bruker 500 or a 400 MHz instrument. All ^{1}H NMR experiments are reported in units, parts per million (ppm), and were measured relative to the signals for residual chloroform (7.26 ppm) in the deuterated solvent, unless otherwise stated. All ^{13}C NMR spectra was reported in ppm relative to deuteron chloroform (77.16 ppm), unless otherwise stated, and all were obtained with ^{1}H decoupling.

2) General procedure for synthesis of starting material

a) Synthesis of *N*-protected 2-alkynylbenzenamine[1]

Step-1st

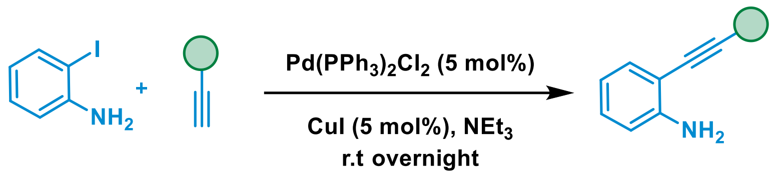

Adapting a known procedure, CuI (43.5 mg, 0.23 mmol, 5 mol %), $PdCl_2(PPh_3)_2$ (160.2 mg, 0.23 mmol, 5 mol %) and alkyne (5.70 mmol, 1.25 equiv.) were added to a stirred solution of 2-iodoaniline (1.00 g, 4.566 mmol, 1.0 equiv.) in Et_3N (12.7 mL, 91.32 mmol, 20 equiv.), at room temperature. The mixture was allowed to react for 12–18 h (until starting material get consumed), concentrated under reduced pressure, and submitted to column chromatography on silica gel to afford the compound.

Step-2nd

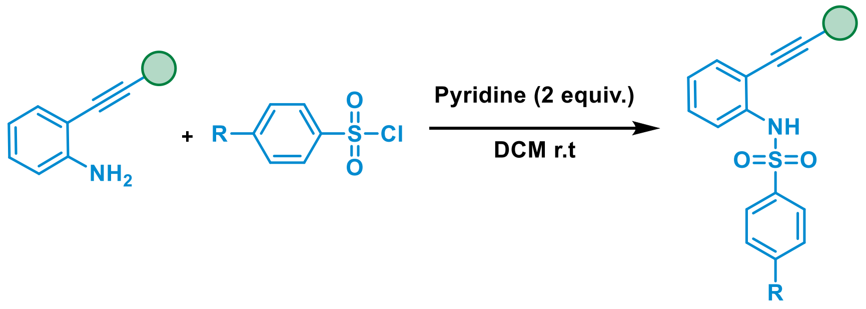

To a solution of 2-(ethynyl)benzenamine (1 equiv.) and pyridine (2 equiv.) in 20 mL of dichloromethane was added 4-methylbenzene-1-sulfonyl chloride or substituted benzene-1-sulfonyl chloride (1 equiv.) at 0 °C, then the reaction solution was warmed to room temperature. After the reaction was complete (about 10 h) as monitored by TLC, water was added, and the mixture was extracted with ether. The combined organic phase was washed with brine, dried over anhydrous Na_2SO_4. The solvent was evaporated under the reduced pressure and the residue was purified by chromatography on silica gel to afford the compound.

b) Procedure for the synthesis of natural product containing *N*-protected 2-alkynylbenzenamine[2]

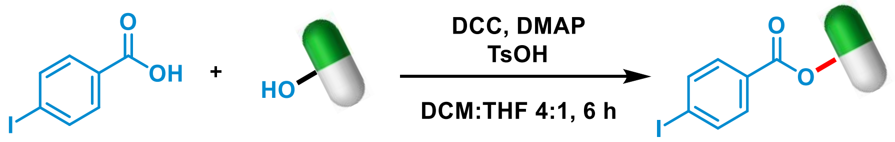

Into a 100 mL roundbottom flask equipped with a stirring bar were dissolved (1 equiv) of 4-iodobenzoic acid, (1.5 equiv) of DCC, (0.2 equiv) of DMAP, and (0.2 equiv) of TsOH in dry DCM/THF mixture (4:1 v/ v). The solution was cooled to 0°C with an ice bath, into which (2 mmol) of alcohal dissolved in 10 mL of DCM/THF (4:1 v/v) was added dropwise. The reaction mixture was stirred overnight. After filtration, the solution was concentrated by a rotary evaporator and purified by column chromatography (silica gel).

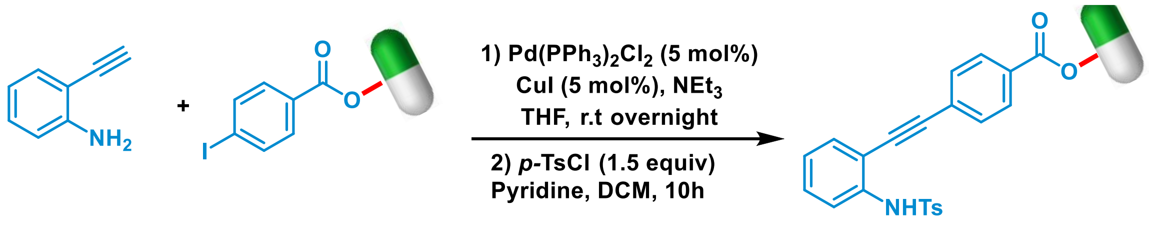

A mixture of substituted iodobenzene (1 equiv.), CuI (5 mol %), $PdCl_2(PPh_3)_2$ (5 mol %) and Et_3N (4 equiv.) in THF was stirred under N_2 atmosphere for 20 min. Then 2-ethynylaniline (1.25 equiv.) was added very slowly. The resulting reaction mixture was stirred at room temperature for 12–18 h (until starting material get consumed) under N_2 atmosphere. After the removal of triethylamine, the reaction mixture was poured in water (50 mL) and extracted with chloroform (3 × 40 mL). The combined chloroform layer was washed with water and dried over anhydrous Na_2SO_4. After the removal of solvent, the residue was chromatographed over silica gel using petroleum ether/ethyl acetate (95/5, V/V) as eluent, affording desired product.

The coupling product aniline was dissolved in dichloromethane (20 mL), pyridine (2 equiv) and 4-methylbenzene-1-sulfonyl chloride (1.5 equiv.) was added at 0 ^{o}C. The reaction solution was warmed to room temperature. After the reaction was complete (about 10 h) as monitored by TLC, water was added, and the mixture was extracted with ether. The combined organic phase was washed with brine, dried over anhydrous Na_2SO_4. The solvent was evaporated under the reduced pressure and the residue was purified by chromatography on silica gel to afford the compound.

c) Procedure for the synthesis of quinolyl ether

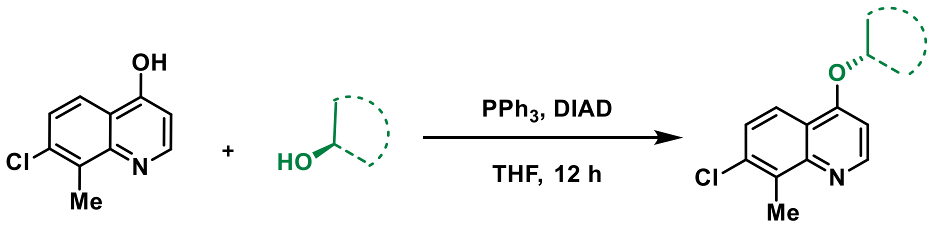

A three-necked, round-bottomed flask is equipped with a stirring bar, nitrogen inlet and rubber septum. The flask is charged with alcohol (1 equiv.), 7-chloro-8-methylquinolin-4-ol (1.2 equiv.), triphenylphosphine (PPh_3) (1.5 equiv.) and dry tetrahydrofuran (0.1 M). The flask is immersed in an ice bath, diethyl azodicarboxylate (1.5 equiv.) is added dropwise at a rate such that the temperature of the reaction mixture is maintained below 10°C. Upon completion of the addition, the flask is removed from the ice bath and the solution is allowed to stir at room temperature overnight (12–14 hr). The reaction mixture is diluted with ether, and washed twice with saturated aqueous sodium bicarbonate solution. The combined organic phase was dried over sodium sulphate. The solvent was evaporated under the reduced pressure and the residue was purified by chromatography on silica gel to afford the compound.

3) Optimization of the reaction conditions[a]

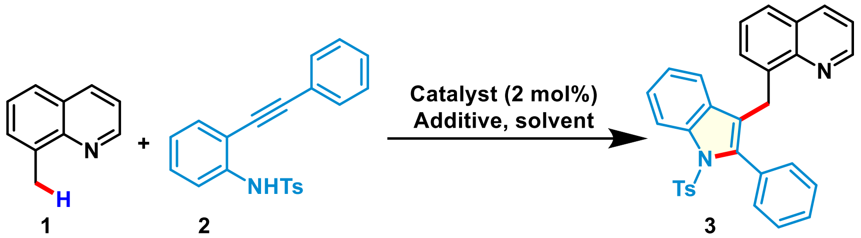

Entry[a]	Catalyst	Additive 1 (mol%)	Additive 2 (mol%)	T(°C)	Solvent	Yield (%)*b*
1	$Pd(OAc)_2$	$Cu(OAc)_2$ (30)	-	rt-80	MeCN	0
2	$Pd(OAc)_2$	$Cu(OAc)_2$ (30)	-	rt-80	HFIP	0
3	Ru(*p*-cymene)Cl_2	$Cu(OAc)_2$ (30)	AgOTf (10)	rt	DCE	0
4	Ru(*p*-cymene)Cl_2	NaOAc (30)	AgOTf (10)	rt	DCE	0
5	Ru(*p*-cymene)Cl_2	Ag_2CO_3(30)	AgOTf (10)	rt	DCE	trace
6	Ru(*p*-cymene)Cl_2	Ag_2CO_3(30)	AgOTf (10)	80	DCE	20
7	Ru(*p*-cymene)Cl_2	Ag_2CO_3(30)	AgOTf (10)	80	MeOH	trace
8	$[Cp^*Co(CO)I_2]_2$	$AdCO_2H$(30)	AgOTf (10)	rt-80	TFE	0
9	$[Cp^*Co(CO)I_2]_2$	$Cu(OAc)_2$ (30)	AgOTf (10)	rt-80	TFE	0
10	$[Cp^*RhCl_2]_2$	$Cu(OAc)_2$ (30)	-	rt-80	DCE	0
11	$[Cp^*RhCl_2]_2$	$Cu(OAc)_2$ (30)	AgOTf (10)	rt	DCE	63
12	$[Cp^*RhCl_2]_2$	$Cu(OAc)_2$ (30)	AgOTf (10)	70	DCE	54
13	$[Cp^*RhCl_2]_2$	$Cu(OAc)_2$ (30)	AgOTf (10)	rt	DCM	32
14	$[Cp^*RhCl_2]_2$	$Cu(OAc)_2$ (30)	AgOTf (10)	rt	DCB	25
15	$[Cp^*RhCl_2]_2$	$Cu(OAc)_2$ (30)	AgOTf (10)	rt	MeCN	nr
16	$[Cp^*RhCl_2]_2$	$Cu(OAc)_2$ (30)	AgOTf (10)	rt	THF	nr
17	$[Cp^*RhCl_2]_2$	$Cu(OAc)_2$ (30)	AgOTf (10)	rt	Dioxane	nr
18	$[Cp^*RhCl_2]_2$	$Cu(OAc)_2$ (30)	AgOTf (10)	rt	MeOH	nr
19[c]	$[Cp^*RhCl_2]_2$	$Cu(OAc)_2$ (30)	AgOTf (10)	rt	DCE	74
**20[d]**	**$[Cp^*RhCl_2]_2$**	**$Cu(OAc)_2$ (30)**	**AgOTf (10)**	**rt**	**DCE**	**82**
21[d]	$[Cp^*RhCl_2]_2$	AgOAc (100)	AgOTf (10)	rt	DCE	44
22[d]	$[Cp^*RhCl_2]_2$	AgOPiv (100)	AgOTf (10)	rt	DCE	42

[a]All reactions were carried out using substituted **1** (1 equiv.), **2** (1.1 equiv.), catalyst (2 mol%), [b]NMR Yield, [c]**2** (1.5 equiv.) [d]**2** (2.0 equiv.)

4) General procedure for cascade C-H activation and nucleophilic cyclization

a) General procedure for Sp³ C-H activation and nucleophilic (C-N) cyclization

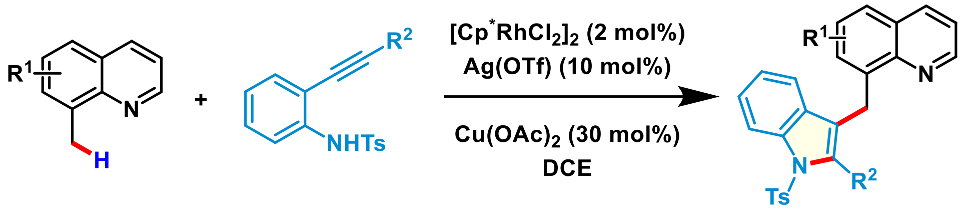

To an oven dried screw-cap reaction tube equipped with stir bar, 8-methylquinoline (0.1 mmol), *o*-ethynyl aniline (0.2 mmol), $[Cp^{*}RhCl_2]_2$ catalyst (2 mol%), $Cu(OAc)_2$ (0.03 mmol) and Ag(OTf) (0.01 mmol) were added sequentially, followed by DCE (1 mL). The reaction mixture was stirred at room temperature for 24–30 hrs (until consumption of starting material). The completion of the reaction was confirmed by checking TLC under UV detector. Then, the organic phase was evaporated under reduced pressure and the product was purified by using silica-gel column chromatography (eluent: Hexane/Ethyl acetate =9/1)

b) General procedure for cascade Sp³ C-H activation and nucleophilic (C-C) cyclization

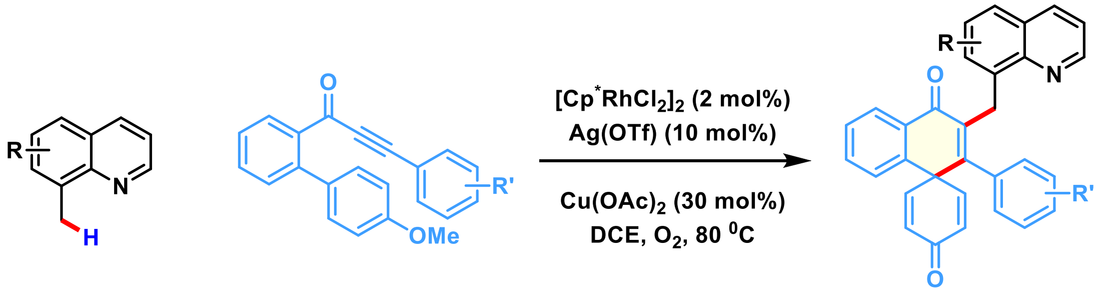

To an oven dried screw-cap reaction tube equipped with stir bar, 8-methylquinoline (0.1 mmol), methoxybiaryl ynone (0.2 mmol), $[Cp^{*}RhCl_2]_2$ catalyst (2 mol%), $Cu(OAc)_2$ (0.03 mmol) and Ag(OTf) (0.01 mmol) were added sequentially, followed by DCE (1 mL). The reaction mixture was stirred at 80 ^{0}C under O_2 for 14-16 hrs. The completion of the reaction was confirmed by checking TLC under UV detector. Then, the organic phase was evaporated under reduced pressure and the product was purified by using silica-gel column chromatography (eluent: Hexane/Ethyl acetate =7/3)

c) General procedure for cascade Sp^2 C-H activation, OAT and nucleophilic cyclization

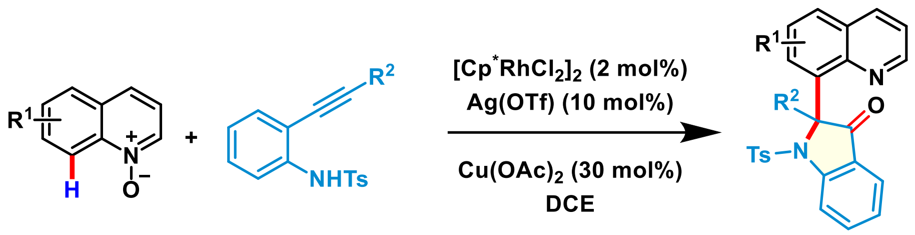

To an oven dried screw-cap reaction tube equipped with stir bar, quinoline *N*-oxide (0.1 mmol), *o*-ethynyl aniline (0.2 mmol), [Cp*RhCl$_2$]$_2$ catalyst (2 mol%), Cu(OAc)$_2$ (0.03 mmol) and AgOTf (0.01 mmol) were added sequentially, followed by DCE (1 mL). The reaction mixture was stirred at room temperature for 30–36 hrs. The completion of the reaction was confirmed by checking TLC under UV detector. Then, the organic phase was evaporated under reduced pressure and the product was purified by using silica-gel column chromatography (eluent : Hexane/Ethyl acetate =8/2).

5. Gram scale synthesis and late stage functionalization

a. Gram scale

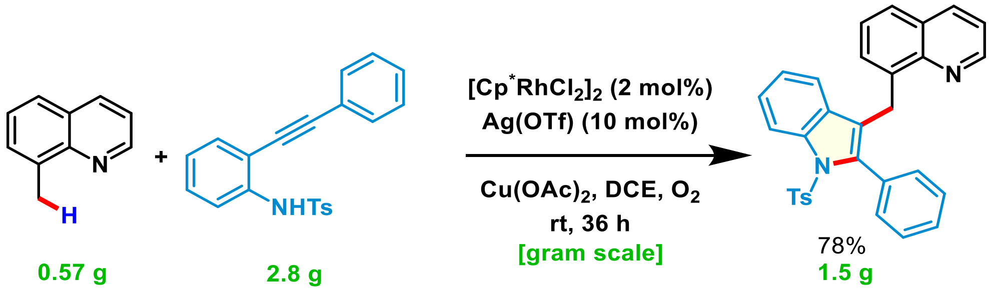

A mixture of 8-methylquinoline (4.0 mmol, 572 mg), *o*-ethynyl aniline (8.0 mmol), [Cp*RhCl$_2$]$_2$ catalyst (2 mol%), Cu(OAc)$_2$ (0.03 mmol) and Ag(OTf) (0.01 mmol) were added sequentially, followed by DCE (15 mL). The reaction mixture was stirred at room temperature under oxygen for 30 hrs. The completion of the reaction was confirmed by checking TLC under UV detector. Then, the organic phase was evaporated under reduced pressure and the product was purified by using silica-gel column chromatography (eluent: Hexane/Ethyl acetate =9/1) affording **3** (1.5gm, 78%).

b. Removal of directing group[3]

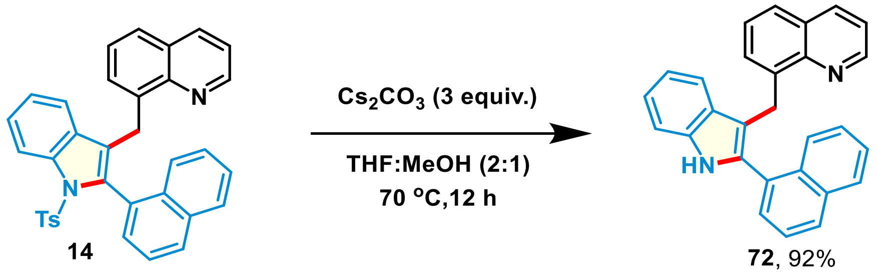

In an oven dried reaction tube 0.1 mmol of **14** was taken. Then cesium carbonate (3 equiv) in THF:MeOH (2:1) 1.5 mL was added to it. The mixture was stirred in a preheated oil bath at 70 °C for 12 hours. After completion reaction was cooled to room temperature and evaporated under vacuum. Pure product of **72** with 92% yield was isolated through silica gel column chromatography (mesh 100–200). Eluent: ethyl acetate / petroleum ether (5:95 v/v).

c. Reduction of 3[4]

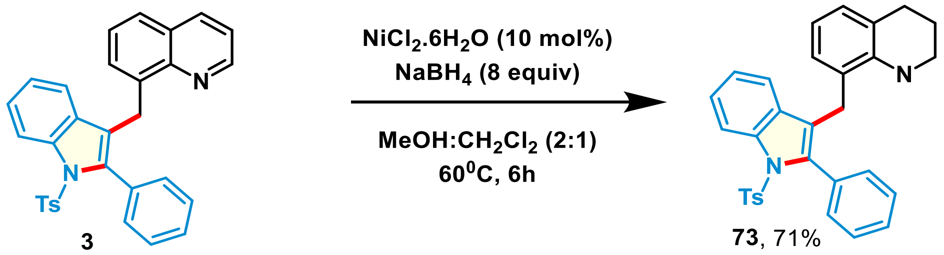

In an oven dried reaction tube 0.1 mmol of **3** was taken in 2:1 mixture of MeOH:CH_2Cl_2 (1.5 mL). Then $NiCl_2.6H_2O$ (10 mol%, 0.01 mmol) and sodium borohydride (8 equiv, 0.8 mmol) was added to it. The mixture was stirred in a preheated oil bath at 70 °C for 12 hours. After completion reaction was cooled to room temperature and evaporated under vacuum. Pure product **73** with 71% yield was isolated through silica gel column chromatography (mesh 100–200). Eluent: ethyl acetate / petroleum ether (5:95 v/v).

6. Control Experiments

a. Preparation of Intermediate 70[4]

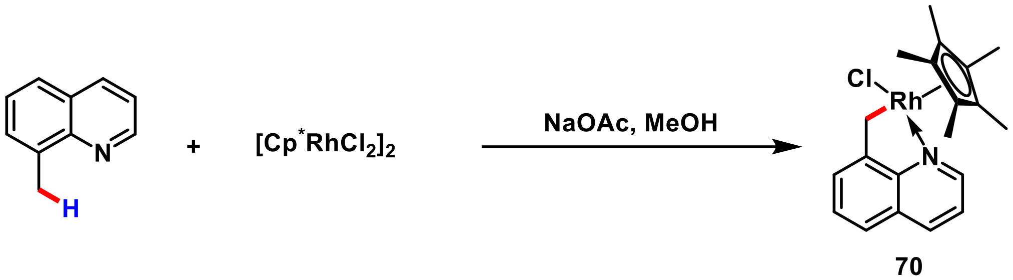

Intermediate **70** was prepared by heating the mixture of 8-methyl-quinoline (85.9 mg, 0.6 mmol), $[Cp^*RhCl_2]_2$ (18.6 mg, 0.03 mmol, 5.0 mol %), NaOAc (24.6 mg, 0.3 mmol) and

MeOH (1 mL) in a reaction tube for 6h. The reaction mixture was then concentrated under vacuum. The product was purified by column chromatography on silica gel (eluent: EtOAc/petroleum ether = 1: 2).

b. Preparation of deuterated quinoline[5]

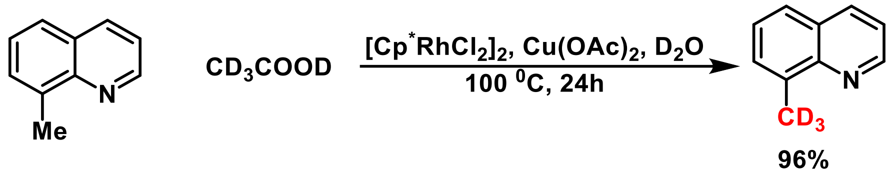

A mixture of 8- methylquinoline (**1a**) (172.0 mg, 1.2 mmol), $[RhCp^{*}Cl_2]_2$ (18.4 mg, 0.03 mmol, 2.5 mol %), CD_3COOD (216.4 mg, 3.6 mmol, 300 mol %), $Cu(OAc)_2$ (436.0 mg, 2.4 mmol, 200 mol %) and D_2O (4 mL) in 10 mL reaction tube was heated at 100 ^{0}C under air for 48 h. After cooling down, the reaction mixture was extracted with EtOAc (3x30 mL). The organic layer was dried over MgSO4 and concentrated in vacuo. The residue was purified by flash column chromatography (n-hexanes/EtOAc = 10:1) to afford 1a-d3 (168.4 mg, 95% D) in 96% yield.

c. General procedure for the KIE experiments:

(i) P_H/P_D

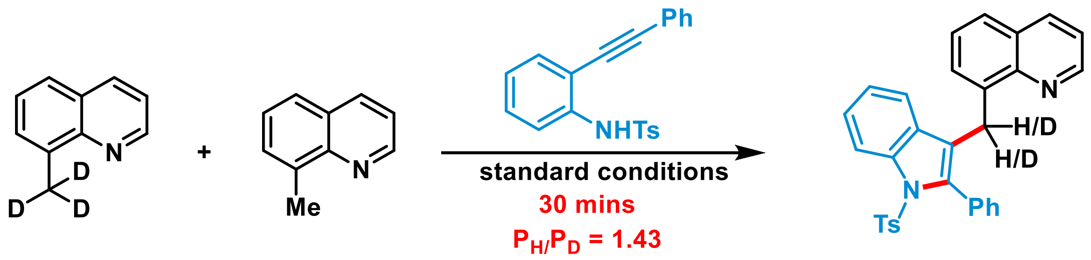

A mixture of substituted 8-methyl-quinoline (0.1 mmol, 1 equiv.), or 8-methyl-quinoline-D3 (0.1 mmol, 1 equiv.), 4-methyl-N-(2-(phenylethynyl)phenyl)benzenesulfonamide (0.24 mmol, 2.4 equiv), $[Cp^{*}RhCl_2]_2$ (5.0 mol %), and $Cu(OAc)_2$ (30 mol %) were weighted in a same reaction tube equipped with a stir bar. DCE (2.0 mL) was added and the mixture was stirred at room temperature for 30 min. The solvent was evaporated under reduced pressure and the residue was absorbed to small amounts of silica. The purification was performed by column chromatography on silica gel (eluent: EtOAc/petroleum ether = 5:95).

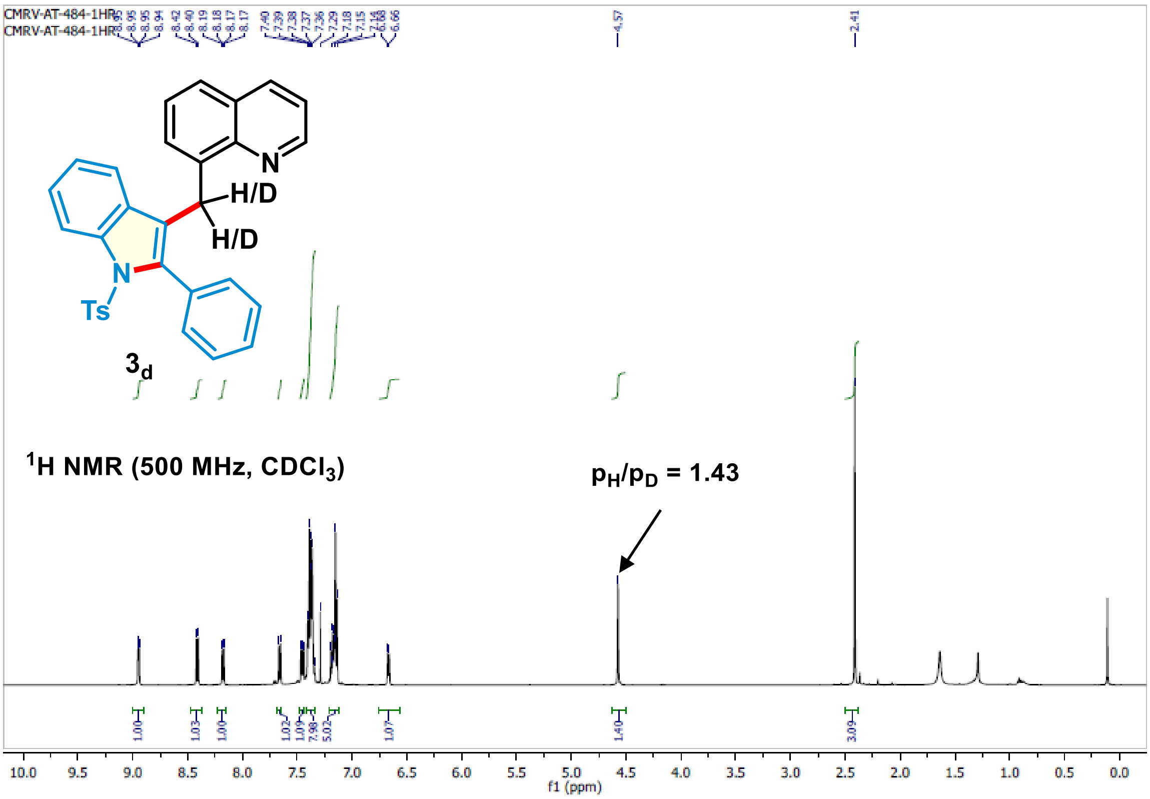

(ii) K_H/K_D

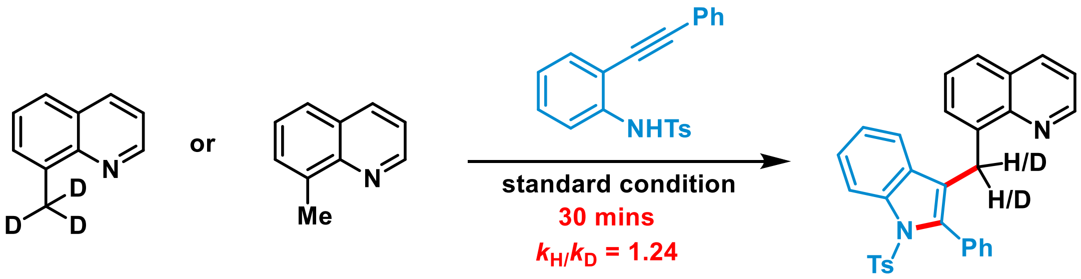

A mixture of substituted 8-methyl-quinoline (0.1 mmol, 1 equiv.), or 8-methyl-quinoline-D3 (0.1 mmol, 1 equiv.), 4-methyl-N-(2-(phenylethynyl)phenyl)benzenesulfonamide (0.12 mmol, 1.2 equiv), $[Cp^*RhCl_2]_2$ (2.0 mol %), and $Cu(OAc)_2$ (30 mol %) were weighted in a reaction tube equipped with a stir bar. DCE (2.0 mL) was added and the mixture was stirred at room temperature for 30 min. Afterwards, the two independent reactions were poured into a same round flask, the solvent was evaporated under reduced pressure and the residue was absorbed to small amounts of silica. The purification was performed by column chromatography on silica gel (eluent: EtOAc/petroleum ether = 5:95).

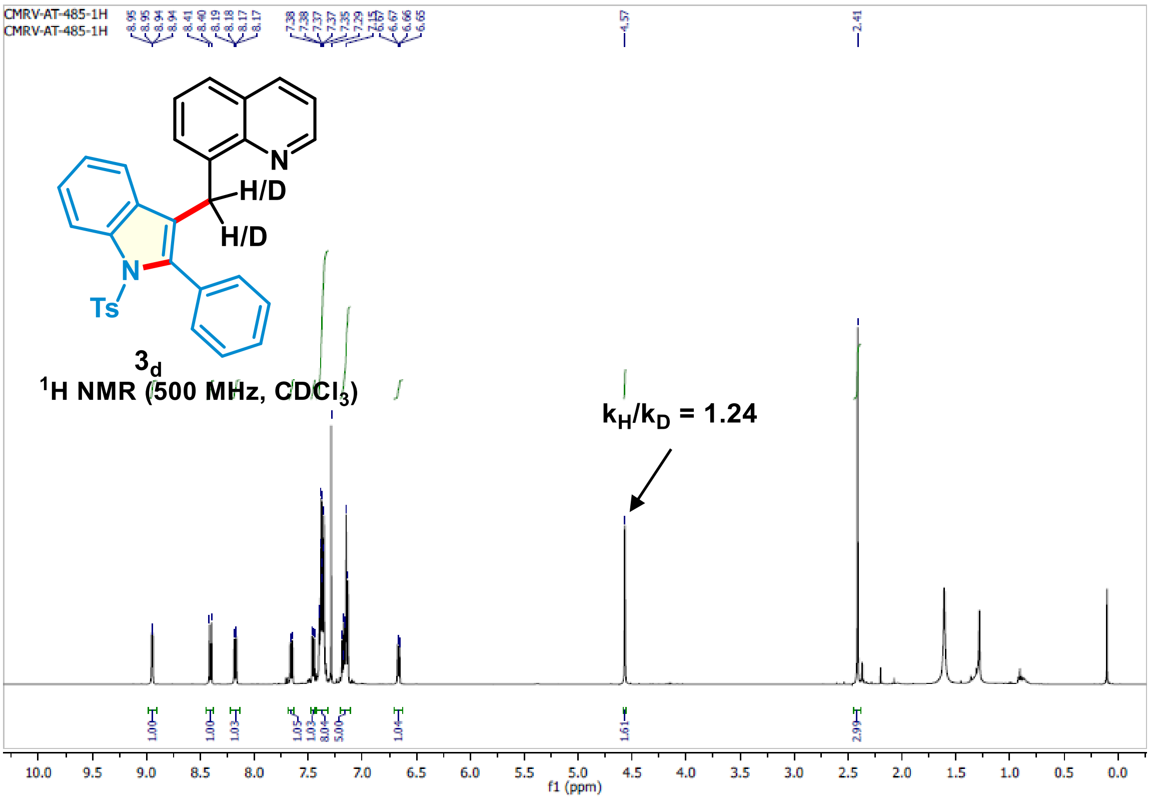

CMRV-AT-485-1H
CMRV-AT-485-1H
H/D
H/D
N
N
Ts
3d
1H NMR (500 MHz, CDCl3)
kH/kD = 1.24
f1 (ppm)

7. Spectroscopic data for new compounds

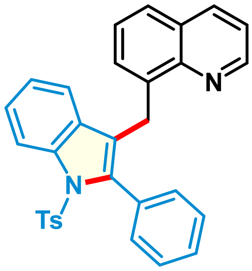

3, 80%

8-((2-phenyl-1-tosyl-1*H*-indol-3-yl)methyl)quinolone (3)

39.1 mg, 80% yield, white solid.

***R_f*:** 0.50 (hexane/ethyl acetate, 9:1 v/v).

^{1}H NMR (500 MHz, CDCl$_3$) δ 8.95 (dd, *J* = 4.2, 1.7 Hz, 1H), 8.41 (d, *J* = 8.4 Hz, 1H), 8.17 (dd, *J* = 8.3, 1.7 Hz, 1H), 7.66 (d, *J* = 8.0 Hz, 1H), 7.45 (dd, *J* = 8.3, 4.2 Hz, 1H), 7.42 – 7.33 (m, 8H), 7.21 – 7.12 (m, 5H), 6.67 (dd, *J* = 7.1, 0.9 Hz, 1H), 4.57 (s, 2H), 2.41 (s, 3H).

^{13}C NMR (126 MHz, CDCl$_3$) δ 149.50, 146.67, 144.53, 138.93, 138.28, 138.16, 136.48, 134.90, 131.88, 131.42, 131.04, 129.31, 128.64, 128.29, 127.86, 127.62, 127.12, 126.28, 126.23, 125.17, 124.36, 123.13, 121.24, 120.06, 116.99, 25.65, 21.75.

HRMS (ESI-TOF) m/z: $[M+H]^+$calcd for $C_{31}H_{25}N_2O_2S$ 489.1631; Found 489.1652.

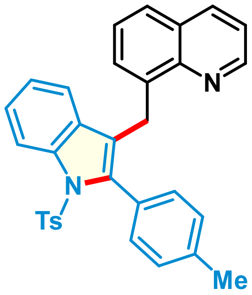

4, 81%

8-((2-(p-tolyl)-1-tosyl-1*H*-indol-3-yl)methyl)quinoline (4)

40.7 mg, 81% yield, white solid.

***R_f*:** 0.50 (hexane/ethyl acetate, 9:1 v/v).

^{1}H NMR (400 MHz, CDCl$_3$) δ 8.92 (d, *J* = 2.7 Hz, 1H), 8.38 (d, *J* = 8.4 Hz, 1H), 8.15 (d, *J* = 8.2 Hz, 1H), 7.63 (d, *J* = 8.1 Hz, 1H), 7.43 (dd, *J* = 8.2, 4.2 Hz, 1H), 7.36 – 7.34 (m, 3H), 7.29 – 7.22 (m, 2H), 7.13 (p, *J* = 6.1 Hz, 7H), 6.63 (d, *J* = 7.0 Hz, 1H), 4.54 (s, 2H), 2.39 (s, 3H), 2.37 (s, 3H).

^{13}C NMR (100 MHz, CDCl$_3$) δ 149.48, 146.69, 144.46, 139.14, 138.49, 138.37, 138.11, 136.46, 134.89, 132.00, 130.87, 129.27, 128.48, 128.40, 128.28, 127.86, 127.13, 126.23, 125.02, 124.33, 122.86, 121.21, 119.95, 117.01, 25.68, 21.75, 21.57.

HRMS (ESI-TOF) m/z: $[M+Na]^+$calcd for $C_{32}H_{26}N_2NaO_2S$ 525.1607; Found 525.1599.

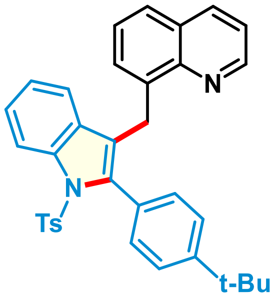

5, 76%

8-((2-(4-(tert-butyl)phenyl)-1-tosyl-1*H*-indol-3-yl)methyl)quinoline (5)

41.5 mg, 76% yield, white solid.

***R$_f$*:** 0.50 (hexane/ethyl acetate, 9:1 v/v).

^{1}H NMR (400 MHz, CDCl$_3$) δ 8.95 (dd, *J* = 4.1, 1.6 Hz, 1H), 8.40 (d, *J* = 8.4 Hz, 1H), 8.18 (dd, *J* = 8.3, 1.5 Hz, 1H), 7.66 (d, *J* = 8.1 Hz, 1H), 7.46 (dd, *J* = 8.3, 4.2 Hz, 1H), 7.39 – 7.32 (m, 5H), 7.31 – 7.28 (m, 2H), 7.20 – 7.09 (m, 5H), 6.67 (d, *J* = 7.0 Hz, 1H), 4.57 (s, 2H), 2.41 (s, 3H), 1.35 (s, 9H).

^{13}C NMR (125 MHz, CDCl$_3$) δ 151.49, 149.49, 146.74, 144.39, 139.24, 138.44, 138.18, 136.49, 134.94, 131.94, 130.68, 129.24, 128.30, 127.93, 127.22, 126.26, 126.22, 124.98, 124.53, 124.26, 122.79, 121.22, 119.96, 117.02, 34.82, 31.47, 25.73, 21.76.

HRMS (ESI-TOF) m/z: $[M+H]^+$calcd for $C_{35}H_{33}N_2O_2S$ 545.2257; Found 545.2268.

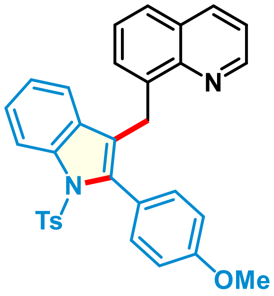

6, 82%

8-((2-(4-methoxyphenyl)-1-tosyl-1*H*-indol-3-yl)methyl)quinoline (6)

42.5 mg, 82% yield, white solid.

***R$_f$*:** 0.50 (hexane/ethyl acetate, 9:1 v/v).

^{1}H NMR (400 MHz, CDCl$_3$) δ 8.93 (dd, *J* = 4.1, 1.7 Hz, 1H), 8.38 (d, *J* = 8.4 Hz, 1H), 8.15 (dd, *J* = 8.3, 1.6 Hz, 1H), 7.63 (d, *J* = 8.0 Hz, 1H), 7.43 (dd, *J* = 8.2, 4.2 Hz, 1H), 7.38 – 7.30 (m, 3H), 7.28 (d, *J* = 8.7 Hz, 2H), 7.16 – 7.10 (m, 5H), 6.86 (d, *J* = 8.7 Hz, 2H), 6.63 (d, *J* = 7.0 Hz, 1H), 4.53 (s, 2H), 3.81 (s, 3H), 2.38 (s, 3H).

^{13}C NMR (100 MHz, CDCl$_3$) δ 159.90, 149.47, 146.66, 144.46, 138.87, 138.35, 138.06, 136.49, 134.98, 132.33, 131.95, 129.28, 128.29, 127.83, 127.10, 126.24, 124.95, 124.31, 123.60, 122.52, 121.22, 119.87, 117.00, 113.12, 55.33, 25.69, 21.75.

HRMS (ESI-TOF) m/z:[M+H]$^+$ calcd for $C_{32}H_{27}N_2O_3S$ 519.1737; Found 519.1759.

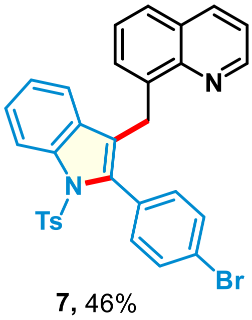

7, 46%

8-((2-(4-bromophenyl)-1-tosyl-1*H*-indol-3-yl)methyl)quinoline (7)

26.1 mg, 46% yield, white solid.

***R_f*:** 0.6 (hexane/ethyl acetate, 9.1 v/v).

^{1}H NMR (500 MHz, CDCl$_3$) δ ^{1}H NMR (400 MHz, CDCl$_3$) δ 8.93 (d, *J* = 2.7 Hz, 1H), 8.38 (d, *J* = 8.4 Hz, 1H), 8.17 (d, *J* = 8.0 Hz, 1H), 7.69 – 7.60 (m, 2H), 7.49 – 7.41 (m, 3H), 7.38 (ddd, *J* = 8.4, 6.2, 2.3 Hz, 1H), 7.32 (d, *J* = 8.2 Hz, 2H), 7.21 (d, *J* = 8.4 Hz, 2H), 7.16 (d, *J* = 6.5 Hz, 2H), 7.12 (d, *J* = 8.0 Hz, 2H), 6.59 (d, *J* = 6.9 Hz, 1H), 4.53 (s, 2H), 2.39 (s, 3H).

^{13}C NMR (125 MHz, CDCl$_3$) δ 149.51, 144.75, 142.11, 139.26, 138.21, 137.57, 134.75, 133.10, 132.49, 131.82, 130.89, 130.40, 129.79, 129.41, 128.35, 127.61, 127.04, 126.28, 125.49, 124.56, 123.72, 123.09, 121.33, 120.11, 117.05, 25.72, 21.77.

HRMS (ESI-TOF) m/z: [M+H]$^+$calcd for $C_{31}H_{24}BrN_2O_2S$ 567.0736; Found 567.0756.

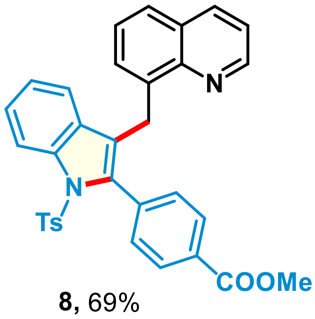

8, 69%

methyl 4-(3-(quinolin-8-ylmethyl)-1-tosyl-1H-indol-2-yl)benzoate (8)

37.7 mg, 69% yield, white solid.

***R_f*:** 0.40 (hexane/ethyl acetate, 9:1 v/v).

^{1}H NMR (400 MHz, $CDCl_3$) δ 8.94 (d, *J* = 2.8 Hz, 1H), 8.41 (d, *J* = 8.4 Hz, 1H), 8.19 (d, *J* = 8.1 Hz, 1H), 8.03 (d, *J* = 8.3 Hz, 2H), 7.67 (d, *J* = 8.2 Hz, 1H), 7.48 – 7.46 (m, 3H), 7.42 (ddd, *J* = 8.4, 6.1, 2.4 Hz, 1H), 7.34 (d, *J* = 8.2 Hz, 2H), 7.19 – 7.17 (m, 3H), 7.14 (d, *J* = 8.1 Hz, 2H), 6.61 (d, *J* = 7.0 Hz, 1H), 4.57 (s, 2H), 3.93 (s, 3H), 2.41 (s, 3H).

^{13}C NMR (100 MHz, $CDCl_3$) δ 166.95, 149.47, 144.79, 138.39, 137.79, 137.71, 136.18, 134.50, 131.91, 130.90, 129.99, 129.41, 128.87, 128.34, 127.79, 127.04, 126.50, 126.24, 125.68, 124.66, 124.41, 121.33, 120.22, 117.13, 52.30, 25.73, 21.77.

HRMS (ESI-TOF) m/z: $[M+H]^+$calcd for $C_{33}H_{27}N_2O_4S$ 547.1686; Found 547.1703.

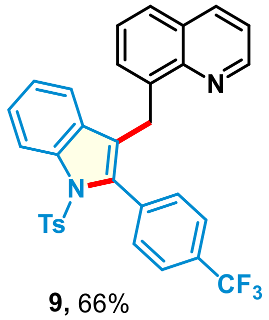

9, 66%

8-((1-tosyl-2-(4-(trifluoromethyl)phenyl)-1H-indol-3-yl)methyl)quinoline (9)

36.7 mg, 66% yield, white solid.

***R_f*:** 0.40 (hexane/ethyl acetate, 9:1 v/v).

^{1}H NMR (400 MHz, $CDCl_3$) δ ^{1}H NMR (400 MHz, $CDCl_3$) δ 8.91 (d, *J* = 3.9 Hz, 1H), 8.39 (d, *J* = 8.4 Hz, 1H), 8.16 (d, *J* = 8.2 Hz, 1H), 7.65 (d, *J* = 8.2 Hz, 1H), 7.57 (d, *J* = 8.1 Hz, 2H), 7.47 (d, *J* = 8.6 Hz, 2H), 7.45 – 7.36 (m, 2H), 7.31 (d, *J* = 8.0 Hz, 2H), 7.20 – 7.12 (m, 5H), 6.59 (d, *J* = 7.1 Hz, 1H), 4.53 (s, 2H), 2.40 (s, 3H).

^{13}C NMR (101 MHz, $CDCl_3$) δ 149.62, 146.58, 144.84, 138.36, 137.90, 137.18, 136.51, 135.23, 134.58, 131.84, 131.18, 130.23 (q, *J* = 33.0 Hz), 129.44, 128.35, 127.75, 127.04, 126.55, 126.17, 125.77, 124.69, 124.62 (q, *J* = 4.0 Hz), 121.39, 120.27, 117.12, 25.74, 21.78.

^{19}F NMR (376 MHz, $CDCl_3$) δ -62.57.

HRMS (ESI-TOF) m/z: $[M+H]^+$calcd for $C_{32}H_{24}F_3N_2O_2S$ 557.1506; Found 557.1532.

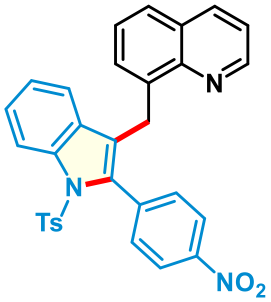

10, 52%

8-((2-(4-nitrophenyl)-1-tosyl-1*H*-indol-3-yl)methyl)quinoline (10)

27.8 mg, 52% yield, yellow solid.

***R_f*:** 0.30 (hexane/ethyl acetate, 9:1 v/v).

^{1}H NMR (500 MHz, CDCl$_3$) δ ^{1}H NMR (500 MHz, CDCl$_3$) δ 8.92 (dd, *J* = 4.0, 1.5 Hz, 1H), 8.41 (d, *J* = 8.4 Hz, 1H), 8.20 – 8.17 (m, 3H), 7.68 (d, *J* = 8.3 Hz, 1H), 7.56 (d, *J* = 8.8 Hz, 2H), 7.49 – 7.42 (m, 2H), 7.32 (d, *J* = 8.3 Hz, 2H), 7.23 (d, *J* = 4.0 Hz, 2H), 7.20 – 7.11 (m, 3H), 6.60 (d, *J* = 7.0 Hz, 1H), 4.58 (s, 2H), 2.42 (s, 3H).

^{13}C NMR (125 MHz, CDCl$_3$) δ 149.68, 147.64, 146.52, 145.05, 138.61, 138.36, 137.67, 136.52, 136.28, 134.28, 131.96, 131.60, 129.52, 128.39, 127.71, 127.38, 126.98, 126.74, 126.22, 126.14, 124.97, 122.86, 121.49, 120.44, 117.26, 25.85, 21.79.

HRMS (ESI-TOF) m/z: $[M+H]^+$calcd for $C_{31}H_{24}N_3O_4S$ 534.1482; Found 534.1498.

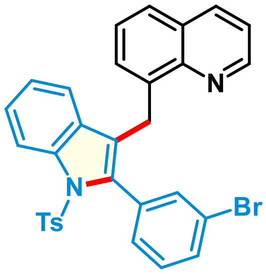

11, 40%

8-((2-(3-bromophenyl)-1-tosyl-1*H*-indol-3-yl)methyl)quinoline (11)

22.7 mg, 40% yield, white solid.

***R_f*:** 0.50 (hexane/ethyl acetate, 9:1 v/v).

^{1}H NMR (400 MHz, CDCl$_3$) δ 8.93 (d, *J* = 2.8 Hz, 1H), 8.35 (d, *J* = 8.4 Hz, 1H), 8.16 (d, *J* = 7.8 Hz, 1H), 7.64 (dd, *J* = 7.6, 3.6 Hz, 2H), 7.53 (d, *J* = 8.3 Hz, 2H), 7.43 (dd, *J* = 8.1, 4.1 Hz, 1H), 7.41 – 7.35 (m, 1H), 7.33 – 7.26 (m, 4H), 7.23 (d, *J* = 7.7 Hz, 1H), 7.19 (d, *J* = 8.1 Hz, 2H), 7.14 (t, *J* = 7.8 Hz, 2H), 4.55 (q, *J* = 17.2 Hz, 2H), 2.39 (s, 3H).

^{13}C NMR (101 MHz, CDCl$_3$) δ 149.18, 144.79, 136.98, 136.38, 136.00, 133.23, 132.83, 132.73, 130.48, 130.42, 129.79, 129.62, 129.14, 128.34, 127.60, 127.21, 126.98, 126.55, 126.32, 125.22, 123.62, 122.38, 121.06, 120.57, 115.51, 25.54, 21.75

HRMS (ESI-TOF) m/z: [M+H]$^+$calcd for $C_{31}H_{24}BrN_2O_2S$ 567.0736; Found 567.0752.

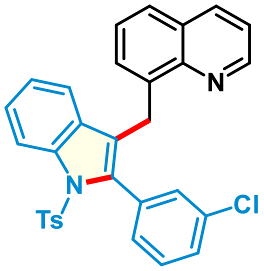

12, 71%

8-((2-(3-chlorophenyl)-1-tosyl-1*H*-indol-3-yl)methyl)quinoline (12)

37.1 mg, 71% yield, white solid.

***R_f*:** 0.50 (hexane/ethyl acetate, 9:1 v/v).

^{1}H NMR (400 MHz, CDCl$_3$) δ 8.94 (d, J = 3.8 Hz, 1H), 8.40 (d, J = 8.4 Hz, 1H), 8.17 (d, J = 8.2 Hz, 1H), 7.66 (d, J = 8.1 Hz, 1H), 7.45 (dd, J = 8.2, 4.1 Hz, 1H), 7.42 – 7.39 (m, 1H), 7.36 (d, J = 8.0 Hz, 3H), 7.29 – 7.28 (m, 3H), 7.26 – 7.10 (m, 5H), 6.73 (d, J = 7.1 Hz, 1H), 4.57 (s, 2H), 2.42 (s, 3H).

^{13}C NMR (100 MHz, CDCl$_3$) δ 149.54, 146.58, 144.80, 138.13, 137.98, 137.04, 136.48, 134.93, 133.48, 133.20, 131.53, 131.03, 129.44, 129.33, 128.86, 128.74, 128.34, 127.96, 127.06, 126.46, 126.23, 125.49, 124.40, 123.81, 121.29, 120.25, 116.82, 25.57, 21.76.

HRMS (ESI-TOF) m/z: [M+Na]$^+$calcd for $C_{31}H_{23}ClN_2NaO_2S$ 545.1061; Found 545.1054.

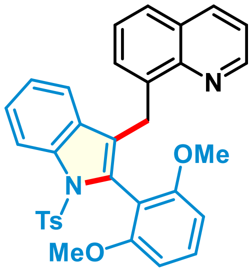

13, 68%

8-((2-(2,6-dimethoxyphenyl)-1-tosyl-1*H*-indol-3-yl)methyl)quinoline (13)

37.3 mg, 68% yield, red solid.

***R_f*:** 0.50 (hexane/ethyl acetate, 8:2 v/v).

^{1}H NMR (400 MHz, CDCl$_3$) δ 8.94 (s, 1H), 8.24 (d, *J* = 8.4 Hz, 1H), 8.13 (d, *J* = 8.1 Hz, 1H), 7.59 (d, *J* = 7.3 Hz, 1H), 7.55 (d, *J* = 8.3 Hz, 2H), 7.40 (dd, *J* = 8.1, 3.9 Hz, 1H), 7.29 (t, *J* = 5.8 Hz, 1H), 7.25 (dd, *J* = 7.2, 5.9 Hz, 3H), 7.17 (d, *J* = 7.7 Hz, 1H), 7.13 (d, *J* = 8.2 Hz, 2H), 7.06 (t, *J* = 7.5 Hz, 1H), 6.49 (d, *J* = 8.4 Hz, 2H), 4.48 (s, 2H), 3.55 (s, 6H), 2.35 (s, 3H).

^{13}C NMR (100 MHz, CDCl$_3$) δ 159.69, 149.10, 143.89, 138.22, 137.30, 136.89, 136.60, 131.23, 131.01, 130.68, 129.29, 129.15, 127.27, 126.46, 125.80, 124.05, 122.78, 121.51, 120.84, 119.95, 114.94, 108.95, 103.33, 55.41, 25.33, 21.66.

HRMS (ESI-TOF) m/z: [M+Na]$^+$calcd for $C_{33}H_{28}N_2NaO_4S$ 571.1662; Found 571.1661.

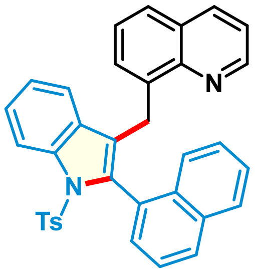

14, 80%

8-((2-(naphthalen-1-yl)-1-tosyl-1*H*-indol-3-yl)methyl)quinoline (14)

43.1 mg, 80% yield, white solid.

***R$_f$*:** 0.50 (hexane/ethyl acetate, 9:1 v/v).

^{1}H NMR (400 MHz, CDCl$_3$) δ 8.82 (dd, *J* = 4.0, 1.5 Hz, 1H), 8.47 (d, *J* = 8.4 Hz, 1H), 8.06 (dd, *J* = 8.2, 1.4 Hz, 1H), 7.88 (d, *J* = 7.9 Hz, 1H), 7.82 (d, *J* = 8.1 Hz, 1H), 7.57 (dd, *J* = 11.5, 8.6 Hz, 2H), 7.44 – 7,39 (m, 4H), 7.36 – 7.33 (m, 1H), 7.32 – 7.26 (m, 4H), 7.19 (t, *J* = 7.2 Hz, 2H), 7.00 (d, *J* = 8.0 Hz, 3H), 4.63 (d, *J* = 17.1 Hz, 1H), 4.32 (d, *J* = 17.1 Hz, 1H), 2.31 (s, 3H).

^{13}C NMR (100 MHz, CDCl$_3$) δ 149.26, 146.49, 144.52, 137.93, 137.61, 136.40, 136.10, 135.69, 133.95, 133.23, 130.79, 129.86, 129.68, 129.30, 128.84, 128.44, 128.19, 128.09, 127.12, 126.34, 126.30, 126.27, 126.14, 125.79, 125.02, 124.58, 123.69, 123.21, 121.01, 120.39, 115.80, 25.52, 21.63.

HRMS (ESI-TOF) m/z: [M+H]$^+$calcd for $C_{35}H_{27}N_2O_2S$ 539.1788; Found 539.1787.

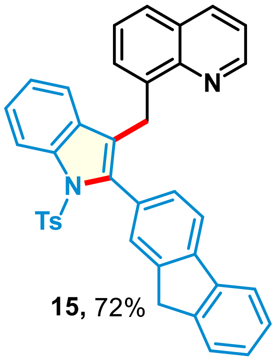

15, 72%

8-((2-(9H-fluoren-2-yl)-1-tosyl-1*H*-indol-3-yl)methyl)quinoline (15)

41.5 mg, 72% yield, white solid.

***R_f*:** 0.50 (hexane/ethyl acetate, 9:1 v/v).

^{1}H NMR (400 MHz, CDCl$_3$) δ 8.91 (d, *J* = 2.7 Hz, 1H), 8.41 (d, *J* = 8.4 Hz, 1H), 8.15 (d, *J* = 7.8 Hz, 1H), 7.76 (d, *J* = 7.4 Hz, 1H), 7.71 (d, *J* = 7.8 Hz, 1H), 7.64 (d, *J* = 8.1 Hz, 1H), 7.53 (s, 2H), 7.42 (dd, *J* = 8.3, 4.3 Hz, 1H), 7.39 – 7.25 (m, 6H), 7.20 – 7.15 (m, 3H), 7.11 (d, *J* = 8.1 Hz, 2H), 6.73 (d, *J* = 7.0 Hz, 1H), 4.60 (s, 2H), 3.87 (s, 2H), 2.39 (s, 3H).

^{13}C NMR (125 MHz, CDCl$_3$) δ 149.36, 144.52, 143.75, 142.40, 142.14, 141.43, 139.33, 138.22, 138.16, 135.02, 131.90, 129.65, 129.28, 128.31, 128.11, 127.98, 127.16, 127.09, 126.91, 126.35, 126.28, 125.15, 125.10, 124.33, 122.95, 121.19, 120.23, 120.01, 118.99, 116.96, 37.03, 25.71, 21.75.

HRMS (ESI-TOF) m/z: $[M+H]^+$calcd for $C_{38}H_{29}N_2O_2S$ 577.1944; Found 577.1965.

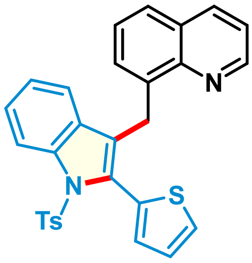

16, 73%

8-((2-(thiophen-2-yl)-1-tosyl-1*H*-indol-3-yl)methyl)quinoline (16)

36.1 mg, 73% yield, white solid.

***R_f*:** 0.40 (hexane/ethyl acetate, 9:1 v/v).

^{1}H NMR (400 MHz, CDCl$_3$) δ 8.94 (dd, *J* = 4.1, 1.4 Hz, 1H), 8.37 (d, *J* = 8.4 Hz, 1H), 8.15 (dd, *J* = 8.2, 1.4 Hz, 1H), 7.63 (d, *J* = 8.1 Hz, 1H), 7.47 – 7.40 (m, 4H), 7.39 – 7.34 (m, 1H), 7.19 - 7.13 (m, 5H), 7.05 (d, *J* = 3.3 Hz, 1H), 7.03 – 6.98 (m, 1H), 6.70 (d, *J* = 7.1 Hz, 1H), 4.62 (s, 2H), 2.39 (s, 3H).

^{13}C NMR (100 MHz, CDCl$_3$) δ 149.50, 146.61, 144.62, 138.20, 138.10, 136.47, 135.03, 131.63, 131.41, 131.24, 130.69, 129.40, 128.31, 128.25, 127.99, 127.14, 126.48, 126.32, 126.23, 125.56, 125.23, 124.29, 121.22, 120.19, 116.75, 25.81, 21.76.

HRMS (ESI-TOF) m/z: [M+Na]$^+$calcd for $C_{29}H_2N_2NaO_2S_2$ 517.1009; Found 517.1015

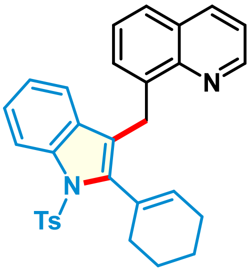

17, 68%

4-(1-((4-(tert-butyl)phenyl)sulfonyl)-2-phenyl-1*H*-indol-3-yl)-*N*-(quinolin-8-yl)butanamide (17)

33.5 mg, 68% yield, yellow sticky solid.

***R$_f$*:** 0.10 (hexane/ethyl acetate, 9:1 v/v).

^{1}H NMR (400 MHz, CDCl$_3$) δ 9.05 – 8.96 (m, 1H), 8.26 (d, *J* = 8.4 Hz, 1H), 8.20 (d, *J* = 8.0 Hz, 1H), 7.64 (d, *J* = 8.4 Hz, 1H), 7.56 (d, *J* = 8.3 Hz, 2H), 7.48 (dd, *J* = 8.2, 4.2 Hz, 1H), 7.28 (dd, *J* = 7.6, 6.3 Hz, 1H), 7.20 – 7.12 (m, 3H), 7.10 – 7.02 (m, 2H), 6.67 (d, *J* = 7.0 Hz, 1H), 5.52 (s, 1H), 4.63 (d, *J* = 40.6 Hz, 2H), 2.87 (d, *J* = 35.6 Hz, 1H), 2.37 (s, 3H), 2.29 – 1.99 (m, 3H), 1.65 – 1.63 (m, 4H).

^{13}C NMR (125 MHz, CDCl$_3$) δ 149.29, 144.42, 141.71, 138.51, 137.60, 135.12, 132.01, 131.11, 130.21, 129.69, 129.28, 128.34, 128.21, 127.41, 127.04, 126.46, 126.16, 124.70, 124.00, 121.19, 121.05, 119.95, 116.36, 30.81, 25.70, 25.68, 22.79, 22.01, 21.73.

HRMS (ESI-TOF) m/z: [M+Na]$^+$calcd for $C_{31}H_{28}N_2NaO_2S$ 515.1764; Found 515.1756.

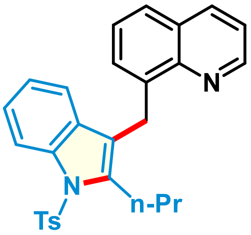

18, 68%

8-((2-propyl-1-tosyl-1H-indol-3-yl)methyl)quinoline (18)

30.9 mg, 68% yield, yellow sticky solid.

R**$_f$:** 0.60 (hexane/ethyl acetate, 9:1 v/v).

^{1}H NMR (500 MHz, $CDCl_3$) δ 9.01 (dd, *J* = 4.1, 1.6 Hz, 1H), 8.23 (d, *J* = 8.4 Hz, 1H), 8.17 (dd, *J* = 8.3, 1.5 Hz, 1H), 7.64 (d, *J* = 8.1 Hz, 1H), 7.58 (d, *J* = 8.3 Hz, 2H), 7.46 (dd, *J* = 8.2, 4.2 Hz, 1H), 7.27 - 7.23 (m, 1H), 7.22 – 7.14 (m, 4H), 7.10 (t, *J* = 7.4 Hz, 1H), 6.84 (d, *J* = 7.1 Hz, 1H), 4.69 (s, 2H), 3.08 – 2.93 (m, 2H), 2.36 (s, 3H), 1.78 – 1.64 (m, 2H), 0.90 (t, *J* = 7.4 Hz, 3H).

^{13}C NMR (125 MHz, $CDCl_3$) δ 149.53, 146.83, 144.46, 139.77, 138.06, 137.42, 136.54, 136.13, 131.65, 129.71, 128.37, 128.02, 126.41, 126.31, 124.20, 123.74, 121.21, 120.30, 119.27, 115.78, 28.80, 25.07, 24.29, 21.68, 14.16.

HRMS (ESI-TOF) m/z: $[M+H]^+$calcd for $C_{28}H_{27}N_2O_2S$ 455.1788; Found 455.1778.

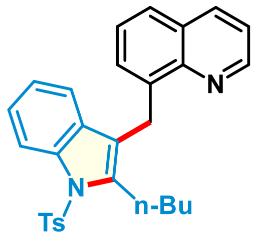

19, 64%

8-((2-butyl-1-tosyl-1*H*-indol-3-yl)methyl)quinoline (19)

30.0 mg, 64% yield, yellow sticky solid.

R**$_f$:** 0.60 (hexane/ethyl acetate, 9:1 v/v).

^{1}H NMR (500 MHz, $CDCl_3$) δ 9.01 (dd, *J* = 4.1, 1.6 Hz, 1H), 8.23 (d, *J* = 8.4 Hz, 1H), 8.18 (dd, *J* = 8.2, 1.5 Hz, 1H), 7.64 (d, *J* = 8.0 Hz, 1H), 7.58 (d, *J* = 8.3 Hz, 2H), 7.47 (dd, *J* = 8.2, 4.2 Hz, 1H), 7.27 - 7.20 (m, 2H), 7.19 – 7.14 (m, 3H), 7.10 (t, *J* = 7.4 Hz, 1H), 6.84 (d, *J* = 7.1 Hz, 1H), 4.68 (s, 2H), 3.21 – 2.85 (m, 2H), 2.36 (s, 3H), 1.63 (dt, *J* = 15.3, 7.6 Hz, 2H), 1.31 (dd, *J* = 14.0, 6.4 Hz, 2H), 0.82 (t, *J* = 7.3 Hz, 3H).

^{13}C NMR (125 MHz, $CDCl_3$) δ 149.47, 144.48, 139.97, 137.98, 137.39, 136.67, 136.08, 131.64, 129.72, 128.37, 128.11, 126.41, 126.36, 126.31, 124.17, 123.74, 121.21, 120.08, 119.21, 115.75, 33.08, 26.69, 25.00, 22.84, 21.69, 13.90.

HRMS (ESI-TOF) m/z: $[M+H]^+$calcd for $C_{29}H_{29}N_2O_2S$ 469.1944; Found 469.1958.

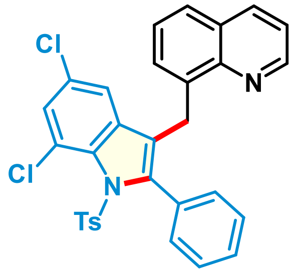

20, 41%

8-((5,7-dichloro-2-phenyl-1-tosyl-1*H*-indol-3-yl)methyl)quinoline (20)

22.9 mg, 41% yield, white solid.

***Rf*:** 0.50 (hexane/ethyl acetate, 9:1 v/v).

^{1}H NMR (400 MHz, CDCl$_3$) δ 8.94 (d, *J* = 3.5 Hz, 1H), 8.21 (d, *J* = 8.1 Hz, 1H), 7.71 (d, *J* = 8.1 Hz, 1H), 7.48 (dd, *J* = 8.2, 4.1 Hz, 1H), 7.40 (s, 1H), 7.34 (dd, *J* = 7.8, 3.9 Hz, 1H), 7.30 – 7.26 (m, 4H), 7.21 – 7.17 (m, 3H), 7.13 (d, *J* = 8.1 Hz, 2H), 6.95 (s, 1H), 6.45 (d, *J* = 6.7 Hz, 1H), 4.47 (s, 2H), 2.45 (s, 3H).

^{13}C NMR (100 MHz, CDCl$_3$) δ 149.53, 144.91, 144.79, 138.37, 137.41, 137.12, 136.33, 134.34, 131.57, 130.89, 130.37, 129.29, 129.13, 128.46, 127.95, 127.83, 127.62, 126.75, 126.29, 126.02, 123.94, 121.51, 118.50, 26.04, 21.86.

HRMS (ESI-TOF) m/z: [M+H]$^+$calcd for $C_{31}H_{23}Cl_2N_2O_2S$ 557.0852; Found 557.0856.

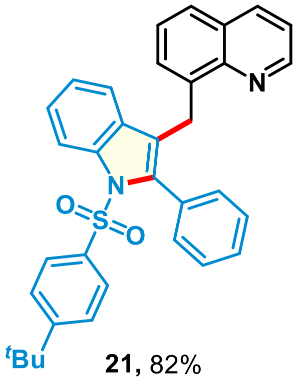

21, 82%

8-((1-((4-(tert-butyl)phenyl)sulfonyl)-2-phenyl-1*H*-indol-3-yl)methyl)quinoline (21)

43.5 mg, 82% yield, white solid.

***Rf*:** 0.50 (hexane/ethyl acetate, 9:1 v/v).

^{1}H NMR (400 MHz, CDCl$_3$) δ 8.92 (dd, *J* = 4.1, 1.5 Hz, 1H), 8.40 (d, *J* = 8.4 Hz, 1H), 8.15 (dd, *J* = 8.2, 1.4 Hz, 1H), 7.63 (d, *J* = 8.1 Hz, 1H), 7.45 – 7.37 (m, 4H), 7.37 – 7.30 (m, 7H), 7.18 (t, *J* = 7.7 Hz, 1H), 7.14 (d, *J* = 4.0 Hz, 2H), 6.80 (d, *J* = 7.1 Hz, 1H), 4.56 (s, 2H), 1.30 (s, 9H).

^{13}C NMR (100 MHz, CDCl$_3$) δ 157.54, 149.44, 138.80, 138.27, 137.98, 136.54, 135.24, 131.51, 131.35, 131.19, 128.65, 128.30, 127.92, 127.59, 126.97, 126.32, 126.27, 125.72, 125.11, 124.15, 122.59, 121.22, 120.10, 116.68, 35.34, 31.18, 25.66.

HRMS (ESI-TOF) m/z: [M+Na]$^+$calcd for $C_{34}H_{30}N_2NaO_2S$ 553.1920; Found 553. 1913.

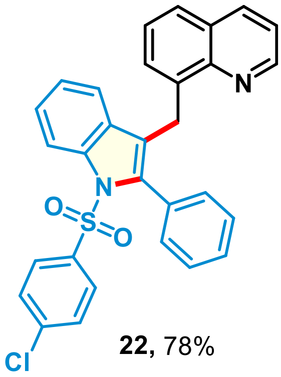

22, 78%

8-((1-((4-chlorophenyl)sulfonyl)-2-phenyl-1*H*-indol-3-yl)methyl)quinoline (22)

39.7 mg, 78% yield, white solid.

***R$_f$*:** 0.50 (hexane/ethyl acetate, 9:1 v/v).

^{1}H NMR (400 MHz, CDCl$_3$) δ 8.95 (dd, *J* = 4.1, 1.6 Hz, 1H), 8.40 (d, *J* = 8.4 Hz, 1H), 8.19 (dd, *J* = 8.3, 1.5 Hz, 1H), 7.73 – 7.64 (m, 1H), 7.46 (dd, *J* = 8.3, 4.2 Hz, 1H), 7.44 – 7.39 (m, 3H), 7.38 - 7.36 (m, 5H), 7.33 - 7.31 (m, 2H), 7.25 (t, *J* = 7.7 Hz, 1H), 7.21 - 7.15 (m, 2H), 6.58 (d, *J* = 6.7 Hz, 1H), 4.58 (s, 2H).

^{13}C NMR (100 MHz, CDCl$_3$) 149.48, 146.51, 140.26, 138.75, 138.10, 138.05, 136.59, 135.86, 132.13, 131.67, 131.14, 130.93, 129.01, 128.83, 128.52, 128.35, 127.75, 126.46, 126.32, 125.47, 124.82, 123.91, 121.30, 120.25, 117.11, 25.71.

HRMS (ESI-TOF) m/z: [M+Na]$^+$calcd for $C_{30}H_{21}ClN_2NaO_2S$ 531.0904; Found 531.0898.

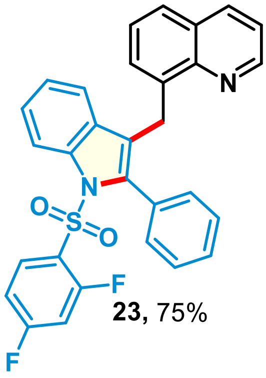

23, 75%

8-((1-((2,4-difluorophenyl)sulfonyl)-2-phenyl-1*H*-indol-3-yl)methyl)quinoline (23)

38.3 mg, 75% yield, white solid.

***R_f*:** 0.40 (hexane/ethyl acetate, 9:1 v/v).

^{1}H NMR (500 MHz, $CDCl_3$) δ 8.95 (d, *J* = 2.7 Hz, 1H), 8.26 (d, *J* = 8.4 Hz, 1H), 8.18 (d, *J* = 8.2 Hz, 1H), 7.68 (d, *J* = 8.1 Hz, 1H), 7.45 (dd, *J* = 8.4, 4.3 Hz, 1H), 7.40 (d, 6.3 Hz, 1H), 7.37 – 7.30 (m, 3H), 7.27 (dd, *J* = 13.3, 6.0 Hz, 5H), 7.18 (t, *J* = 7.5 Hz, 1H), 7.07 (d, *J* = 7.1 Hz, 1H), 6.82 (dt, *J* = 16.4, 5.1 Hz, 2H), 4.61 (s, 2H).

^{13}C NMR (126 MHz, $CDCl_3$) δ 166.69 (dd, *J* = 132.5, 11.25 Hz), 160.29 (dd, *J* = 260.63, 12.50 Hz), 149.43, 146.55, 138.09, 137.54, 136.58, 132.83, 132.74, 131.38, 130.68, 130.65, 128.77, 128.35, 128.06, 127.65, 126.42, 126.38, 125.12, 124.09, 122.19, 121.22, 120.28, 116.16, 111.62 (d, *J* = 26.25 Hz), 105.59 (t, *J* = 25.61 Hz), 25.53.

^{19}F NMR (376 MHz, $CDCl_3$) δ -98.56, -102.42.

HRMS (ESI-TOF) m/z: $[M+Na]^+$calcd for $C_{30}H_{20}F_2N_2NaO_2S$ 533.1106; Found 533.1099.

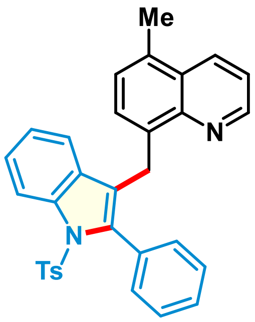

24, 69%

5-methyl-8-((2-phenyl-1-tosyl-1*H*-indol-3-yl)methyl)quinoline (24)

34.7 mg, 69% yield, white solid.

***R_f*:** 0.50 (hexane/ethyl acetate, 9:1 v/v).

^{1}H NMR (500 MHz, $CDCl_3$) δ 8.95 (dd, *J* = 4.0, 1.4 Hz, 1H), 8.40 (d, *J* = 8.4 Hz, 1H), 8.35 (dd, *J* = 8.4, 1.2 Hz, 1H), 7.48 (dd, *J* = 8.5, 4.1 Hz, 1H), 7.41 – 7.33 (m, 8H), 7.15 (dd, *J* = 13.9, 7.3 Hz, 4H), 7.00 (d, *J* = 7.3 Hz, 1H), 6.53 (d, *J* = 7.2 Hz, 1H), 4.53 (s, 2H), 2.64 (s, 3H), 2.42 (s, 3H).

^{13}C NMR (125 MHz, $CDCl_3$) δ 148.90, 146.78, 144.50, 138.90, 138.17, 136.08, 134.89, 132.97, 132.76, 131.97, 131.46, 131.03, 129.75, 129.31, 128.60, 127.68, 127.60, 127.12, 126.74, 125.13, 124.35, 123.28, 120.80, 120.08, 117.00, 25.67, 21.76, 18.67.

HRMS (ESI-TOF) m/z: $[M+H]^+$calcd for $C_{32}H_{27}N_2O_2S$ 503.1789; Found 503.1807.

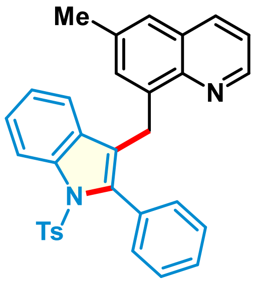

25, 60 %

6-methyl-8-((2-phenyl-1-tosyl-1H-indol-3-yl)methyl)quinoline (25)

30.2 mg, 60% yield, white solid.

R**$_f$:**0.50 (hexane/ethyl acetate, 9:1 v/v).

^{1}H NMR (400 MHz, CDCl$_3$) δ 8.87 (dd, *J* = 4.1, 1.5 Hz, 1H), 8.41 (d, *J* = 8.4 Hz, 1H), 8.08 (dd, *J* = 8.2, 1.4 Hz, 1H), 7.45 – 7.34 (m, 10H), 7.20 – 7.11 (m, 4H), 6.63 (s, 1H), 4.55 (s, 2H), 2.37 (s, 3H), 2.24 (s, 3H).

^{13}C NMR (100 MHz, CDCl$_3$) δ 148.52, 144.50, 138.78, 137.96, 137.83, 136.02, 135.86, 135.13, 131.70, 131.44, 131.09, 130.23, 129.37, 128.67, 128.45, 127.61, 127.08, 125.12, 125.09, 124.25, 122.94, 121.24, 120.17, 116.72, 25.57, 21.83, 21.72.

HRMS (ESI-TOF) m/z: [M+H]$^+$calcd for $C_{32}H_{27}N_2O_2S$ 503.1789; Found 503.1791.

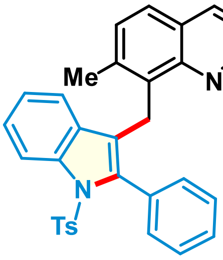

26, 56%

7-methyl-8-((2-phenyl-1-tosyl-1*H*-indol-3-yl)methyl)quinoline (26)

28.2 mg, 56% yield, yellow sticky liquid.

R**$_f$:** 0.50 (hexane/ethyl acetate, 9:1 v/v).

^{1}H NMR (500 MHz, CDCl$_3$) δ 8.92 (d, *J* = 3.4 Hz, 1H), 8.31 (d, *J* = 8.4 Hz, 1H), 8.12 (d, *J* = 8.0 Hz, 1H), 7.56 (d, *J* = 8.3 Hz, 1H), 7.38 (d, *J* = 3.7 Hz, 3H), 7.31 (dd, *J* = 16.4, 8.2 Hz, 5H), 7.23 (t, *J* = 7.8 Hz, 1H), 7.19 (d, *J* = 8.3 Hz, 1H), 7.08 (d, *J* = 8.1 Hz, 2H), 6.89 (t, *J* = 7.5 Hz, 1H), 6.65 (d, *J* = 6.7 Hz, 1H), 4.74 (s, 2H), 2.35 (s, 3H), 1.99 (s, 3H).

^{13}C NMR (125 MHz, CDCl$_3$) δ 149.25, 144.54, 137.45, 136.91, 135.26, 134.96, 131.79, 131.70, 131.03, 130.20, 129.36, 128.41, 127.18, 126.92, 126.65, 126.04, 124.60, 123.72, 122.45, 120.16, 120.12, 116.02, 23.29, 21.67, 20.60.

HRMS (ESI-TOF) m/z: [M+Na]$^+$calcd for $C_{32}H_{26}N_2NaO_2S$ 525.1607; Found 525.1601.

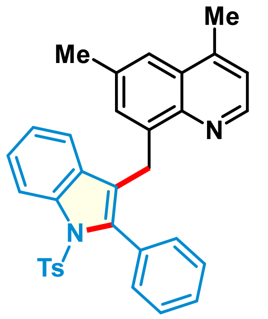

27, 74%

4,6-dimethyl-8-((2-phenyl-1-tosyl-1*H*-indol-3-yl)methyl)quinoline (27)

38.2 mg, 74% yield, white solid.

***R_f*: ** 0.60 (hexane/ethyl acetate, 9:1 v/v).

^{1}H NMR (400 MHz, CDCl$_3$) δ ^{1}H NMR (500 MHz, CDCl$_3$) δ 8.73 (d, *J* = 4.2 Hz, 1H), 8.41 (d, *J* = 8.4 Hz, 1H), 7.60 (s, 1H), 7.41 – 7.35 (m, 8H), 7.25 (d, *J* = 4.1 Hz, 1H), 7.18 – 7.14 (m, 4H), 6.62 (s, 1H), 4.55 (s, 2H), 2.70 (s, 3H), 2.37 (s, 3H), 2.27 (s, 3H).

^{13}C NMR (100 MHz, CDCl$_3$) δ148.12, 144.49, 138.77, 138.26, 137.98, 135.60, 135.09, 131.76, 131.45, 131.07, 129.80, 129.37, 128.64, 128.31, 127.60, 127.08, 125.10, 124.26, 123.12, 122.14, 121.19, 120.19, 116.73, 25.92, 22.15, 21.73, 19.18.

HRMS (ESI-TOF) m/z: [M+H]$^+$calcd for $C_{33}H_{29}N_2O_2S$ 517.1944; Found 517.1956.

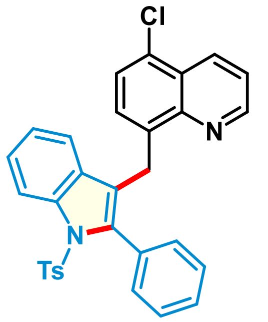

28, 59%

5-chloro-8-((2-phenyl-1-tosyl-1H-indol-3-yl)methyl)quinoline (28)

30.9 mg, 59% yield, white solid.

***R_f*: ** 0.50 (hexane/ethyl acetate, 9:1 v/v).

^{1}H NMR (400 MHz, CDCl$_3$) δ 8.98 (d, *J* = 2.8 Hz, 1H), 8.61 (d, *J* = 8.4 Hz, 1H), 8.42 (d, *J* = 8.4 Hz, 1H), 7.57 (dd, *J* = 8.4, 4.1 Hz, 1H), 7.44 – 7.38 (m, 2H), 7.37 – 7.36 (m, 5H), 7.29 (s, 1H), 7.24 (d, *J* = 7.8 Hz, 1H), 7.22 – 7.09 (m, 4H), 6.57 (d, *J* = 7.7 Hz, 1H), 4.52 (s, 2H), 2.42 (s, 3H).

^{13}C NMR (100 MHz, CDCl$_3$) δ 149.91, 146.99, 144.63, 139.02, 138.08, 137.65, 134.95, 133.54, 131.57, 131.27, 131.02, 129.52, 129.35, 128.75, 127.82, 127.67, 127.12, 126.37, 126.30, 125.30, 124.40, 122.42, 122.00, 119.90, 116.98, 25.61, 21.77.

HRMS (ESI-TOF) m/z: $[M+Na]^+$calcd for $C_{31}H_{23}ClN_2NaO_2S$ 545.1061; Found 545.1056.

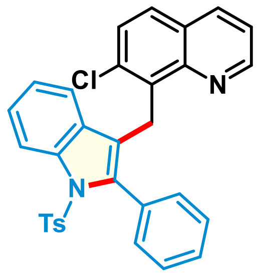

29, 48%

7-chloro-8-((2-phenyl-1-tosyl-1*H*-indol-3-yl)methyl)quinoline (29)

25.1 mg, 48% yield, white solid.

***R$_f$*:** 0.50 (hexane/ethyl acetate, 9:1 v/v).

^{1}H NMR (400 MHz, CDCl$_3$) δ 8.85 (dd, *J* = 4.0, 1.4 Hz, 1H), 8.31 (d, *J* = 8.4 Hz, 1H), 8.07 (dd, *J* = 8.2, 1.2 Hz, 1H), 7.55 (d, *J* = 8.7 Hz, 1H), 7.39 (dd, *J* = 8.2, 4.2 Hz, 1H), 7.36 (d, *J* = 8.7 Hz, 1H), 7.34 – 7.29 (m, 4H), 7.27 – 7.20 (m, 4H), 7.08 (d, *J* = 8.1 Hz, 2H), 7.02 (t, *J* = 7.5 Hz, 1H), 6.96 (d, *J* = 7.8 Hz, 1H), 4.76 (s, 2H), 2.35 (s, 3H).

^{13}C NMR (100 MHz, CDCl$_3$) δ 150.01, 144.42, 137.25, 137.19, 136.40, 135.66, 135.50, 131.79, 131.61, 131.03, 129.35, 128.40, 128.23, 127.37, 126.93, 126.84, 126.79, 124.53, 123.66, 121.35, 121.03, 119.75, 115.97, 24.41, 21.68.

HRMS (ESI-TOF) m/z: $[M+H]^+$calcd for $C_{31}H_{24}ClN_2O_2S$ 523.1242; Found 523.1274.

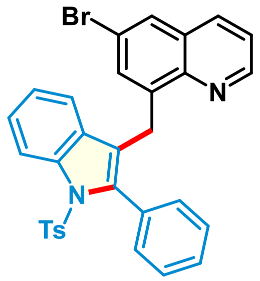

30, 52 %

6-bromo-8-((2-phenyl-1-tosyl-1H-indol-3-yl)methyl)quinoline (30)

29.5 mg, 52% yield, yellow solid.

***R$_f$*:** 0.60 (hexane/ethyl acetate, 9:1 v/v).

^{1}H NMR (400 MHz, CDCl$_3$) δ ^{1}H NMR (500 MHz, CDCl$_3$) δ 8.93 (dd, *J* = 4.0, 1.5 Hz, 1H), 8.41 (d, *J* = 8.4 Hz, 1H), 8.08 (dd, *J* = 8.3, 1.4 Hz, 1H), 7.83 (d, *J* = 1.6 Hz, 1H), 7.46 (dd, *J* = 8.3, 4.2 Hz, 1H), 7.41 – 7.35 (m, 8H), 7.22 – 7.15 (m, 4H), 6.89 (s, 1H), 4.54 (s, 2H), 2.37 (s, 3H).

^{13}C NMR (100 MHz, CDCl$_3$) δ 149.77, 145.31, 144.77, 141.06, 139.14, 137.98, 135.46, 134.96, 131.30, 131.24, 131.15, 131.04, 129.59, 129.48, 128.82, 128.35, 127.69, 126.82, 125.30, 124.37, 122.16, 121.87, 120.55, 119.90, 116.80, 25.64, 21.91.

HRMS (ESI-TOF) m/z: $[M+H]^+$calcd for $C_{31}H_{24}BrN_2O_2S$ 567.0736; Found 567.0741.

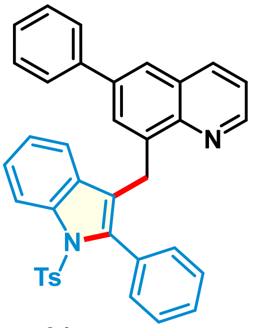

31, 64 %

6-phenyl-8-((2-(p-tolyl)-1-tosyl-1*H*-indol-3-yl)methyl)quinoline (31)

36.1 mg, 64% yield, white solid.

***R$_f$*:** 0.50 (hexane/ethyl acetate, 9:1 v/v).

^{1}H NMR (400 MHz, CDCl$_3$) δ 8.94 (d, *J* = 2.7 Hz, 1H), 8.40 (d, *J* = 8.4 Hz, 1H), 8.21 (d, *J* = 7.6 Hz, 1H), 7.82 (s, 1H), 7.46 (dd, *J* = 8.2, 4.2 Hz, 1H), 7.42 - 7.39 (m, 11H), 7.33 (d, *J* = 8.1 Hz, 2H), 7.30 (d, *J* = 7.9 Hz, 1H), 7.26 (s, 1H), 7.20 (t, *J* = 7.5 Hz, 1H), 6.95 (d, *J* = 8.0 Hz, 2H), 4.65 (s, 2H), 2.16 (s, 3H).

^{13}C NMR (100 MHz, CDCl$_3$) δ 149.40, 146.04, 144.56, 140.57, 138.94, 138.78, 138.69, 137.80, 136.65, 135.30, 131.35, 129.32, 128.96, 128.72, 128.54, 127.85, 127.72, 127.63, 127.42, 126.76, 125.17, 124.13, 124.09, 122.44, 121.58, 120.27, 116.45, 25.68, 21.56.

HRMS (ESI-TOF) m/z: $[M+H]^+$calcd for $C_{37}H_{29}N_2O_2S$ 565.1944; Found 565.1953.

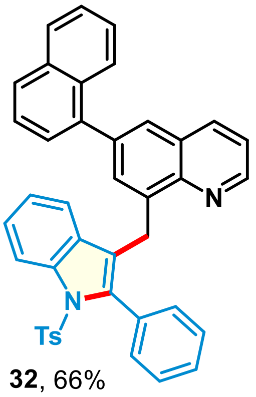

32, 66%

6-(naphthalen-1-yl)-8-((2-phenyl-1-tosyl-1*H*-indol-3-yl)methyl)quinoline (32)

40.6 mg, 66% yield, white solid.

R**f: 0.50 (hexane/ethyl acetate, 9:1 v/v).

^{1}H NMR (400 MHz, CDCl$_3$) δ 8.99 (dd, *J* = 4.1, 1.6 Hz, 1H), 8.36 (d, *J* = 8.4 Hz, 1H), 8.24 (dd, *J* = 8.2, 1.5 Hz, 1H), 7.93 (t, *J* = 8.0 Hz, 2H), 7.79 (d, *J* = 1.1 Hz, 1H), 7.56 (dd, *J* = 7.8, 4.7 Hz, 2H), 7.52 (dd, *J* = 7.9, 3.7 Hz, 1H), 7.51 – 7.48 (m, 1H), 7.45 (dd, *J* = 9.3, 5.1 Hz, 1H), 7.42 (d, *J* = 7.4 Hz, 1H), 7.39 – 7.30 (m, 4H), 7.29 – 7.28 (m, 3H), 7.18 – 7.07 (m, 2H), 7.00 (d, *J* = 8.2 Hz, 2H), 6.31 (d, *J* = 8.1 Hz, 2H), 4.67 (s, 2H), 1.84 (s, 3H).

^{13}C NMR (100 MHz, CDCl$_3$) δ 149.61, 146.09, 144.31, 139.45, 139.18, 138.92, 138.29, 137.73, 136.64, 134.99, 133.91, 131.40, 131.26, 131.21, 131.04, 130.54, 128.88, 128.70, 128.60, 128.46, 128.26, 127.52, 127.36, 127.19, 126.31, 126.24, 126.17, 125.76, 125.58, 125.20, 124.17, 122.10, 121.67, 120.28, 116.80, 25.96, 21.21.

HRMS (ESI-TOF) m/z: $[M+H]^+$calcd for $C_{41}H_{31}N_2O_2S$ 615.2102; Found 615.2127.

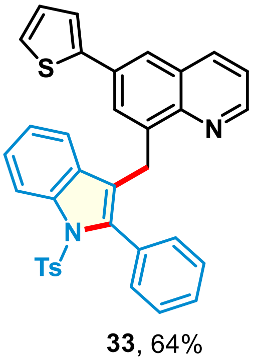

33, 64%

8-((2-phenyl-1-tosyl-1*H*-indol-3-yl)methyl)-6-(thiophen-2-yl)quinoline (33)

36.5 mg, 64% yield, yellow sticky liquid.

R**f: 0.50 (hexane/ethyl acetate, 9:1 v/v).

^{1}H NMR (400 MHz, CDCl$_3$) δ 8.89 (dd, *J* = 4.0, 1.4 Hz, 1H), 8.41 (d, *J* = 8.4 Hz, 1H), 8.15 (dd, *J* = 8.2, 1.3 Hz, 1H), 7.82 (s, 1H), 7.47 – 7.34 (m, 9H), 7.29 (t, *J* = 7.9 Hz, 3H), 7.18 (t, *J* = 7.5 Hz, 1H), 7.06 (d, *J* = 7.8 Hz, 3H), 6.98 (d, *J* = 3.4 Hz, 1H), 4.59 (s, 2H), 2.26 (s, 3H).
^{13}C NMR (100 MHz, CDCl$_3$) δ 149.30, 146.12, 144.57, 143.77, 139.01, 138.70, 137.66, 136.42, 135.43, 132.18, 131.39, 131.36, 131.27, 129.43, 128.79, 128.66, 128.25, 127.68, 126.92, 126.70, 125.64, 125.18, 124.14, 123.94, 122.34, 122.31, 121.80, 120.24, 116.36, 25.49, 21.67.
HRMS (ESI-TOF) m/z: [M+H]$^+$calcd for $C_{35}H_{27}N_2O_2S_2$ 571.1508; Found 571.1517.

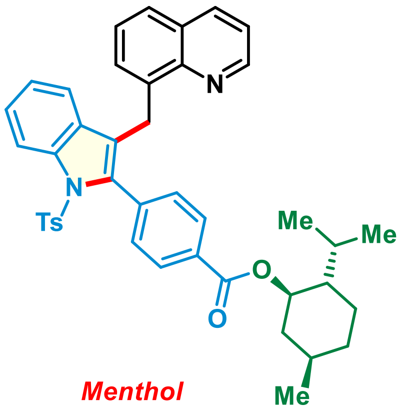

Menthol
34, 69%

(1*R*,2*R*,5*R*)-2-isopropyl-5-methylcyclohexyl 4-(3-(quinolin-8-ylmethyl)-1-tosyl-1*H*-indol-2-yl)benzoate (34)
46.3 mg, 69% yield, yellow sticky liquid.
***R*$_f$:** 0.40 (hexane/ethyl acetate, 9:1 v/v).
^{1}H NMR (400 MHz, CDCl$_3$) δ 8.94 (d, *J* = 2.8 Hz, 1H), 8.38 (d, *J* = 8.4 Hz, 1H), 8.20 (d, *J* = 7.8 Hz, 1H), 8.00 (d, *J* = 8.3 Hz, 2H), 7.66 (d, *J* = 8.1 Hz, 1H), 7.46 (t, *J* = 8.0 Hz, 3H), 7.43 – 7.36 (m, 1H), 7.34 (d, *J* = 8.3 Hz, 2H), 7.19 – 7.11 (m, 5H), 6.64 (d, *J* = 6.6 Hz, 1H), 4.93 (td, *J* = 10.8, 4.3 Hz, 1H), 4.57 (s, 2H), 2.39 (s, 3H), 2.12 (d, *J* = 11.8 Hz, 1H), 2.03 – 1.95 (m, 1H), 1.73 (d, *J* = 11.9 Hz, 2H), 1.59 – 1.49 (m, 2H), 1.15 – 1.06 (m, 2H), 0.92 (dd, *J* = 6.8, 1.7 Hz, 6H), 0.89 – 0.84 (m, 1H), 0.80 (d, *J* = 6.9 Hz, 3H).
^{13}C NMR (100 MHz, CDCl$_3$) δ 165.93, 144.81, 138.31, 137.88, 135.91, 134.58, 132.89, 131.80, 130.85, 130.78, 129.44, 128.87, 128.45, 127.06, 126.53, 125.66, 124.63, 121.29, 120.17, 117.07, 75.07, 47.42, 41.12, 34.46, 31.60, 26.47, 25.79, 23.64, 22.17, 21.76, 20.98, 16.53.
HRMS (ESI-TOF) m/z: [M+H]$^+$calcd for $C_{42}H_{43}N_2O_2S$ 671.2938; Found 671.2975.

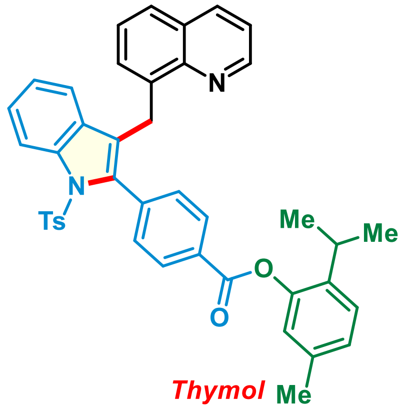

35, 66%

2-isopropyl-5-methylphenyl 4-(3-(quinolin-8-ylmethyl)-1-tosyl-1*H*-indol-2-yl)benzoate (35)

43.8 mg, 66% yield, brown sticky liquid.

R

$\boldsymbol{R_f}$**:** 0.40 (hexane/ethyl acetate, 9:1 v/v).

^{1}H NMR (400 MHz, CDCl$_3$) δ 8.93 (d, *J* = 2.8 Hz, 1H), 8.40 (d, *J* = 8.4 Hz, 1H), 8.21 – 8.10 (m, 3H), 7.66 (d, *J* = 8.1 Hz, 1H), 7.54 (d, *J* = 8.1 Hz, 2H), 7.45 (dd, *J* = 8.3, 4.3 Hz, 1H), 7.37 (d, *J* = 8.2 Hz, 2H), 7.25 (d, *J* = 9.4 Hz, 2H), 7.19 – 7.14 (m, 5H), 7.07 (d, *J* = 7.6 Hz, 1H), 6.93 (s, 1H), 6.60 (d, *J* = 7.1 Hz, 1H), 4.58 (s, 2H), 3.13 – 3.01 (m, 1H), 2.41 (s, 3H), 2.34 (s, 3H), 1.22 (d, *J* = 6.9 Hz, 6H).

^{13}C NMR (126 MHz, CDCl$_3$) δ 166.47, 149.53, 144.75, 142.66, 138.39, 137.92, 137.78, 136.52, 136.04, 134.48, 131.97, 130.82, 130.38, 129.38, 128.88, 128.32, 127.72, 127.04, 126.47, 126.17, 125.64, 124.70, 124.64, 124.45, 123.88, 121.34, 120.21, 118.43, 117.14, 92.48, 39.67, 26.42, 25.80, 21.76, 17.83, 16.70.

HRMS (ESI-TOF) m/z: [M+H]$^+$calcd for $C_{42}H_{37}N_2O_4S$ 665.2469; Found 665.2501.

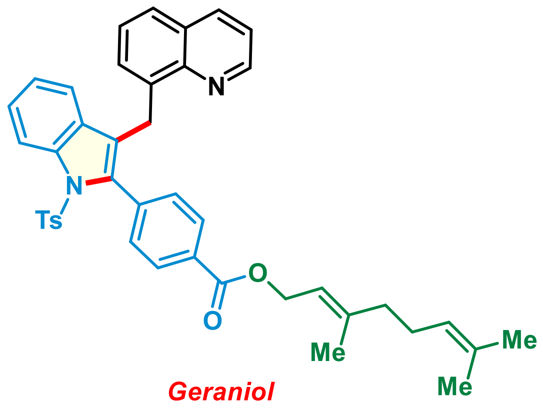

36, 64%

(*E*)-3,7-dimethylocta-2,6-dien-1-yl 4-(3-(quinolin-8-ylmethyl)-1-tosyl-1*H*-indol-2-yl)benzoate (36)

42.8 mg, 64% yield, brown sticky liquid.

R**f:** 0.40 (hexane/ethyl acetate, 9:1 v/v).

^{1}H NMR (500 MHz, CDCl$_3$) δ 8.94 (s, 1H), 8.41 (d, *J* = 8.4 Hz, 1H), 8.18 (d, *J* = 8.2 Hz, 1H), 8.05 (d, *J* = 8.4 Hz, 2H), 7.67 (d, *J* = 8.0 Hz, 1H), 7.47 (dd, *J* = 11.2, 6.2 Hz, 3H), 7.42 (ddd, *J* = 8.5, 5.8, 2.7 Hz, 1H), 7.34 (d, *J* = 8.3 Hz, 2H), 7.19 (dd, *J* = 8.3, 5.0 Hz, 2H), 7.18 – 7.14 (m, 3H), 6.59 (d, *J* = 7.0 Hz, 1H), 5.50 – 5.47 (m, 1H), 5.16 – 5.07 (m, 1H), 4.86 (d, *J* = 7.1 Hz, 2H), 4.57 (s, 2H), 2.42 (s, 3H), 2.19 – 2.12 (m, 2H), 2.12 – 2.06 (m, 2H), 1.79 (s, 3H), 1.70 (s, 3H), 1.63 (s, 3H).

^{13}C NMR (126 MHz, CDCl$_3$) δ 166.47, 149.53, 144.75, 142.66, 138.39, 137.92, 137.78, 136.52, 136.04, 134.48, 131.97, 130.82, 130.38, 129.38, 128.88, 128.32, 127.72, 127.04, 126.47, 126.17, 125.64, 124.64, 124.45, 123.88, 121.34, 120.21, 118.43, 117.14, 62.02, 39.67, 26.42, 25.80, 25.72, 21.76, 17.83, 16.70.

HRMS (ESI-TOF) m/z: [M+H]$^+$calcd for $C_{42}H_{41}N_2O_4S$ 669.2782; Found 669.2796.

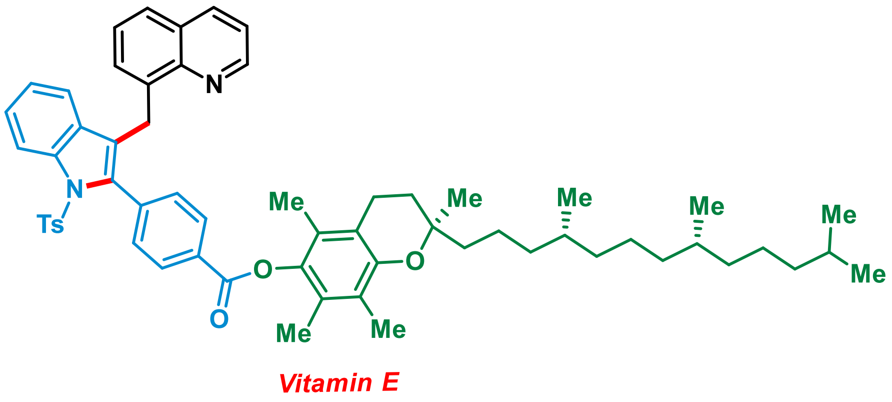

37, 60%

(*R*)-2,5,7,8-tetramethyl-2-((4*R*,8*R*)-4,8,12-trimethyltridecyl)chroman-6-yl 4-(3-(quinolin-8-ylmethyl)-1-tosyl-1*H*-indol-2-yl)benzoate (37)

56.7 mg, 60% yield, brown sticky liquid.

R**f:** 0.40 (hexane/ethyl acetate, 9:1 v/v).

^{1}H NMR (500 MHz, CDCl$_3$) δ 8.96 (s, 1H), 8.43 (d, *J* = 8.4 Hz, 1H), 8.26 – 8.17 (m, 3H), 7.74 – 7.63 (m, 2H), 7.56 (d, *J* = 7.8 Hz, 2H), 7.50 – 7.44 (m, 2H), 7.40 (d, *J* = 7.9 Hz, 2H), 7.21 – 7.15 (m, 4H), 6.62 (d, *J* = 6.9 Hz, 1H), 4.61 (s, 2H), 2.88 (s, 2H), 2.66 (d, *J* = 6.3 Hz, 2H), 2.44 (s, 3H), 2.16 (d, *J* = 8.9 Hz, 3H), 2.10 (s, 3H), 2.06 (s, 3H), 1.90 – 1.78 (m, 3H), 1.42 (s, 3H), 1.29 (s, 10H), 1.19 – 1.11 (m, 5H), 0.89 (t, *J* = 7.5 Hz, 15H).

^{13}C NMR (126 MHz, CDCl$_3$) δ 165.06, 149.64, 149.55, 144.82, 140.82, 138.48, 137.93, 137.74, 136.74, 134.52, 132.02, 131.03, 129.71, 129.49, 129.45, 128.39, 127.88, 127.52, 127.12, 126.53, 126.25, 126.09, 125.75, 125.33, 124.72, 123.26, 121.99, 121.38, 120.98, 120.28, 117.62, 117.21, 75.24, 39.53, 37.71, 37.62, 37.55, 37.45, 32.94, 32.87, 29.84, 28.13, 25.83, 24.96, 24.60, 22.86, 22.77, 21.78, 21.22, 20.79, 19.91, 19.84, 18.41, 13.30, 12.46, 11.99.

HRMS (ESI-TOF) m/z: [M+H]$^+$calcd for $C_{61}H_{73}N_2O_5S$ 945.5225; Found 945.5235

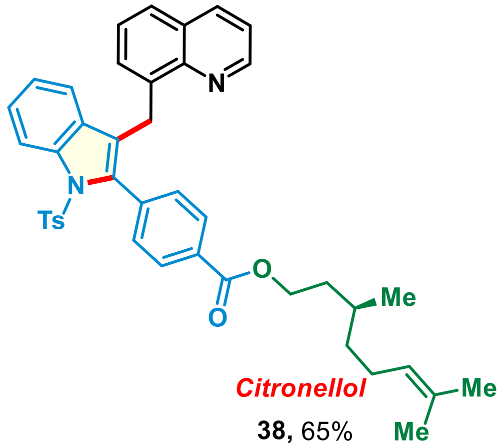

38, 65%

3,7-dimethyloct-6-en-1-yl 4-(3-(quinolin-8-ylmethyl)-1-tosyl-1*H*-indol-2-yl)benzoate (38)

43.6 mg, 65% yield, brown sticky liquid.

***R$_f$*:** 0.40 (hexane/ethyl acetate, 9:1 v/v).

^{1}H NMR (400 MHz, CDCl$_3$) δ 8.92 (d, *J* = 2.8 Hz, 1H), 8.39 (d, *J* = 8.4 Hz, 1H), 8.16 (d, *J* = 8.2 Hz, 1H), 8.00 (d, *J* = 8.2 Hz, 2H), 7.65 (d, *J* = 8.2 Hz, 1H), 7.44 (t, *J* = 7.5 Hz, 3H), 7.43 - 7.37 (m, 1H), 7.32 (d, *J* = 8.2 Hz, 2H), 7.17 – 7.11 (m, 5H), 6.57 (d, *J* = 7.0 Hz, 1H), 5.10 (t, *J* = 7.0 Hz, 1H), 4.54 (s, 2H), 4.41 – 4.28 (m, 2H), 2.39 (s, 3H), 2.00 (dd, *J* = 16.6, 7.8 Hz, 2H), 1.80 (dt, *J* = 12.8, 6.5 Hz, 1H), 1.65 (s, 3H), 1.59 (s, 3H), 1.45 – 1.34 (m, 1H), 1.28 - 1.18 (m, 2H), 0.97 (d, *J* = 6.5 Hz, 3H).

^{13}C NMR (101 MHz, CDCl$_3$) δ 166.52, 149.52, 144.77, 138.40, 137.79, 136.07, 134.51, 131.95, 131.52, 130.86, 130.38, 129.40, 128.84, 128.34, 127.78, 127.06, 126.48, 126.21, 125.66, 124.71, 124.66, 124.45, 121.34, 120.22, 117.15, 37.12, 35.65, 29.63, 25.83, 25.74, 25.52, 21.77, 19.61, 17.81.

HRMS (ESI-TOF) m/z: [M+H]$^+$calcd for $C_{42}H_{43}N_2O_4S$ 671.2938; Found 671.2967.

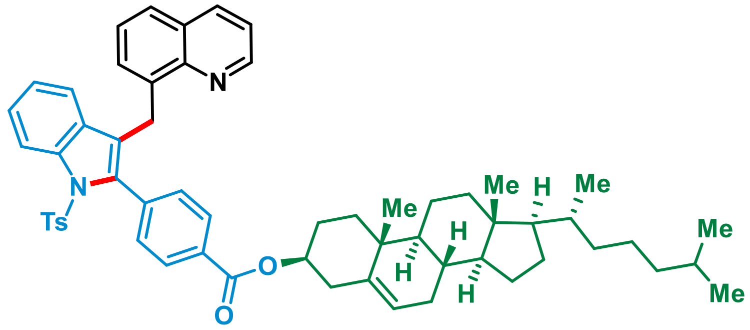

Cholesterol

39, 62%

(3*S*,8*S*,9*S*,10*R*,13*R*,14*S*,17*R*)-10,13-dimethyl-17-((*R*)-6-methylheptan-2-yl)-2,3,4,7,8,9,10,11,12,13,14,15,16,17-tetradecahydro-1*H*-cyclopenta[a]phenanthren-3-yl 4-(3-(quinolin-8-ylmethyl)-1-tosyl-1*H*-indol-2-yl)benzoate (39)

55.9 mg, 62% yield, white solid.

***R_f*:** 0.40 (hexane/ethyl acetate, 9:1 v/v).

^{1}H NMR (500 MHz, $CDCl_3$) δ 8.93 (dd, *J* = 4.1, 1.6 Hz, 1H), 8.41 (d, *J* = 8.4 Hz, 1H), 8.17 (dd, *J* = 8.3, 1.5 Hz, 1H), 8.03 (d, *J* = 8.4 Hz, 2H), 7.66 (d, *J* = 8.1 Hz, 1H), 7.46 (dd, *J* = 12.5, 6.3 Hz, 3H), 7.41 (ddd, *J* = 8.4, 5.6, 2.9 Hz, 1H), 7.35 (d, *J* = 8.3 Hz, 2H), 7.19 (dd, *J* = 7.7, 5.1 Hz, 2H), 7.15 (t, *J* = 8.3 Hz, 3H), 6.60 (d, *J* = 7.0 Hz, 1H), 5.44 (d, *J* = 3.6 Hz, 1H), 4.99 – 4.73 (m, 1H), 4.56 (s, 2H), 2.48 (d, *J* = 7.8 Hz, 2H), 2.42 (s, 3H), 2.03 (t, *J* = 15.8 Hz, 3H), 1.93 (t, *J* = 11.7 Hz, 1H), 1.89 – 1.83 (m, 1H), 1.79 – 1.70 (m, 1H), 1.60 (d, *J* = 14.1 Hz, 3H), 1.57 – 1.48 (m, 4H), 1.37 (d, *J* = 9.1 Hz, 3H), 1.31 – 1.28 (m, 2H), 1.27 – 1.20 (m, 3H), 1.14 (dd, *J* = 10.3, 8.4 Hz, 3H), 1.09 (s, 3H), 1.05 – 1.01 (m, 2H), 0.95 (d, *J* = 6.5 Hz, 3H), 0.90 (dd, *J* = 6.6, 2.1 Hz, 6H), 0.72 (s, 3H).

^{13}C NMR (126 MHz, $CDCl_3$) δ 165.84, 149.58, 146.62, 144.75, 139.82, 138.42, 138.03, 137.83, 136.46, 136.02, 134.61, 131.99, 130.84, 130.74, 129.41, 128.86, 128.34, 127.75, 127.08, 126.46, 126.17, 125.64, 124.64, 124.47, 122.94, 121.33, 120.24, 117.16, 74.81, 56.87, 56.32, 50.23, 42.49, 39.92, 39.68, 38.38, 37.20, 36.82, 36.36, 35.96, 32.10, 32.05, 28.39, 28.17, 28.05, 25.74, 24.45, 23.99, 22.97, 22.71, 21.78, 21.21, 19.52, 18.88, 12.02.

HRMS (ESI-TOF) m/z: $[M+H]^+$calcd for $C_{59}H_{69}N_2O_4S$ 901.4973; Found 901.4966.

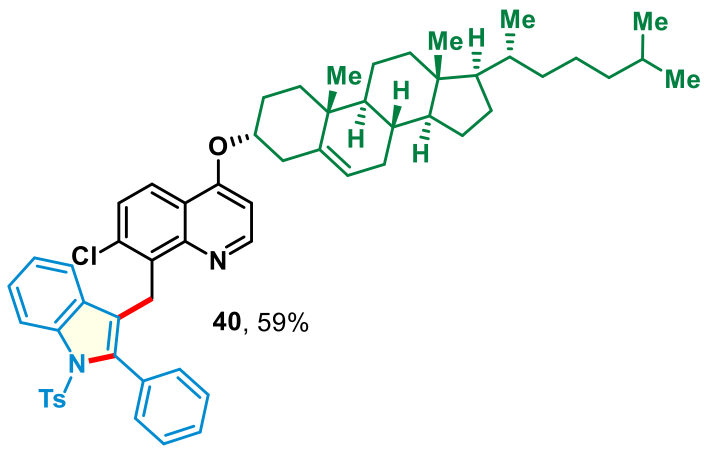

40, 59%

7-chloro-4-(((3*S*,8*S*,9*S*,10*R*,13*R*,14*S*,17*R*)-10,13-dimethyl-17-((*R*)-6-methylheptan-2-yl)-2,3,4,7,8,9,10,11,12,13,14,15,16,17-tetradecahydro-1*H*-cyclopenta[a]phenanthren-3-yl)oxy)-8-((2-phenyl-1-tosyl-1*H*-indol-3-yl)methyl)quinoline (40)

53.6 mg, 59% yield, brown sticky liquid.

***R$_f$*: ** 0.30 (hexane/ethyl acetate, 9:1 v/v).

^{1}H NMR (500 MHz, CDCl$_3$) δ 8.67 (d, *J* = 5.2 Hz, 1H), 8.30 (d, *J* = 8.4 Hz, 1H), 7.90 (d, *J* = 8.9 Hz, 1H), 7.33 (dd, *J* = 11.1, 8.1 Hz, 4H), 7.26 (dt, *J* = 7.1, 5.1 Hz, 5H), 7.08 (d, *J* = 8.1 Hz, 2H), 7.00 (t, *J* = 7.5 Hz, 1H), 6.93 (d, *J* = 7.8 Hz, 1H), 6.70 (d, *J* = 5.3 Hz, 1H), 5.29 (d, *J* = 4.7 Hz, 1H), 4.85 (s, 1H), 4.78 – 4.65 (m, 2H), 2.67 (d, *J* = 15.0 Hz, 1H), 2.48 (d, *J* = 15.0 Hz, 1H), 2.34 (s, 3H), 2.08 (dd, *J* = 25.4, 13.5 Hz, 2H), 2.03 – 1.91 (m, 2H), 1.90 – 1.84 (m, 1H), 1.74 (t, *J* = 11.0 Hz, 1H), 1.64 – 1.47 (m, 8H), 1.44 – 1.25 (m, 6H), 1.23 – 1.14 (m, 4H), 1.12 (s, 3H), 1.05 (dd, *J* = 21.3, 9.5 Hz, 3H), 0.95 (d, *J* = 6.5 Hz, 3H), 0.90 (dd, *J* = 6.6, 2.1 Hz, 7H), 0.72 (s, 3H).

^{13}C NMR (126 MHz, CDCl$_3$) δ 160.63, 150.92, 137.96, 137.29, 137.17, 136.40, 135.59, 134.60, 131.90, 131.64, 131.10, 129.32, 128.19, 127.05, 126.91, 126.86, 124.45, 123.65, 123.06, 121.90, 121.57, 120.89, 119.93, 115.95, 101.98, 73.76, 56.81, 56.28, 50.51, 42.44, 39.85, 39.65, 37.20, 36.31, 36.26, 35.94, 33.88, 32.09, 31.98, 28.36, 28.15, 25.86, 24.66, 24.39, 23.96, 22.96, 22.70, 21.67, 20.96, 19.14, 18.86, 12.01.

HRMS (ESI-TOF) m/z: [M+H]$^+$calcd for $C_{58}H_{68}ClN_2O_3S$ 907.4634; Found 907.4652.

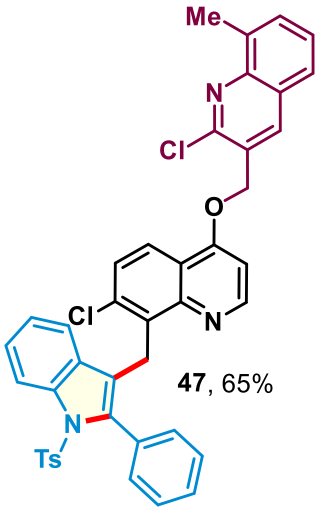

47, 65%

2-chloro-3-(((7-chloro-8-((2-phenyl-1-tosyl-1H-indol-3-yl)methyl)quinolin-4-yl)oxy)methyl)-8-methylquinoline (47)

47.3 mg, 65% yield, brown sticky liquid.

RR_f**:** 0.40 (hexane/ethyl acetate, 9:1 v/v).

^{1}H NMR (400 MHz, $CDCl_3$) δ 8.71 (d, *J* = 5.1 Hz, 1H), 8.31 (d, *J* = 8.3 Hz, 2H), 8.07 (d, *J* = 8.9 Hz, 1H), 7.72 (d, *J* = 8.1 Hz, 1H), 7.64 (d, *J* = 6.9 Hz, 1H), 7.51 (t, *J* = 7.6 Hz, 1H), 7.33 (dd, *J* = 11.8, 8.7 Hz, 5H), 7.29 - 7.22 (m, 4H), 7.08 (d, *J* = 8.1 Hz, 2H), 7.02 (t, *J* = 7.5 Hz, 1H), 6.97 (d, *J* = 7.8 Hz, 1H), 6.85 (d, *J* = 5.1 Hz, 1H), 5.50 (s, 2H), 4.74 (s, 2H), 2.83 (s, 3H), 2.34 (s, 3H).

^{13}C NMR (100 MHz, $CDCl_3$) δ 161.03, 151.03, 148.98, 147.86, 146.77, 144.41, 137.54, 137.24, 137.18, 136.81, 136.72, 135.61, 135.14, 131.81, 131.62, 131.16, 131.02, 129.33, 128.20, 127.58, 127.49, 127.19, 127.11, 126.90, 126.84, 125.68, 124.51, 123.67, 121.38, 121.34, 119.80, 119.75, 115.95, 101.39, 67.28, 24.72, 21.66, 17.95.

HRMS (ESI-TOF) m/z: $[M+H]^+$calcd for $C_{42}H_{32}Cl_2N_3O_3S$ 728.1536; Found 728.1545.

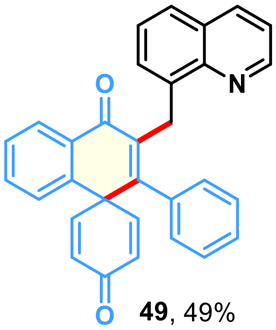

49, 49%

2'-phenyl-3'-(quinolin-8-ylmethyl)-4'*H*-spiro[cyclohexane-1,1'-naphthalene]-2,5-diene-4,4'-dione (49)

21.5 mg, 49% yield, yellow sticky liquid.

Rf**:** 0.50 (hexane/ethyl acetate, 4:6 v/v).

^{1}H NMR (500 MHz, CDCl$_3$) δ 8.82 (dd, *J* = 4.0, 1.5 Hz, 1H), 8.31 (d, *J* = 7.7 Hz, 1H), 8.11 (d, *J* = 8.2 Hz, 1H), 7.68 – 7.58 (m, 2H), 7.55 (t, *J* = 7.2 Hz, 1H), 7.43 – 7.29 (m, 4H), 7.19 – 7.14 (m, 1H), 7.11 (t, *J* = 7.4 Hz, 2H), 6.95 (d, *J* = 7.2 Hz, 2H), 6.86 (d, *J* = 10.0 Hz, 2H), 6.34 (d, *J* = 10.0 Hz, 2H), 4.42 (s, 2H).

^{13}C NMR (126 MHz, CDCl$_3$) δ 185.19, 183.71, 153.28, 149.64, 149.20, 146.47, 139.21, 138.72, 138.27, 136.35, 136.19, 133.20, 130.76, 130.14, 129.02, 128.37, 128.34, 128.21, 127.80, 127.76, 127.02, 126.23, 126.16, 121.02, 51.12, 29.30.

HRMS (ESI-TOF) m/z: [M+H]$^+$calcd for $C_{31}H_{22}NO_2$ 440.1645; Found 440.1671.

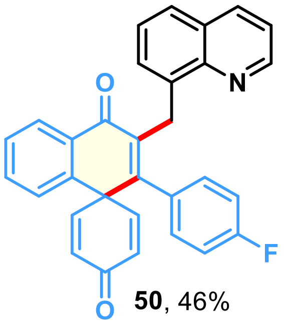

50, 46%

2'-(4-fluorophenyl)-3'-(quinolin-8-ylmethyl)-4'*H*-spiro[cyclohexane-1,1'-naphthalene]-2,5-diene-4,4'-dione (50)

21.0 mg, 46% yield, yellow sticky liquid.

Rf**:** 0.50 (hexane/ethyl acetate, 4:6 v/v).

^{1}H NMR (500 MHz, CDCl$_3$) δ 8.79 (dd, *J* = 4.0, 1.5 Hz, 1H), 8.32 (d, *J* = 6.8 Hz, 1H), 8.11 (dd, *J* = 8.2, 1.4 Hz, 1H), 7.62 (ddd, *J* = 11.1, 8.8, 4.7 Hz, 2H), 7.56 (t, *J* = 7.2 Hz, 1H), 7.41 – 7.35 (m, 2H), 7.33 (d, *J* = 7.6 Hz, 1H), 7.29 (s, 1H), 6.87 (dd, *J* = 8.5, 5.4 Hz, 2H), 6.82 (d, *J* = 10.0 Hz, 2H), 6.77 (t, *J* = 8.6 Hz, 2H), 6.34 (d, *J* = 9.9 Hz, 2H), 4.43 (s, 2H).

^{13}C NMR (126 MHz, CDCl$_3$) δ 184.95, 183.62, 162.43 (d, *J* = 248.3 Hz), 152.04, 149.43, 149.21, 146.39, 140.05, 138.65, 138.17, 136.33, 133.28, 132.09 (d, *J* = 3.5 Hz), 130.63, 130.26, 129.71 (d, *J* = 8.2 Hz), 129.09, 128.35, 128.33, 128.27, 127.23, 126.26, 126.22, 121.07, 114.91 (d, *J* = 21.7 Hz), 51.08, 29.06.

^{19}F NMR (376 MHz, CDCl$_3$) δ -113.15.

HRMS (ESI-TOF) m/z: [M+H]$^+$calcd for $C_{31}H_{21}FNO_2$ 458.1551; Found 458.1561.

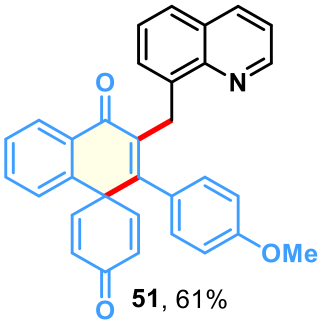

51, 61%

2'-(4-methoxyphenyl)-3'-(quinolin-8-ylmethyl)-4'*H*-spiro[cyclohexane-1,1'naphthalene]-2,5-diene-4,4'-dione (51)

28.6 mg, 61% yield, yellow solid.

***R_f*:** 0.50 (hexane/ethyl acetate, 4:6 v/v).

^{1}H NMR (500 MHz, $CDCl_3$) δ 8.82 (dd, *J* = 4.2, 1.7 Hz, 1H), 8.30 (dd, *J* = 7.8, 1.3 Hz, 1H), 8.11 (dd, *J* = 8.3, 1.7 Hz, 1H), 7.64 (d, *J* = 7.9 Hz, 1H), 7.61 – 7.58 (m, 1H), 7.56 – 7.52 (m, 1H), 7.43 – 7.30 (m, 4H), 6.93 – 6.78 (m, 4H), 6.69 – 6.57 (m, 2H), 6.47 – 6.19 (m, 2H), 4.45 (s, 2H), 3.70 (s, 3H).

^{13}C NMR (126 MHz, $CDCl_3$) δ 185.26, 183.76, 159.35, 153.34, 149.84, 149.16, 146.51, 139.62, 138.87, 138.34, 136.32, 133.11, 130.79, 130.09, 128.97, 128.95, 128.65, 128.36, 128.31, 128.18, 127.02, 126.22, 126.11, 120.98, 113.24, 55.19, 51.41, 29.25.

HRMS (ESI-TOF) m/z: $[M+H]^+$calcd for $C_{32}H_{24}NO_3$ 470.1751; Found 470.1763.

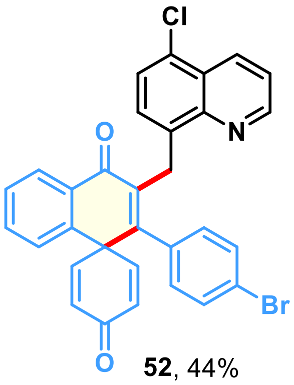

52, 44%

2'-(4-bromophenyl)-3'-((5-chloroquinolin-8-yl)methyl)-4'*H*-spiro[cyclohexane-1,1'-naphthalene]-2,5-diene-4,4'-dione (52)

24.3 mg, 44% yield, yellow solid.

***R_f*:** 0.50 (hexane/ethyl acetate, 4:6 v/v).

^{1}H NMR (500 MHz, $CDCl_3$) δ 8.84 (dd, *J* = 4.1, 1.6 Hz, 1H), 8.54 (dd, *J* = 8.5, 1.6 Hz, 1H), 8.30 (dd, *J* = 7.7, 1.3 Hz, 1H), 7.61 (dd, *J* = 7.6, 1.4 Hz, 1H), 7.55 (dd, *J* = 10.7, 4.3 Hz, 1H), 7.50 – 7.45 (m, 2H), 7.34 (d, *J* = 7.8 Hz, 1H), 7.22 (d, *J* = 7.8 Hz, 1H), 7.19 – 7.15 (m, 1H),

7.13 (t, J = 7.3 Hz, 2H), 6.97 – 6.91 (m, 2H), 6.84 (d, J = 10.0 Hz, 2H), 6.34 (d, J = 10.0 Hz, 2H), 4.38 (s, 2H).

^{13}C NMR (126 MHz, CDCl$_3$) δ 185.08, 183.68, 153.56, 149.68, 149.43, 146.98, 138.90, 138.30, 138.26, 136.12, 133.30, 133.11, 130.66, 130.21, 129.29, 129.06, 128.42, 128.36, 128.20, 127.85, 127.72, 126.96, 126.29, 126.25, 121.77, 51.10, 29.22.

HRMS (ESI-TOF) m/z: $[M+H]^+$calcd for $C_{31}H_{20}BrClNO_2$ 552.0360; Found 552.0371.

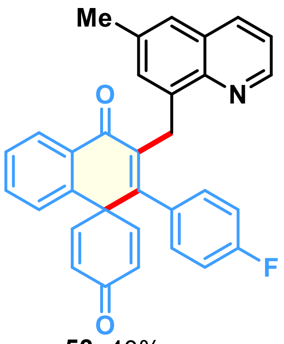

53, 40%

2'-(4-fluorophenyl)-3'-((6-methylquinolin-8-yl)methyl)-4'H-spiro[cyclohexane-1,1'-naphthalene]-2,5-diene-4,4'-dione (53)

18.9 mg, 40% yield, yellow solid.
***R$_f$*:** 0.50 (hexane/ethyl acetate, 4:6 v/v).

^{1}H NMR (500 MHz, CDCl$_3$) δ 8.81 (s, 1H), 8.31 (dd, J = 12.3, 8.3 Hz, 2H), 7.66 – 7.51 (m, 2H), 7.43 – 7.38 (m, 1H), 7.33 (d, J = 7.4 Hz, 1H), 7.22 (d, J = 7.1 Hz, 1H), 7.17 (d, J = 7.3 Hz, 1H), 6.93 – 6.87 (m, 2H), 6.87 – 6.72 (m, 4H), 6.35 (d, J = 9.4 Hz, 2H), 4.38 (s, 2H), 2.65 (s, 3H).

^{13}C NMR (126 MHz, CDCl$_3$) δ 185.01, 183.65, 162.45 (d, J = 246.3 Hz), 161.46, 151.92, 149.54, 148.68, 146.57, 140.10, 138.16, 136.45, 133.25, 132.76, 130.69, 130.24, 129.73 (d, J = 8.8 Hz), 129.08, 128.31 (d, J = 8.8 Hz), 127.73, 126.82, 126.68, 120.65, 114.99, 114.82, 113.77, 51.10, 29.17, 18.65.

^{19}F NMR (376 MHz, CDCl$_3$) δ -113.19.

HRMS (ESI-TOF) m/z: $[M+H]^+$calcd for $C_{32}H_{23}FNO_2$ 472.1707; Found 472.1743.

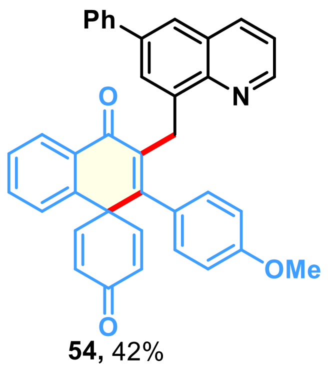

54, 42%

2'-(4-methoxyphenyl)-3'-((6-phenylquinolin-8-yl)methyl)-4'H-spiro[cyclohexane-1,1'-naphthalene]-2,5-diene-4,4'-dione (54)

22.9 mg, 42% yield, yellow solid.
***Rf*:** 0.50 (hexane/ethyl acetate, 4:6 v/v).

^{1}H NMR (500 MHz, $CDCl_3$) δ 8.80 (s, 1H), 8.33 (s, 1H), 8.16 (d, *J* = 6.6 Hz, 1H), 7.81 (s, 1H), 7.63 – 7.56 (m, 3H), 7.48 (d, *J* = 5.6 Hz, 3H), 7.40 (d, *J* = 5.9 Hz, 2H), 7.30 (t, *J* = 7.5 Hz, 3H), 6.83 – 6.76 (m, 3H), 6.62 (d, *J* = 6.1 Hz, 2H), 6.40 – 6.23 (m, 2H), 4.50 (d, *J* = 5.0 Hz, 2H), 3.66 (d, *J* = 6.1 Hz, 3H).

^{13}C NMR (126 MHz, $CDCl_3$) δ 185.25, 183.56, 159.52, 149.85, 149.11, 140.90, 139.97, 139.52, 139.48, 138.78, 136.47, 133.08, 130.08, 129.06, 128.98, 128.55, 128.27, 127.71, 127.56, 127.48, 127.43, 123.95, 123.93, 121.32, 114.09, 113.27, 55.14, 51.42, 29.41.

HRMS (ESI-TOF) m/z: $[M+H]^+$calcd for $C_{38}H_{28}NO_3$ 546.2064; Found 546.2072.

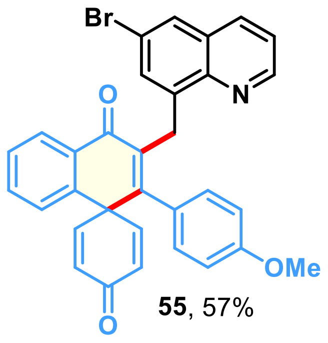

55, 57%

3'-((6-bromoquinolin-8-yl)methyl)-2'-(4-methoxyphenyl)-4'*H*-spiro[cyclohexane-1,1'-naphthalene]-2,5-diene-4,4'-dione (55)

31.2 mg, 57% yield, yellow sticky liquid.

***Rf*:** 0.50 (hexane/ethyl acetate, 4:6 v/v).

^{1}H NMR (500 MHz, $CDCl_3$) δ 8.80 (dd, *J* = 4.1, 1.4 Hz, 1H), 8.32 (d, *J* = 7.5 Hz, 1H), 8.01 (dd, *J* = 8.3, 1.5 Hz, 1H), 7.79 (d, *J* = 1.9 Hz, 1H), 7.61 – 7.56 (m, 2H), 7.38 (dd, *J* = 8.3, 4.2

Hz, 1H), 7.33 (d, J = 7.7 Hz, 1H), 7.28 (d, J = 1.1 Hz, 1H), 6.80 (d, J = 9.1 Hz, 4H), 6.63 (d, J = 8.5 Hz, 2H), 6.34 (d, J = 9.8 Hz, 2H), 4.41 (s, 2H), 3.72 (s, 3H).

^{13}C NMR (126 MHz, CDCl$_3$) δ 185.21, 183.55, 159.44, 153.68, 149.61, 149.43, 145.14, 141.48, 139.21, 138.32, 135.30, 133.24, 130.78, 130.64, 130.19, 129.43, 129.04, 128.95, 128.45, 128.37, 128.26, 128.02, 121.84, 120.30, 113.31, 55.22, 51.39, 29.01.

HRMS (ESI-TOF) m/z: $[M+H]^+$calcd for $C_{32}H_{23}BrNO_3$ 548.0856; Found 548.0887.

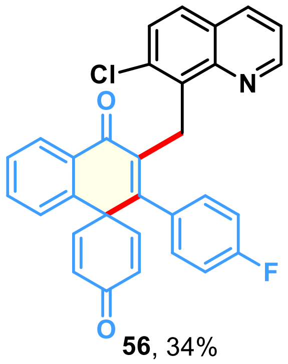

56, 34%

3'-((7-chloroquinolin-8-yl)methyl)-2'-(4-fluorophenyl)-4'*H*-spiro[cyclohexane-1,1'-naphthalene]-2,5-diene-4,4'-dione (56)

16.7 mg, 34% yield, yellow sticky liquid.

***R$_f$*:** 0.50 (hexane/ethyl acetate, 4:6 v/v).

^{1}H NMR (500 MHz, CDCl$_3$) δ 8.71 (dd, J = 4.2, 1.7 Hz, 1H), 8.42 – 8.35 (m, 1H), 8.00 (dd, J = 8.2, 1.7 Hz, 1H), 7.59 – 7.55 (m, 2H), 7.52 (d, J = 8.8 Hz, 1H), 7.35 (d, J = 8.8 Hz, 1H), 7.33 – 7.30 (m, 1H), 7.27 – 7.23 (m, 1H), 6.69 (d, J = 10.1 Hz, 2H), 6.61 – 6.54 (m, 2H), 6.43 (t, J = 8.7 Hz, 2H), 6.22 (d, J = 10.1 Hz, 2H), 4.75 (s, 2H).

^{13}C NMR (126 MHz, CDCl$_3$) δ 185.10, 183.35, 161.78 (d, J = 241.3 Hz), 149.67, 149.42, 148.90, 140.05, 137.88, 137.14, 136.04, 134.68, 133.04, 131.90, 130.46, 129.95, 129.93, 129.39 (d, J = 8.8 Hz), 128.97, 128.42, 128.18, 128.08, 126.98, 126.64, 120.95, 113.95 (d, J = 21.3 Hz), 51.12, 26.40.

^{19}F NMR (376 MHz, CDCl$_3$) δ -114.18.

HRMS (ESI-TOF) m/z: $[M+H]^+$calcd for $C_{31}H_{20}ClFNO_2$ 492.1161; Found 492.1165.

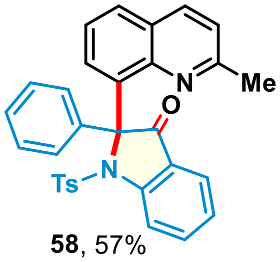

58, 57%

2-(2-methylquinolin-8-yl)-2-phenyl-1-tosylindolin-3-one (58)

28.8 mg, 57% yield, white solid.

***R*$_f$: ** 0.50 (hexane/ethyl acetate, 8:2 v/v).

^{1}H NMR (500 MHz, CDCl$_3$) δ 8.41 (d, *J* = 8.3 Hz, 1H), 7.87 (dd, *J* = 7.4, 1.2 Hz, 3H), 7.77 – 7.70 (m, 4H), 7.52 (t, *J* = 7.7 Hz, 1H), 7.45 (t, *J* = 7.5 Hz, 2H), 7.42 – 7.37 (m, 1H), 7.24 (td, *J* = 7.5, 0.6 Hz, 1H), 6.80 (d, *J* = 8.4 Hz, 1H), 6.60 (d, *J* = 8.3 Hz, 2H), 6.41 (d, *J* = 8.0 Hz, 2H), 2.06 (s, 3H), 1.68 (s, 3H).

^{13}C NMR (126 MHz, CDCl$_3$) δ 197.01, 156.74, 151.25, 145.64, 142.62, 136.60, 136.36, 136.32, 136.21, 135.67, 135.58, 128.67, 128.23, 128.18, 127.96, 126.19, 125.13, 124.99, 124.57, 123.28, 121.32, 114.98, 79.43, 23.19, 21.19.

HRMS (ESI-TOF) m/z: $[M+H]^+$calcd for $C_{31}H_{25}N_2O_3S$ 505.1580; Found 505.1555.

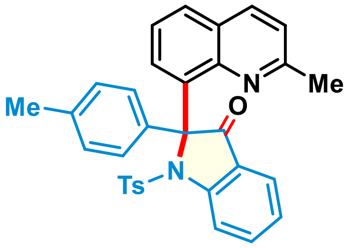

59, 59%

2-(2-methylquinolin-8-yl)-2-(p-tolyl)-1-tosylindolin-3-one (59)

30.6 mg, 59% yield, white solid.

***R*$_f$: ** 0.50 (hexane/ethyl acetate, 8:2 v/v).

^{1}H NMR (500 MHz, CDCl$_3$) δ 8.39 (d, *J* = 8.3 Hz, 1H), 7.95 – 7.82 (m, 2H), 7.77 – 7.68 (m, 4H), 7.52 (t, *J* = 7.7 Hz, 1H), 7.34 – 7.15 (m, 4H), 6.80 (d, *J* = 8.4 Hz, 1H), 6.60 (d, *J* = 8.3 Hz, 2H), 6.41 (d, *J* = 8.1 Hz, 2H), 2.40 (s, 3H), 2.05 (s, 3H), 1.68 (s, 3H).

^{13}C NMR (126 MHz, CDCl$_3$) δ 197.11, 156.70, 151.25, 145.65, 142.55, 138.04, 136.66, 136.33, 136.25, 135.64, 135.51, 133.20, 128.96, 128.62, 127.93, 126.18, 125.15, 125.11, 124.99, 124.61, 123.21, 121.28, 114.93, 79.30, 23.21, 21.18.

HRMS (ESI-TOF) m/z: $[M+H]^+$calcd for $C_{32}H_{27}N_2O_3S$ 519.1737; Found 519.1772.

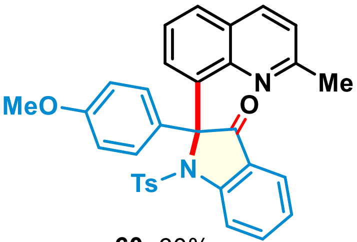

60, 60%

2-(4-methoxyphenyl)-2-(2-methylquinolin-8-yl)-1-tosylindolin-3-one (60)

32.1 mg, 60% yield, brown sticky liquid.

***Rf*: 0.40** (hexane/ethyl acetate, 8:2 v/v).

^{1}H NMR (500 MHz, $CDCl_3$) δ 8.39 (d, *J* = 8.4 Hz, 1H), 7.88 (dd, *J* = 7.4, 1.2 Hz, 2H), 7.79 – 7.70 (m, 4H), 7.53 (t, *J* = 7.7 Hz, 1H), 7.23 (t, *J* = 7.2 Hz, 1H), 6.98 (dd, *J* = 7.7, 1.5 Hz, 2H), 6.80 (d, *J* = 8.4 Hz, 1H), 6.60 (d, *J* = 8.3 Hz, 2H), 6.41 (d, *J* = 8.1 Hz, 2H), 3.85 (s, 3H), 2.06 (s, 3H), 1.68 (s, 3H).

^{13}C NMR (126 MHz, $CDCl_3$) δ 197.16, 159.70, 156.71, 151.20, 145.64, 142.58, 136.62, 136.32, 136.23, 135.64, 135.50, 132.58, 130.00, 128.64, 127.95, 127.49, 126.19, 125.15, 125.01, 124.62, 123.25, 121.29, 114.95, 114.63, 78.97, 55.44, 23.22, 21.19.

HRMS (ESI-TOF) m/z: $[M+H]^+$calcd for $C_{32}H_{27}N_2O_4S$ 535.1686; Found 535.1694.

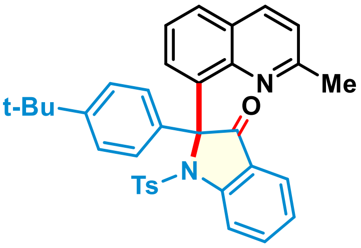

61, 62%

2-(4-(tert-butyl)phenyl)-2-(2-methylquinolin-8-yl)-1-tosylindolin-3-one (61)

34.7 mg, 62% yield, white solid.

***Rf*:** 0.50 (hexane/ethyl acetate, 8:2 v/v).

^{1}H NMR (400 MHz, $CDCl_3$) δ 8.37 (d, *J* = 8.3 Hz, 1H), 7.90 – 7.84 (m, 3H), 7.72 (dd, *J* = 12.2, 5.8 Hz, 4H), 7.50 (t, *J* = 7.7 Hz, 1H), 7.43 (d, *J* = 8.6 Hz, 2H), 7.20 (t, *J* = 7.4 Hz, 1H), 6.77 (d, *J* = 8.4 Hz, 1H), 6.58 (d, *J* = 8.1 Hz, 2H), 6.39 (d, *J* = 8.1 Hz, 2H), 2.03 (s, 3H), 1.65 (s, 3H), 1.34 (s, 9H).

^{13}C NMR (101 MHz, $CDCl_3$) δ 197.21, 156.69, 151.28, 151.00, 145.65, 142.55, 136.67, 136.37, 136.26, 135.65, 135.51, 133.02, 128.62, 127.94, 126.19, 125.24, 125.22, 125.14, 124.99, 124.58, 123.20, 121.27, 114.94, 79.38, 34.64, 31.45, 23.21, 21.19.

HRMS (ESI-TOF) m/z: $[M+Na]^+$calcd for $C_{35}H_{32}N_2NaO_3S$ 583.2026; Found 586.2033.

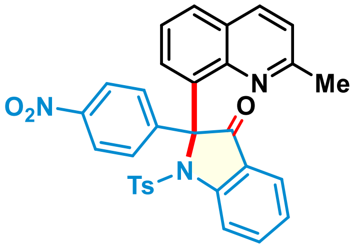

62, 46%

2-(2-methylquinolin-8-yl)-2-(4-nitrophenyl)-1-tosylindolin-3-one (62)

25.28 mg, 46% yield, yellow solid.

***Rf*:** 0.30 (hexane/ethyl acetate, 8:2 v/v).

^{1}H NMR (400 MHz, CDCl$_3$) δ 8.39 (d, *J* = 8.4 Hz, 1H), 8.32 – 8.10 (m, 3H), 7.80 – 7.65 (m, 5H), 7.51 (t, *J* = 7.8 Hz, 1H), 7.33 – 7.18 (m, 2H), 6.82 (d, *J* = 8.4 Hz, 1H), 6.58 (d, *J* = 8.3 Hz, 2H), 6.40 (d, *J* = 8.1 Hz, 2H), 2.04 (s, 3H), 1.68 (s, 3H).

^{13}C NMR (101 MHz, CDCl$_3$) δ 195.83, 157.13, 151.25, 147.91, 145.39, 144.30, 143.05, 136.19, 136.16, 136.09, 135.82, 135.43, 129.17, 128.06, 126.31, 125.19, 125.00, 124.78, 124.67, 123.76, 123.30, 121.68, 115.11, 79.17, 23.15, 21.23.

HRMS (ESI-TOF) m/z: [M+H]$^+$calcd for $C_{31}H_{24}N_3O_5S$ 550.1431; Found 550.1433.

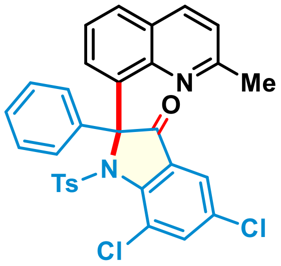

63, 40%

(5,7-dichloro-2-(2-methylquinolin-8-yl)-2-phenyl-1-tosylindolin-3-one (63)

22.9 mg, 40% yield, yellow solid.

***Rf*:** 0.50 (hexane/ethyl acetate, 9:1 v/v).

^{1}H NMR (500 MHz, CDCl$_3$) δ 8.09 (d, *J* = 7.5 Hz, 1H), 8.03 (d, *J* = 8.4 Hz, 1H), 7.96 (s, 2H), 7.82 (d, *J* = 8.0 Hz, 1H), 7.73 (d, *J* = 2.2 Hz, 1H), 7.66 (d, *J* = 2.2 Hz, 1H), 7.53 (t, *J* = 7.8 Hz, 1H), 7.46 (t, *J* = 7.6 Hz, 2H), 7.41 (t, *J* = 7.3 Hz, 1H), 7.16 (d, *J* = 8.4 Hz, 1H), 6.73 (d, *J* = 8.3 Hz, 2H), 6.65 (d, *J* = 8.2 Hz, 2H), 2.17 (s, 3H), 2.03 (s, 3H).

^{13}C NMR (126 MHz, CDCl$_3$) δ 193.61, 157.34, 145.87, 145.32, 142.86, 139.52, 137.97, 137.00, 136.76, 136.48, 135.78, 129.93, 129.13, 128.84, 128.74, 128.53, 128.40, 126.51, 125.74, 125.37, 123.24, 122.03, 119.98, 83.05, 23.63, 21.35.

HRMS (ESI-TOF) m/z: $[M+H]^+$calcd for $C_{31}H_{23}Cl_2N_2O_3S$ 573.0801; Found 573.0808.

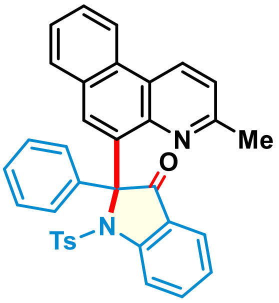

64, 54%

2-(3-methylbenzo[f]quinolin-5-yl)-2-phenyl-1-tosylindolin-3-one (64)

30.0 mg, 54% yield, white solid.

***Rf*:** 0.50 (hexane/ethyl acetate, 8:2 v/v).

^{1}H NMR (500 MHz, CDCl$_3$) δ 8.54 – 8.46 (m, 2H), 8.43 (d, *J* = 8.3 Hz, 1H), 8.21 (s, 1H), 8.08 - 7.94 (m, 3H), 7.76 (dd, *J* = 13.4, 7.0 Hz, 3H), 7.67 (t, *J* = 7.4 Hz, 1H), 7.46 (dd, *J* = 16.4, 7.0 Hz, 3H), 7.26 (t, *J* = 7.4 Hz, 1H), 6.97 (d, *J* = 8.4 Hz, 1H), 6.60 (d, *J* = 7.9 Hz, 2H), 6.22 (d, *J* = 7.8 Hz, 2H), 2.02 (s, 3H), 1.72 (s, 3H).

^{13}C NMR (126 MHz, CDCl$_3$) δ 196.78, 155.91, 151.48, 145.21, 142.55, 138.40, 136.63, 136.22, 135.58, 134.83, 130.69, 130.45, 130.22, 130.01, 128.36, 128.33, 128.24, 127.88, 127.23, 125.31, 125.05, 124.65, 123.30, 123.03, 121.81, 121.27, 114.99, 22.60, 21.23.

HRMS (ESI-TOF) m/z: $[M+H]^+$calcd for $C_{35}H_{27}N_3O_3S$ 555.1737; Found 555.1735.

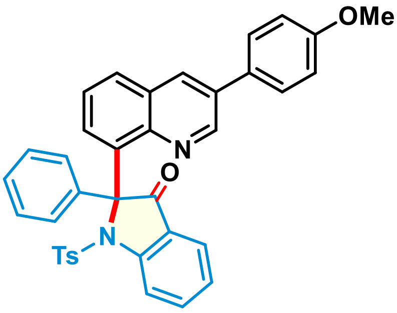

65, 52%

2-(3-(4-methoxyphenyl)quinolin-8-yl)-2-phenyl-1-tosylindolin-3-one (65)

31.0 mg, 52% yield, white solid.

***R_f*:** 0.40 (hexane/ethyl acetate, 8:2 v/v).

^{1}H NMR (500 MHz, CDCl$_3$) δ 8.41 (d, *J* = 8.4 Hz, 1H), 8.12 (d, *J* = 2.2 Hz, 1H), 8.07 – 7.91 (m, 2H), 7.88 (d, *J* = 7.3 Hz, 1H), 7.83 (d, *J* = 8.0 Hz, 1H), 7.80 – 7.72 (m, 2H), 7.61 (t, *J* =

7.8 Hz, 1H), 7.46 – 7.39 (m, 5H), 7.26 (t, *J* = 7.5 Hz, 1H), 7.00 (d, *J* = 8.7 Hz, 2H), 6.63 (d, *J* = 8.2 Hz, 2H), 6.43 (d, *J* = 8.2 Hz, 2H), 3.88 (s, 3H), 1.89 (s, 3H).

^{13}C NMR (126 MHz, CDCl$_3$) δ 197.00, 159.91, 151.32, 147.31, 144.97, 142.88, 136.77, 136.75, 136.39, 136.33, 135.89, 133.30, 131.58, 129.83, 129.16, 128.33, 128.26, 128.17, 127.96, 126.44, 125.23, 124.88, 124.71, 123.47, 114.91, 114.68, 79.57, 55.52, 21.30.

HRMS (ESI-TOF) m/z: [M+H]$^+$calcd for $C_{37}H_{29}N_2O_4S$ 597.1843; Found 597.1853.

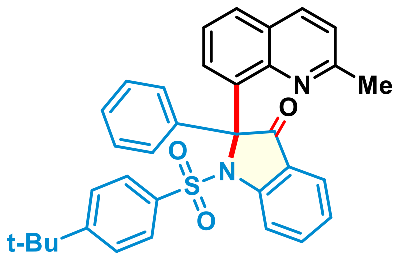

66, 64%

1-((4-(*tert*-butyl)phenyl)sulfonyl)-2-(2-methylquinolin-8-yl)-2-phenylindolin-3-one (66)

35.0 mg, 64% yield, white solid.

***R$_f$*:** 0.40 (hexane/ethyl acetate, 8:2 v/v).

^{1}H NMR (400 MHz, CDCl$_3$) δ 8.38 (d, *J* = 8.6 Hz, 1H), 8.02 – 7.86 (m, 3H), 7.72 (t, *J* = 8.0 Hz, 4H), 7.52 (t, *J* = 7.7 Hz, 1H), 7.44 - 7.35 (m, 3H), 7.21 (t, *J* = 7.4 Hz, 1H), 6.74 (d, *J* = 8.3 Hz, 1H), 6.64 (dd, *J* = 18.8, 8.5 Hz, 4H), 1.62 (s, 3H), 1.09 (s, 9H).

^{13}C NMR (101 MHz, CDCl$_3$) δ 197.04, 156.61, 155.42, 151.20, 145.67, 136.45, 136.37, 136.35, 136.32, 135.71, 135.55, 128.59, 128.23, 128.20, 126.13, 125.19, 125.16, 125.10, 124.58, 124.40, 123.25, 121.86, 114.98, 79.47, 34.80, 30.95, 23.16

HRMS (ESI-TOF) m/z: [M+Na]$^+$calcd for $C_{34}H_{30}N_2NaO_3S$ 569.1869; Found 569.1877.

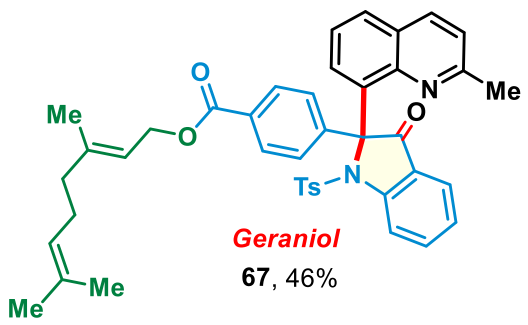

67, 46%

(*E*)-3,7-dimethylocta-2,6-dien-1-yl-4-(2-(2-methylquinolin-8-yl)-3-oxo-1-tosylindolin-2-yl)benzoate (67)

31.5 mg, 46% yield, brown sticky liquid.

***Rf*:** 0.40 (hexane/ethyl acetate, 8:2 v/v).

^{1}H NMR (500 MHz, CDCl$_3$) δ 8.41 (d, *J* = 8.3 Hz, 1H), 8.08 (dd, *J* = 39.1, 10.7 Hz, 4H), 7.79 (d, *J* = 7.3 Hz, 1H), 7.78 – 7.71 (m, 4H), 7.51 (t, *J* = 7.7 Hz, 1H), 7.25 (t, *J* = 7.4 Hz, 1H), 6.82 (d, *J* = 8.4 Hz, 1H), 6.60 (d, *J* = 8.2 Hz, 2H), 6.41 (d, *J* = 8.1 Hz, 2H), 5.49 (t, *J* = 6.6 Hz, 1H), 5.13 (t, *J* = 6.6 Hz, 1H), 4.87 (d, *J* = 7.0 Hz, 2H), 2.19 – 2.13 (m, 2H), 2.12 – 2.07 (m, 2H), 2.05 (s, 3H), 1.79 (s, 3H), 1.70 (d, *J* = 8.2 Hz, 6H), 1.64 (s, 3H).

^{13}C NMR (126 MHz, CDCl$_3$) δ 196.32, 166.53, 156.88, 151.25, 145.51, 142.77, 142.59, 141.60, 136.45, 136.29, 135.81, 135.78, 135.72, 131.95, 130.41, 129.37, 128.87, 127.98, 127.33, 126.23, 125.14, 124.98, 124.58, 123.91, 123.46, 121.45, 118.48, 115.02, 79.41, 61.94, 39.66, 26.44, 25.81, 23.16, 21.19, 17.84, 16.70

HRMS (ESI-TOF) m/z: $[M+Na]^+$calcd for $C_{42}H_{41}N_2O_5S$ 685.2731; Found 685.2735.

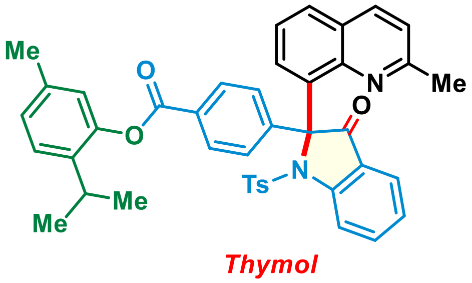

68, 49%

2-isopropyl-5-methylphenyl-4-(2-(2-methylquinolin-8-yl)-3-oxo-1-tosylindolin-2-yl)benzoate (68)

33.4 mg, 49% yield, white solid.

***Rf*:** 0.40 (hexane/ethyl acetate, 8:2 v/v).

^{1}H NMR (400 MHz, CDCl$_3$) δ 8.41 (d, *J* = 8.7 Hz, 1H), 8.25 (dt, *J* = 23.4, 8.6 Hz, 3H), 7.83 (d, *J* = 7.2 Hz, 1H), 7.76 (dd, *J* = 20.3, 8.3 Hz, 4H), 7.53 (t, *J* = 7.7 Hz, 1H), 7.26 (t, *J* = 6.6 Hz, 3H), 7.07 (d, *J* = 7.9 Hz, 1H), 6.95 (s, 1H), 6.82 (d, *J* = 8.4 Hz, 1H), 6.60 (d, *J* = 8.0 Hz, 2H), 6.41 (d, *J* = 8.0 Hz, 2H), 3.06 (dd, *J* = 12.9, 6.1 Hz, 1H), 2.35 (s, 3H), 2.04 (s, 3H), 1.69 (s, 3H), 1.23 – 1.20 (m, 6H).

^{13}C NMR (101 MHz, CDCl$_3$) δ 196.32, 165.24, 156.97, 151.31, 148.26, 145.52, 142.85, 142.53, 137.38, 136.74, 136.43, 136.31, 135.94, 135.77, 135.71, 130.88, 130.03, 129.50, 128.97, 128.02, 127.27, 126.58, 126.37, 126.28, 125.20, 125.01, 124.65, 123.55, 123.00, 121.53, 115.06, 79.48, 27.36, 23.25, 23.19, 21.23, 21.02.

HRMS (ESI-TOF) m/z: $[M+H]^+$calcd for $C_{42}H_{37}N_2O_5S$ 681.2418; Found 681.2437.

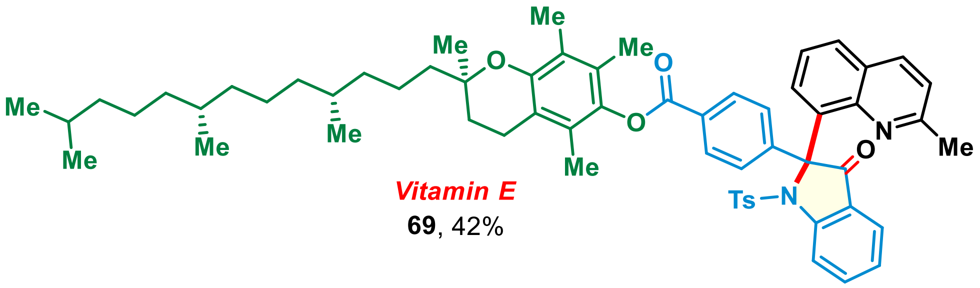

Vitamin E

69, 42%

(*R*)-2,5,7,8-tetramethyl-2-((4*R*,8*R*)-4,8,12-trimethyltridecyl)chroman-6-yl-4-(-2-(2-methylquinolin-8-yl)-3-oxo-1-tosylindolin-2-yl)benzoate (69)

40.4 mg, 42% yield, brown sticky liquid.

***R_f*: ** 0.40 (hexane/ethyl acetate, 8:2 v/v).

^{1}H NMR (500 MHz, CDCl$_3$) δ 8.43 (d, *J* = 8.7 Hz, 1H), 8.32 (d, *J* = 6.0 Hz, 2H), 7.87 (t, *J* = 6.5 Hz, 1H), 7.81 – 7.73 (m, 5H), 7.56 (t, *J* = 7.7 Hz, 1H), 7.31 – 7.25 (m, 2H), 6.84 (d, *J* = 8.4 Hz, 1H), 6.63 (d, *J* = 8.2 Hz, 2H), 6.43 (d, *J* = 8.1 Hz, 2H), 2.65 (t, *J* = 6.0 Hz, 2H), 2.44 – 2.37 (m, 1H), 2.15 (s, 3H), 2.10 – 2.04 (m, 9H), 1.88 – 1.80 (m, 2H), 1.72 (s, 3H), 1.55 (dd, *J* = 13.2, 6.6 Hz, 2H), 1.31 – 1.27 (m, 10H), 1.16 (dd, *J* = 22.9, 16.3 Hz, 8H), 0.89 (t, *J* = 7.6 Hz, 15H).

^{13}C NMR (126 MHz, CDCl$_3$) δ 196.35, 165.05, 156.95, 151.33, 149.58, 145.54, 142.82, 142.34, 140.79, 136.47, 136.35, 135.92, 135.77, 135.75, 130.01, 129.54, 128.96, 128.02, 127.42, 127.11, 126.28, 125.33, 125.20, 125.01, 124.97, 124.66, 123.54, 123.24, 121.52, 117.60, 115.05, 79.50, 75.20, 39.51, 37.68, 37.60, 37.53, 37.43, 32.93, 32.92, 32.87, 28.12, 24.95, 24.59, 23.20, 22.87, 22.77, 21.23, 21.19, 20.77, 19.90, 19.83, 19.74, 13.21, 12.36, 11.99.

HRMS (ESI-TOF) m/z: $[M+H]^+$calcd for $C_{61}H_{73}N_2O_6S$ 977.5184; Found 961.5177.

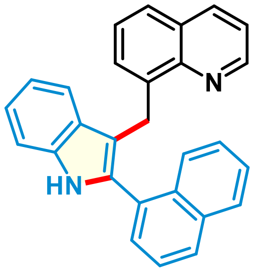

72, 92%

8-((2-(naphthalen-1-yl)-1*H*-indol-3-yl)methyl)quinoline (72)

35.3 mg, 92% yield, white solid.

***R_f*: ** 0.50 (hexane/ethyl acetate, 9:1 v/v).

^{1}H NMR (500 MHz, CDCl$_3$) δ 8.90 (d, J = 2.7 Hz, 1H), 8.30 (s, 1H), 8.13 (d, J = 8.0 Hz, 1H), 7.98 (d, J = 8.4 Hz, 1H), 7.89 (t, J = 8.7 Hz, 2H), 7.65 – 7.56 (m, 2H), 7.54 – 7.42 (m, 5H), 7.42 – 7.35 (m, 2H), 7.33 – 7.25 (m, 2H), 7.09 (t, J = 7.5 Hz, 1H), 4.82 (s, 2H).

^{13}C NMR (126 MHz, CDCl$_3$) δ 149.19, 146.81, 139.72, 136.41, 136.29, 134.99, 133.82, 132.81, 130.64, 129.01, 128.92, 128.84, 128.72, 128.39, 128.23, 126.63, 126.50, 126.14, 125.77, 125.36, 122.26, 120.88, 120.20, 119.68, 113.23, 110.83, 25.78.

HRMS (ESI-TOF) m/z: [M+H]$^+$calcd for $C_{28}H_{21}N_2$ 385.1699; Found 385.1715.

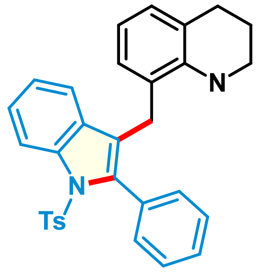

73, 71%

8-((2-phenyl-1-tosyl-1H-indol-3-yl)methyl)-1,2,3,4-tetrahydroquinoline (73)

35.0 mg, 71% yield, white solid.

***R$_f$*:** 0.50 (hexane/ethyl acetate, 9:1 v/v).

^{1}H NMR (500 MHz, CDCl$_3$) δ 8.39 (d, J = 8.4 Hz, 1H), 7.48 – 7.33 (m, 6H), 7.29 (d, J = 7.8 Hz, 2H), 7.22 (d, J = 3.9 Hz, 2H), 7.10 (d, J = 8.2 Hz, 2H), 6.84 (d, J = 7.4 Hz, 1H), 6.31 (t, J = 7.4 Hz, 1H), 6.01 (d, J = 7.5 Hz, 1H), 3.55 (s, 2H), 3.34 – 3.24 (m, 2H), 2.80 (t, J = 6.3 Hz, 2H), 2.38 (s, 3H), 1.97 – 1.88 (m, 2H)

^{13}C NMR (126 MHz, CDCl$_3$) δ 144.62, 138.59, 138.22, 134.46, 132.01, 131.26, 131.05, 130.96, 129.34, 129.30, 128.75, 127.73, 127.64, 127.10, 127.01, 125.93, 125.28, 124.61, 122.20, 120.10, 117.16, 42.37, 27.41, 25.91, 21.79, 21.72.

HRMS (ESI-TOF) m/z: [M+H]$^+$calcd for $C_{31}H_{28}N_2O_2S$ 493.1953; Found 493.1952.

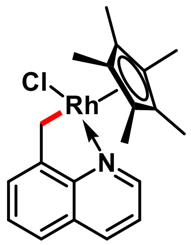

70

^{1}H NMR (400 MHz, CDCl$_3$) δ 8.88 (d, *J* = 4.0 Hz, 1H), 8.02 (dd, *J* = 8.3, 1.4 Hz, 1H), 7.56 (dd, *J* = 6.4, 1.6 Hz, 1H), 7.44 – 7.34 (m, 2H), 7.29 (dd, *J* = 8.3, 4.9 Hz, 1H), 3.91 (d, *J* = 13.3 Hz, 1H), 3.63 (d, *J* = 13.3 Hz, 1H), 1.56 (s, 15H).

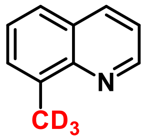

8-(methyl-d3)quinoline

168.4 mg, 96% yield, colourless liquid.

***R_f*:** 0.40 (hexane/ethyl acetate, 9:1 v/v).

^{1}H NMR (500 MHz, CDCl$_3$) δ 8.98 (d, *J* = 2.6 Hz, 1H), 8.17 (dd, *J* = 8.2, 1.3 Hz, 1H), 7.70 (d, *J* = 8.1 Hz, 1H), 7.60 (d, *J* = 6.7 Hz, 1H), 7.47 (t, *J* = 7.6 Hz, 1H), 7.43 (dd, *J* = 8.2, 4.2 Hz, 1H), 2.82 (s, 0.15H).

8. References

1. (a) Brand, J. P.; Chevalley, C.; Waser, J. *Beilstein J. Org. Chem.* **2011,** *7,* 565–569. (b) Boominathan, S. S. K.; Senadi, G. C.; Vandavasi, J. K.; Chen, J. Y.-F.; Wang, J-J. *Chem. Eur. J.* **2015**, *21*, 3193–3197.
2. Pramanick, P. K.; Zhou, Z.; Hou, Z.; Ao, Y.; Yao, B. *Chin. Chem. Lett.* **2020**, *31*, 1327-1331.
3. Bajwa, J. S.; Chen, G.-P.; Prasad, K.; Repic, O.; Blacklock, T. J. *Tetrahedron. Lett.* **2006**, *47*, 6425–6427.
4. Liu, B.; Zhou, T.; Li, B.; Xu, S.; Song, H.; Wang, B. *Angew. Chem. Int. Ed.* **2014**, *53*, 4191–4195.
5. Wang, H.-W.; Wu, J.-X.; Qiao, Y.-H.; Li, Y.-F.; Li, D.-C.; J.-M. D.; Yao, Q.-X.; Lu, Y. *Org. Lett.* **2021**, *23*, 7177−7182.

9. [1] H and ^{13}C NMR Spectra of the Compounds

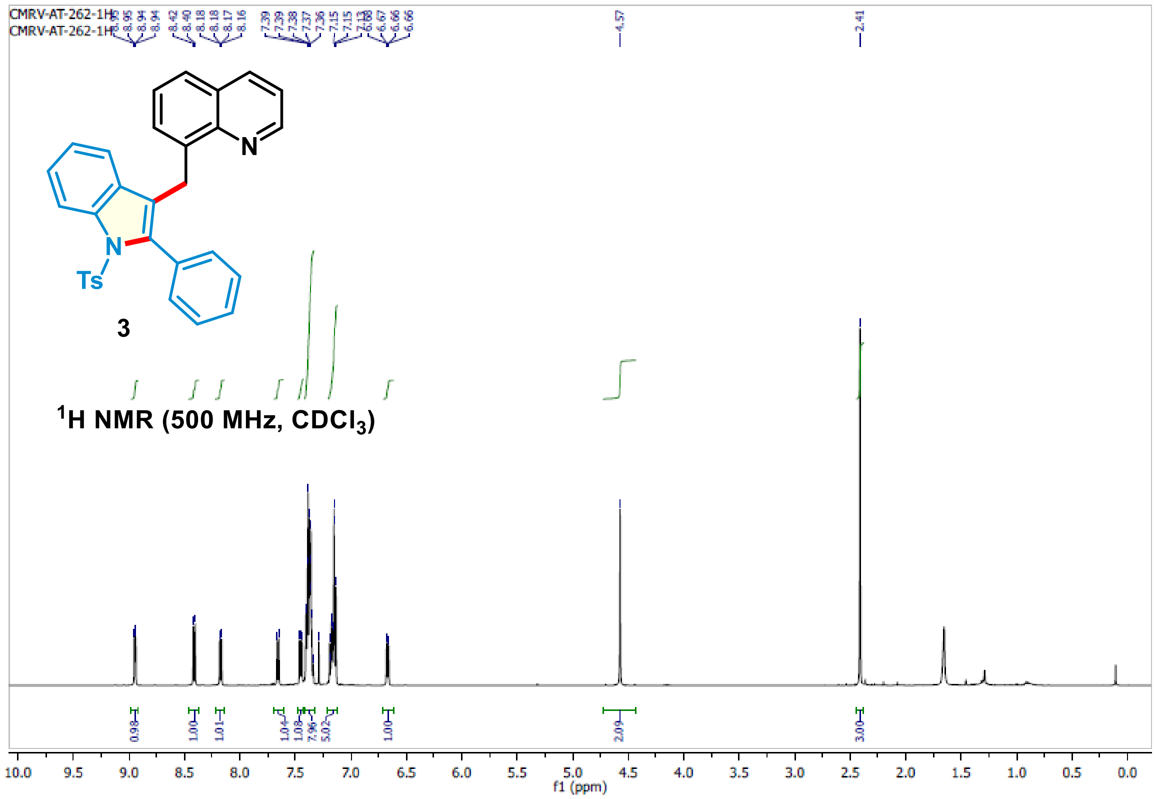

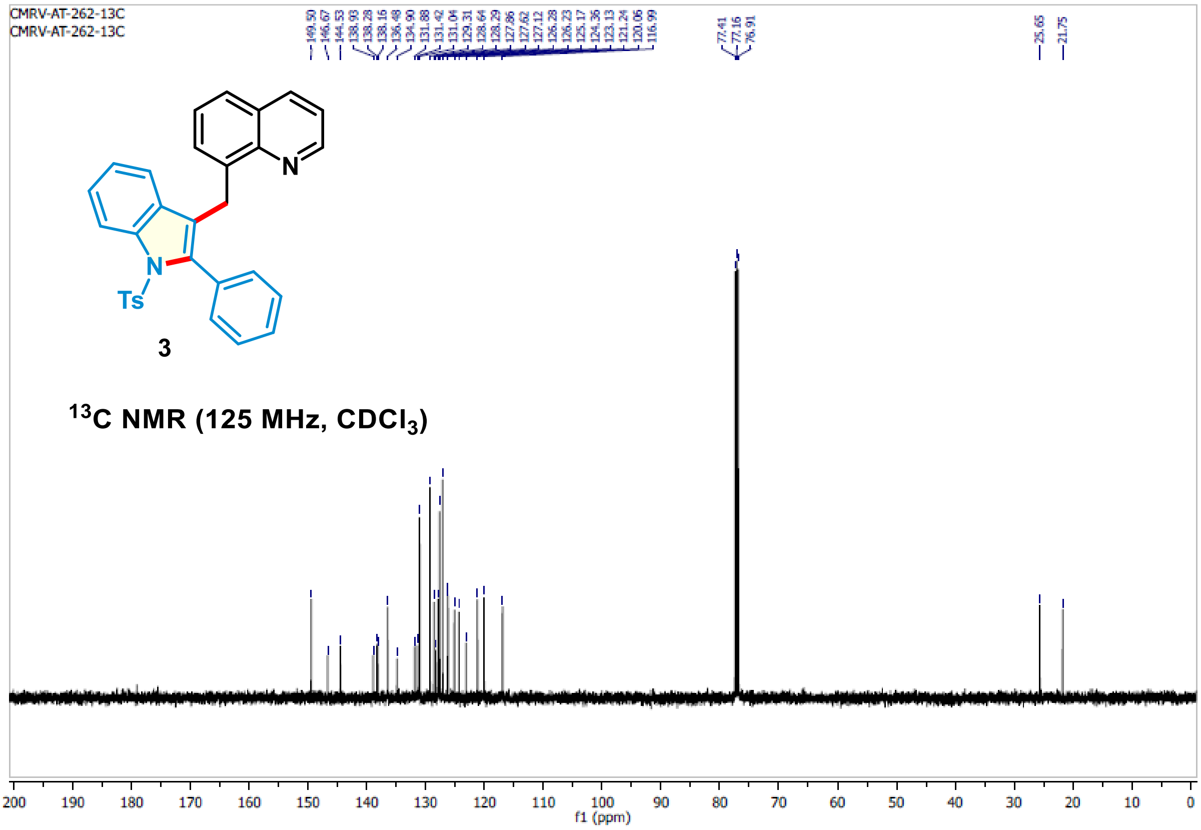

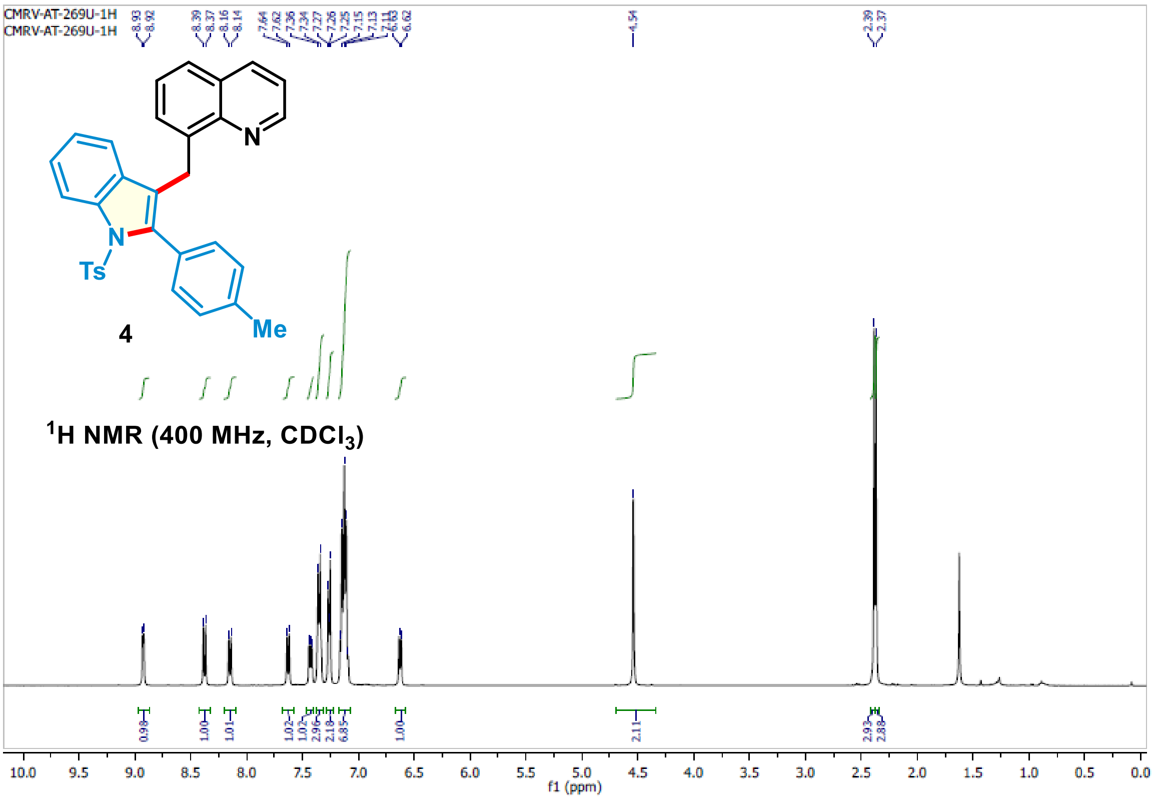
CMRV-AT-269U-1H
4
Ts
Me
N
^{1}H NMR (400 MHz, CDCl$_3$)
f1 (ppm)

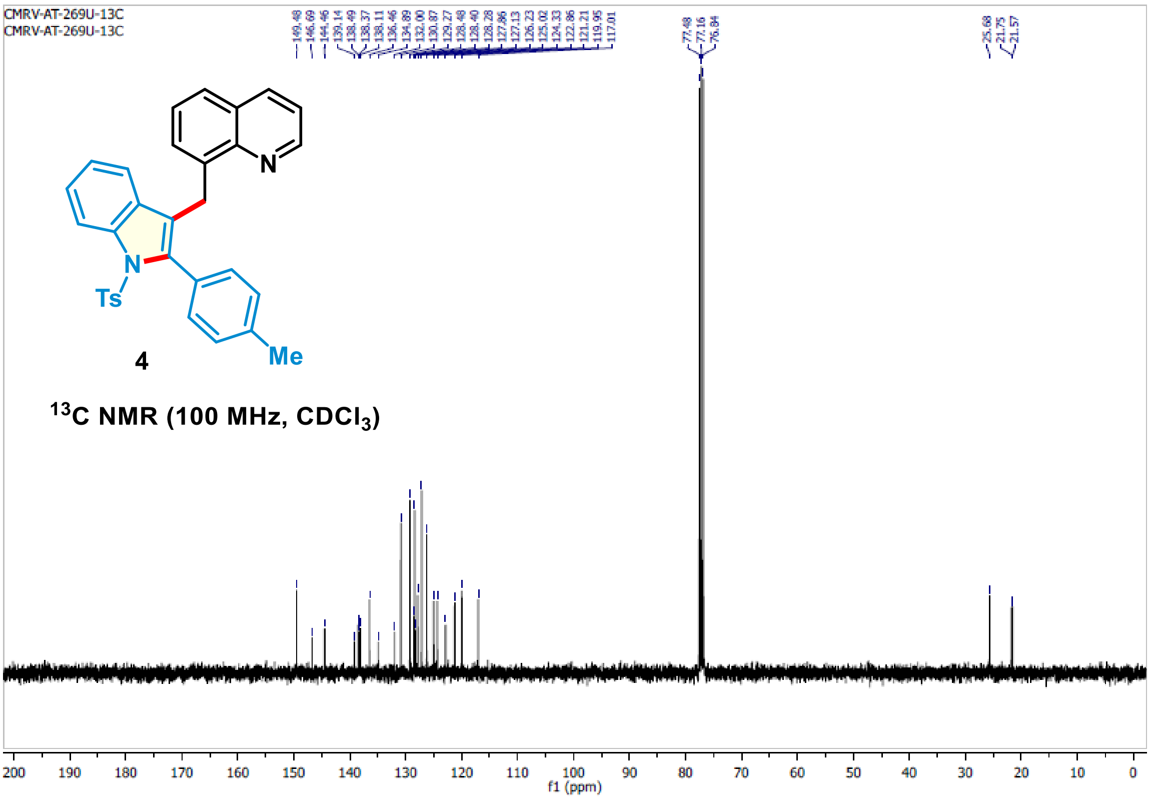
CMRV-AT-269U-13C
4
Ts
Me
N
^{13}C NMR (100 MHz, CDCl$_3$)
f1 (ppm)

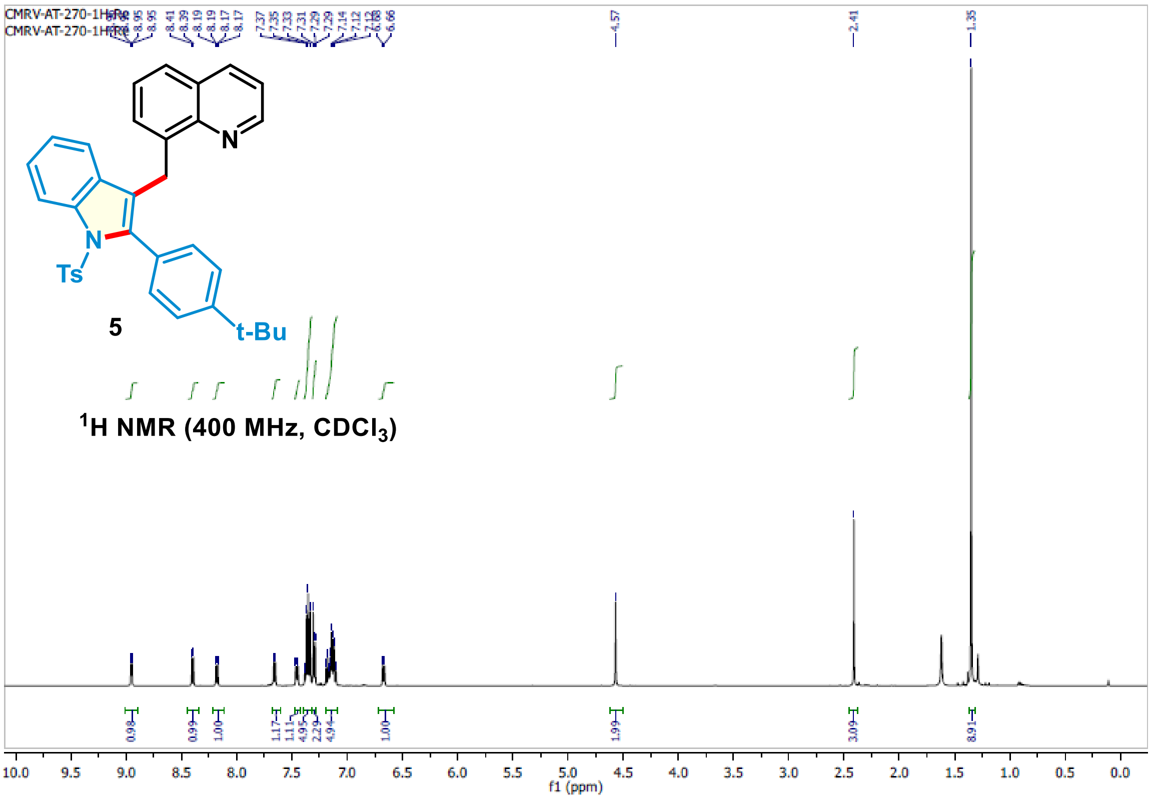
CMRV-AT-270-1H-Re
N
Ts
5
t-Bu
1H NMR (400 MHz, CDCl3)
4.57
2.41
1.35
f1 (ppm)

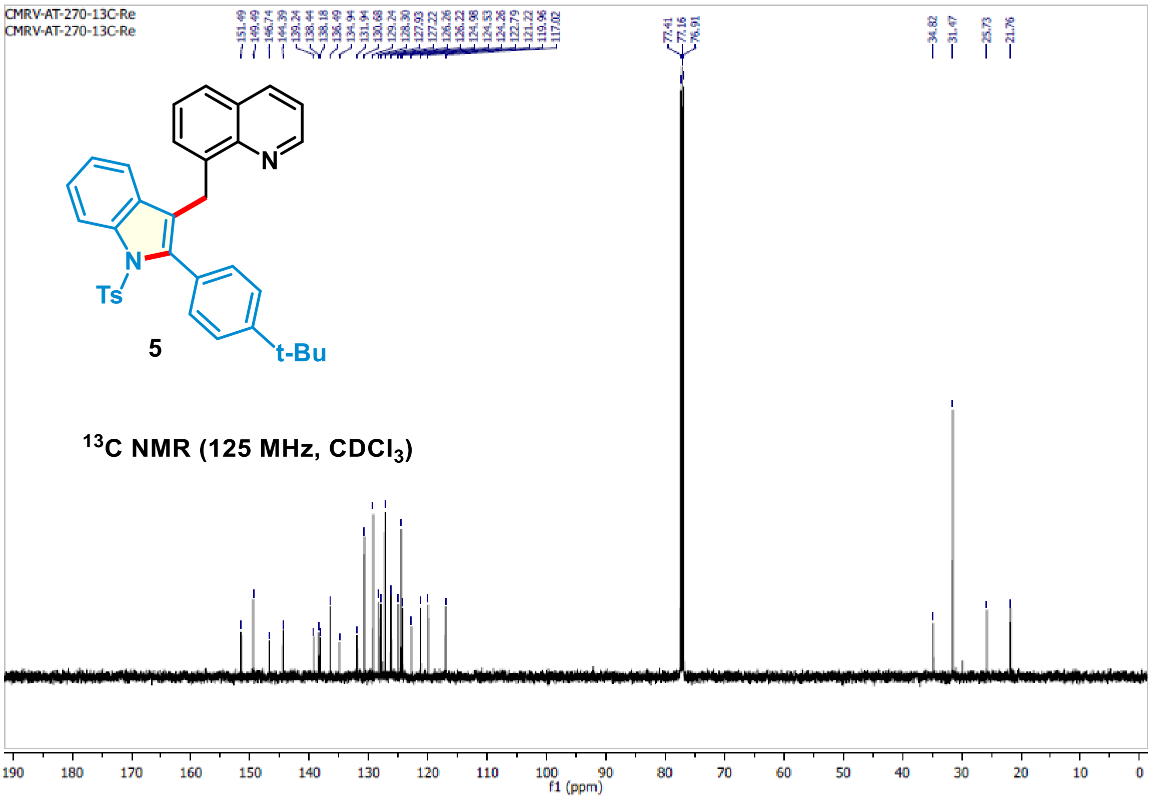
CMRV-AT-270-13C-Re
CMRV-AT-270-13C-Re
151.49
149.49
146.74
144.39
139.24
138.44
138.18
136.49
134.94
131.94
130.68
129.24
128.30
127.93
127.22
126.26
126.22
124.98
124.53
124.26
122.79
121.22
119.96
117.02
77.41
77.16
76.91
34.82
31.47
25.73
21.76
N
Ts
5
t-Bu
13C NMR (125 MHz, CDCl3)
f1 (ppm)

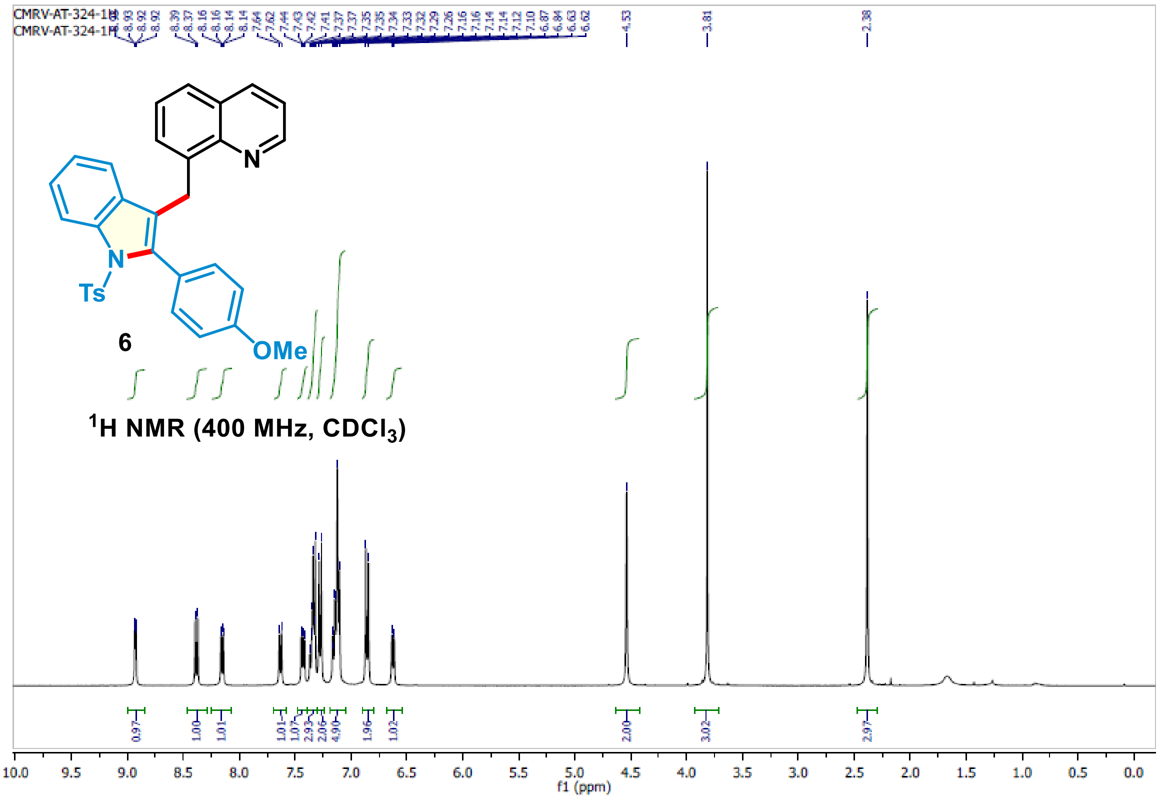
CMRV-AT-324-1H
N
N
Ts
OMe
6
1H NMR (400 MHz, CDCl3)
f1 (ppm)

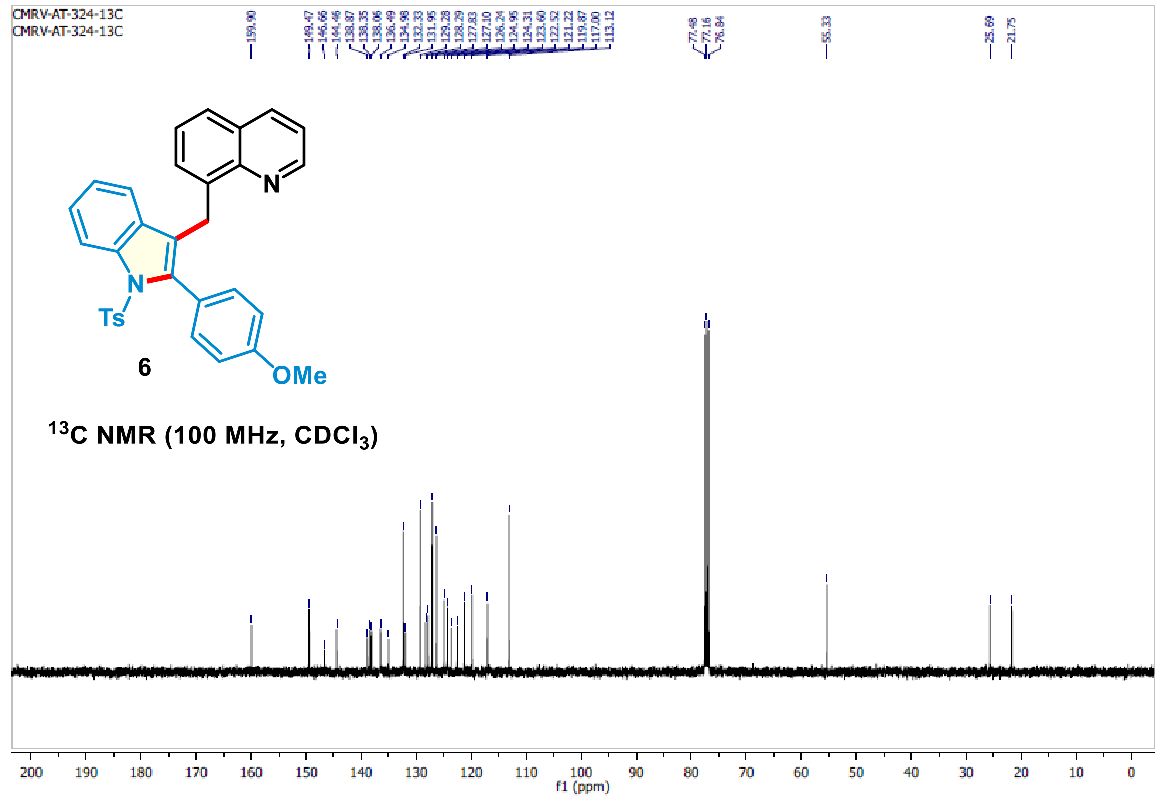
CMRV-AT-324-13C
N
N
Ts
OMe
6
13C NMR (100 MHz, CDCl3)
f1 (ppm)

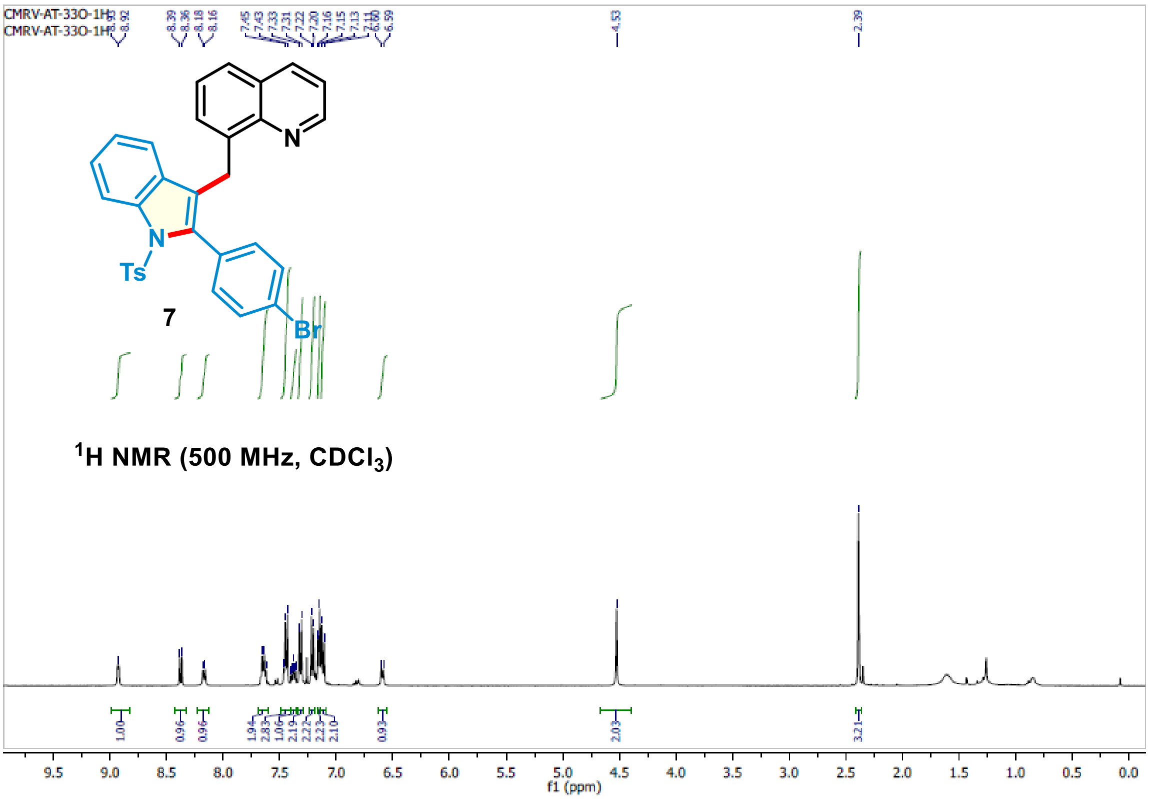
CMRV-AT-330-1H
CMRV-AT-330-1H
8.93
8.92
8.39
8.36
8.18
8.16
7.45
7.43
7.33
7.31
7.22
7.20
7.16
7.15
7.13
7.11
6.60
6.59
4.53
2.39
N
N
Ts
Br
7
1H NMR (500 MHz, CDCl3)
1.00
0.96
0.96
1.94
2.83
1.06
2.19
2.22
2.23
2.10
0.93
2.03
3.21
f1 (ppm)

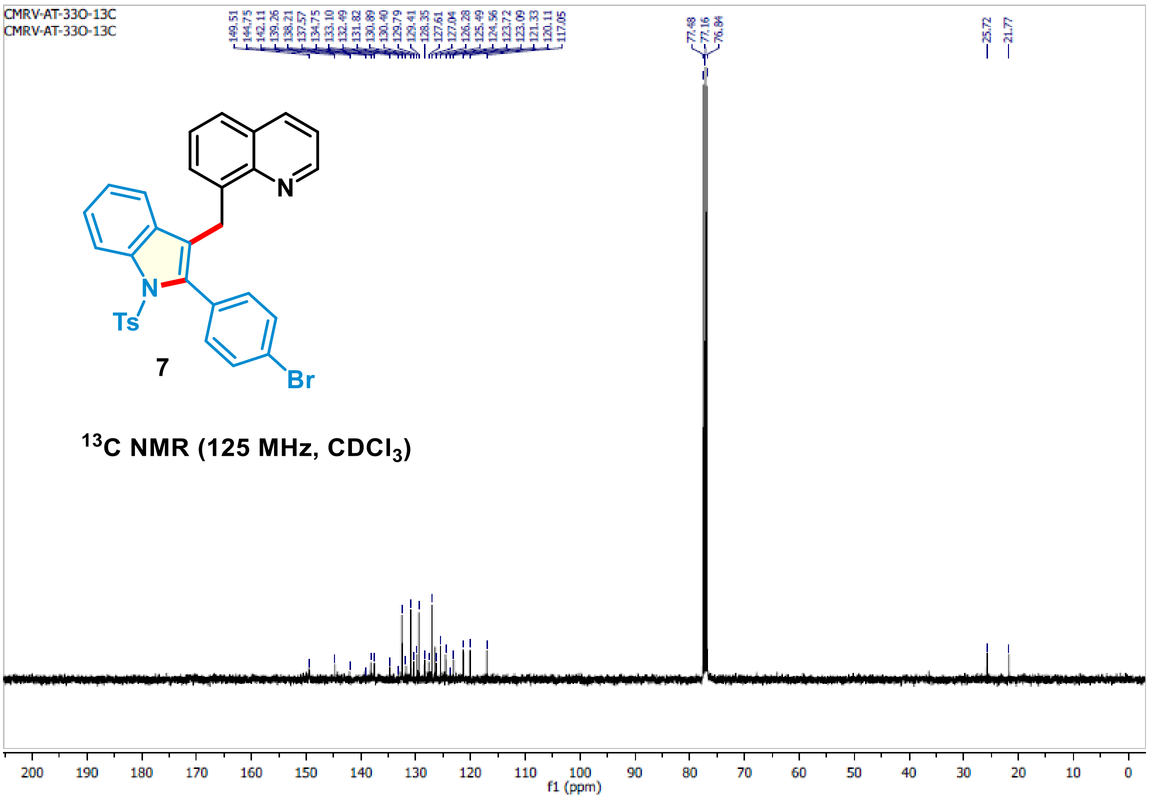
CMRV-AT-330-13C
CMRV-AT-330-13C
149.51
144.75
142.11
139.26
138.21
137.57
134.75
133.10
132.49
131.82
130.89
130.40
129.79
129.41
128.35
127.61
127.04
126.28
125.49
124.56
123.72
123.09
121.33
120.11
117.05
77.48
77.16
76.84
25.72
21.77
N
N
Ts
Br
7
13C NMR (125 MHz, CDCl3)
f1 (ppm)

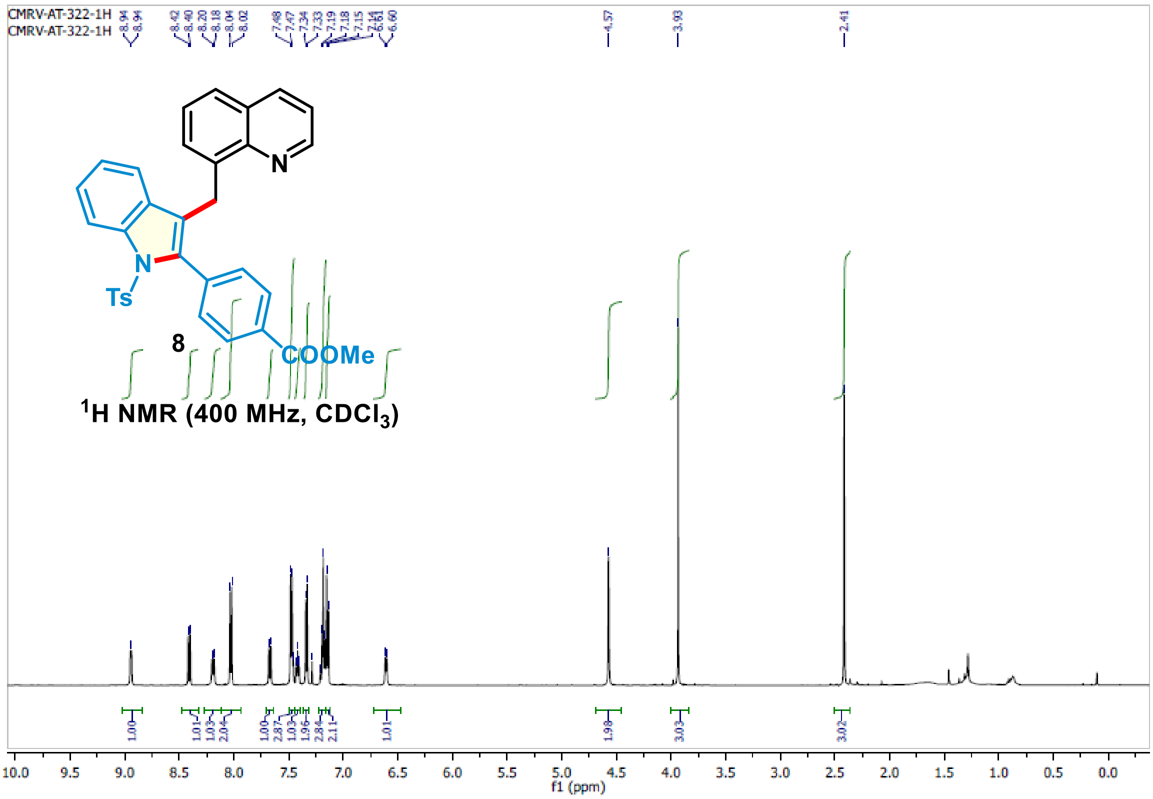
CMRV-AT-322-1H
8
COOMe
Ts
N
¹H NMR (400 MHz, CDCl₃)
f1 (ppm)

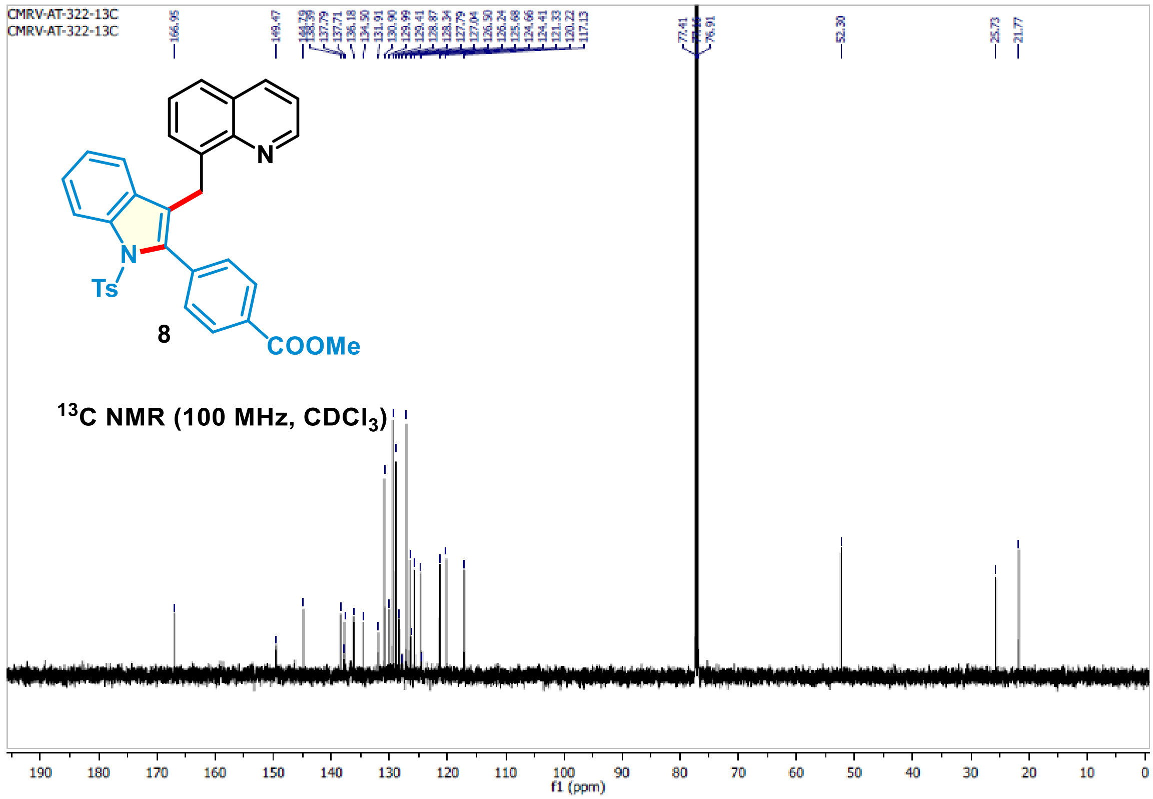
CMRV-AT-322-13C
8
COOMe
Ts
N
¹³C NMR (100 MHz, CDCl₃)
f1 (ppm)

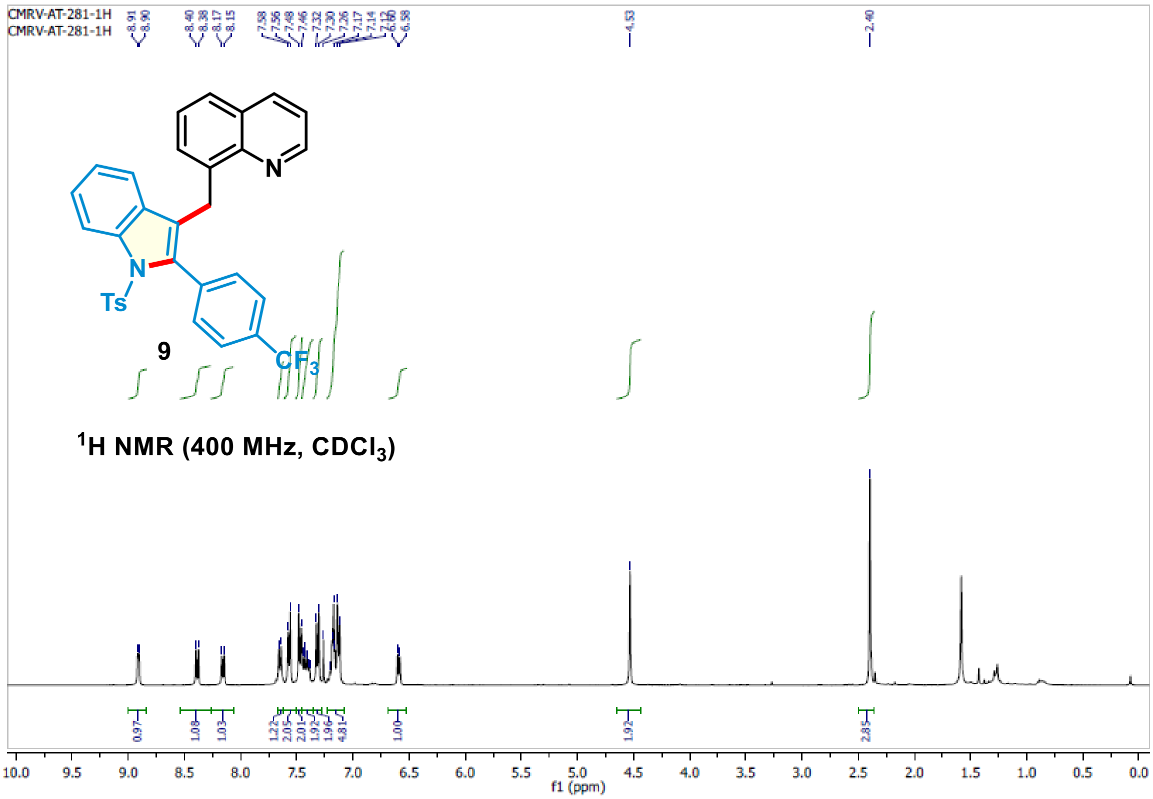
CMRV-AT-281-1H
CMRV-AT-281-1H
8.91
8.90
8.40
8.38
8.17
8.15
7.58
7.56
7.48
7.46
7.32
7.30
7.26
7.17
7.14
7.12
6.60
6.58
4.53
2.40
N
N
Ts
9
CF3
1H NMR (400 MHz, CDCl3)
0.97
1.08
1.03
1.22
2.05
2.01
1.92
1.96
4.81
1.00
1.92
2.85
f1 (ppm)

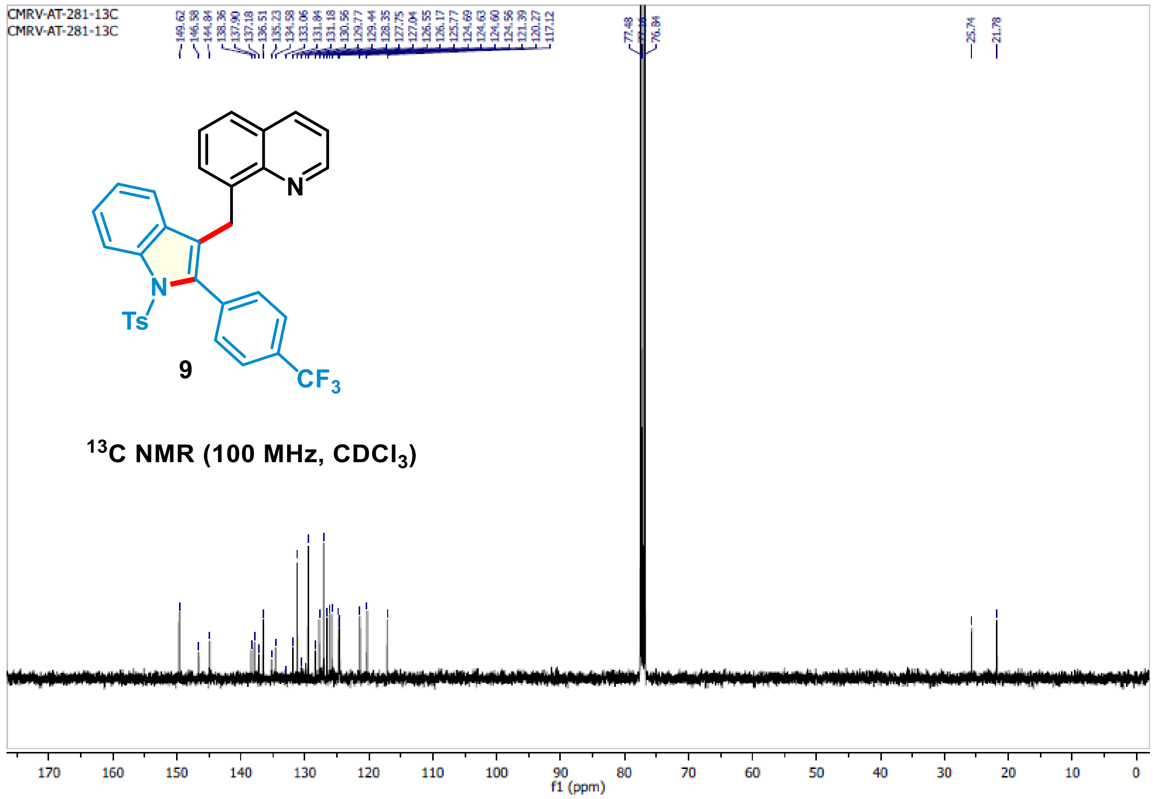
CMRV-AT-281-13C
CMRV-AT-281-13C
149.62
146.58
144.84
138.36
137.90
137.18
136.51
135.23
134.58
133.06
131.84
131.18
130.56
129.77
129.44
128.35
127.75
127.04
126.55
126.17
125.77
124.69
124.63
124.60
124.56
121.39
120.27
117.12
77.48
76.84
25.74
21.78
N
N
Ts
9
CF3
13C NMR (100 MHz, CDCl3)
f1 (ppm)

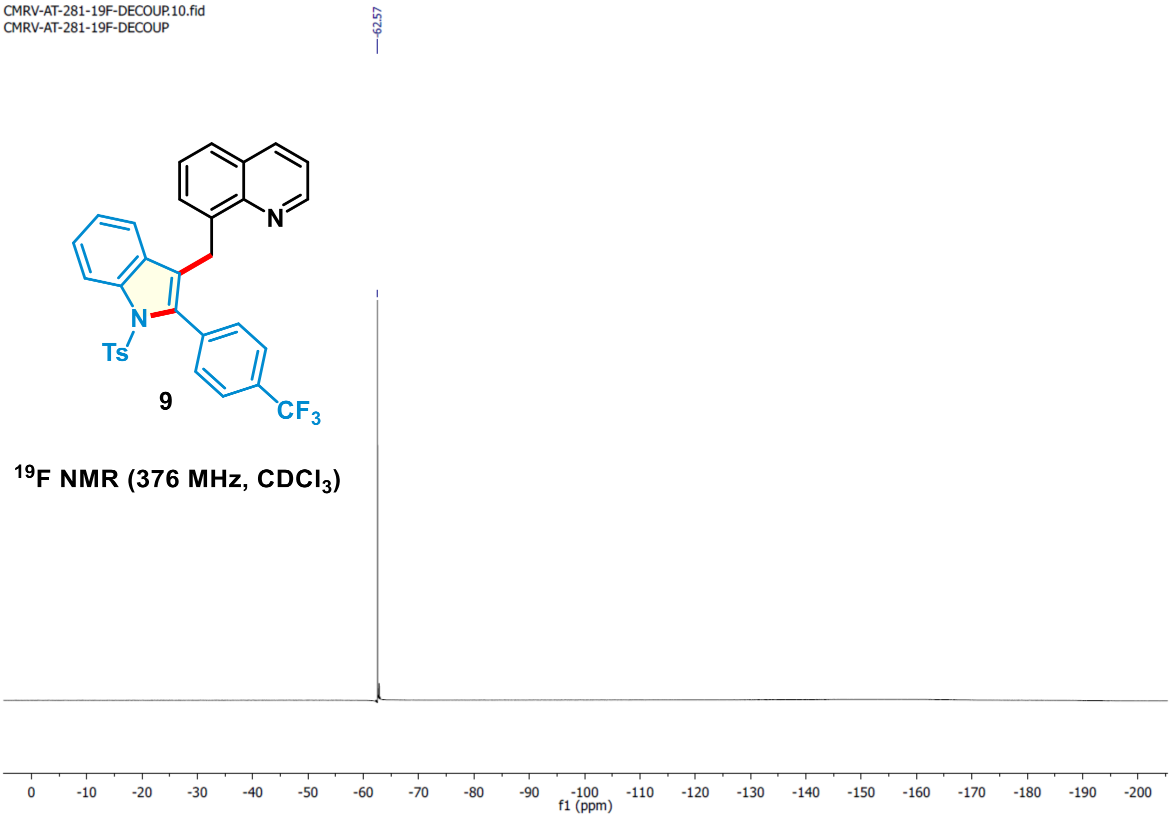
CMRV-AT-281-19F-DECOUP.10.fid
CMRV-AT-281-19F-DECOUP
-62.57
9
^{19}F NMR (376 MHz, $CDCl_3$)
f1 (ppm)

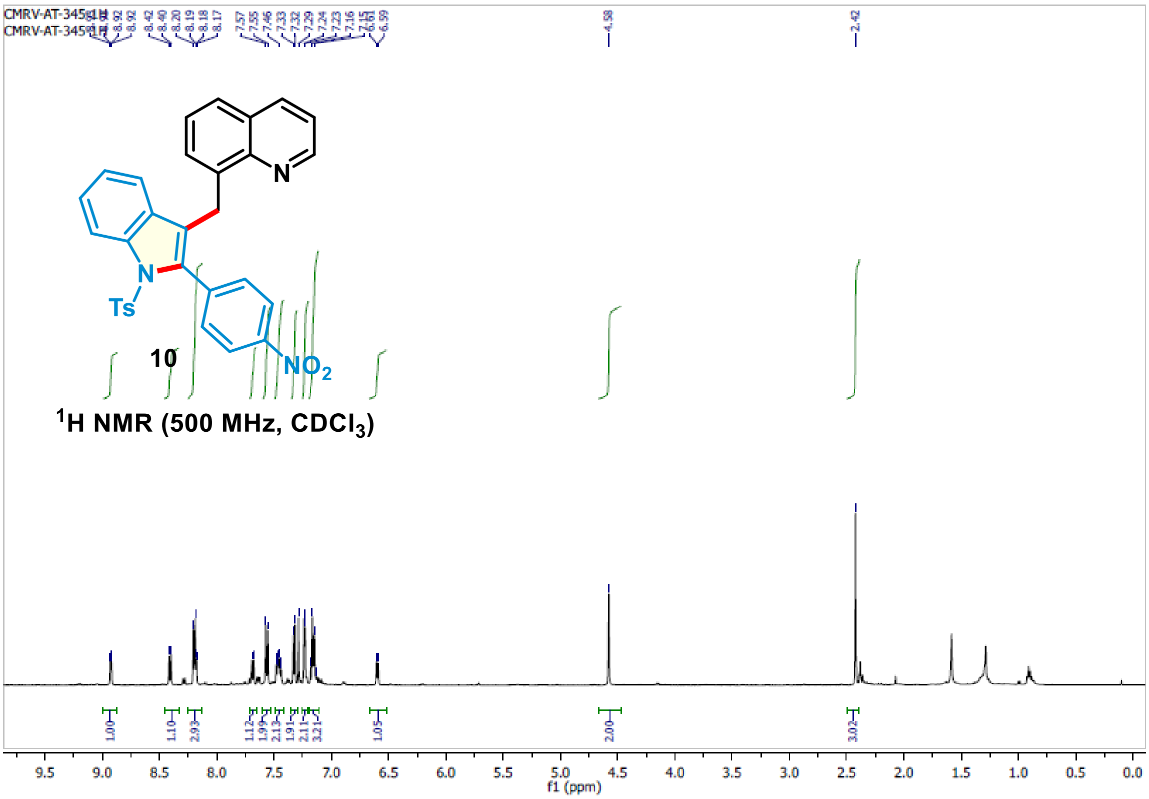
10
^{1}H NMR (500 MHz, $CDCl_3$)
4.58
2.42
1.00
1.10
2.93
1.05
2.00
3.02
f1 (ppm)

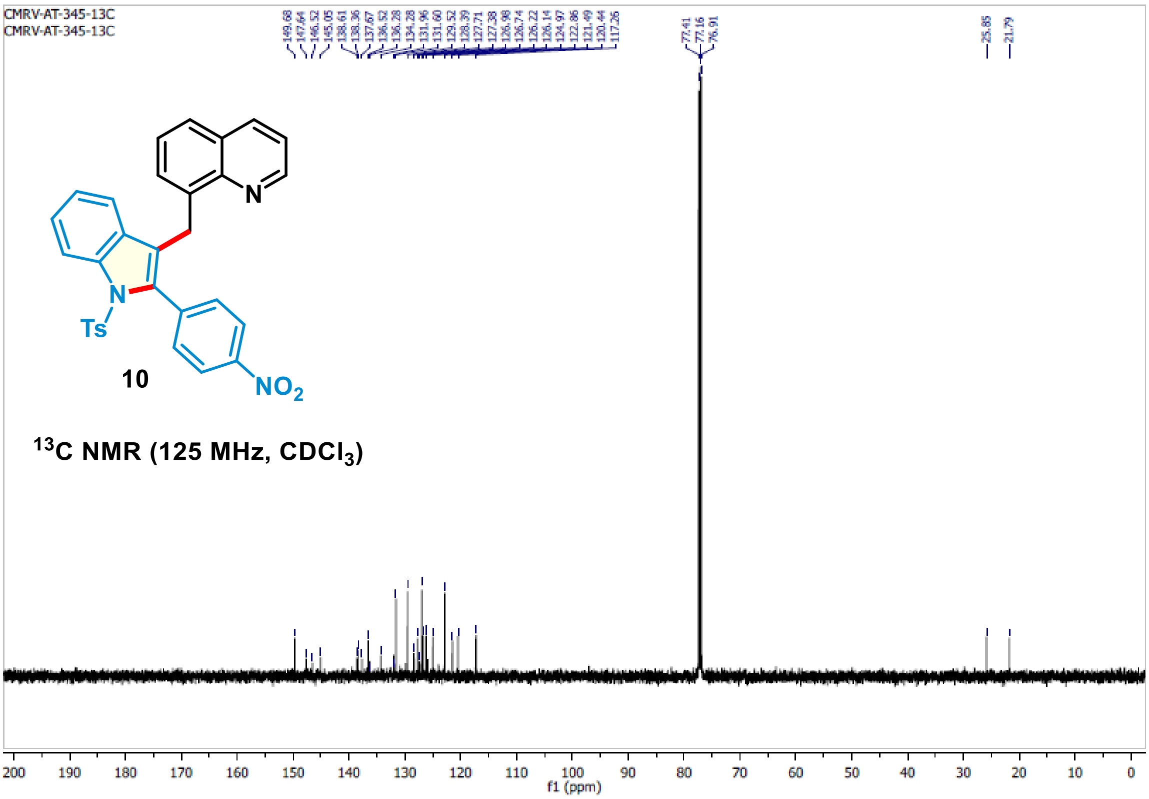

10

^{13}C NMR (125 MHz, $CDCl_3$)

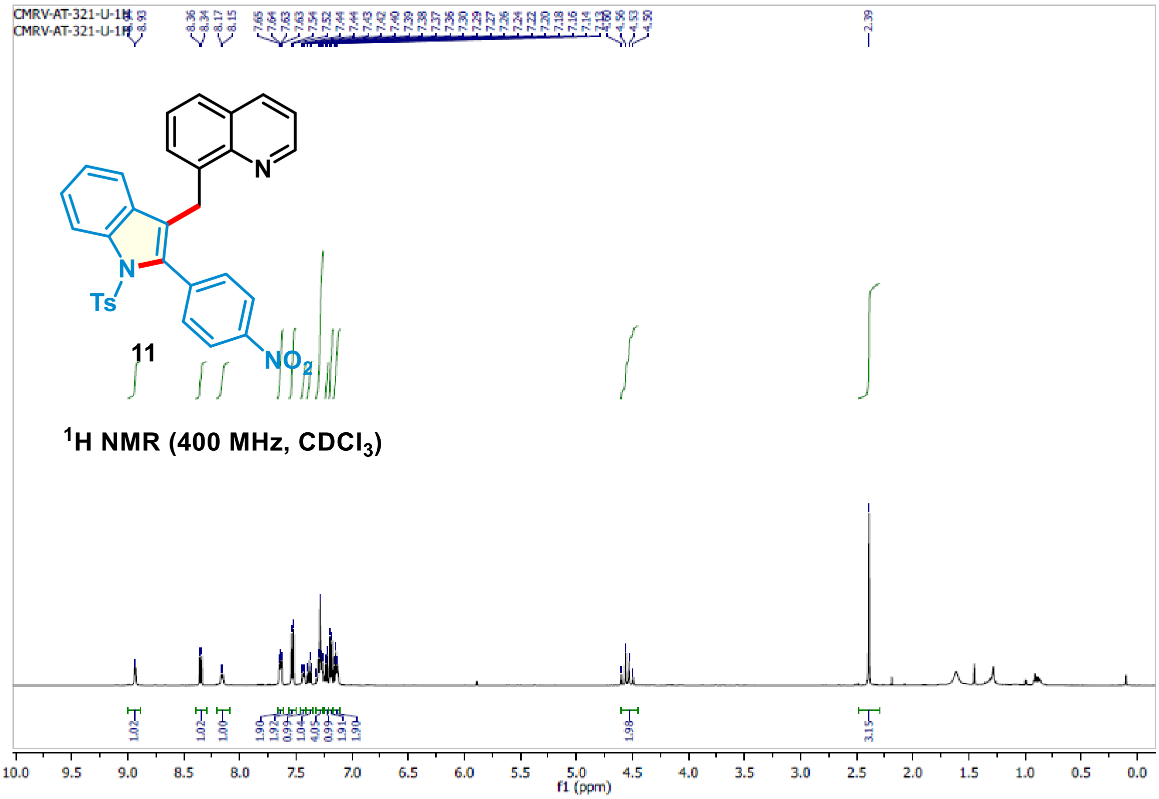

11

^{1}H NMR (400 MHz, $CDCl_3$)

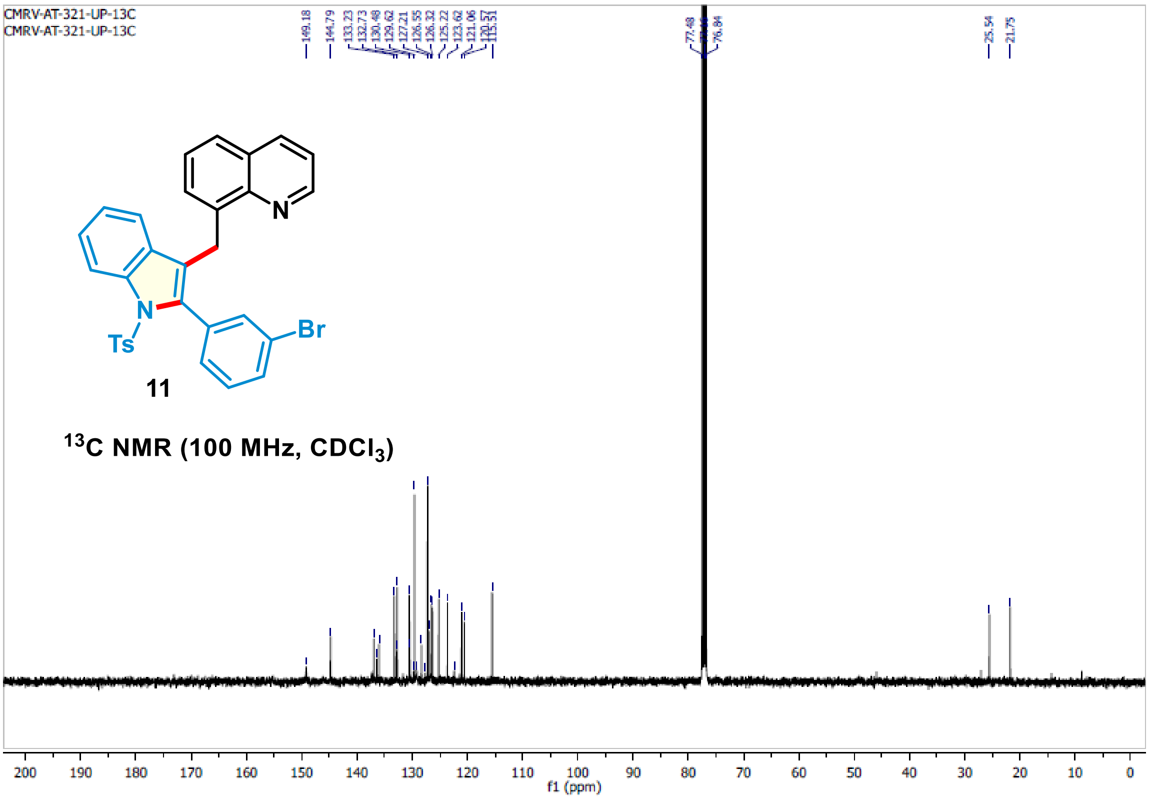
CMRV-AT-321-UP-13C
CMRV-AT-321-UP-13C
149.18
144.79
133.23
132.73
130.48
129.62
127.21
126.55
126.32
125.22
123.62
121.06
120.57
115.51
77.48
76.84
25.54
21.75
N
N
Ts
Br
11
13C NMR (100 MHz, CDCl3)
f1 (ppm)

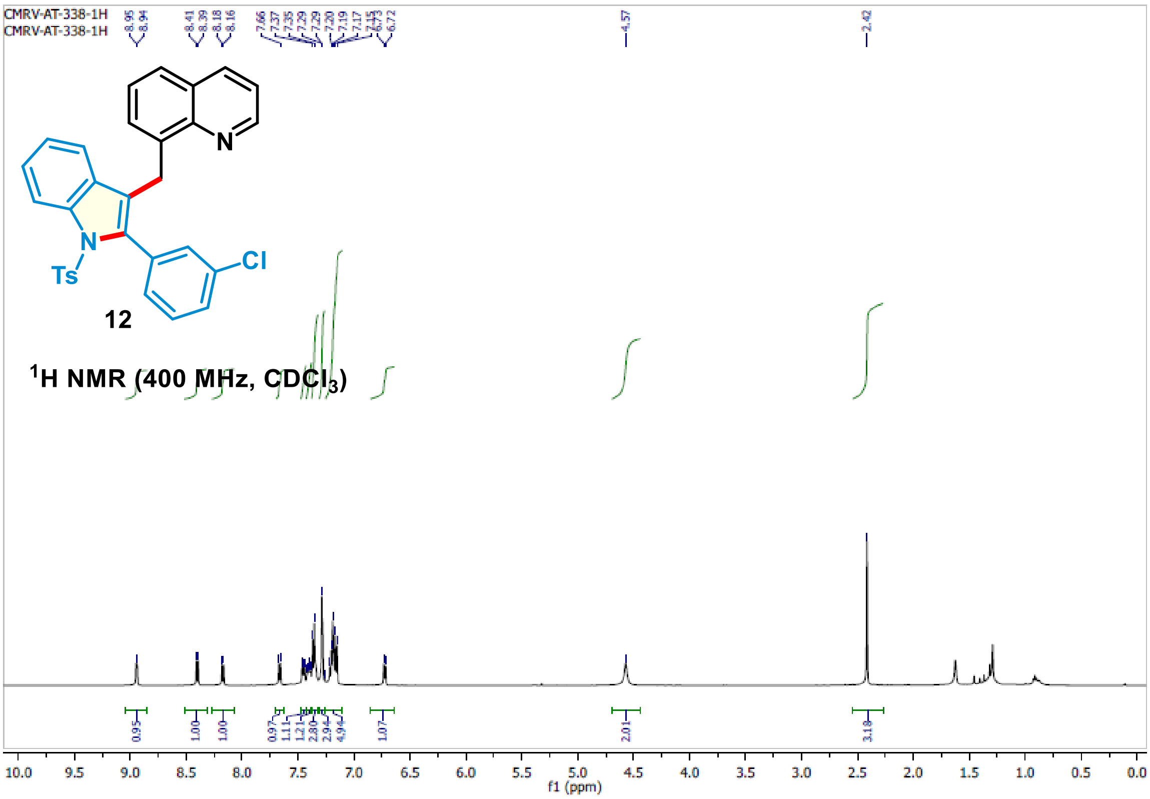
CMRV-AT-338-1H
CMRV-AT-338-1H
8.95
8.94
8.41
8.39
8.18
8.16
7.66
7.37
7.35
7.29
7.29
7.20
7.19
7.17
7.15
6.73
6.72
4.57
2.42
N
N
Ts
Cl
12
1H NMR (400 MHz, CDCl3)
0.95
1.00
1.00
0.97
1.11
1.21
2.80
2.94
4.94
1.07
2.01
3.18
f1 (ppm)

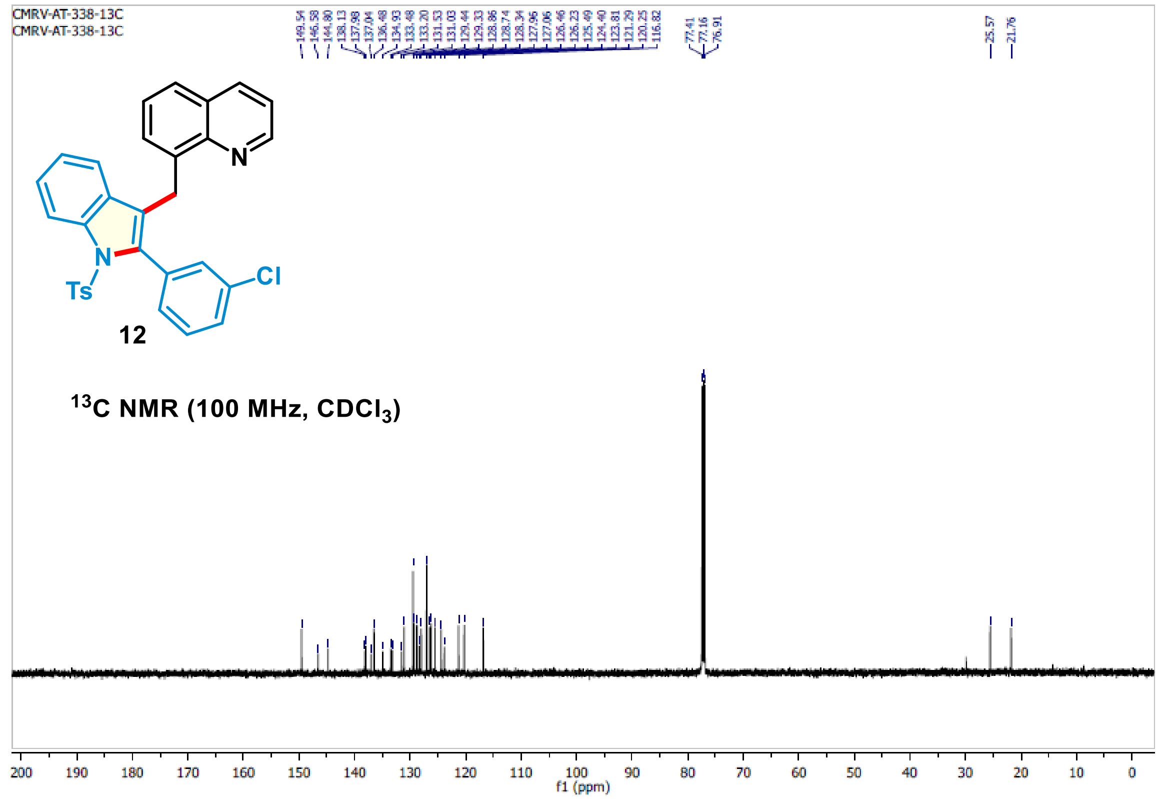
CMRV-AT-338-13C
CMRV-AT-338-13C
149.54 146.58 144.80 138.13 137.98 137.04 136.48 134.93 133.48 133.20 131.53 131.03 129.44 129.33 128.86 128.74 128.34 127.96 127.06 126.46 126.23 125.49 124.40 123.81 121.29 120.25 116.82
77.41 77.16 76.91
25.57
21.76
N
Ts
N
Cl
12
^{13}C NMR (100 MHz, CDCl$_3$)
f1 (ppm)

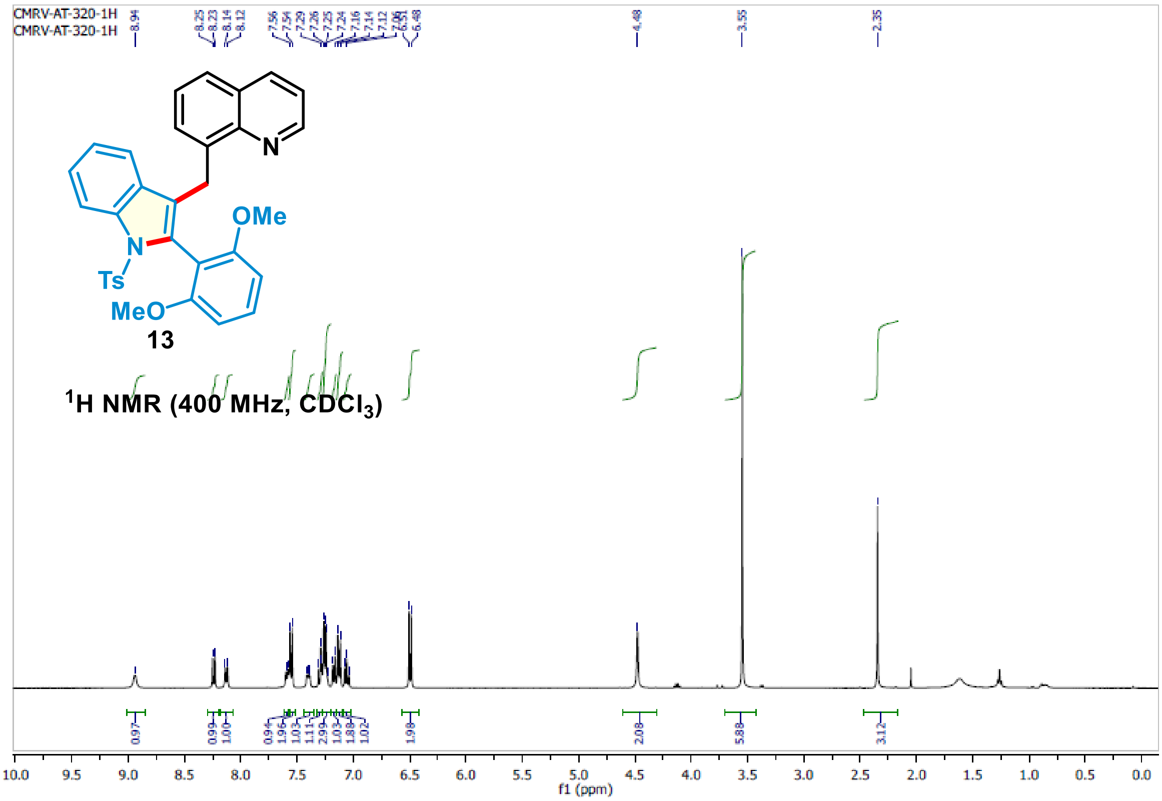
CMRV-AT-320-1H
CMRV-AT-320-1H
8.94
8.25 8.23 8.14 8.12
7.56 7.54 7.29 7.26 7.25 7.24 7.16 7.14 7.12
6.51 6.48
4.48
3.55
2.35
N
OMe
N
Ts
MeO
13
^{1}H NMR (400 MHz, CDCl$_3$)
0.97 0.99 1.00 0.94 1.96 1.03 1.11 2.99 1.03 1.88 1.02 1.98 2.08 5.88 3.12
f1 (ppm)

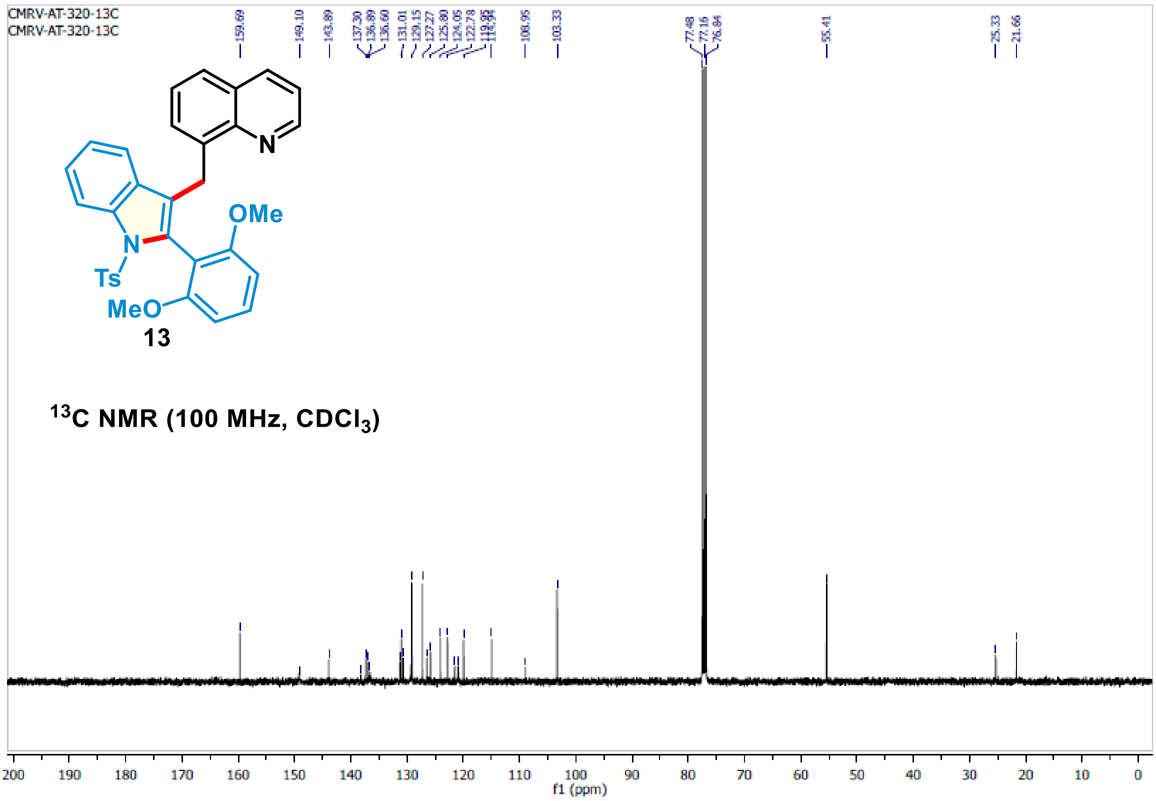
CMRV-AT-320-13C
CMRV-AT-320-13C
OMe
N
Ts
MeO
13
^{13}C NMR (100 MHz, $CDCl_3$)
f1 (ppm)

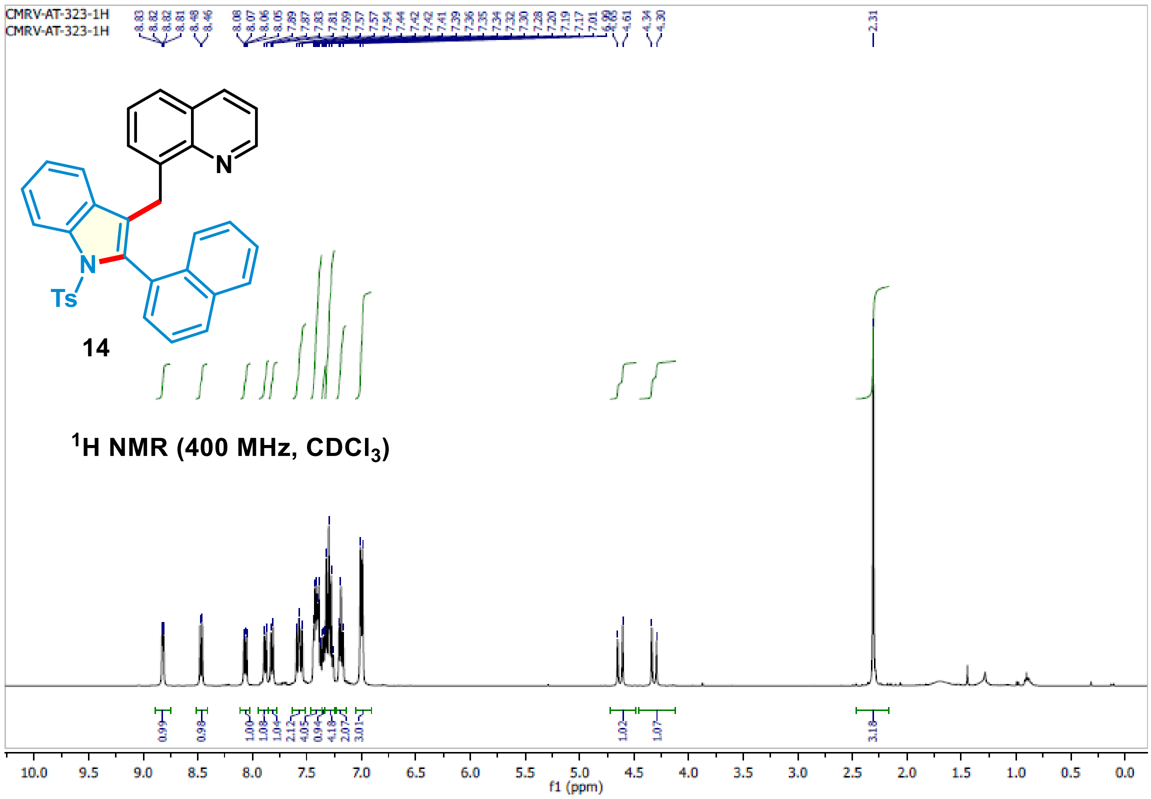
CMRV-AT-323-1H
CMRV-AT-323-1H
N
N
Ts
14
^{1}H NMR (400 MHz, $CDCl_3$)
f1 (ppm)

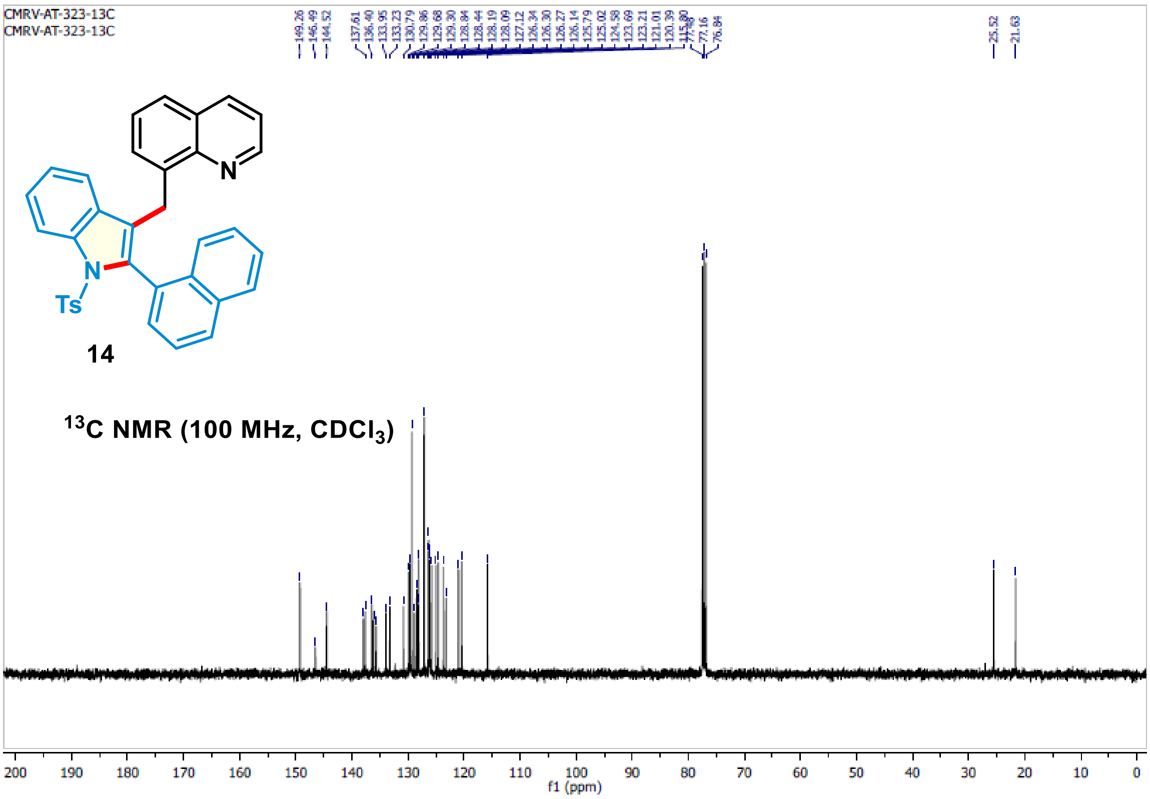
CMRV-AT-323-13C
CMRV-AT-323-13C
14
^{13}C NMR (100 MHz, CDCl$_3$)
f1 (ppm)

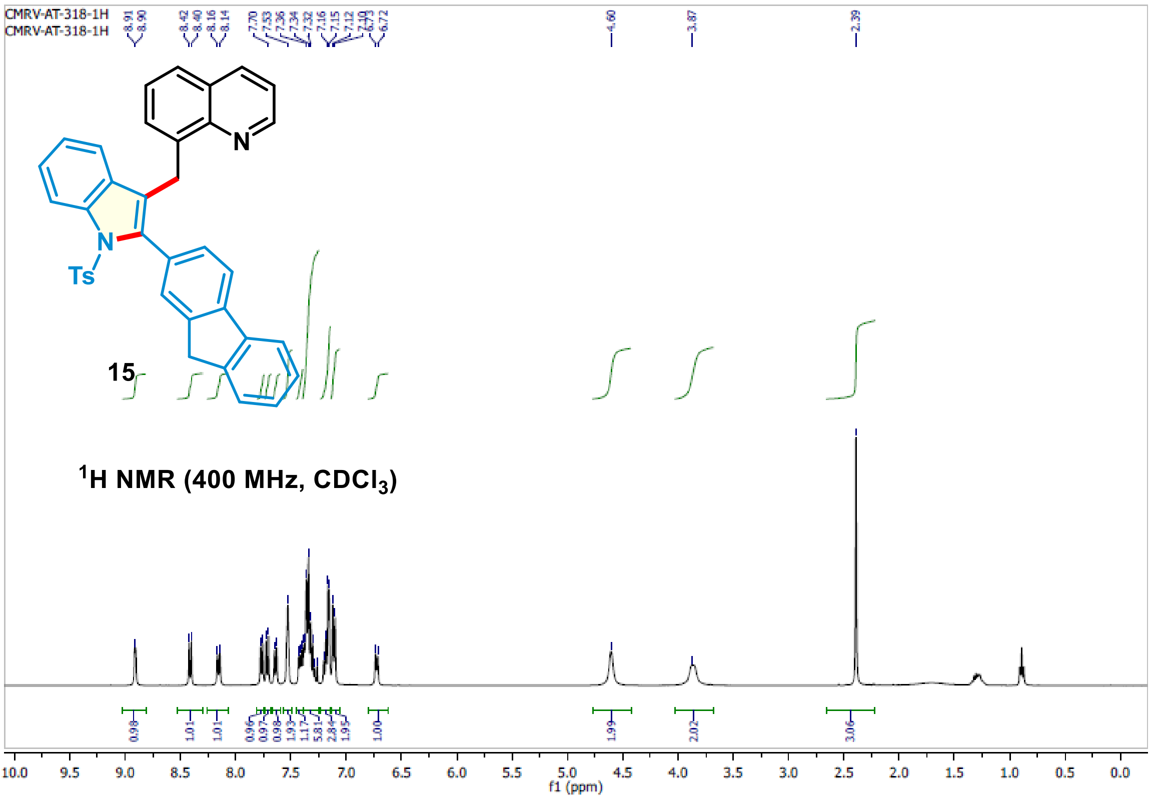
CMRV-AT-318-1H
CMRV-AT-318-1H
15
^{1}H NMR (400 MHz, CDCl$_3$)
f1 (ppm)

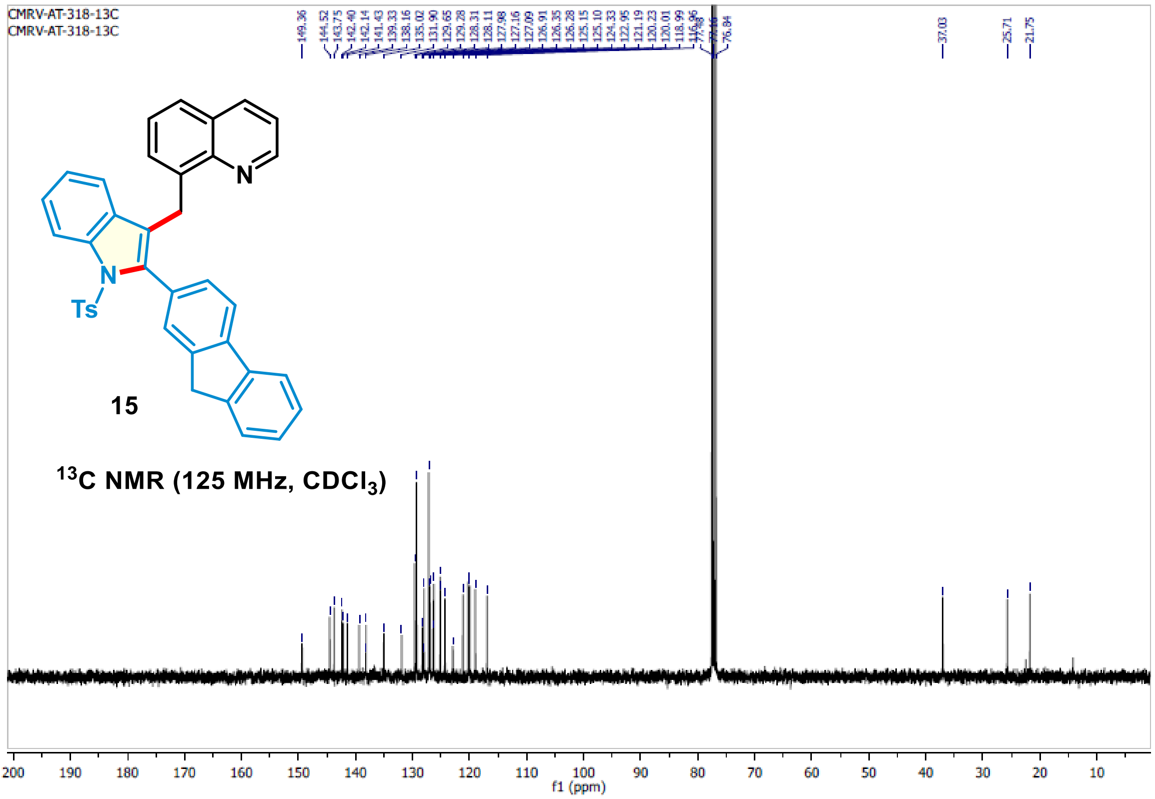
CMRV-AT-318-13C
CMRV-AT-318-13C
149.36
144.52
143.75
142.40
142.14
141.43
139.33
138.16
135.02
131.90
129.65
129.28
128.31
128.11
127.98
127.16
127.09
126.91
126.35
126.28
125.15
125.10
124.33
122.95
121.19
120.23
120.01
118.99
76.84
37.03
25.71
21.75
N
Ts
15
13C NMR (125 MHz, CDCl3)
200 190 180 170 160 150 140 130 120 110 100 90 80 70 60 50 40 30 20 10
f1 (ppm)

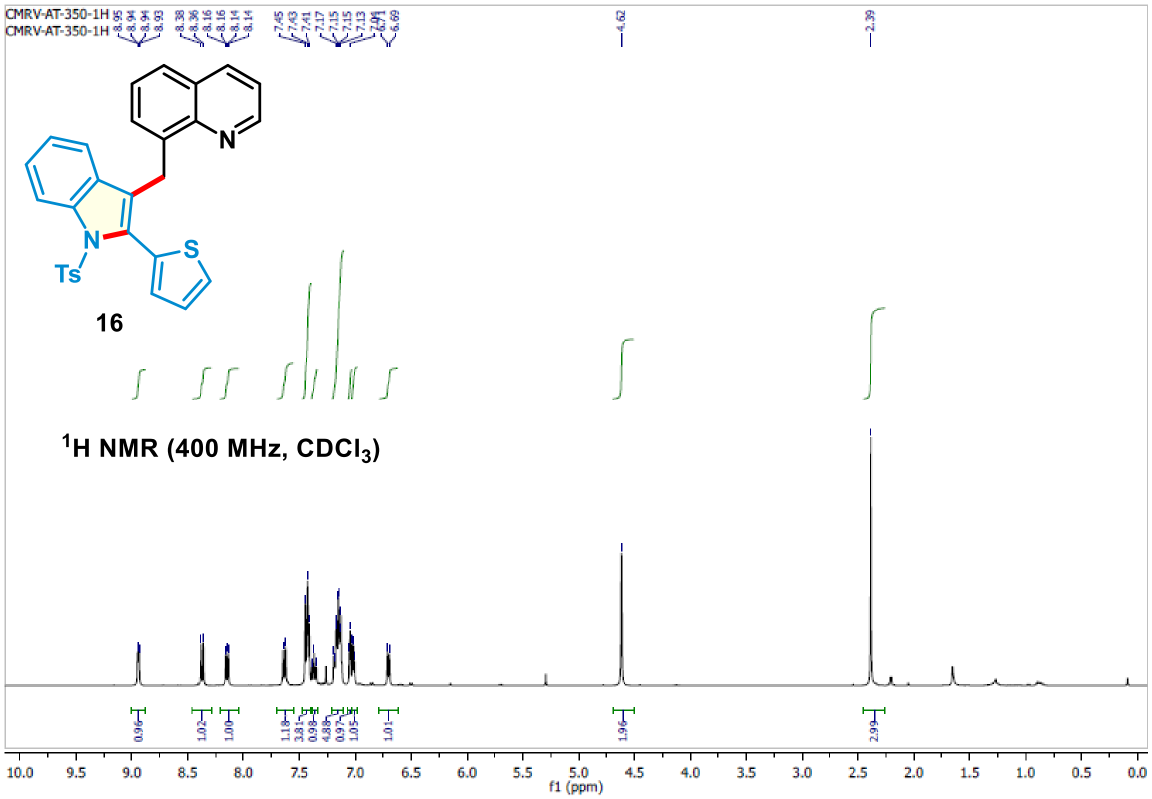
CMRV-AT-350-1H
CMRV-AT-350-1H
8.95
8.94
8.94
8.93
8.38
8.36
8.16
8.16
8.14
8.14
7.45
7.43
7.41
7.17
7.15
7.15
7.13
6.69
4.62
2.39
N
S
Ts
16
1H NMR (400 MHz, CDCl3)
0.96
1.02
1.00
1.18
3.81
0.98
4.88
0.97
1.05
1.01
1.96
2.99
10.0 9.5 9.0 8.5 8.0 7.5 7.0 6.5 6.0 5.5 5.0 4.5 4.0 3.5 3.0 2.5 2.0 1.5 1.0 0.5 0.0
f1 (ppm)

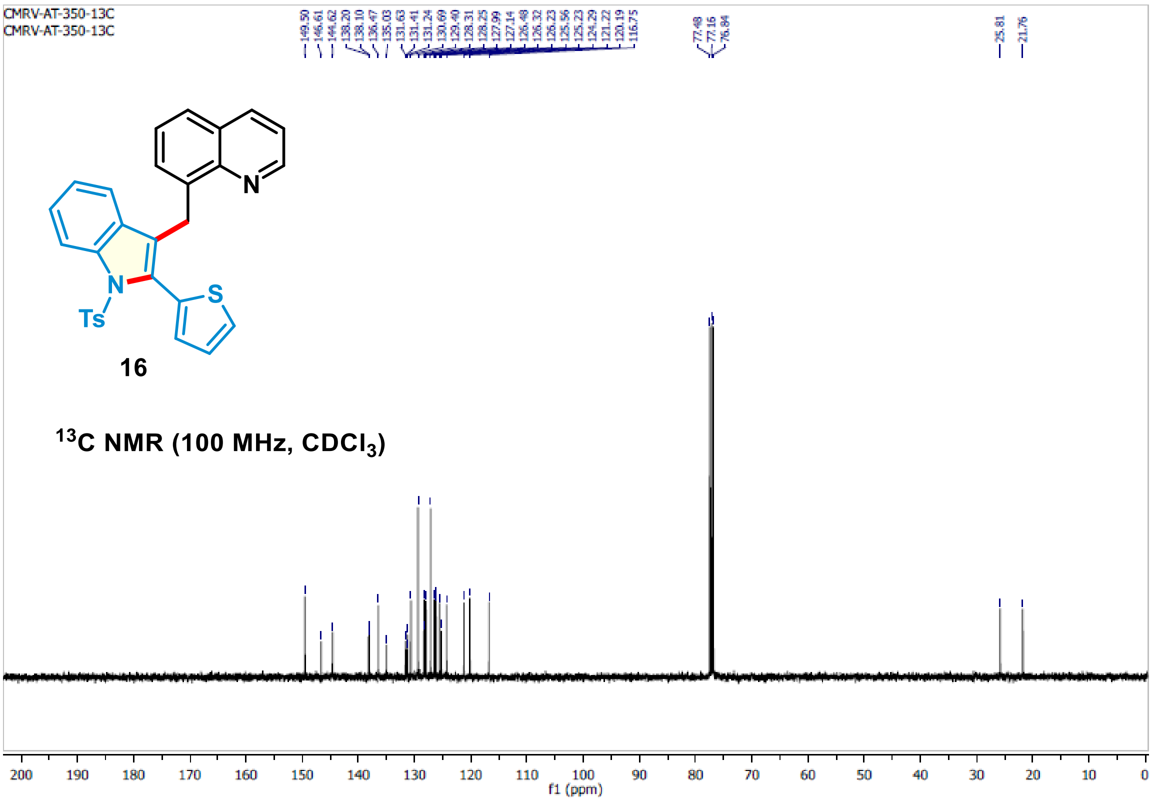
CMRV-AT-350-13C
CMRV-AT-350-13C
N
S
N
Ts
16
13C NMR (100 MHz, CDCl3)
f1 (ppm)

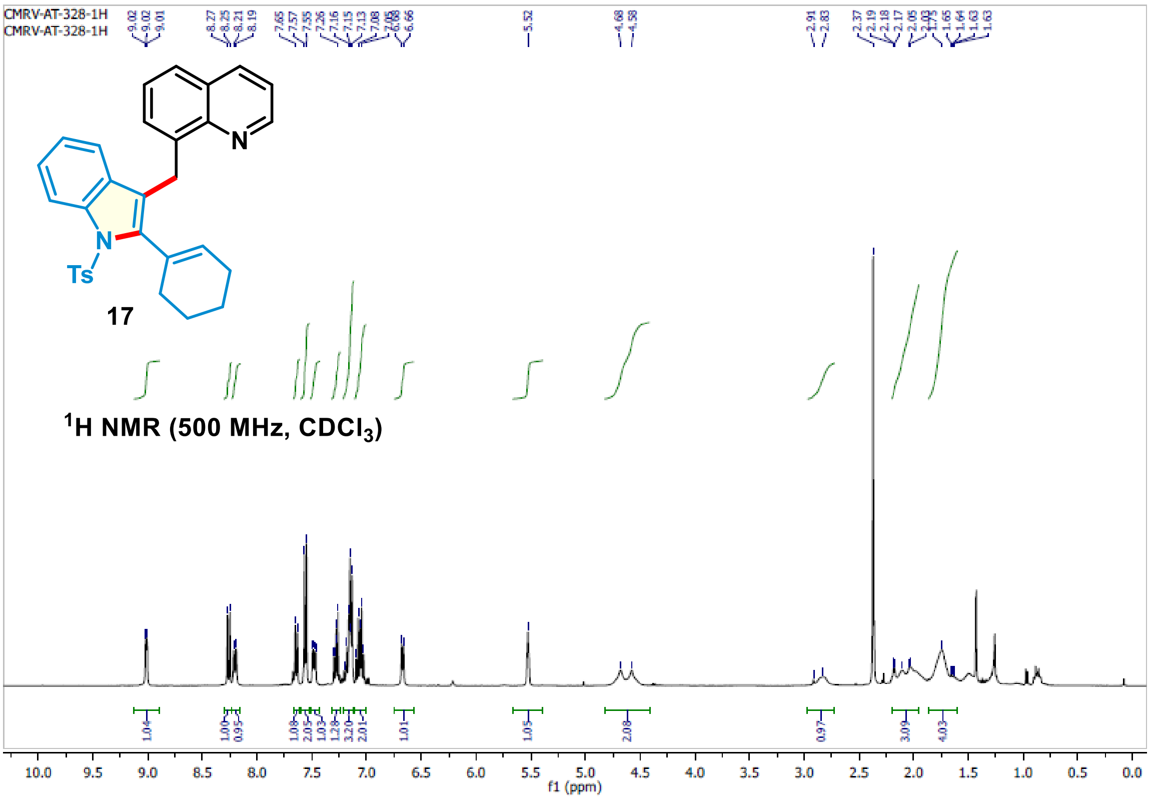
CMRV-AT-328-1H
CMRV-AT-328-1H
N
N
Ts
17
1H NMR (500 MHz, CDCl3)
f1 (ppm)

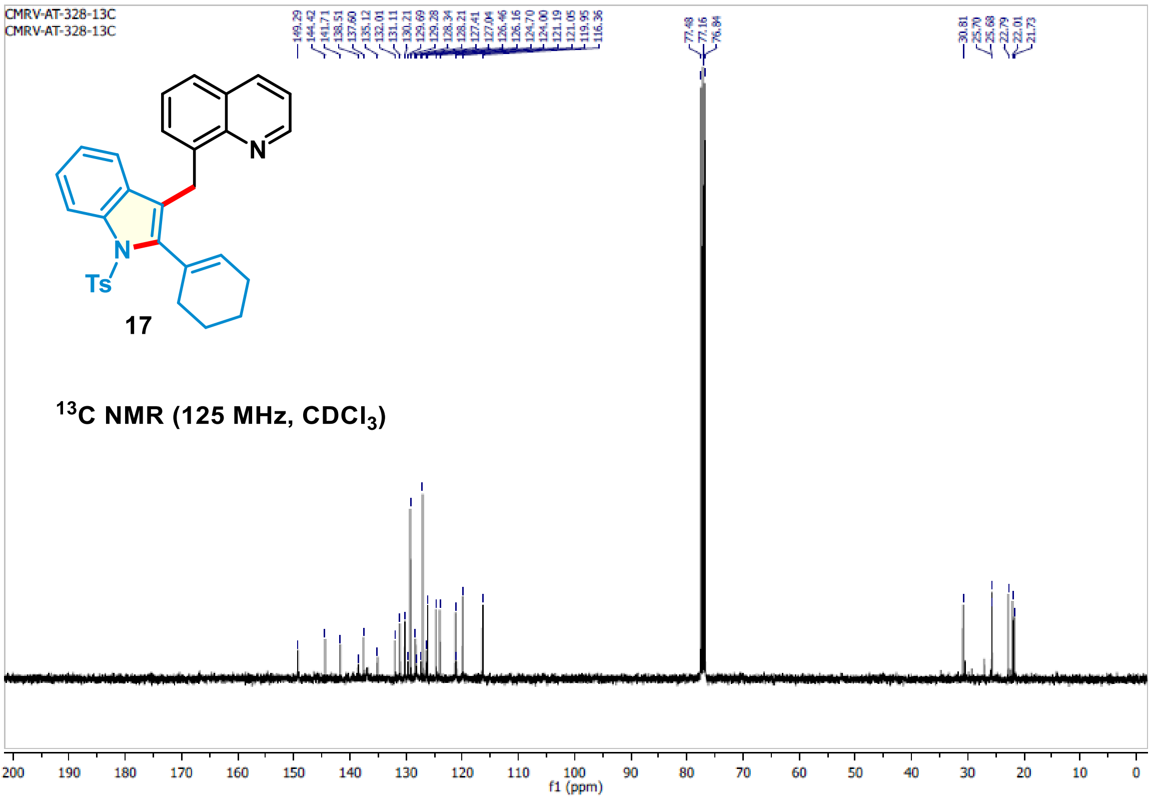
CMRV-AT-328-13C
CMRV-AT-328-13C
17
^{13}C NMR (125 MHz, CDCl$_3$)
f1 (ppm)

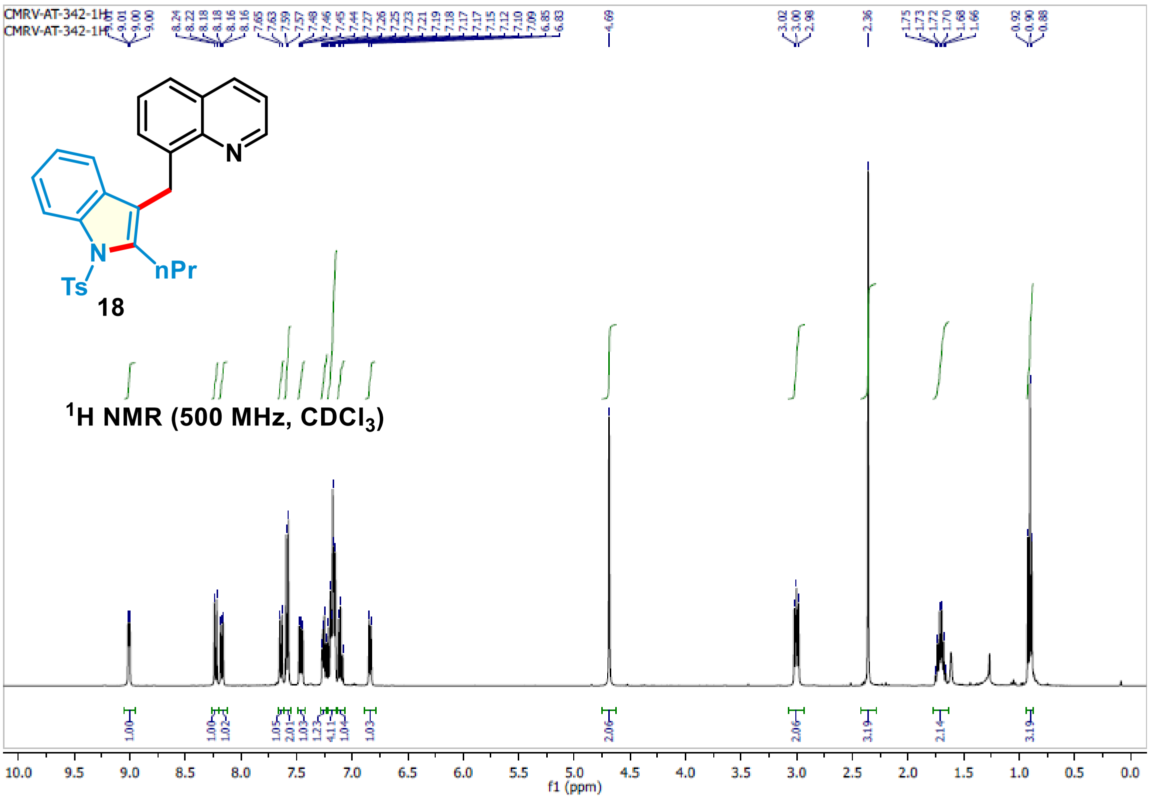
CMRV-AT-342-1H
CMRV-AT-342-1H
18
^{1}H NMR (500 MHz, CDCl$_3$)
f1 (ppm)

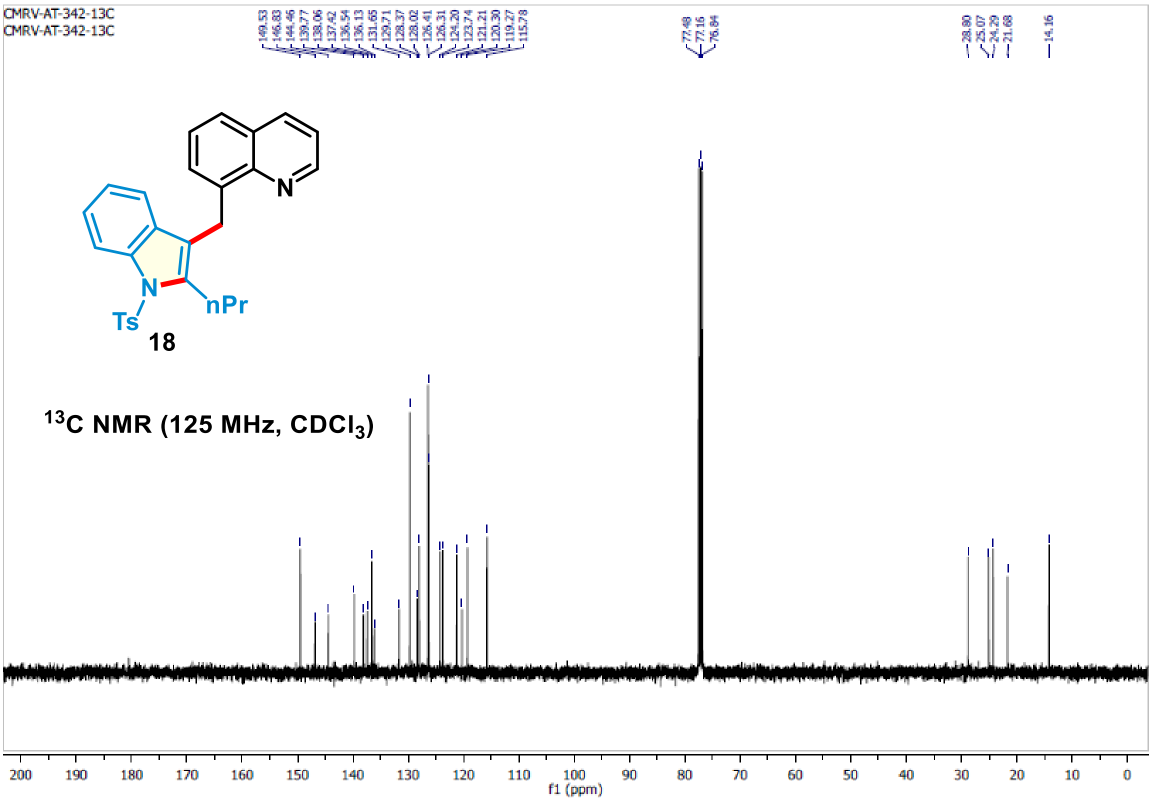
CMRV-AT-342-13C
CMRV-AT-342-13C
18
nPr
N
Ts
^{13}C NMR (125 MHz, CDCl$_3$)
f1 (ppm)

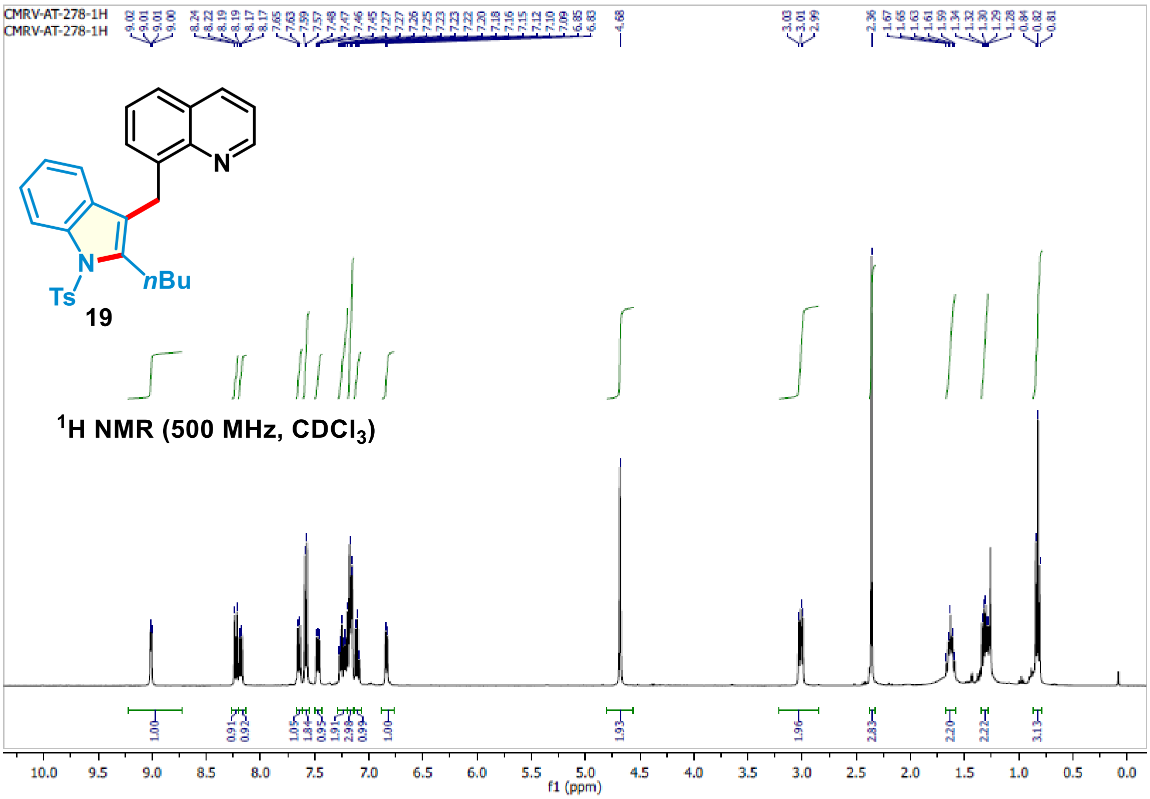
CMRV-AT-278-1H
CMRV-AT-278-1H
19
nBu
N
Ts
^{1}H NMR (500 MHz, CDCl$_3$)
f1 (ppm)

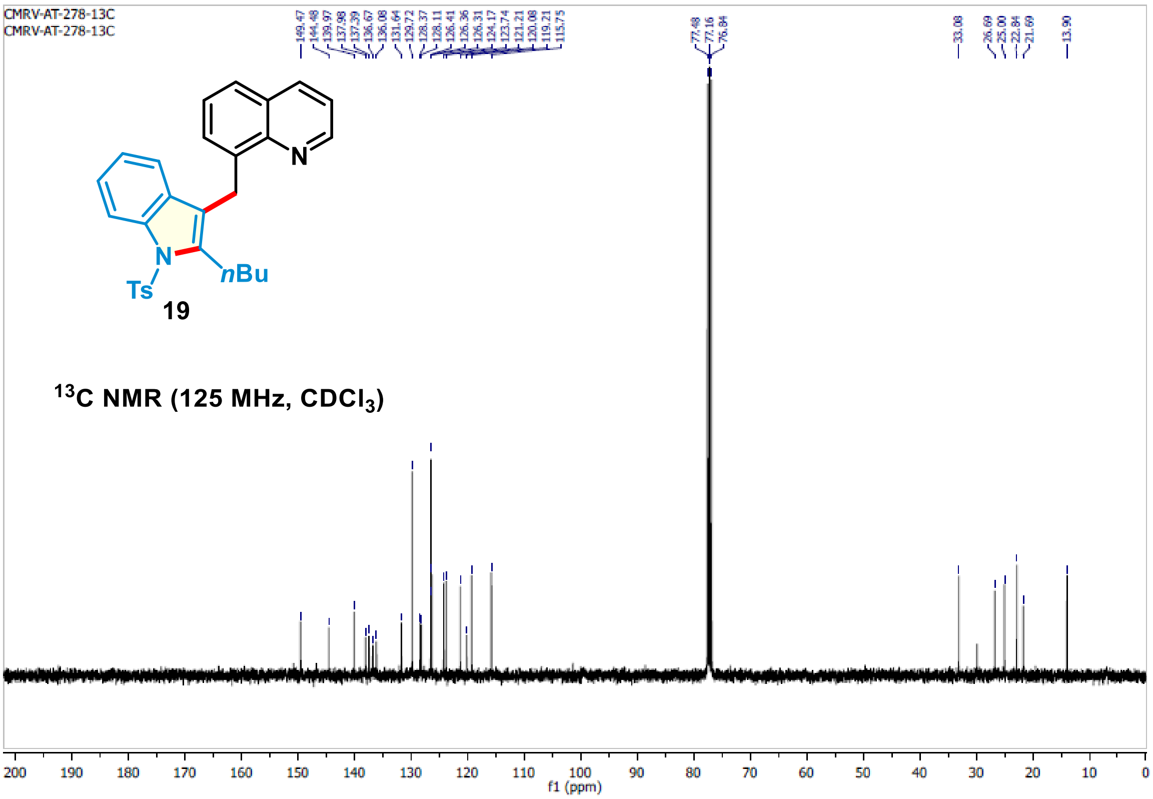
CMRV-AT-278-13C
CMRV-AT-278-13C
149.47
144.48
139.97
137.98
137.39
136.67
136.08
131.64
129.72
128.37
128.11
126.41
126.36
126.31
124.17
123.74
121.21
120.08
119.21
115.75
77.48
77.16
76.84
33.08
26.69
25.00
22.84
21.69
13.90
N
nBu
Ts
19
^{13}C NMR (125 MHz, $CDCl_3$)
f1 (ppm)

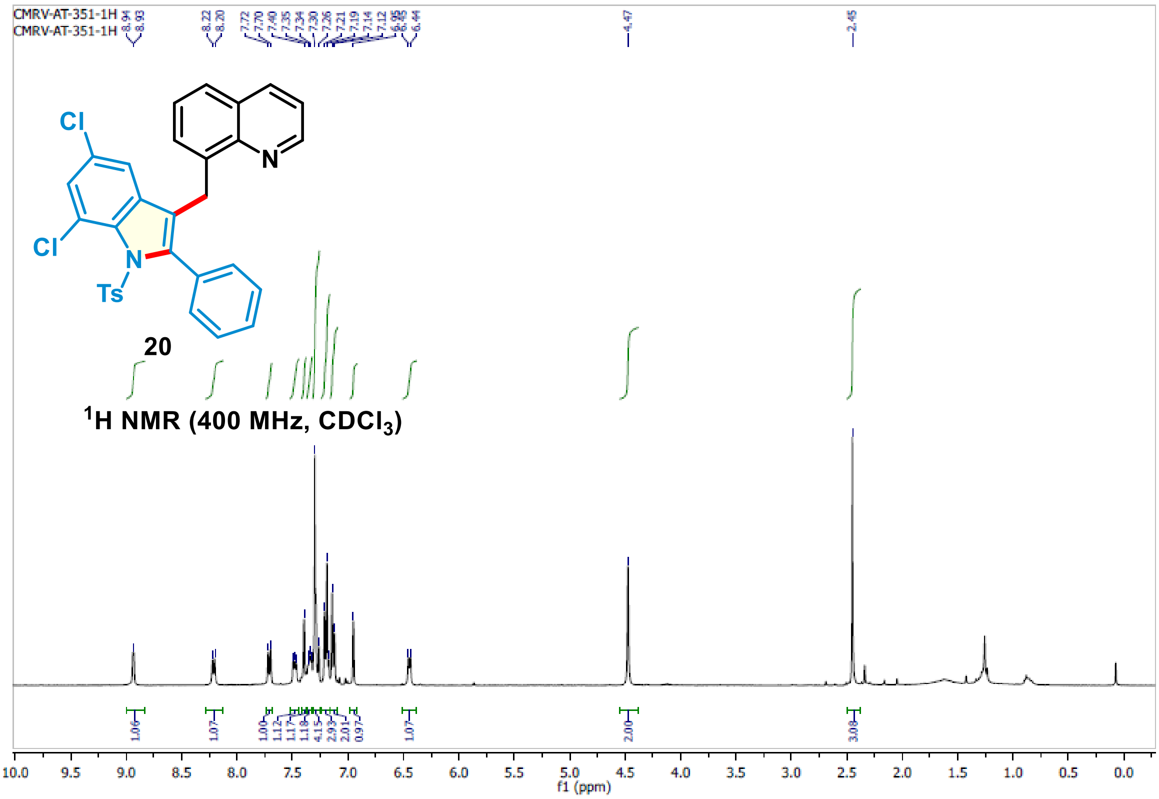
CMRV-AT-351-1H
CMRV-AT-351-1H
8.94
8.93
8.22
8.20
7.72
7.70
7.40
7.35
7.34
7.30
7.26
7.21
7.19
7.14
7.12
6.95
6.45
6.44
4.47
2.45
Cl
Cl
N
N
Ts
20
^{1}H NMR (400 MHz, $CDCl_3$)
1.06
1.07
1.00
1.12
1.17
1.18
4.15
2.93
2.01
0.97
1.07
2.00
3.08
f1 (ppm)

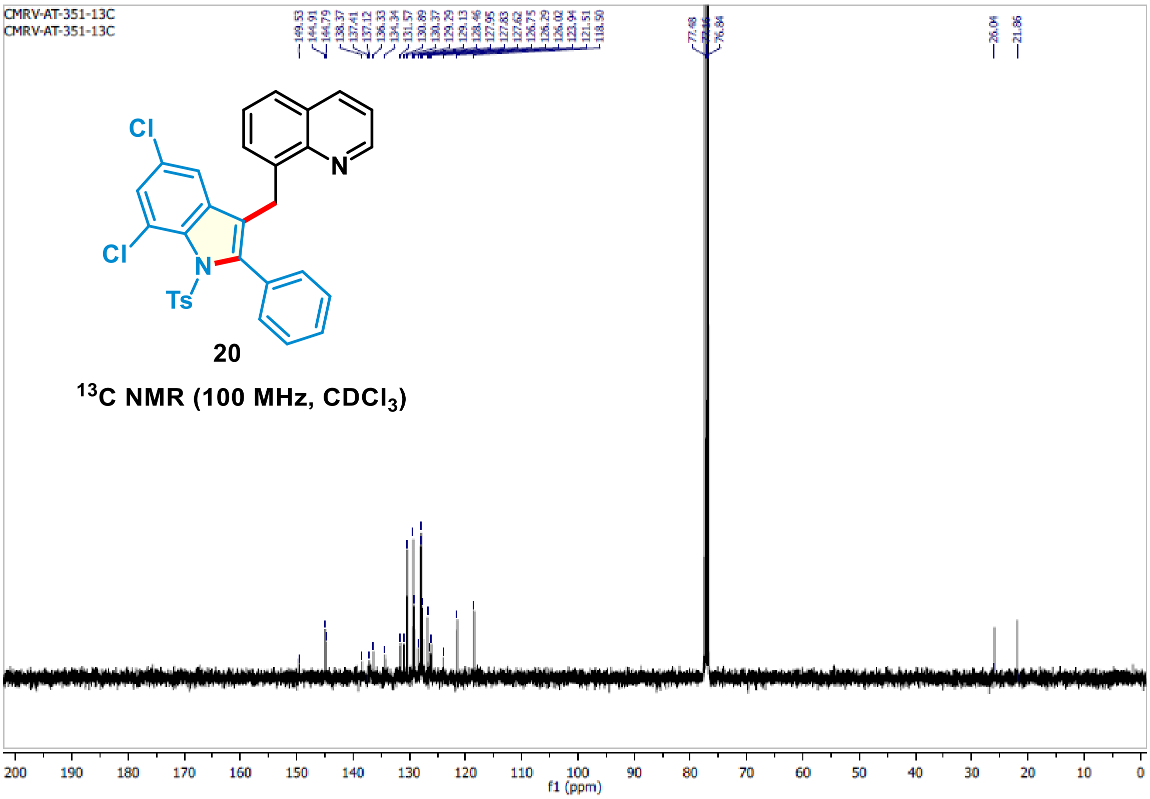
CMRV-AT-351-13C
CMRV-AT-351-13C
20
^{13}C NMR (100 MHz, CDCl$_3$)
f1 (ppm)

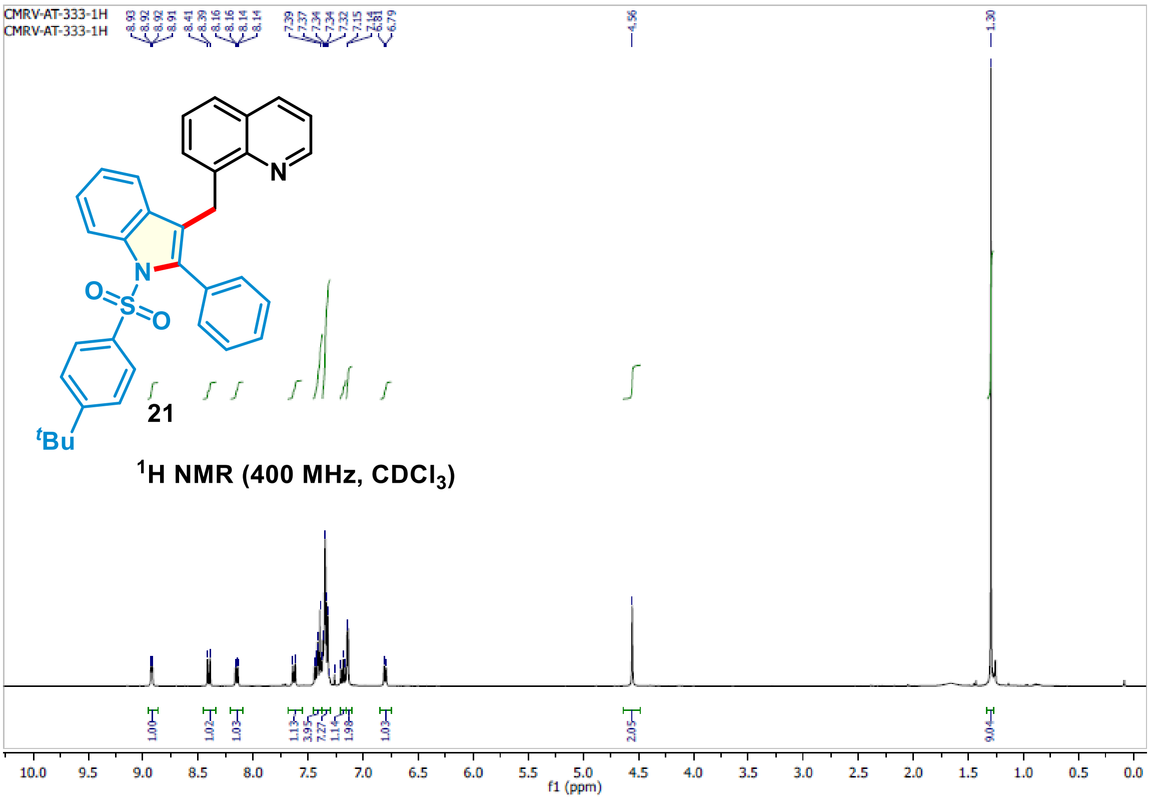
CMRV-AT-333-1H
CMRV-AT-333-1H
21
^{1}H NMR (400 MHz, CDCl$_3$)
f1 (ppm)

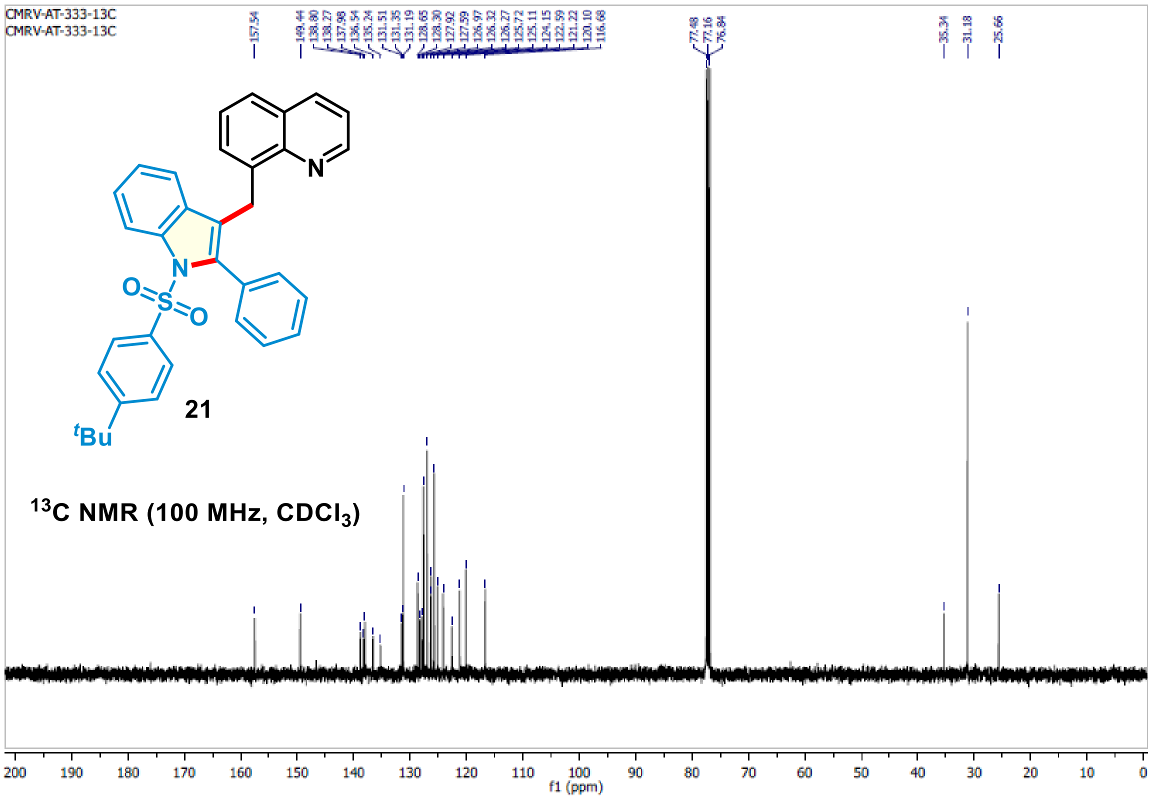
CMRV-AT-333-13C
CMRV-AT-333-13C
157.54
149.44
138.80
138.27
137.98
136.54
135.24
131.51
131.35
131.19
128.65
128.30
127.92
127.59
126.97
126.32
126.27
125.72
125.11
124.15
122.59
121.22
120.10
116.68
77.48
77.16
76.84
35.34
31.18
25.66
N
N
O
S
O
^{t}Bu
21
^{13}C NMR (100 MHz, $CDCl_3$)
f1 (ppm)

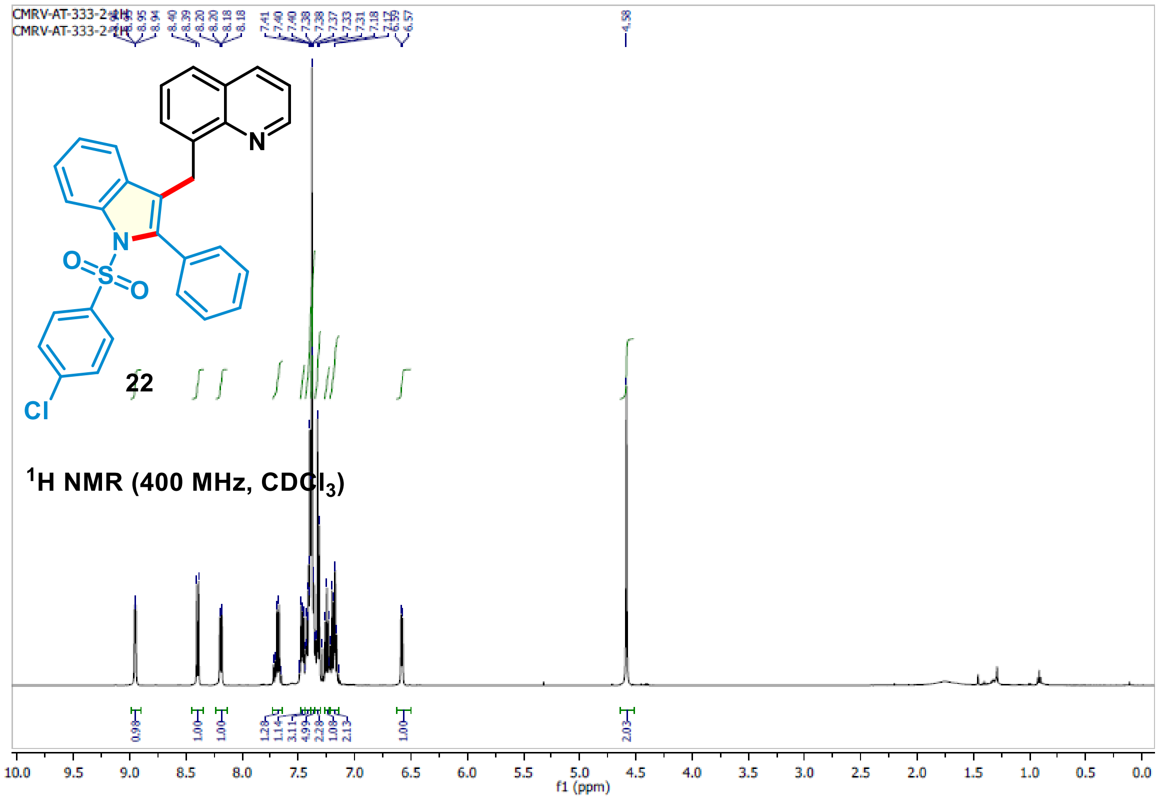
CMRV-AT-333-24H
CMRV-AT-333-24H
8.95
8.94
8.40
8.39
8.20
8.20
8.18
8.18
7.41
7.40
7.40
7.38
7.37
7.33
7.31
7.18
7.17
6.59
6.57
4.58
N
N
O
S
O
Cl
22
^{1}H NMR (400 MHz, $CDCl_3$)
0.98
1.00
1.00
1.28
1.14
3.11
4.99
2.28
1.08
2.13
1.00
2.03
f1 (ppm)

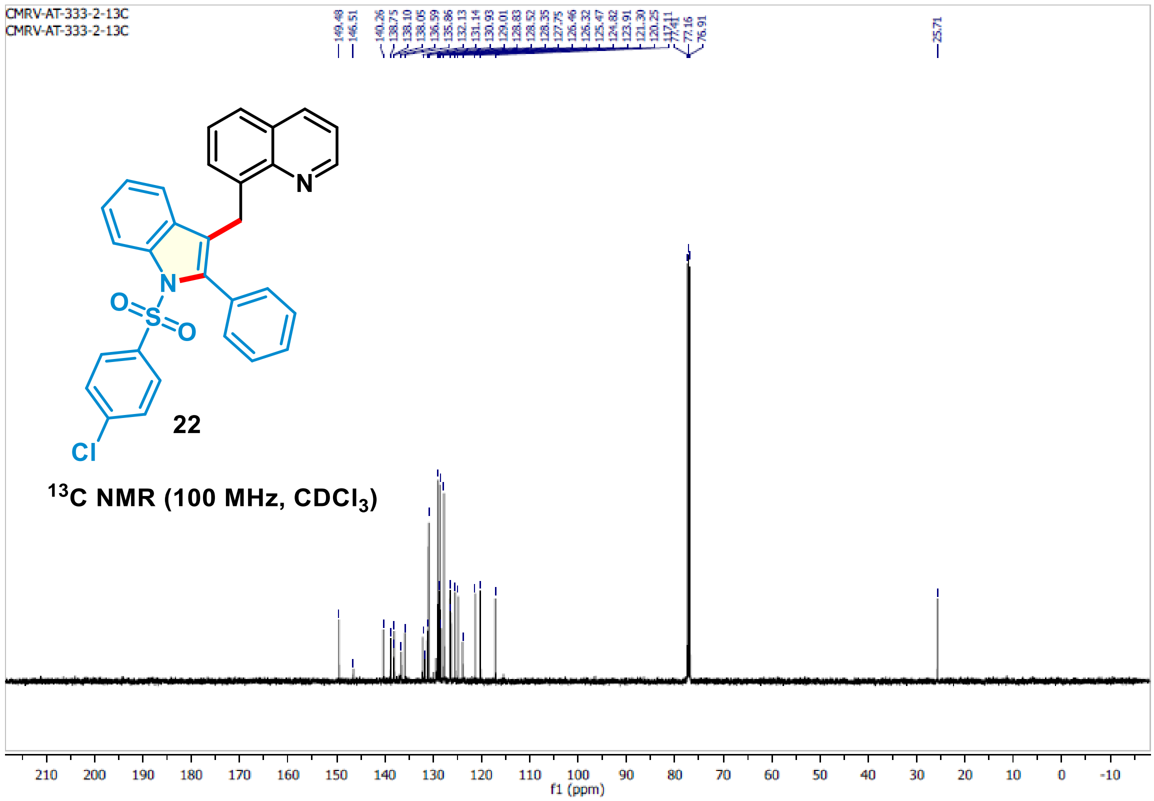
CMRV-AT-333-2-13C
CMRV-AT-333-2-13C
22
^{13}C NMR (100 MHz, CDCl$_3$)
f1 (ppm)

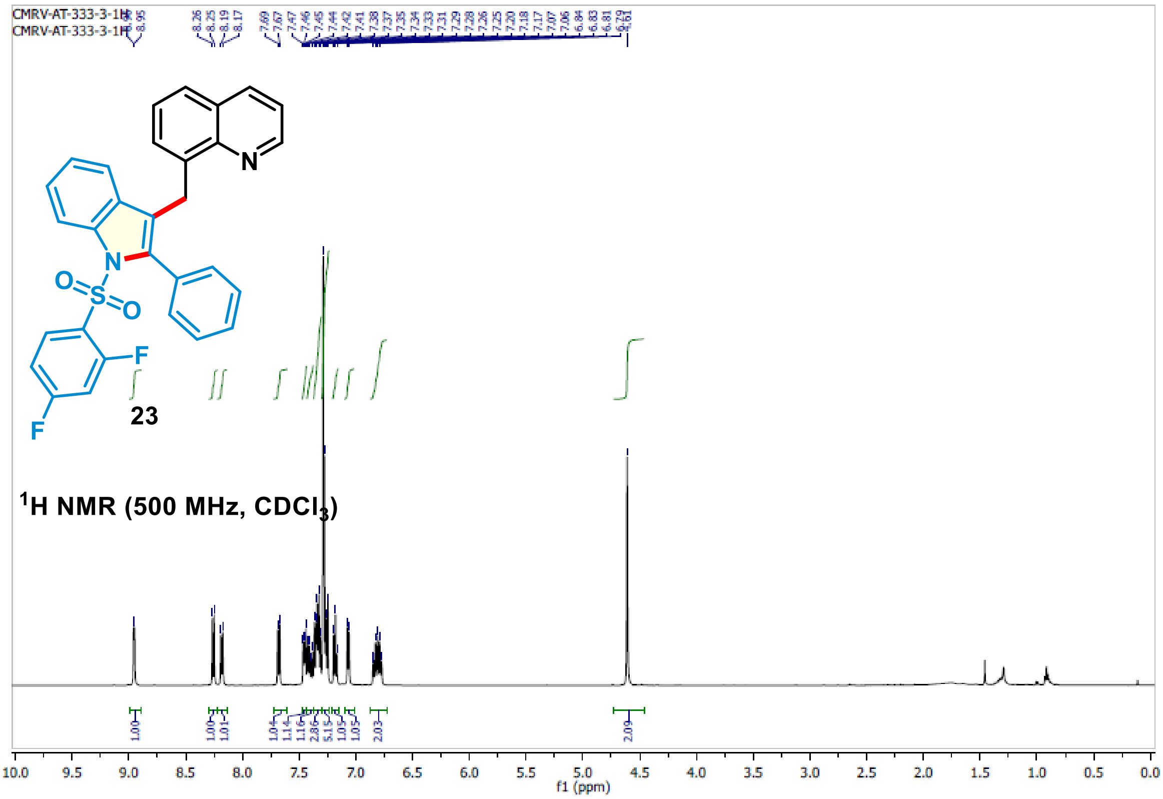
CMRV-AT-333-3-1H
CMRV-AT-333-3-1H
23
^{1}H NMR (500 MHz, CDCl$_3$)
f1 (ppm)

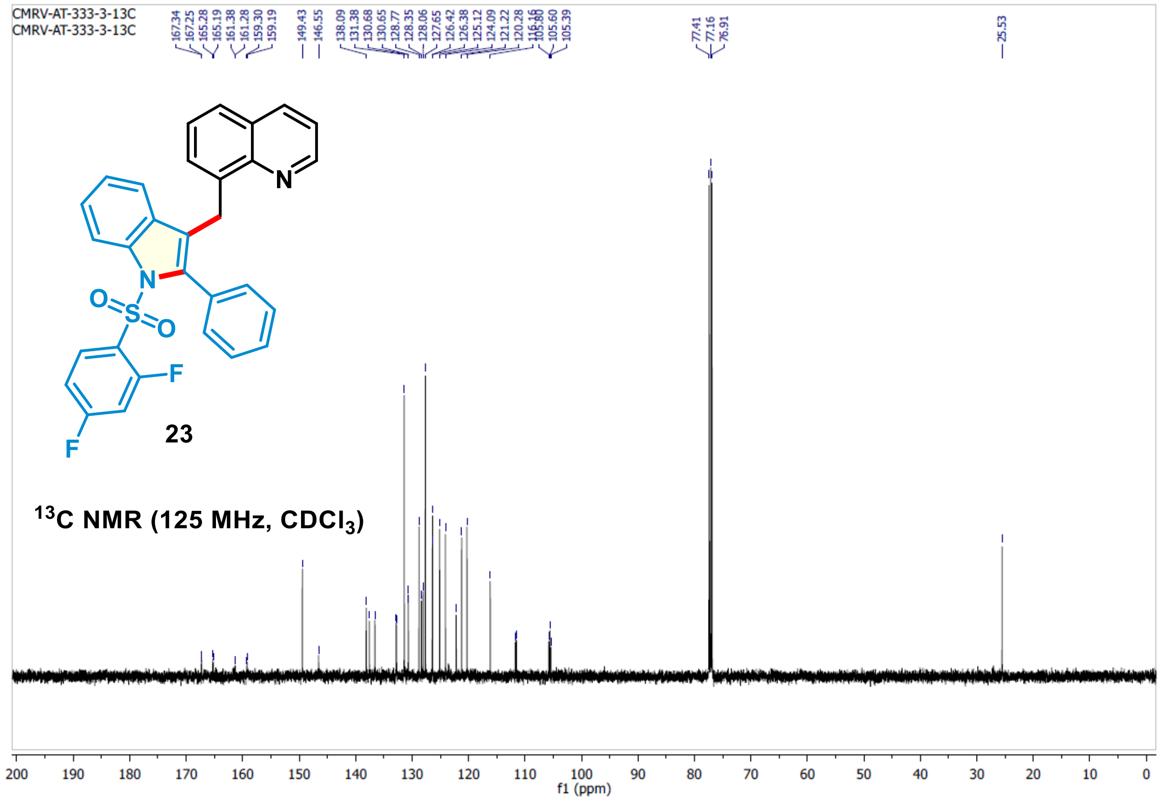
CMRV-AT-333-3-13C
CMRV-AT-333-3-13C
167.34
167.25
165.28
165.19
161.38
161.28
159.30
159.19
149.43
146.55
138.09
131.38
130.68
130.65
128.77
128.35
128.06
127.65
126.42
126.38
125.12
124.09
121.22
120.28
105.60
105.39
77.41
77.16
76.91
25.53
N
O
S
O
F
F
23
13C NMR (125 MHz, CDCl3)
200
190
180
170
160
150
140
130
120
110
100
90
80
70
60
50
40
30
20
10
0
f1 (ppm)

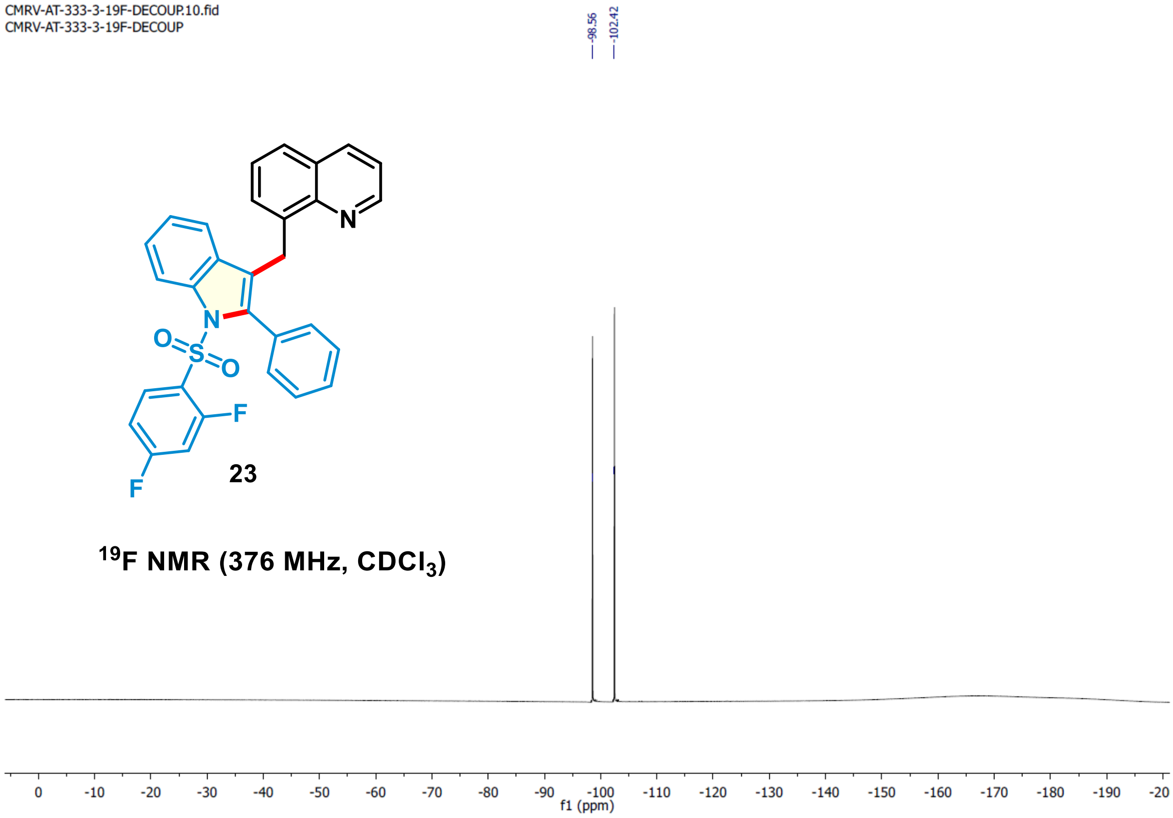
CMRV-AT-333-3-19F-DECOUP.10.fid
CMRV-AT-333-3-19F-DECOUP
-98.56
-102.42
N
N
O
S
O
F
F
23
19F NMR (376 MHz, CDCl3)
0
-10
-20
-30
-40
-50
-60
-70
-80
-90
-100
-110
-120
-130
-140
-150
-160
-170
-180
-190
-20
f1 (ppm)

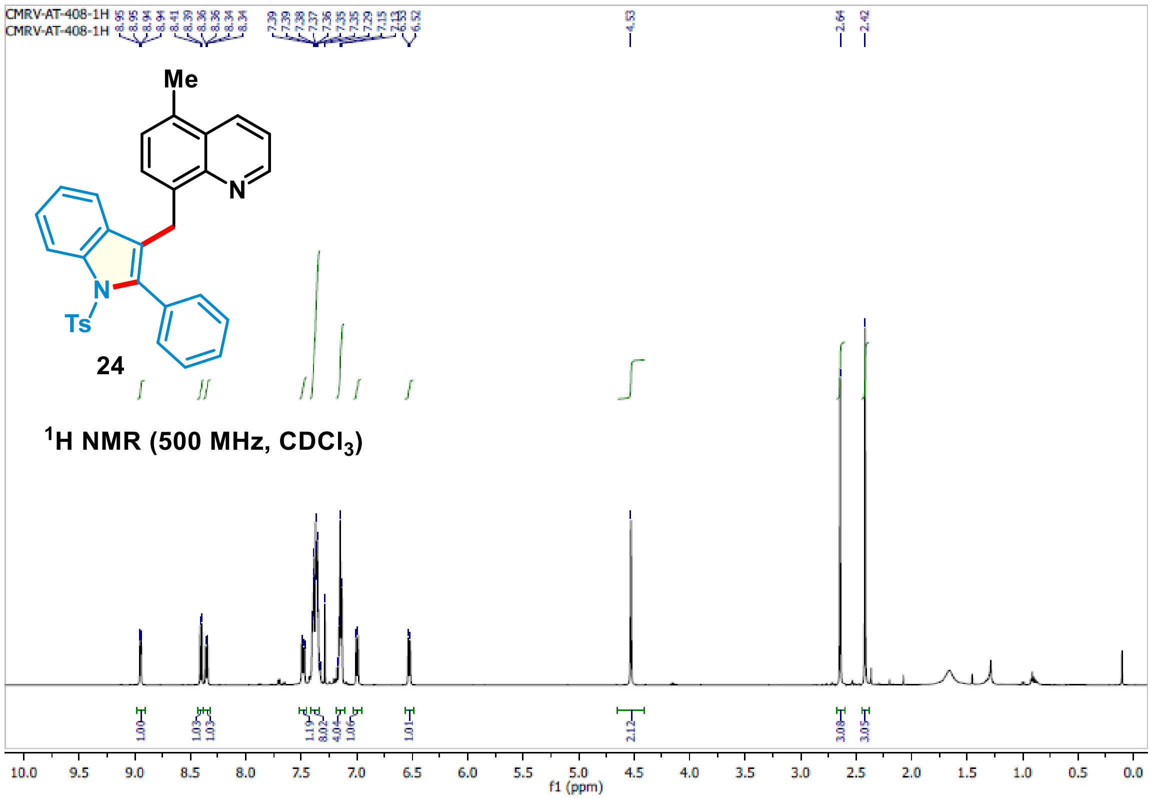
CMRV-AT-408-1H
8.95 8.95 8.94 8.94 8.41 8.39 8.36 8.36 8.34 8.34 7.39 7.39 7.38 7.37 7.36 7.35 7.35 7.29 7.15 7.13 6.53 6.52
4.53
2.64
2.42
Me
N
N
Ts
24
^{1}H NMR (500 MHz, CDCl$_3$)
1.00 1.03 1.03 1.19 8.02 4.04 1.06 1.01 2.12 3.08 3.05
f1 (ppm)

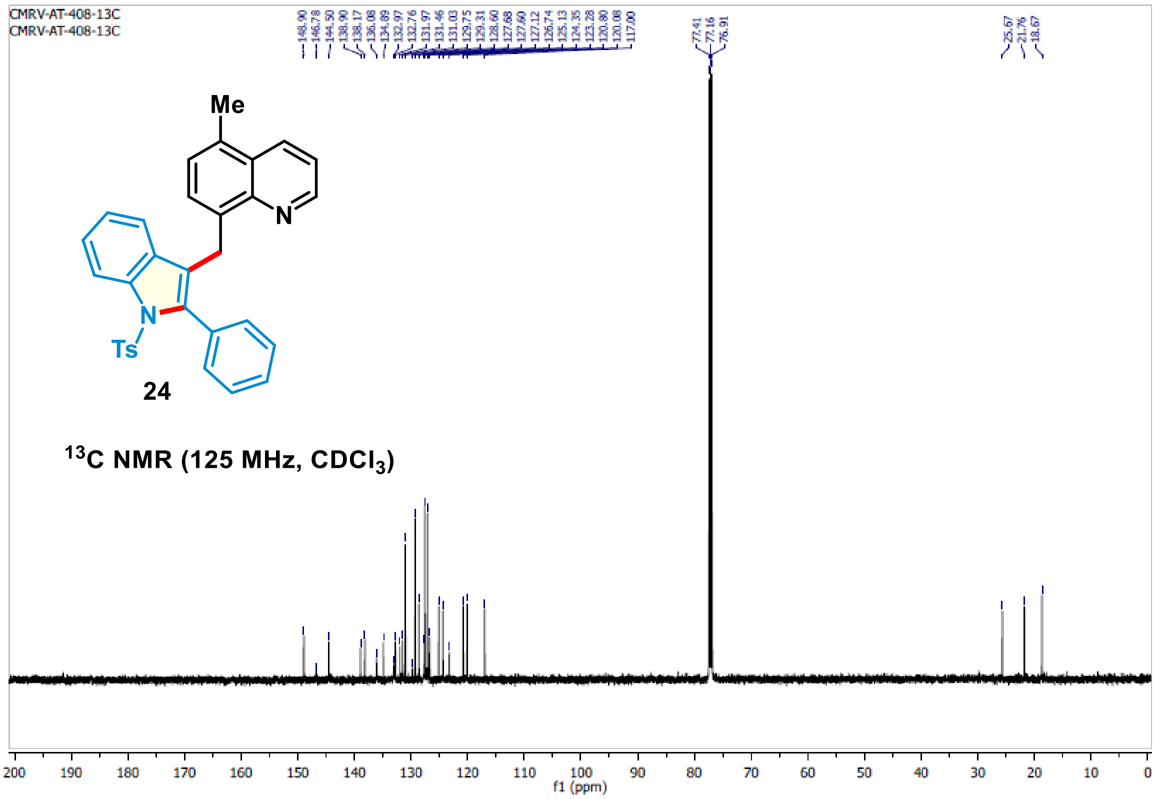
CMRV-AT-408-13C
148.90 146.78 144.50 138.90 138.17 136.08 134.89 132.97 132.76 131.97 131.46 131.03 129.75 129.31 128.60 127.68 127.60 127.12 126.74 125.13 124.35 123.28 120.80 120.08 117.00
77.41 77.16 76.91
25.67 21.76 18.67
Me
N
N
Ts
24
^{13}C NMR (125 MHz, CDCl$_3$)
f1 (ppm)

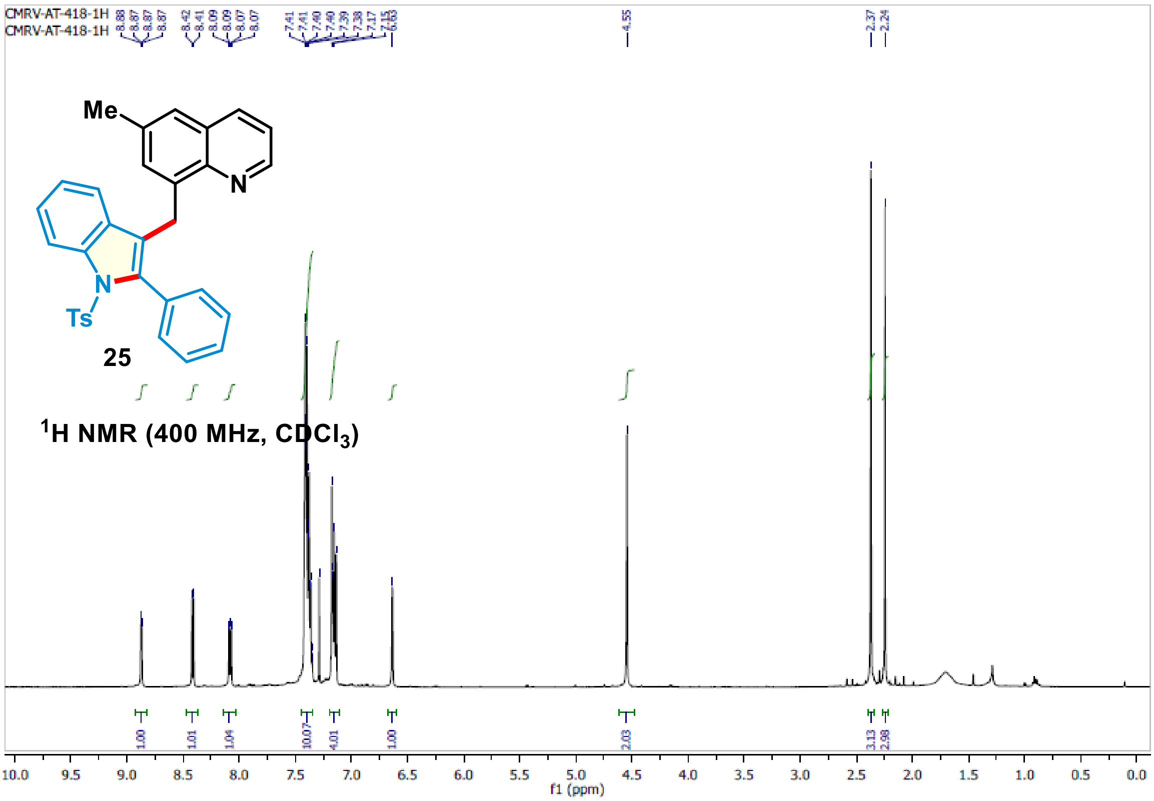
CMRV-AT-418-1H
CMRV-AT-418-1H
Me
N
N
Ts
25
1H NMR (400 MHz, CDCl3)
f1 (ppm)

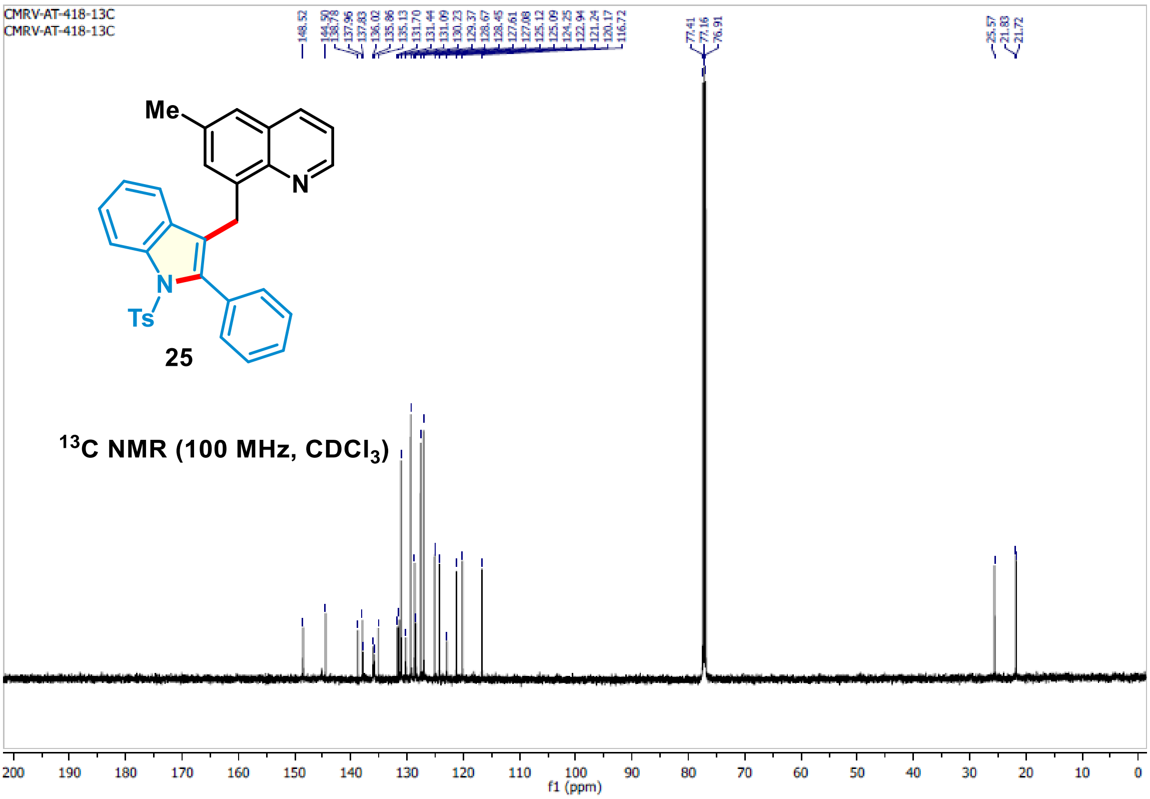
CMRV-AT-418-13C
CMRV-AT-418-13C
Me
N
N
Ts
25
13C NMR (100 MHz, CDCl3)
f1 (ppm)

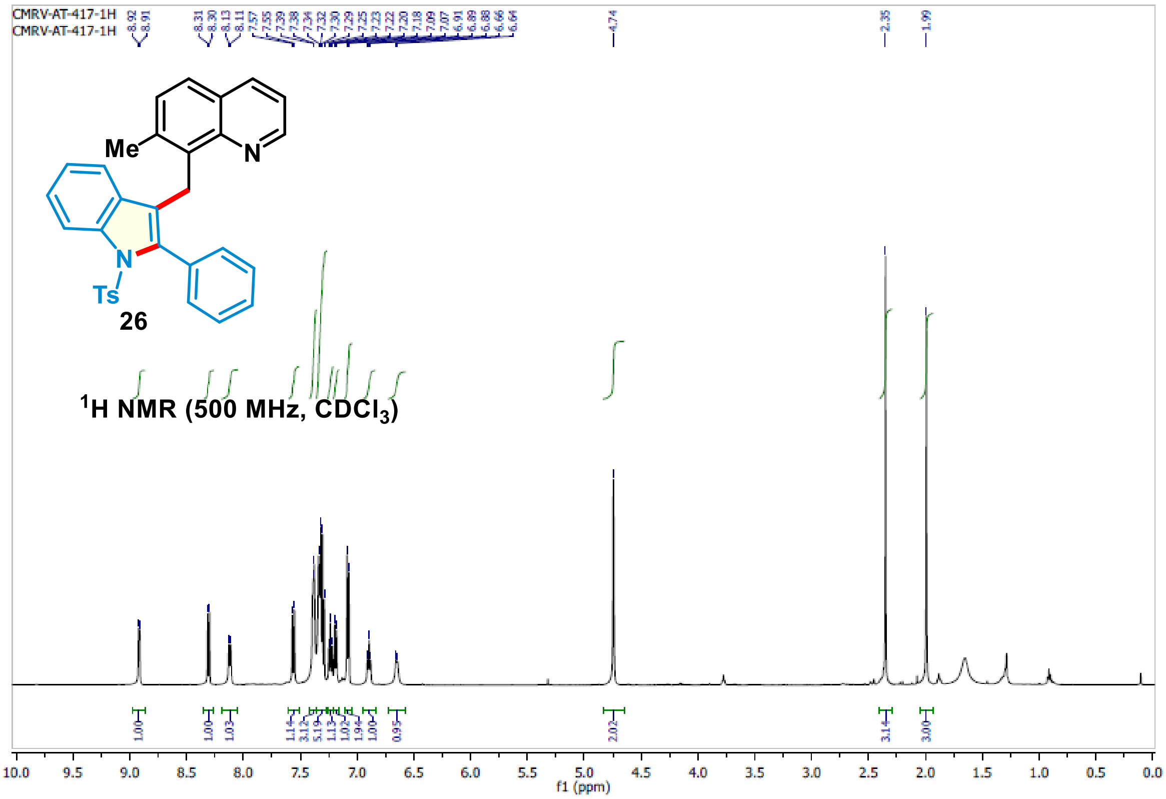
CMRV-AT-417-1H
Me
N
Ts
26
^{1}H NMR (500 MHz, CDCl$_3$)
f1 (ppm)

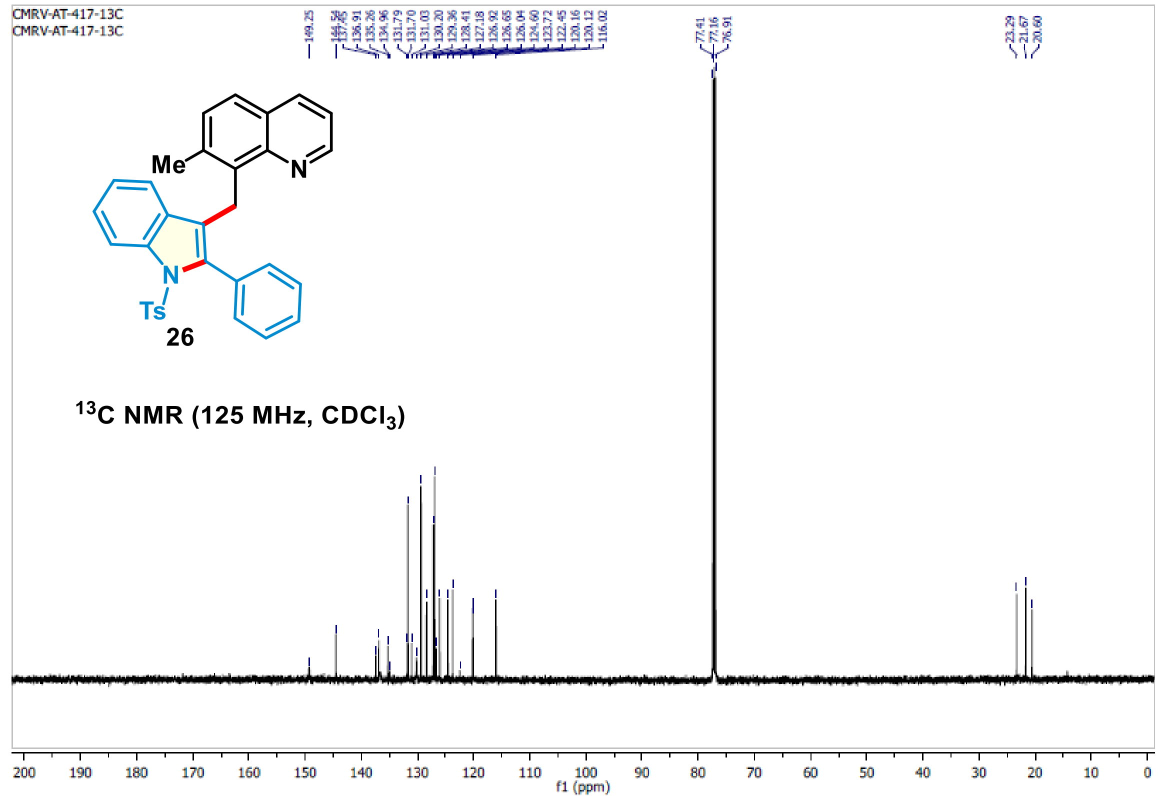
CMRV-AT-417-13C
Me
N
Ts
26
^{13}C NMR (125 MHz, CDCl$_3$)
f1 (ppm)

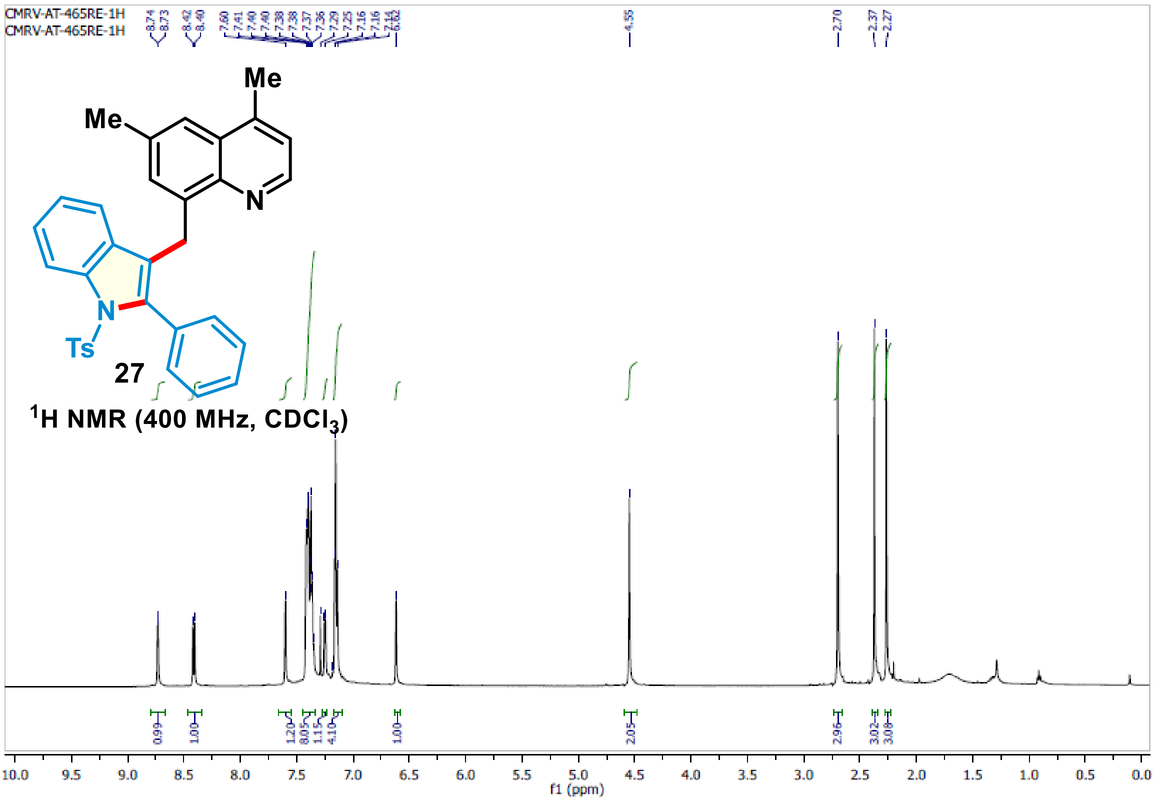
CMRV-AT-465RE-1H
CMRV-AT-465RE-1H
8.74 8.73 8.42 8.40 7.60 7.41 7.40 7.40 7.38 7.38 7.37 7.36 7.29 7.25 7.16 7.16 7.14 6.62
4.55
2.70
2.37 2.27
Me
Me
N
N
Ts
27
^{1}H NMR (400 MHz, $CDCl_3$)
0.99 1.00 1.20 8.05 1.15 4.10 1.00 2.05 2.96 3.02 3.08
f1 (ppm)

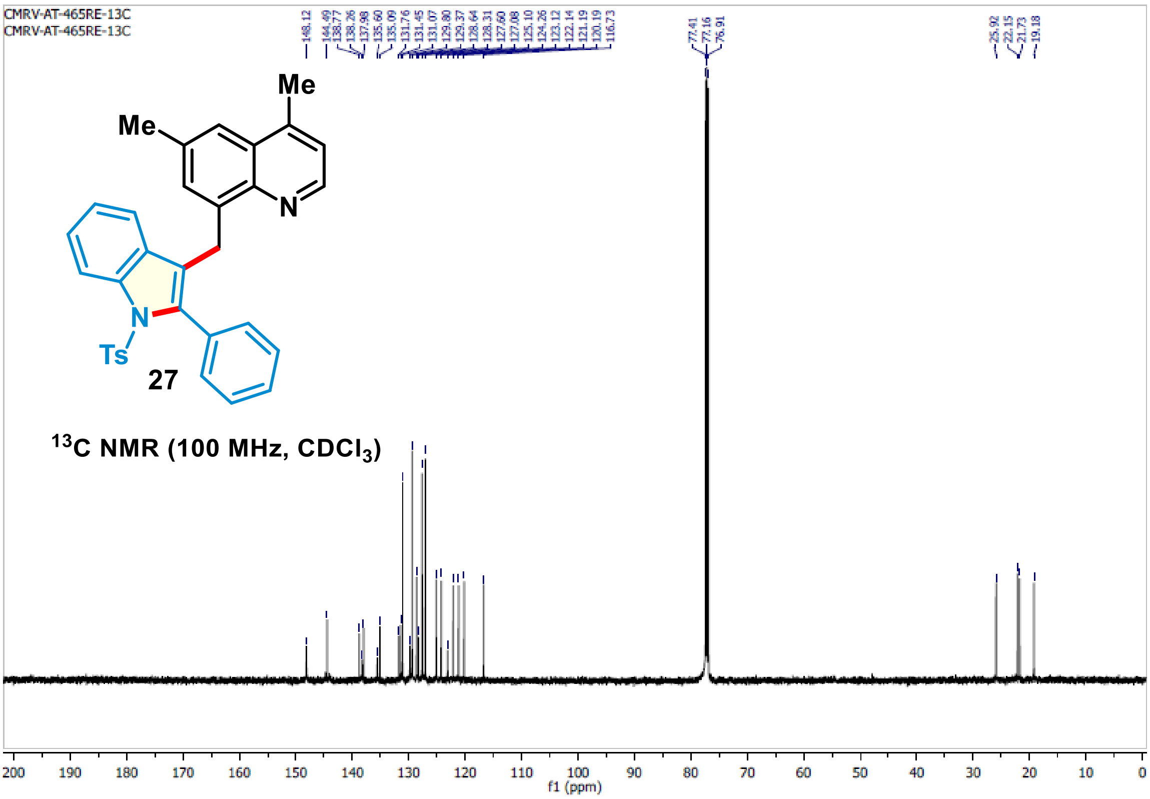
CMRV-AT-465RE-13C
CMRV-AT-465RE-13C
148.12 144.49 138.77 138.26 137.98 135.60 135.09 131.76 131.45 131.07 129.80 129.37 128.64 128.31 127.60 127.08 125.10 124.26 123.12 122.14 121.19 120.19 116.73
77.41 77.16 76.91
25.92 22.15 21.73 19.18
Me
Me
N
N
Ts
27
^{13}C NMR (100 MHz, $CDCl_3$)
f1 (ppm)

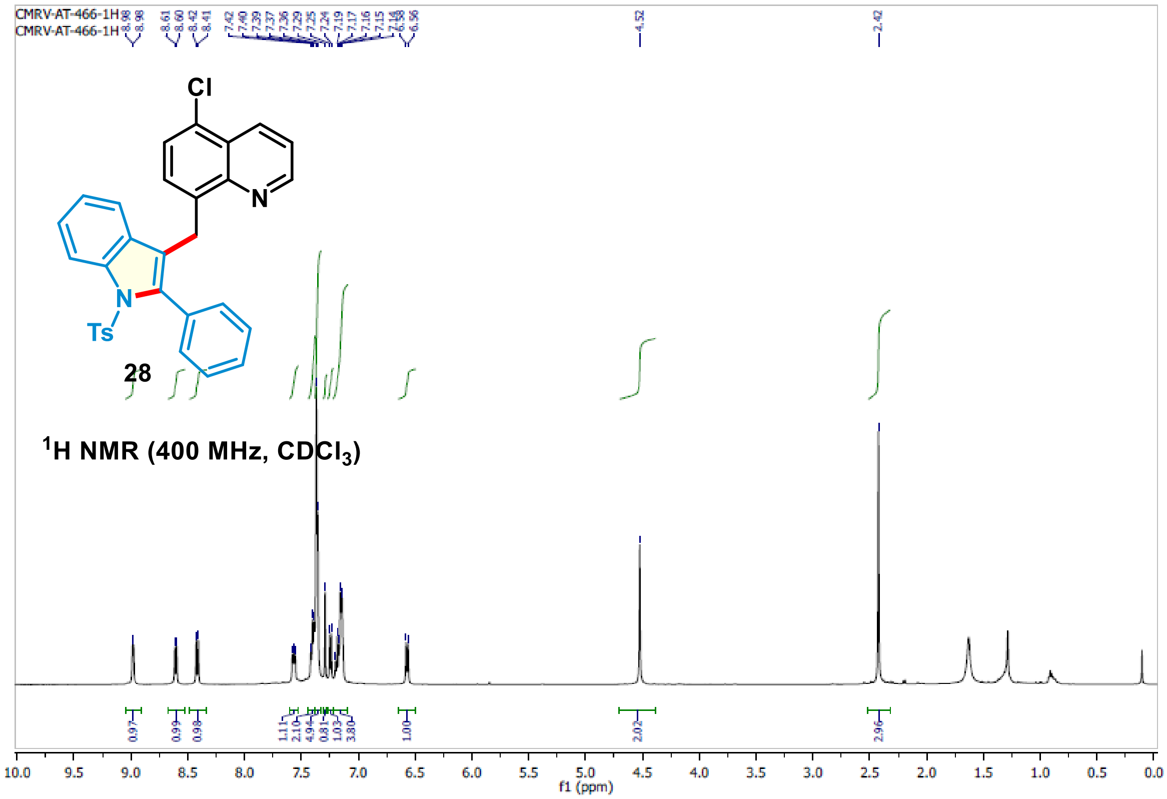
CMRV-AT-466-1H
28
^{1}H NMR (400 MHz, CDCl$_3$)
f1 (ppm)

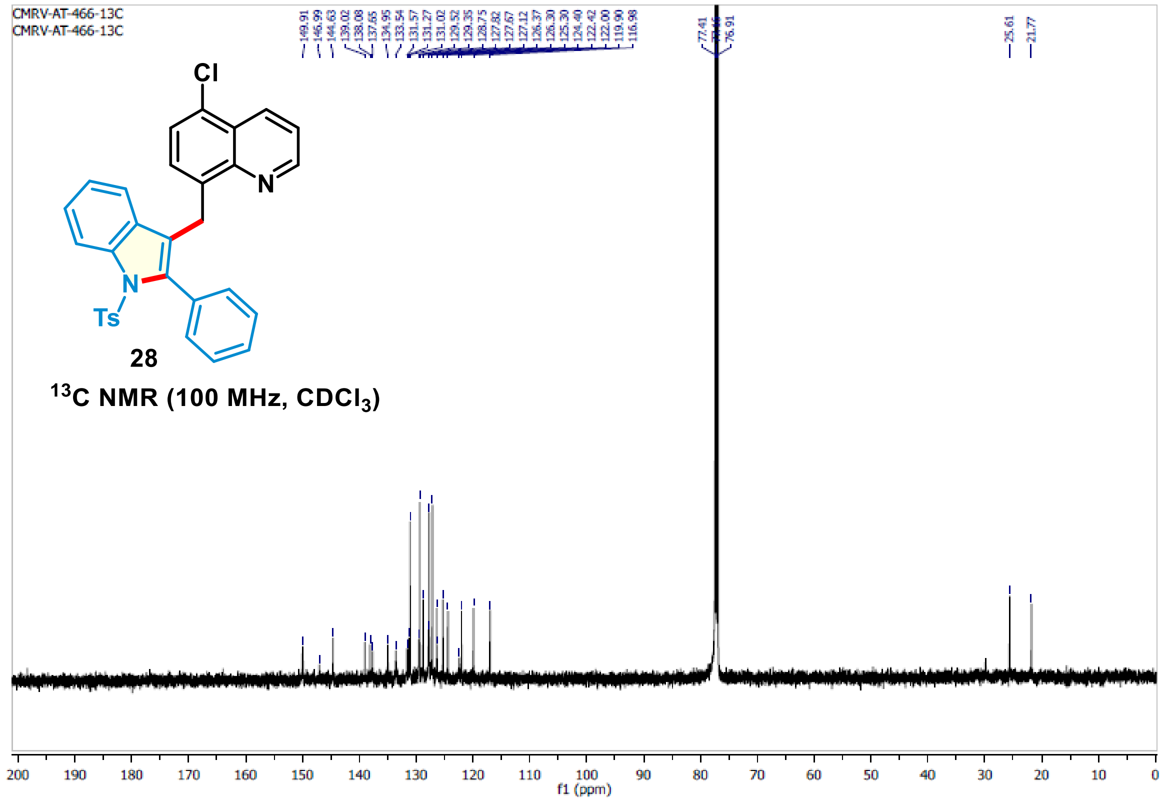
CMRV-AT-466-13C
28
^{13}C NMR (100 MHz, CDCl$_3$)
f1 (ppm)

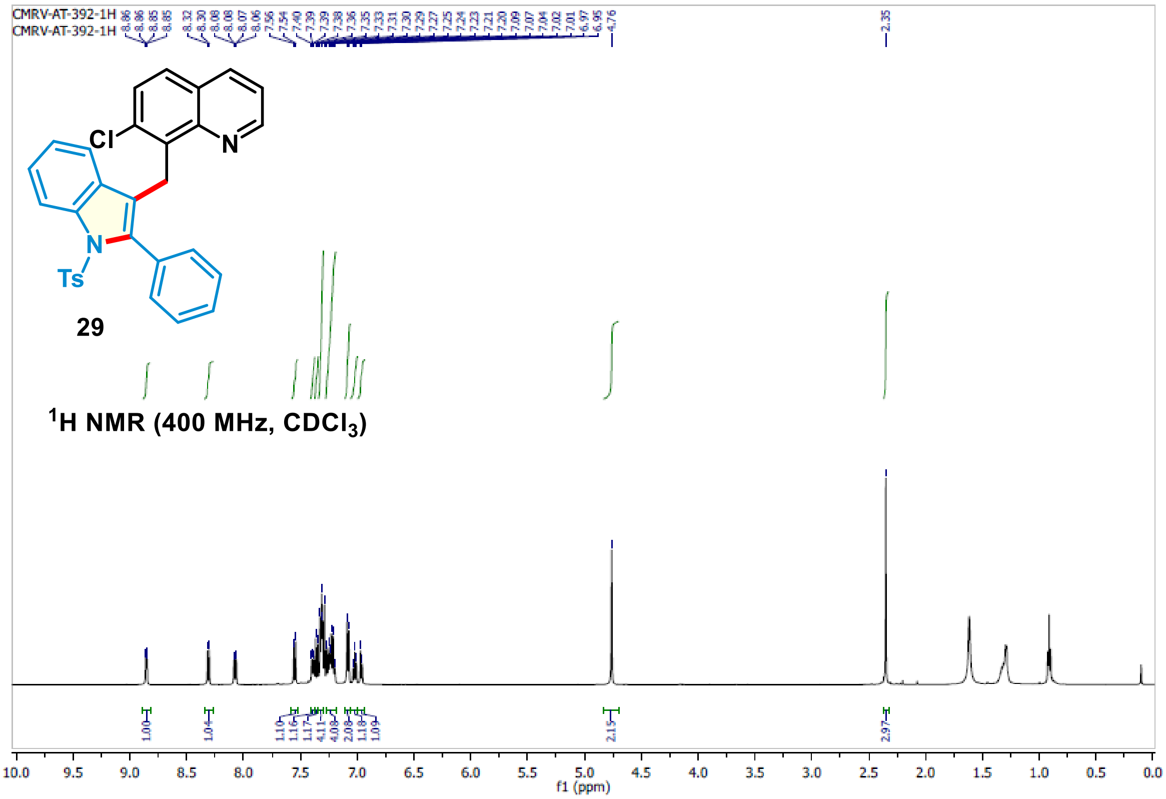
CMRV-AT-392-1H
CMRV-AT-392-1H
Cl
N
N
Ts
29
¹H NMR (400 MHz, CDCl₃)
f1 (ppm)

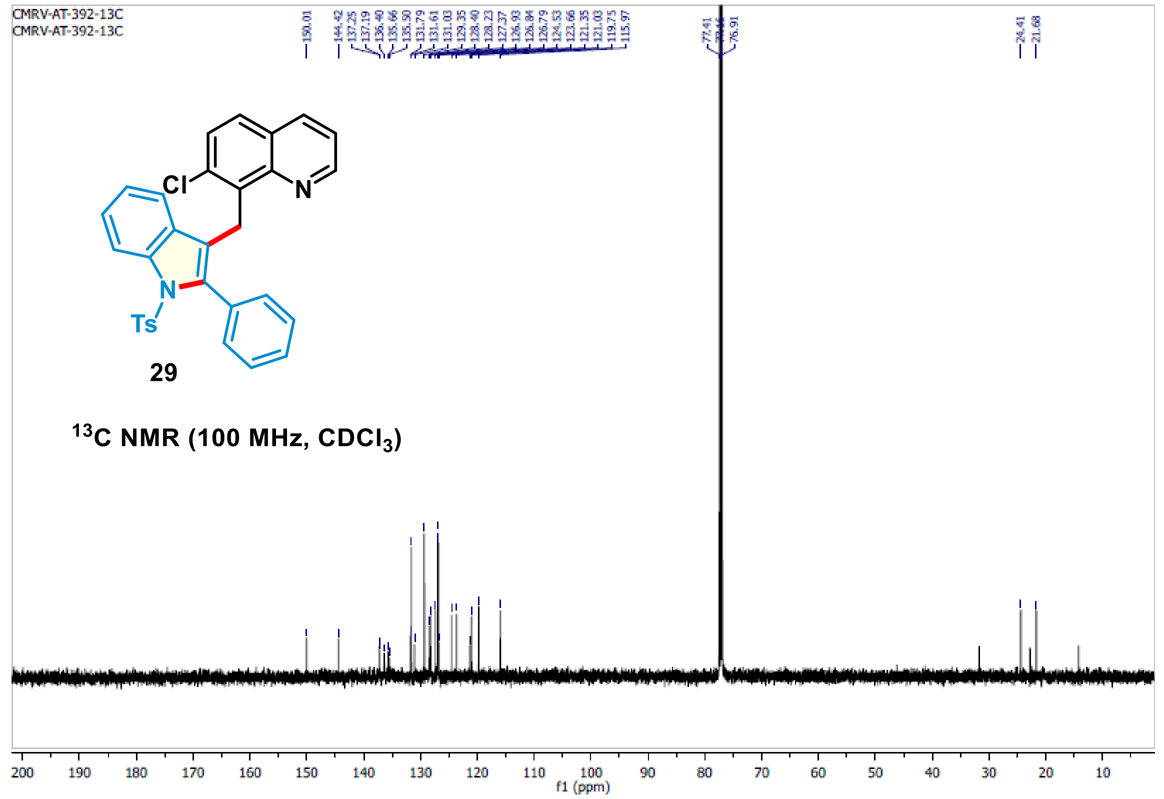
CMRV-AT-392-13C
CMRV-AT-392-13C
Cl
N
N
Ts
29
¹³C NMR (100 MHz, CDCl₃)
f1 (ppm)

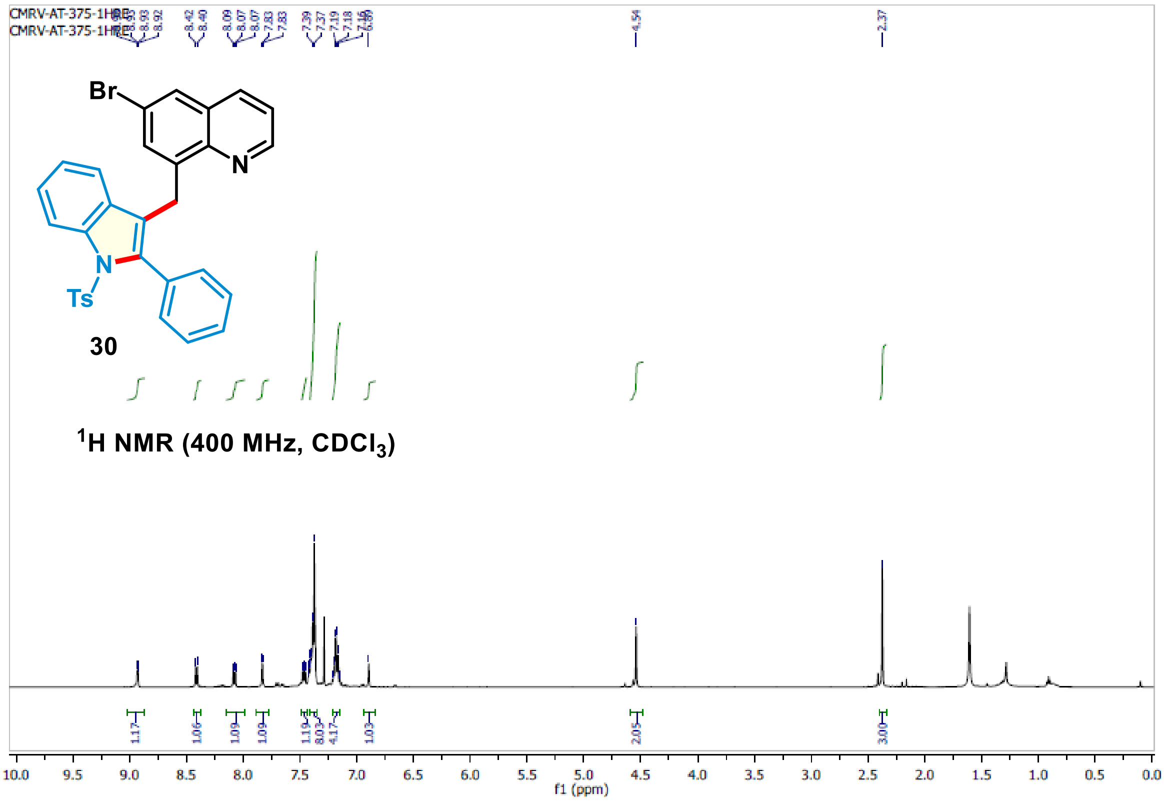
CMRV-AT-375-1HRE
Br
N
N
Ts
30
1H NMR (400 MHz, CDCl3)
8.42
8.40
8.09
8.07
8.07
7.83
7.83
7.39
7.37
7.19
7.18
7.16
4.54
2.37
1.17
1.06
1.09
1.09
1.19
8.03
4.17
1.03
2.05
3.00
f1 (ppm)

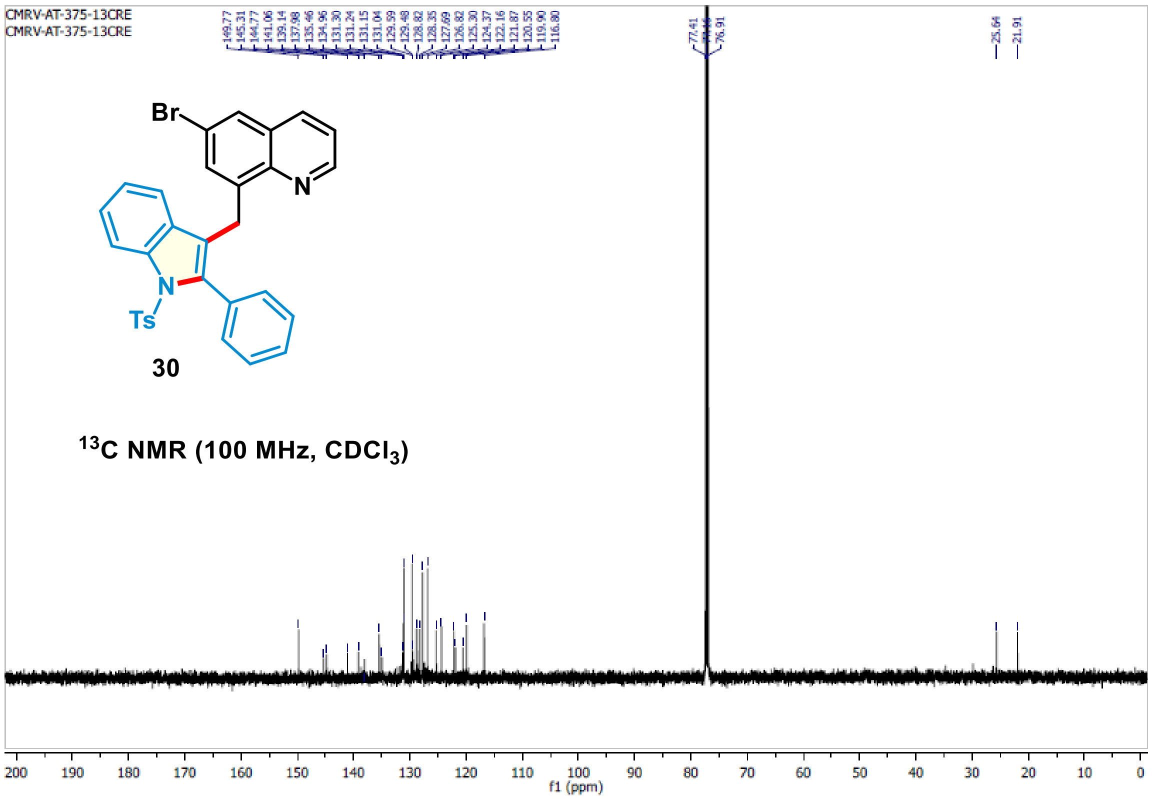
CMRV-AT-375-13CRE
CMRV-AT-375-13CRE
149.77
145.31
144.77
141.06
139.14
137.98
135.46
134.96
131.30
131.24
131.15
131.04
129.59
129.48
128.82
128.35
127.69
126.82
125.30
124.37
122.16
121.87
120.55
119.90
116.80
77.41
76.91
25.64
21.91
Br
N
N
Ts
30
13C NMR (100 MHz, CDCl3)
f1 (ppm)

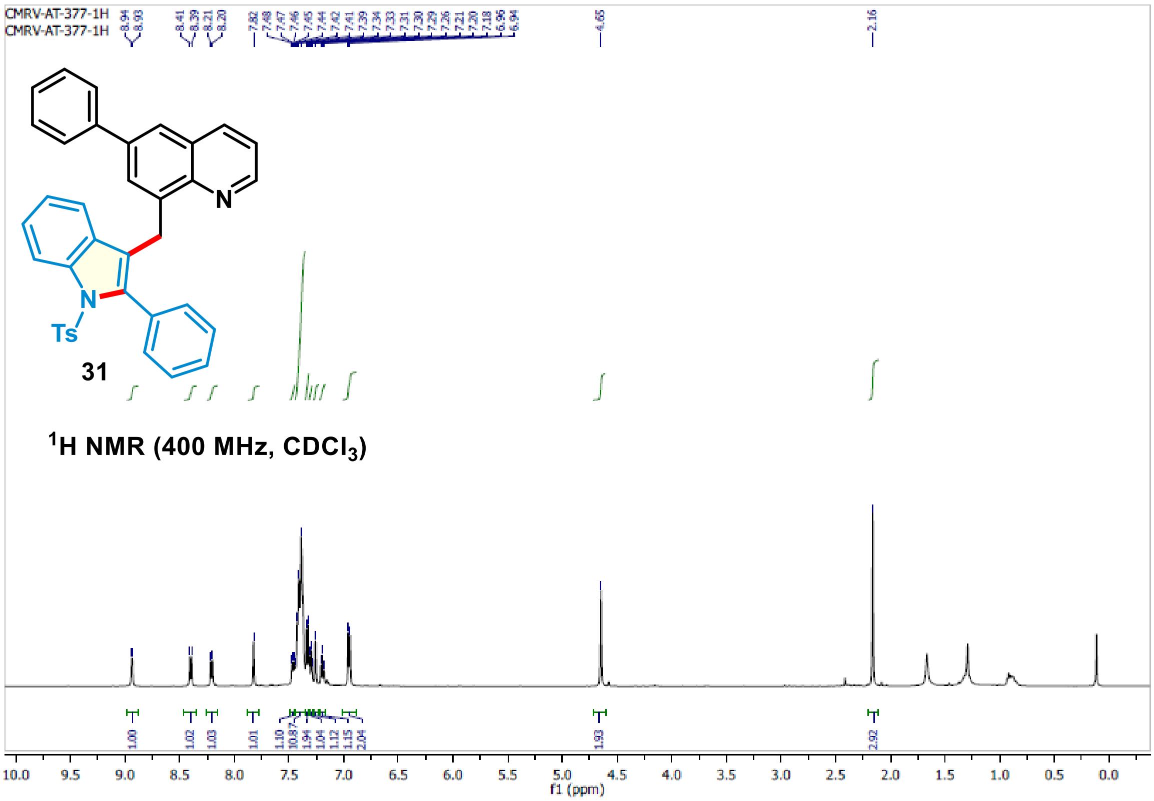
CMRV-AT-377-1H
CMRV-AT-377-1H
8.94
8.93
8.41
8.39
8.21
8.20
7.82
7.48
7.47
7.46
7.45
7.44
7.42
7.41
7.39
7.34
7.33
7.31
7.30
7.29
7.26
7.21
7.20
7.18
6.96
6.94
4.65
2.16
N
Ts
N
31
1H NMR (400 MHz, CDCl3)
1.00
1.02
1.03
1.01
1.10
10.87
1.94
1.04
1.12
1.15
2.04
1.93
2.92
10.0
9.5
9.0
8.5
8.0
7.5
7.0
6.5
6.0
5.5
5.0
4.5
4.0
3.5
3.0
2.5
2.0
1.5
1.0
0.5
0.0
f1 (ppm)

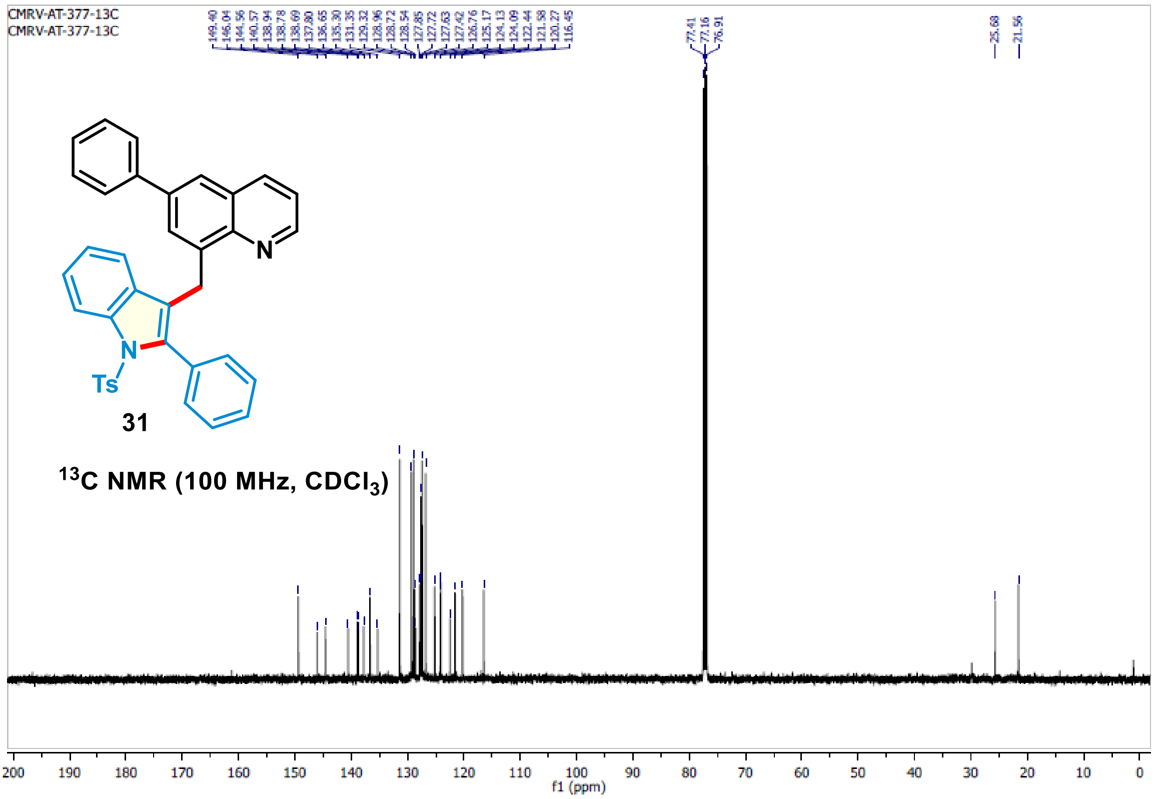
CMRV-AT-377-13C
CMRV-AT-377-13C
149.40
146.04
144.56
140.57
138.94
138.78
138.69
137.80
136.65
135.30
131.35
129.32
128.96
128.72
128.54
127.85
127.72
127.63
127.42
126.76
125.17
124.13
124.09
122.44
121.58
120.27
116.45
77.41
77.16
76.91
25.68
21.56
N
Ts
N
31
13C NMR (100 MHz, CDCl3)
200
190
180
170
160
150
140
130
120
110
100
90
80
70
60
50
40
30
20
10
0
f1 (ppm)

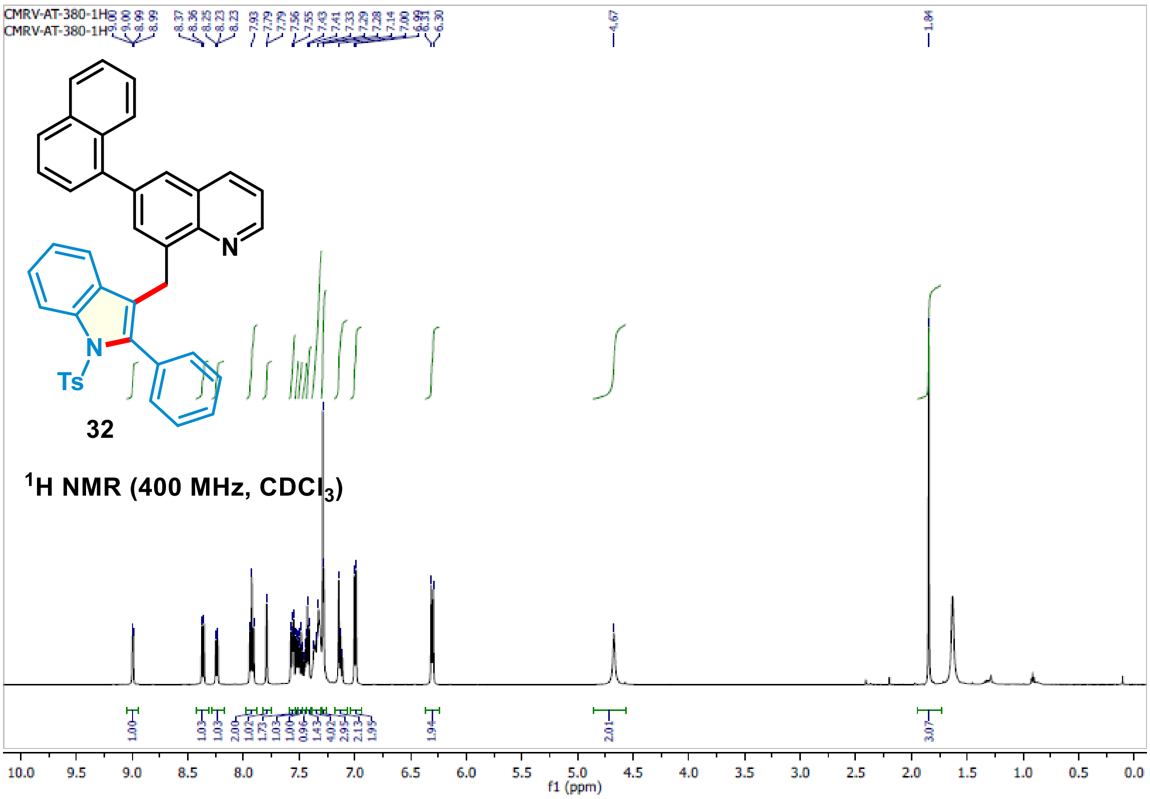
CMRV-AT-380-1H
CMRV-AT-380-1H
9.00 9.00 8.99 8.99 8.37 8.36 8.25 8.23 8.23 7.93 7.79 7.79 7.56 7.55 7.43 7.41 7.33 7.29 7.28 7.14 7.00 6.99 6.31 6.30
4.67
1.84
N
Ts
32
1H NMR (400 MHz, CDCl3)
1.00 1.03 1.03 2.00 1.02 1.73 1.03 1.00 0.96 1.43 4.02 2.95 2.13 1.93 1.94 2.01 3.07
10.0 9.5 9.0 8.5 8.0 7.5 7.0 6.5 6.0 5.5 5.0 4.5 4.0 3.5 3.0 2.5 2.0 1.5 1.0 0.5 0.0
f1 (ppm)

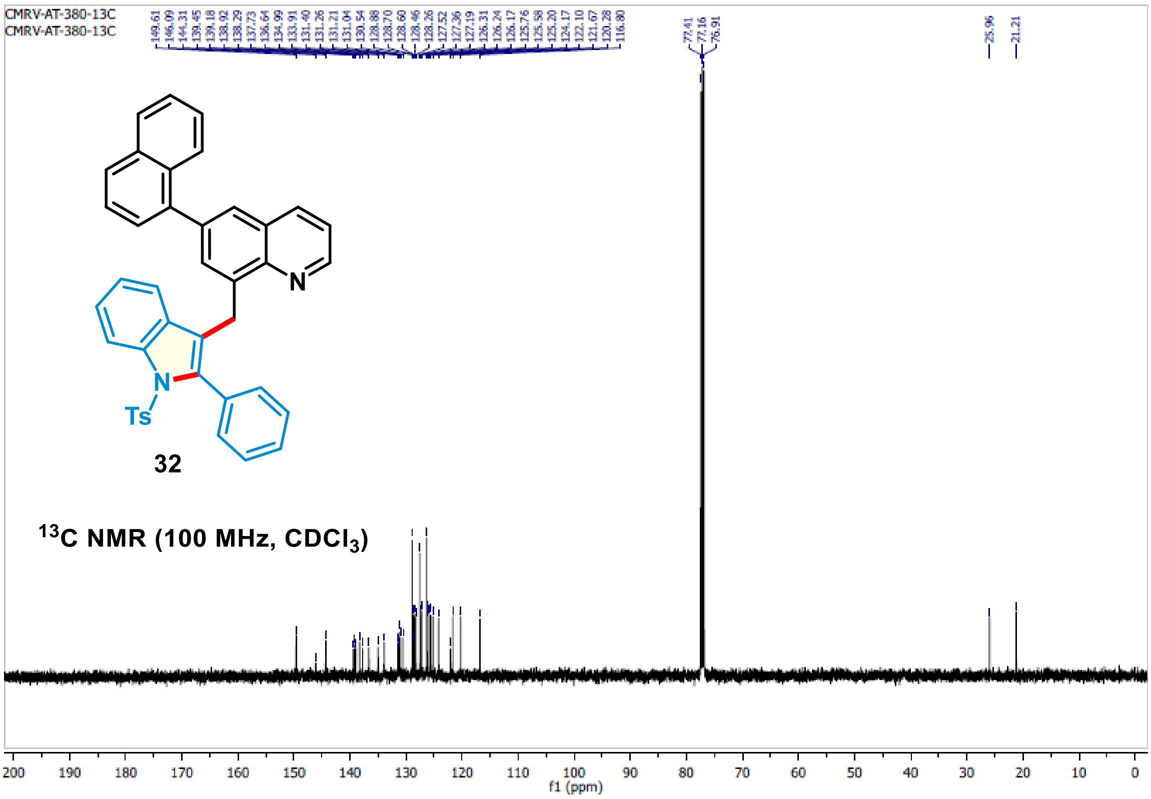
CMRV-AT-380-13C
CMRV-AT-380-13C
149.61 146.09 144.31 139.45 139.18 138.92 138.29 137.73 136.64 134.99 133.91 131.40 131.26 131.21 131.04 130.54 128.88 128.70 128.60 128.46 128.26 127.52 127.36 127.19 126.31 126.24 126.17 125.76 125.58 125.20 124.17 122.10 121.67 120.28 116.80
77.41 77.16 76.91
25.96
21.21
N
N
Ts
32
13C NMR (100 MHz, CDCl3)
200 190 180 170 160 150 140 130 120 110 100 90 80 70 60 50 40 30 20 10 0
f1 (ppm)

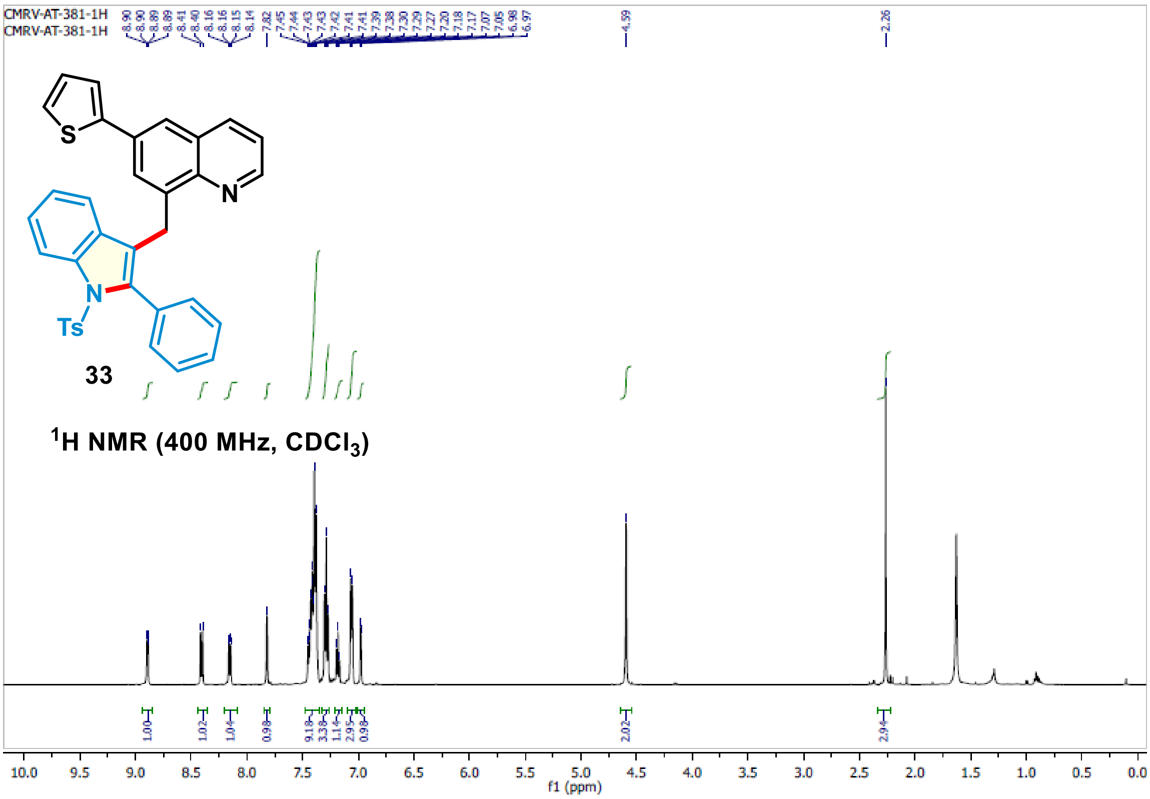
CMRV-AT-381-1H
CMRV-AT-381-1H
33
^{1}H NMR (400 MHz, CDCl$_3$)
f1 (ppm)

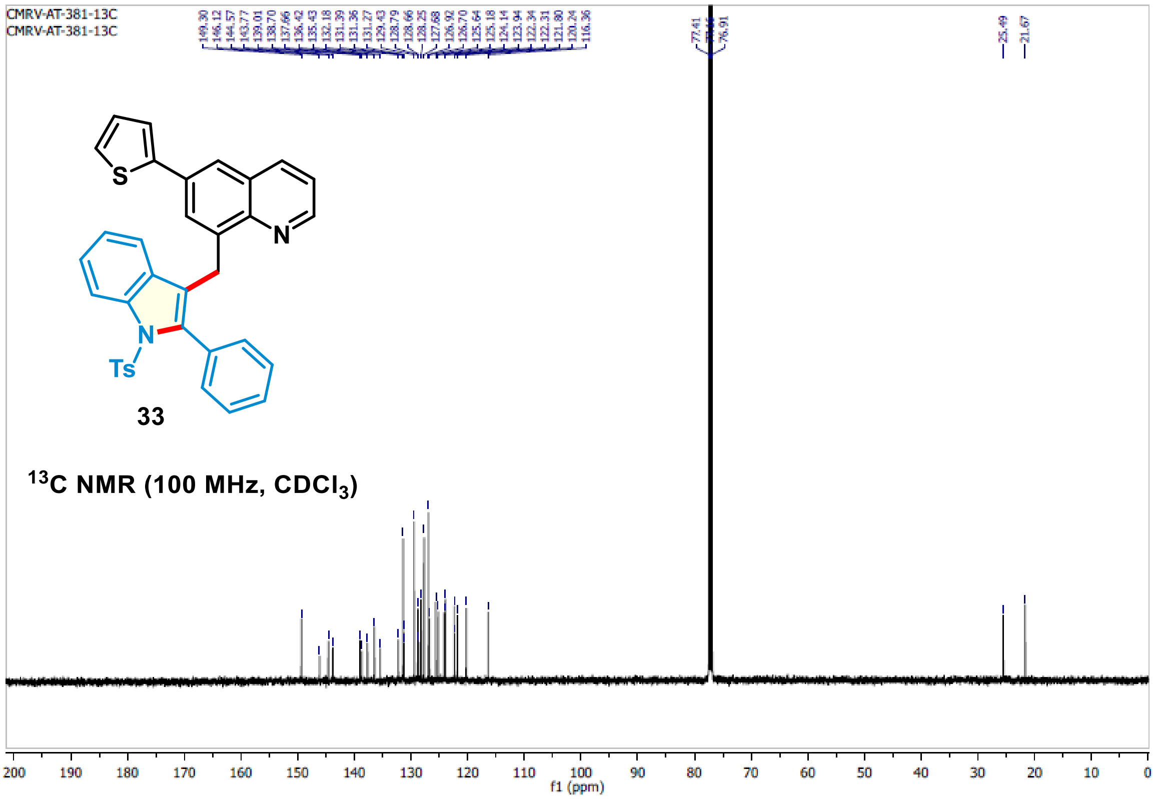
CMRV-AT-381-13C
CMRV-AT-381-13C
33
^{13}C NMR (100 MHz, CDCl$_3$)
f1 (ppm)

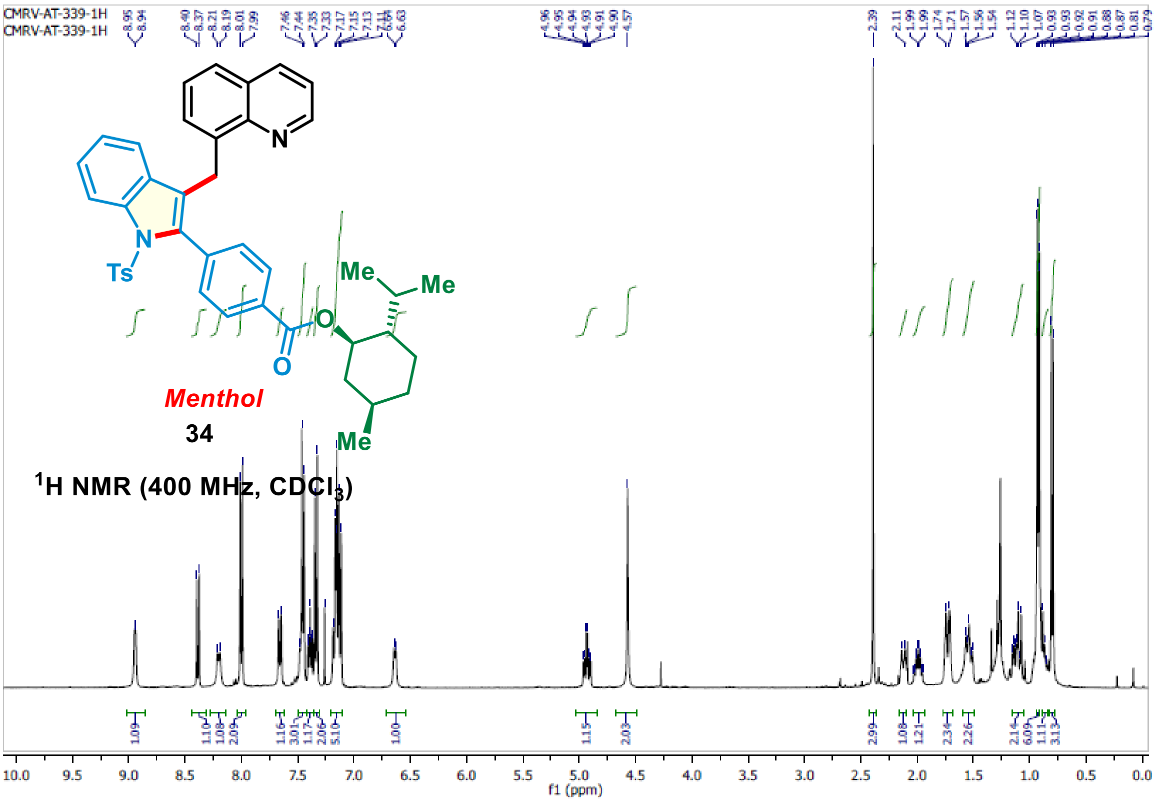
CMRV-AT-339-1H
CMRV-AT-339-1H
8.95 8.94 8.40 8.37 8.21 8.19 8.01 7.99 7.46 7.44 7.35 7.33 7.17 7.15 7.13 7.11 6.64 6.63 4.96 4.95 4.94 4.93 4.91 4.90 4.57 2.39 2.11 1.99 1.99 1.74 1.71 1.57 1.56 1.54 1.12 1.10 1.07 0.93 0.93 0.92 0.91 0.88 0.87 0.81 0.79
N
N
Ts
Me
Me
O
O
Me
Menthol
34
1H NMR (400 MHz, CDCl3)
1.09 1.10 1.08 2.09 1.16 3.01 1.17 2.06 5.10 1.00 1.15 2.03 2.99 1.08 1.21 2.34 2.26 2.16 6.09 1.11 3.13
f1 (ppm)

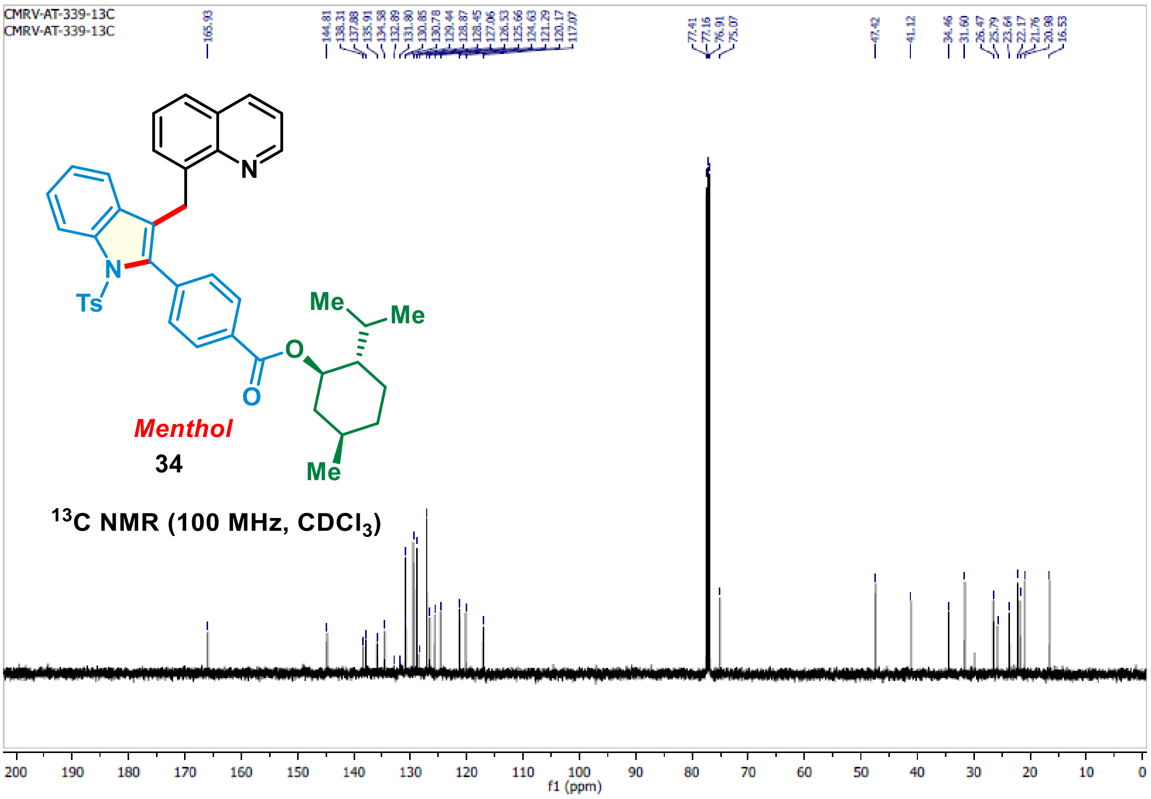
CMRV-AT-339-13C
CMRV-AT-339-13C
165.93 144.81 138.31 137.88 135.91 134.58 132.89 131.80 130.85 130.78 129.44 128.87 128.45 127.06 126.53 125.66 124.63 121.29 120.17 117.07 77.41 77.16 76.91 75.07 47.42 41.12 34.46 31.60 26.47 25.79 23.64 22.17 21.76 20.98 16.53
N
N
Ts
Me
Me
O
O
Me
Menthol
34
13C NMR (100 MHz, CDCl3)
f1 (ppm)

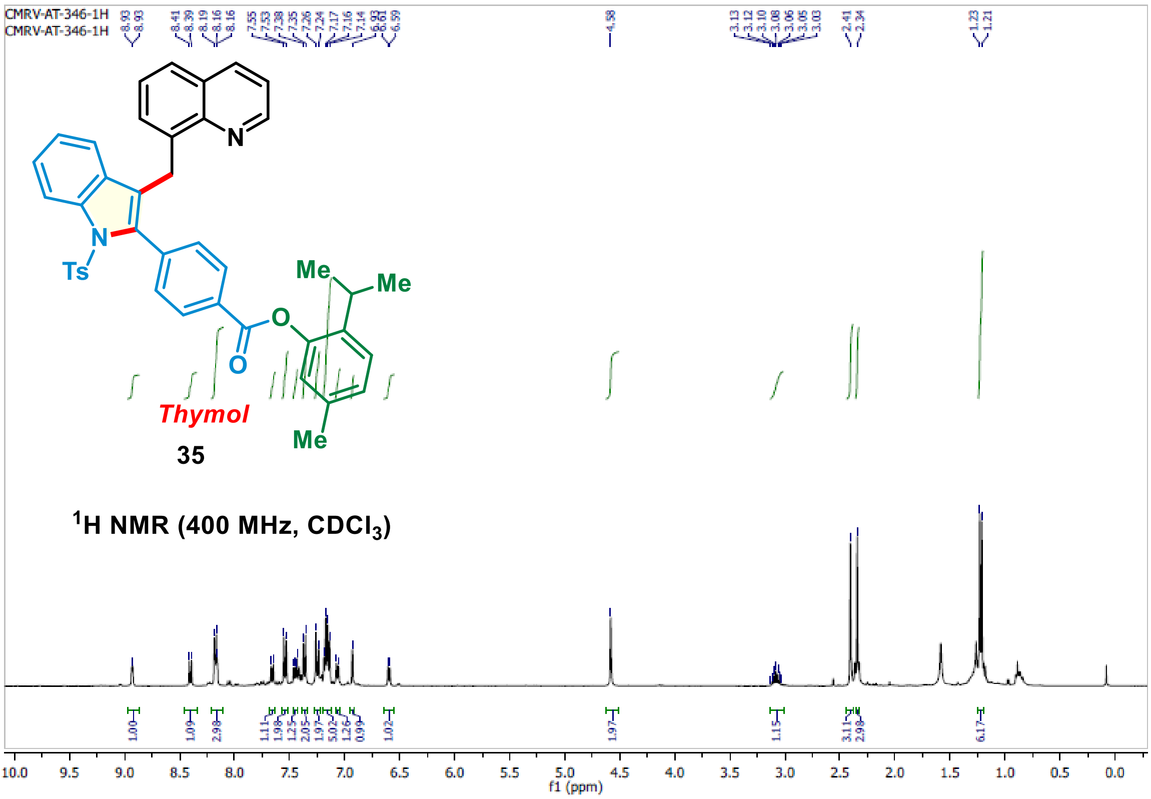
CMRV-AT-346-1H
CMRV-AT-346-1H
N
Ts
Me
Me
O
O
N
Thymol
Me
35
1H NMR (400 MHz, CDCl3)
f1 (ppm)

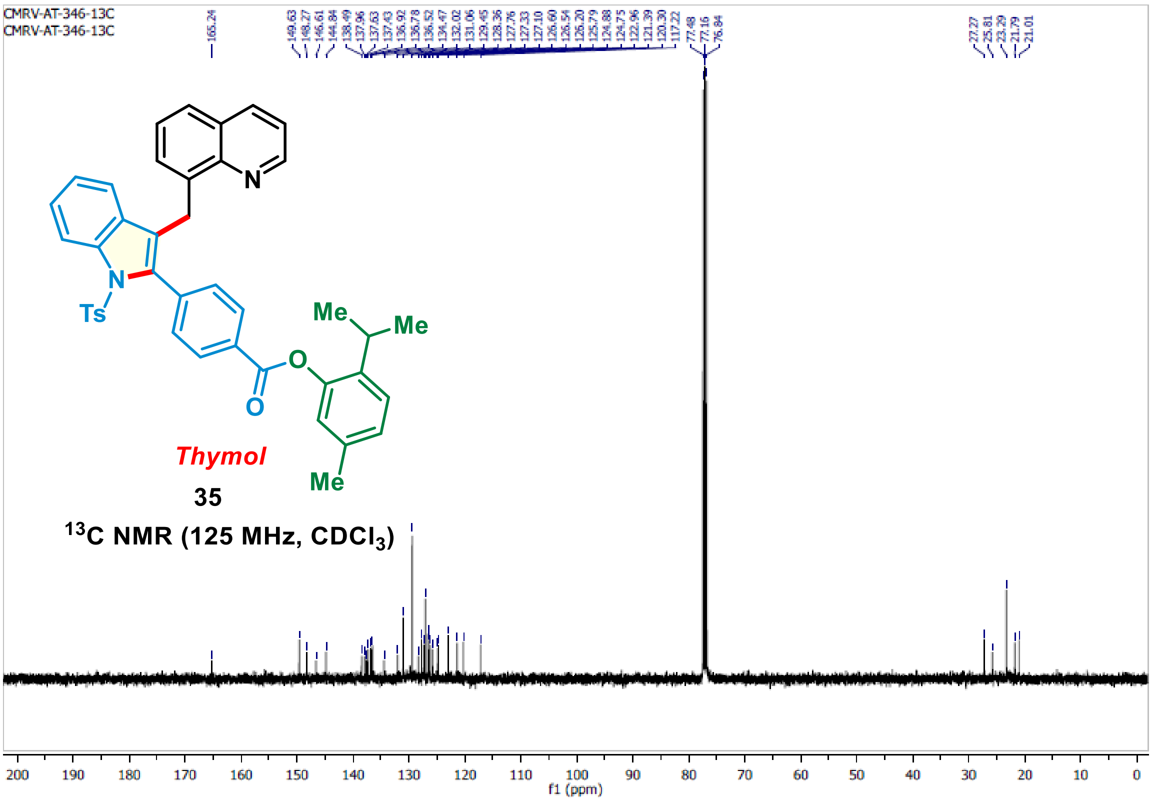
CMRV-AT-346-13C
CMRV-AT-346-13C
N
Ts
Me
Me
O
O
N
Thymol
Me
35
13C NMR (125 MHz, CDCl3)
f1 (ppm)

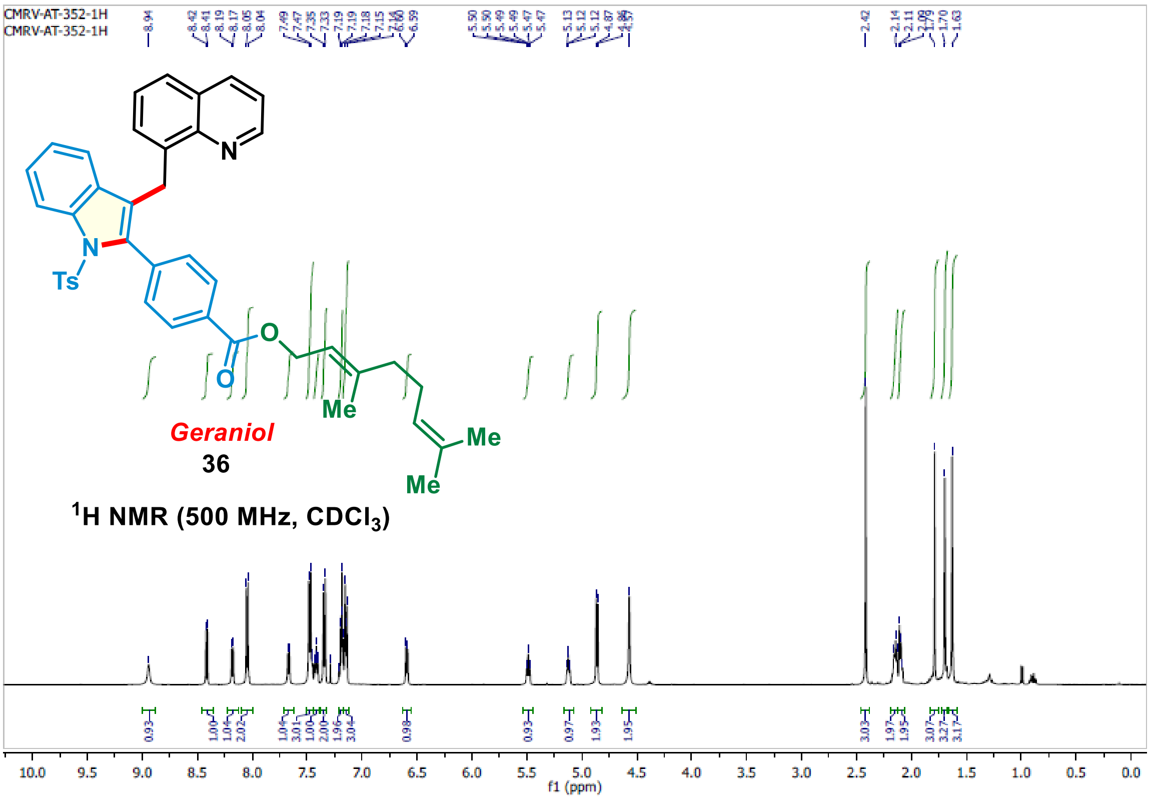
CMRV-AT-352-1H
Geraniol
36
^{1}H NMR (500 MHz, CDCl$_3$)
f1 (ppm)

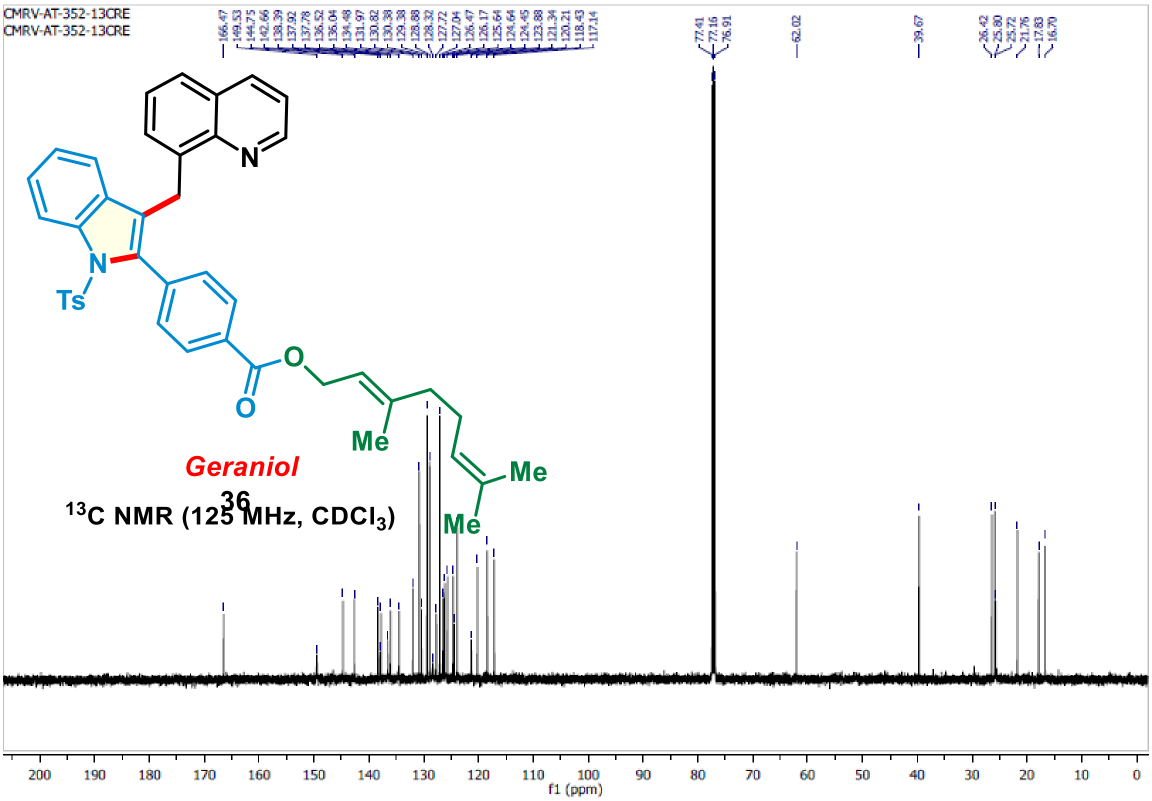
CMRV-AT-352-13CRE
Geraniol
36
^{13}C NMR (125 MHz, CDCl$_3$)
f1 (ppm)

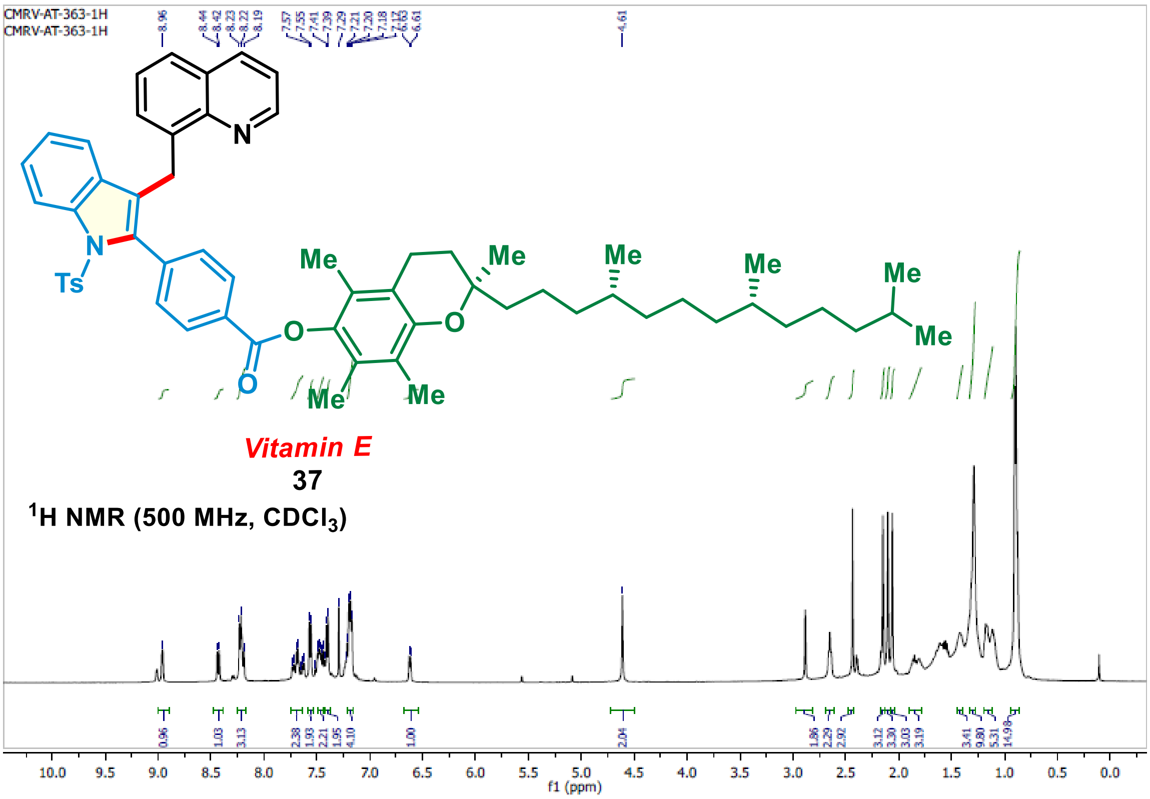
CMRV-AT-363-1H
CMRV-AT-363-1H
N
N
Ts
Me
Me
Me
Me
Me
Me
O
O
O
Me
Me
Vitamin E
37
^{1}H NMR (500 MHz, CDCl$_3$)
f1 (ppm)

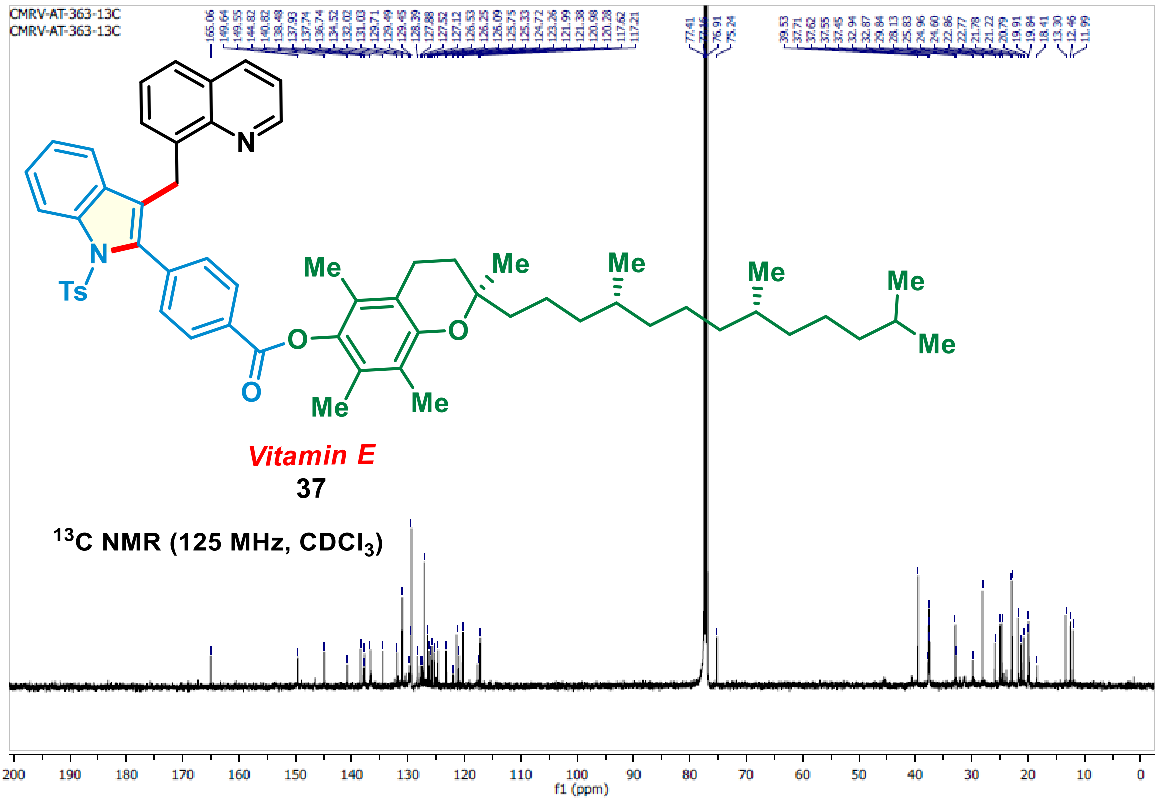
CMRV-AT-363-13C
CMRV-AT-363-13C
N
N
Ts
Me
Me
Me
Me
Me
Me
O
O
O
Me
Me
Vitamin E
37
^{13}C NMR (125 MHz, CDCl$_3$)
f1 (ppm)

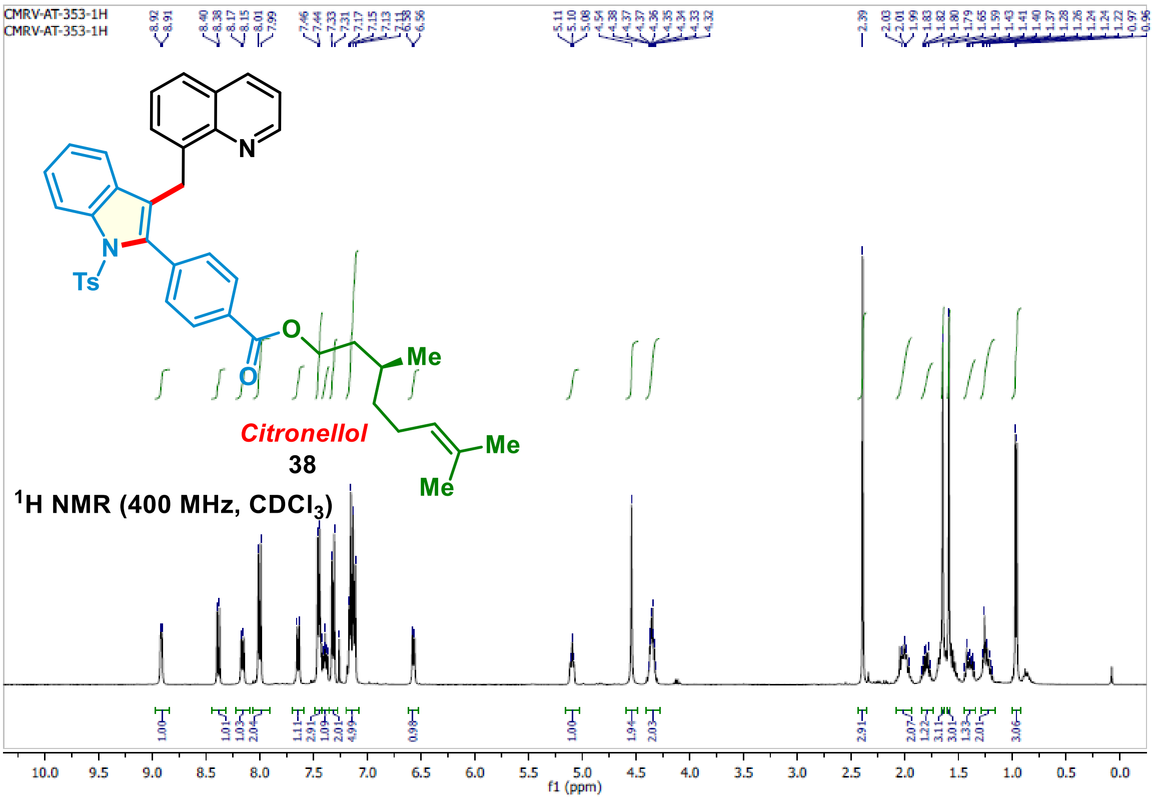
CMRV-AT-353-1H
Citronellol
38
1H NMR (400 MHz, CDCl3)
f1 (ppm)

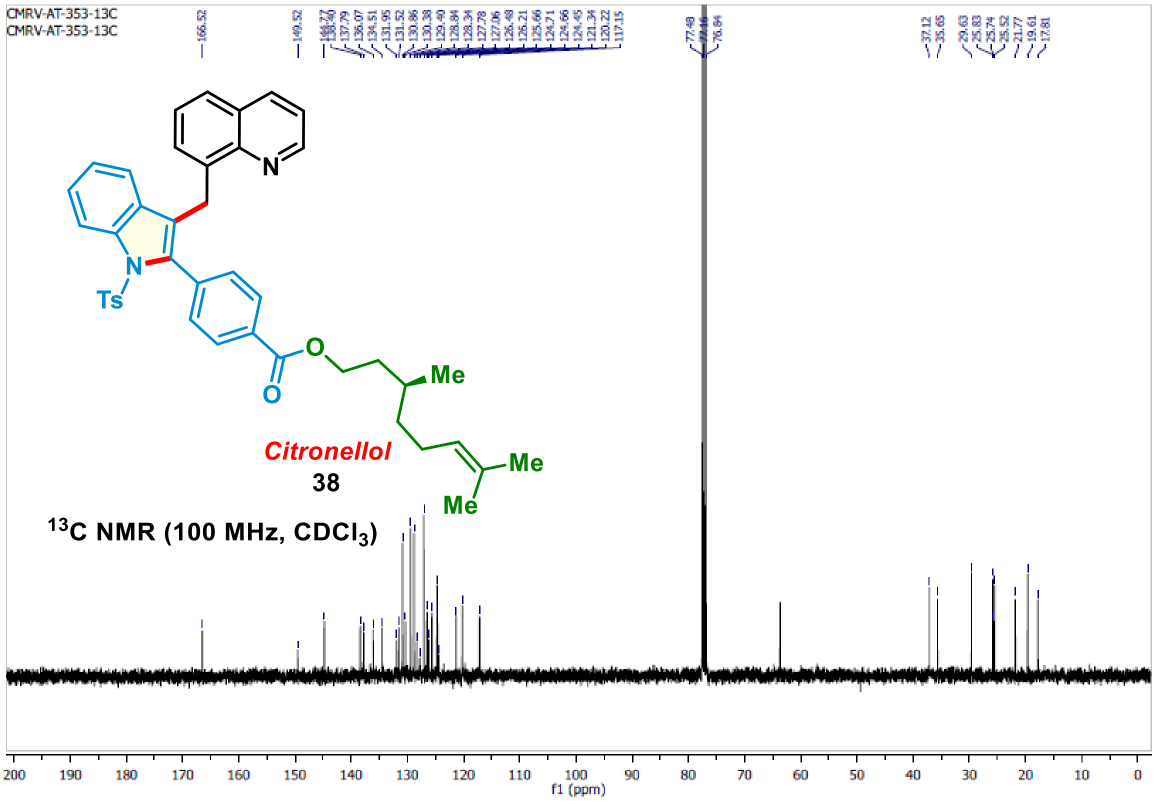
CMRV-AT-353-13C
Citronellol
38
13C NMR (100 MHz, CDCl3)
f1 (ppm)

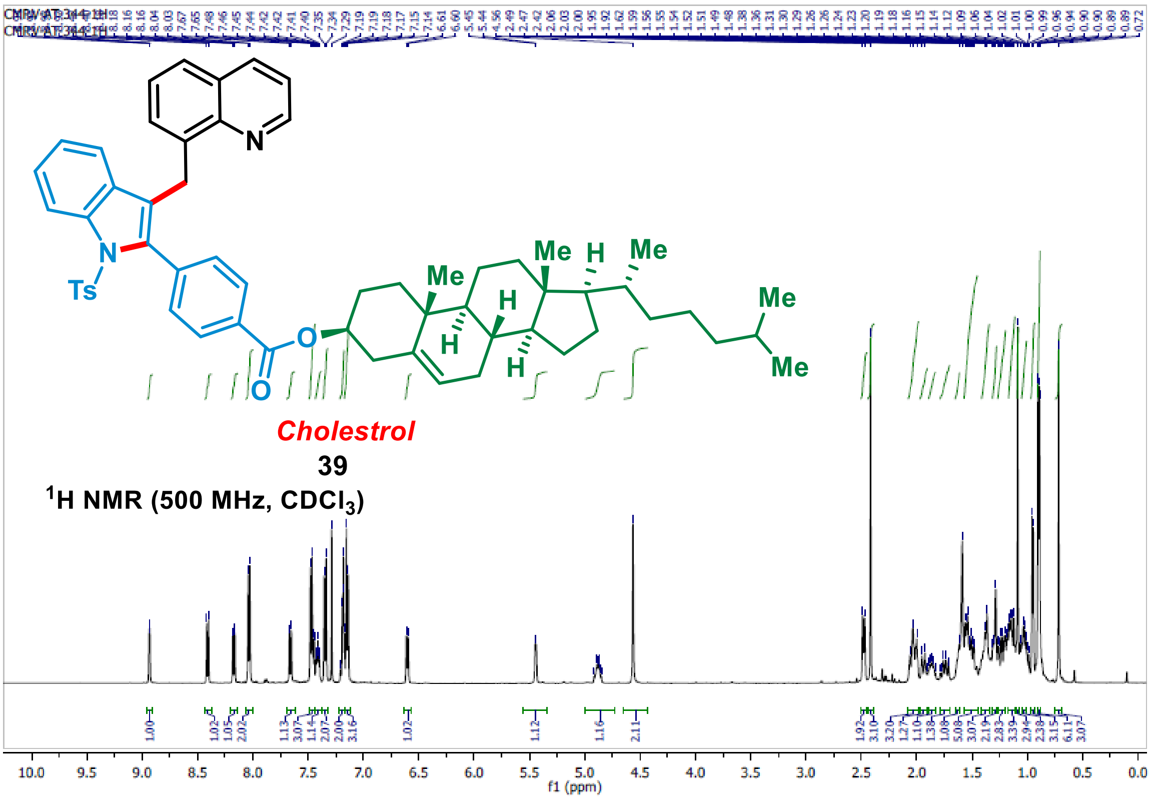
N
N
Ts
O
O
Me
Me
H
Me
H
H
H
Me
Me
Cholestrol
39
1H NMR (500 MHz, CDCl3)
f1 (ppm)

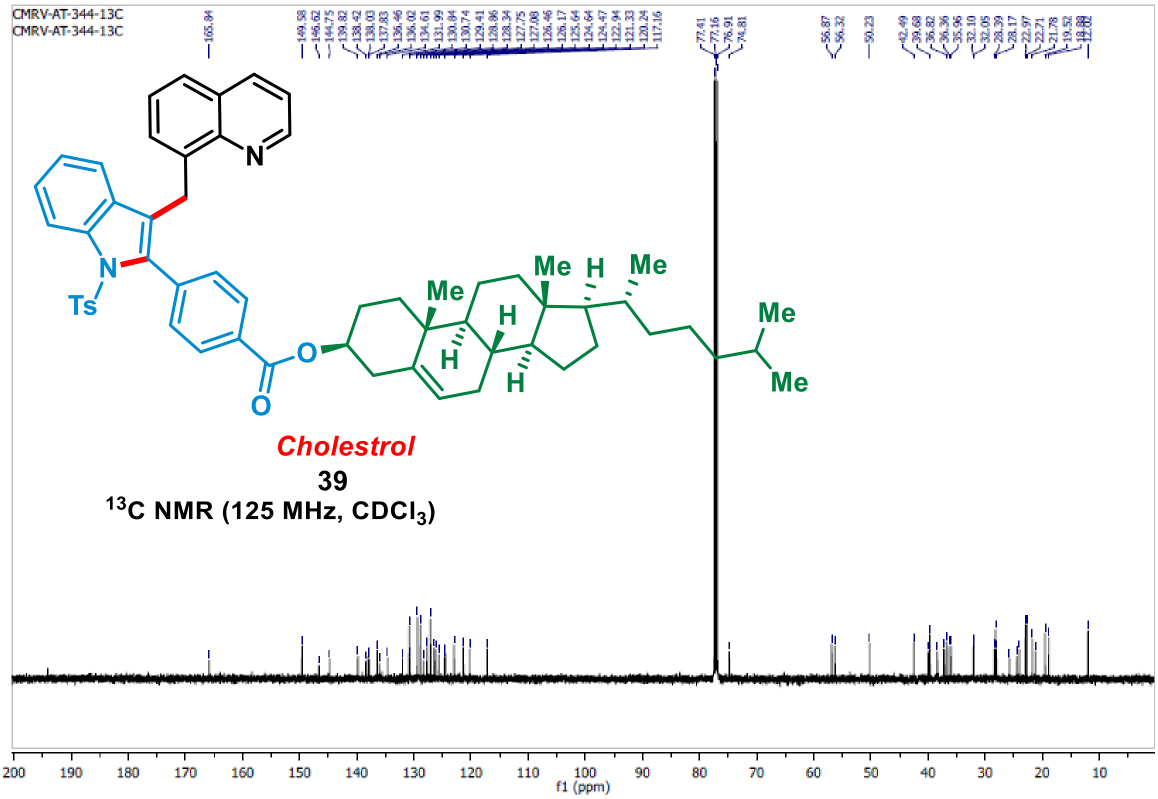
CMRV-AT-344-13C
CMRV-AT-344-13C
165.84
149.58
146.62
144.75
N
N
Ts
O
O
Me
Me
H
Me
H
H
H
Me
Me
Cholestrol
39
13C NMR (125 MHz, CDCl3)
f1 (ppm)

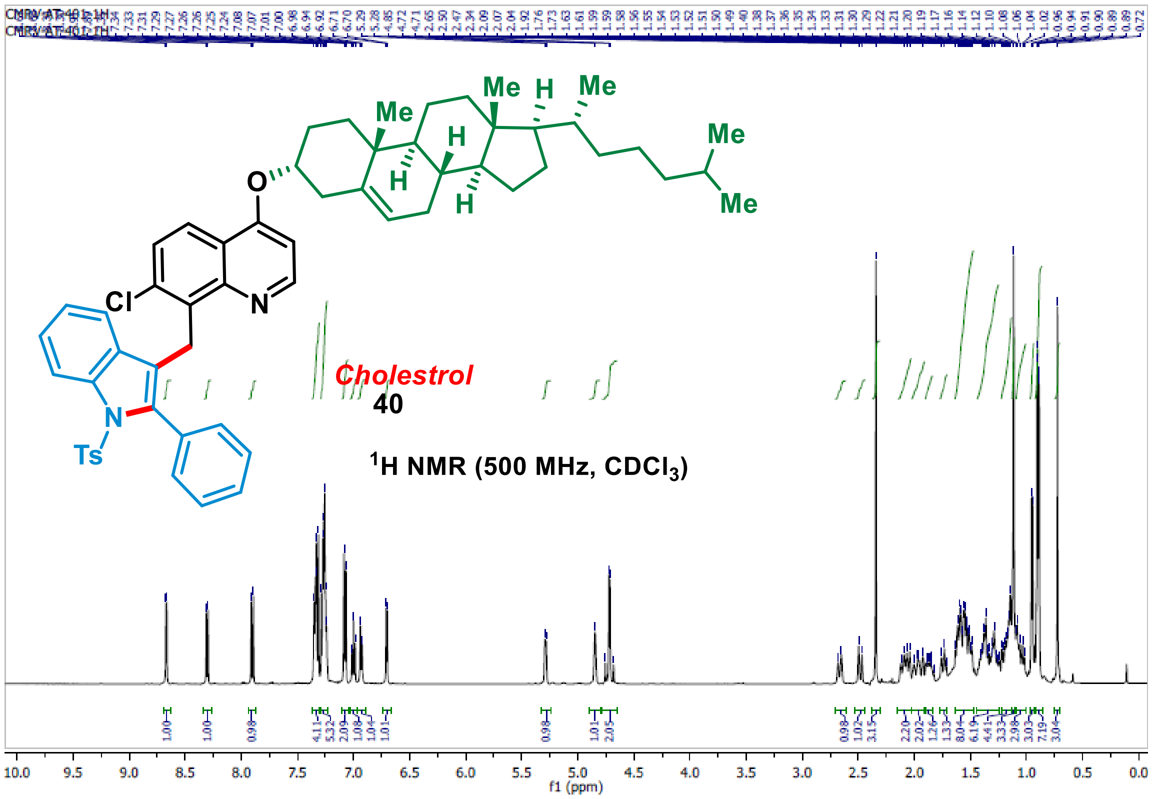

Cholestrol
40
^{1}H NMR (500 MHz, CDCl$_3$)
f1 (ppm)

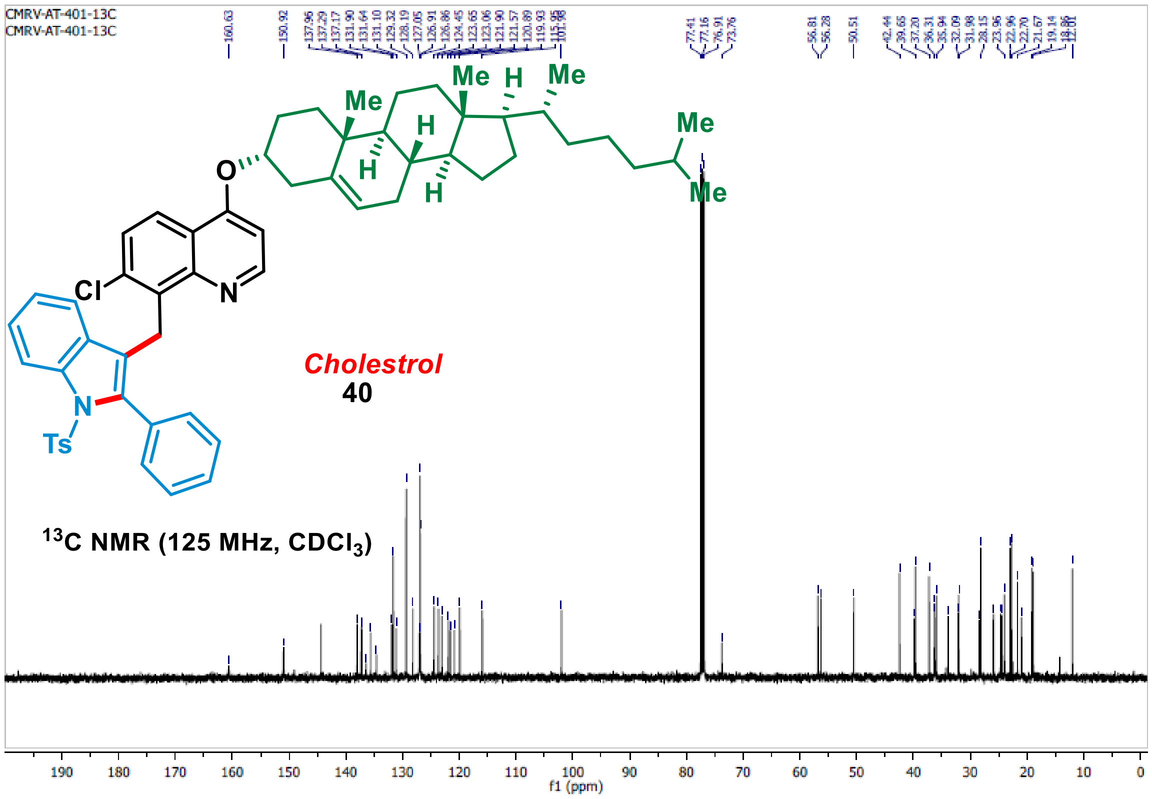

Cholestrol
40
^{13}C NMR (125 MHz, CDCl$_3$)
f1 (ppm)

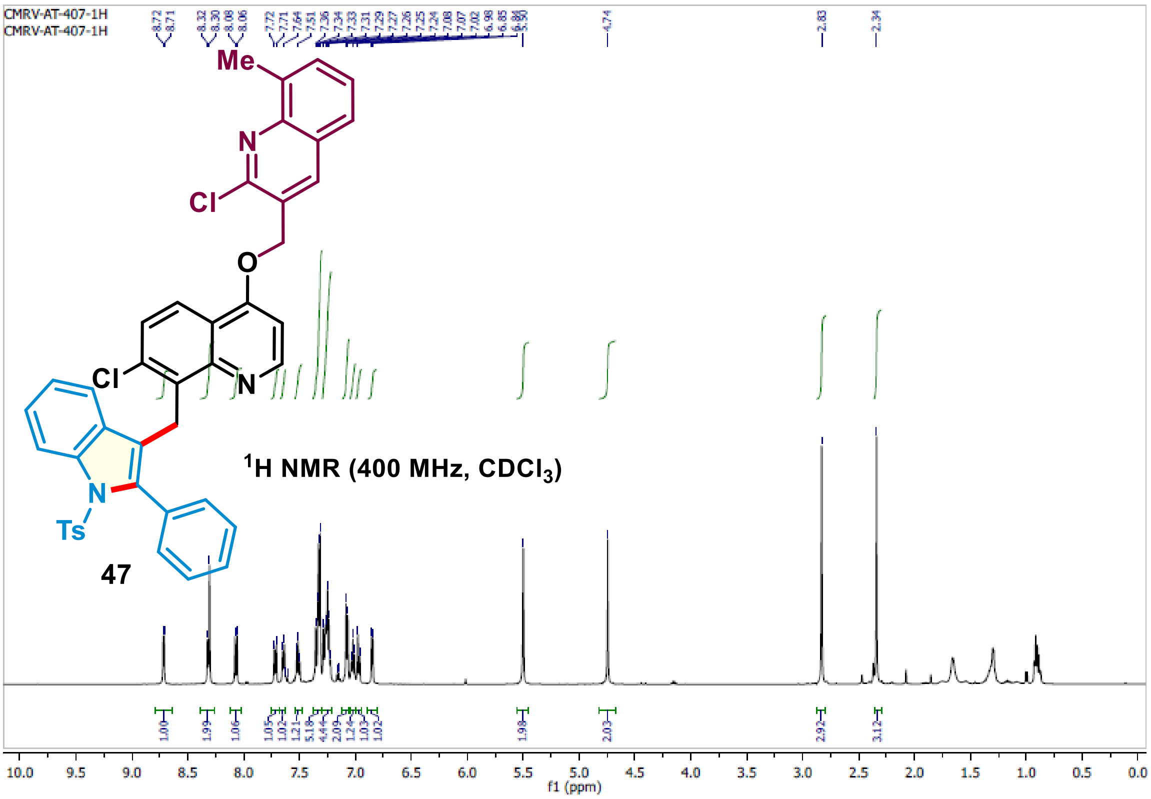
CMRV-AT-407-1H
Me
N
Cl
O
Cl
N
N
Ts
47
1H NMR (400 MHz, CDCl3)
f1 (ppm)

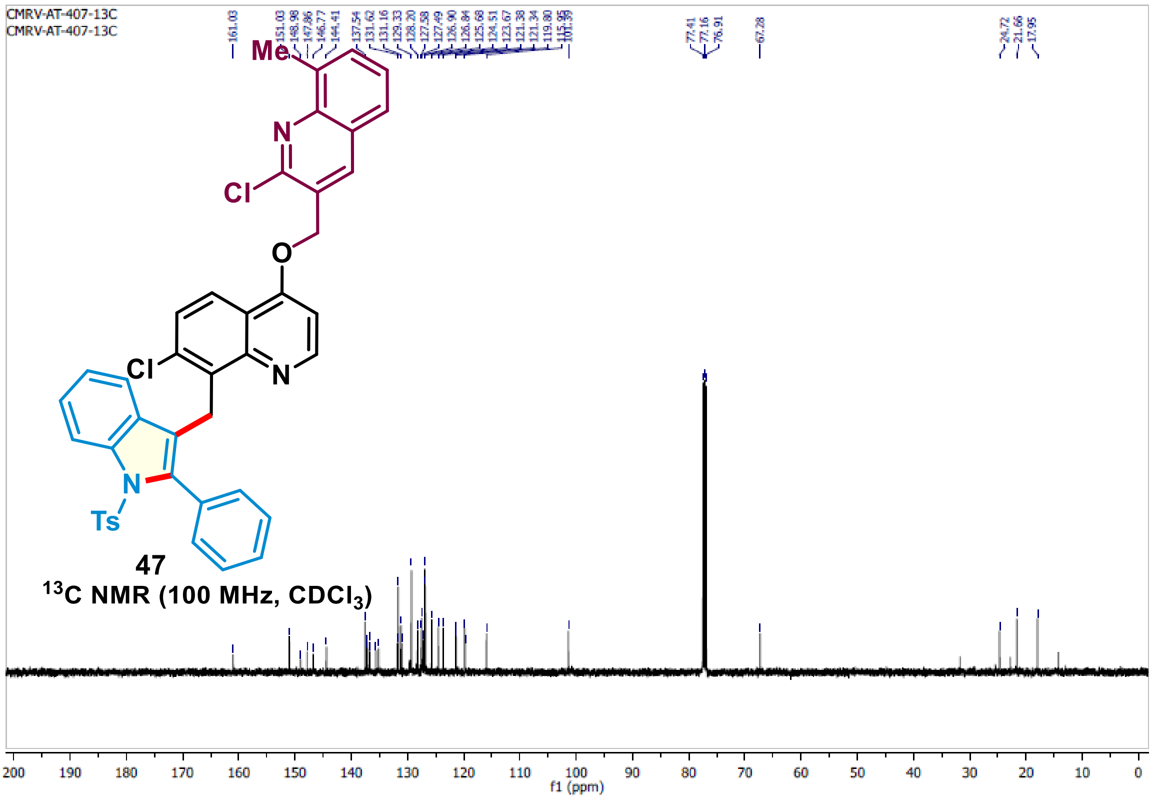
CMRV-AT-407-13C
Me
N
Cl
O
Cl
N
N
Ts
47
13C NMR (100 MHz, CDCl3)
f1 (ppm)

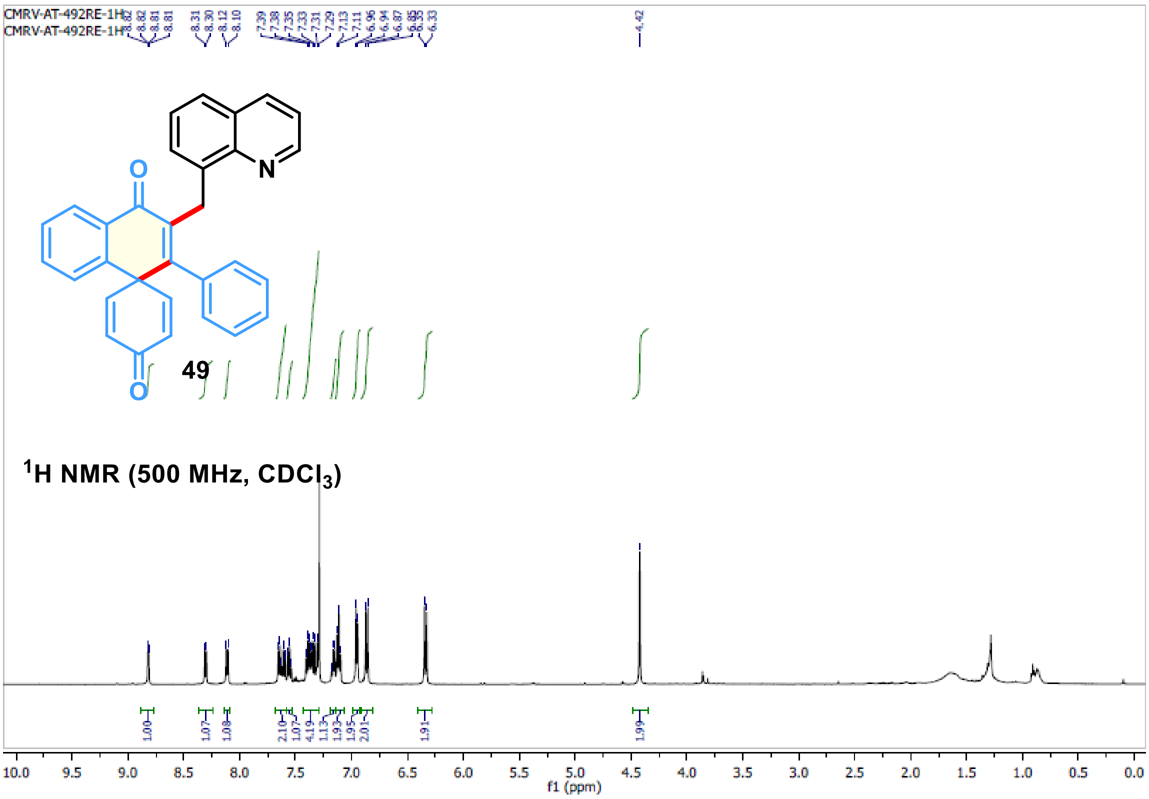
CMRV-AT-492RE-1H
49
1H NMR (500 MHz, CDCl3)
f1 (ppm)

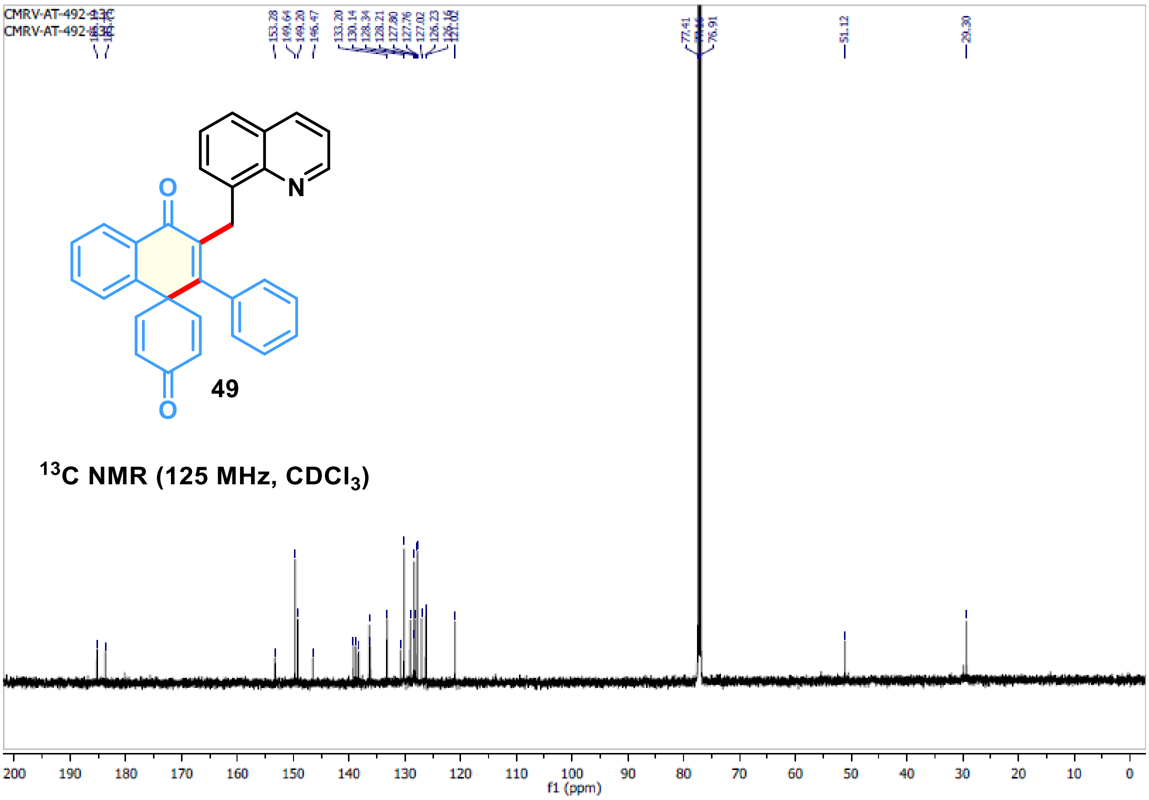
CMRV-AT-492-13C
49
13C NMR (125 MHz, CDCl3)
f1 (ppm)

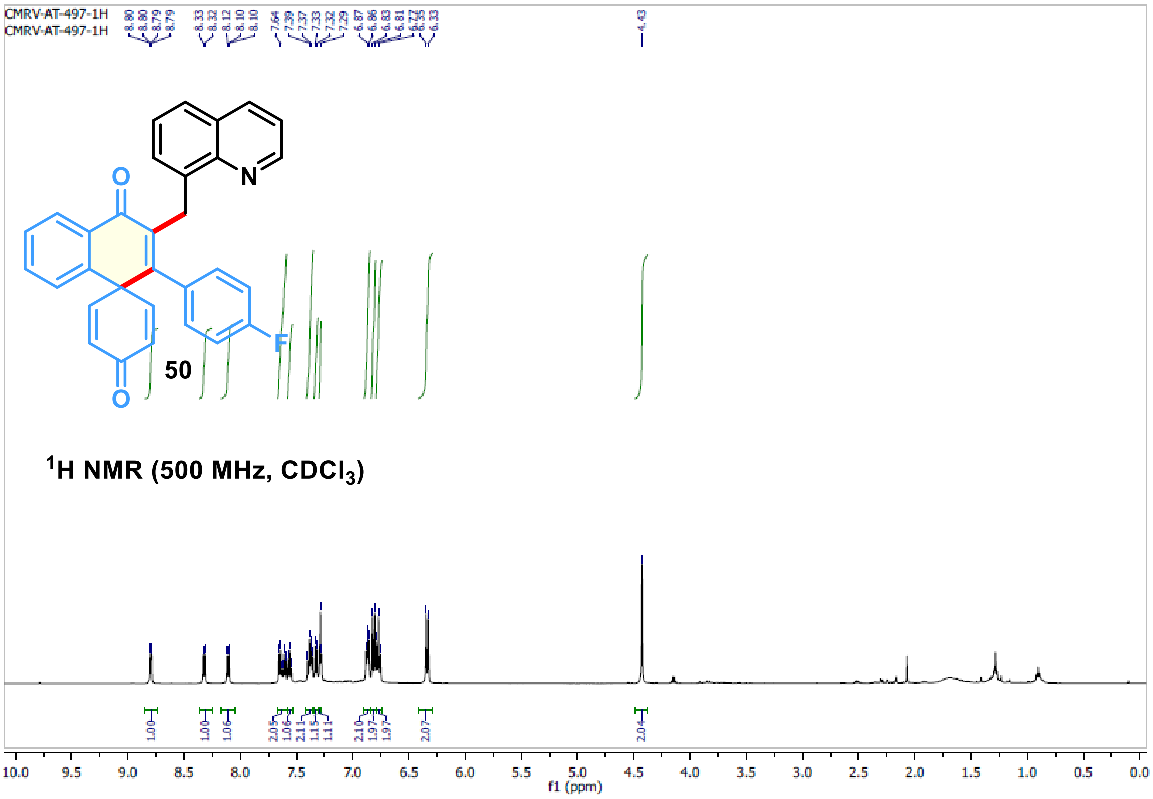
CMRV-AT-497-1H
50
1H NMR (500 MHz, CDCl3)
f1 (ppm)

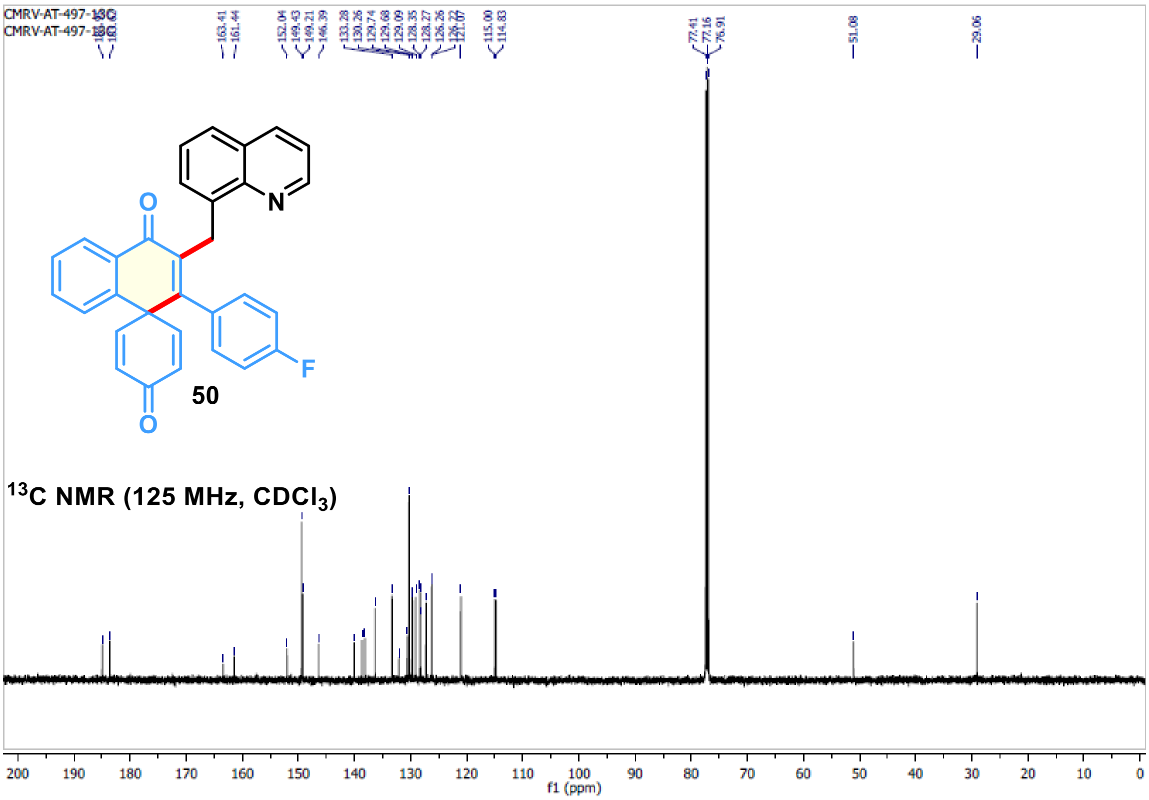
50
13C NMR (125 MHz, CDCl3)
f1 (ppm)

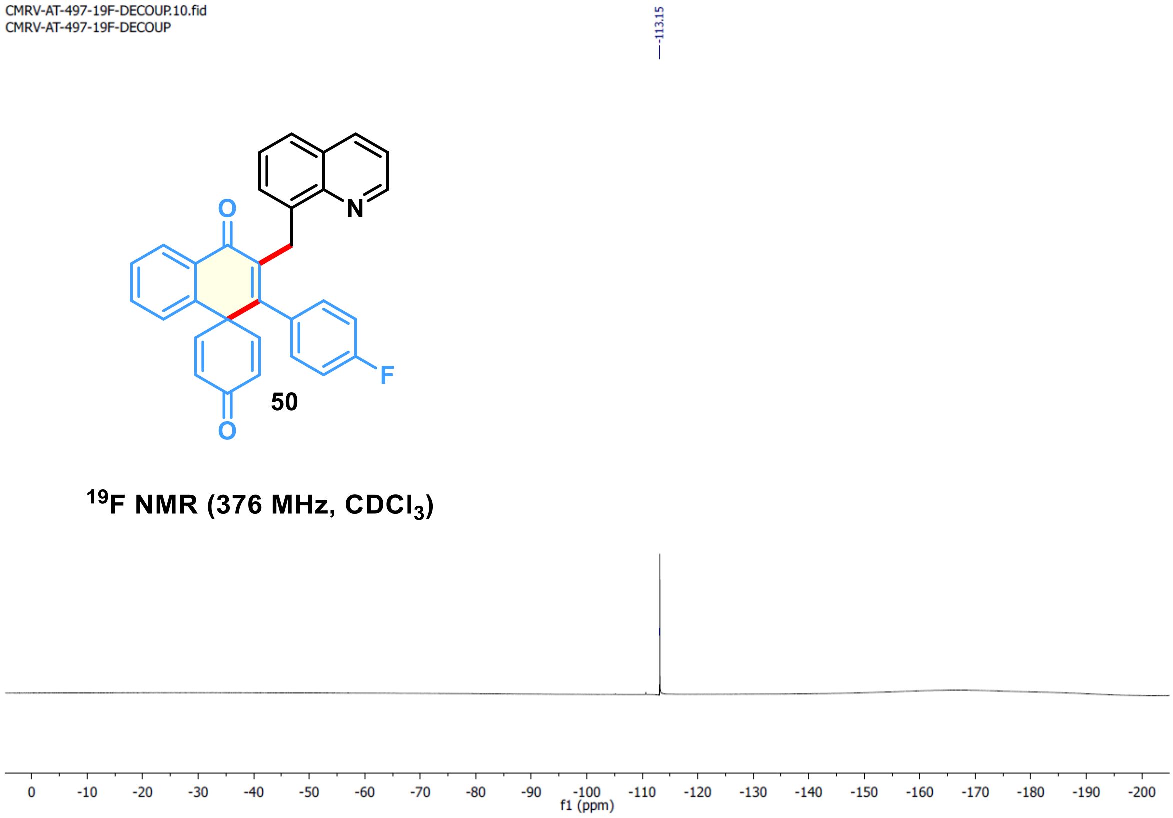
CMRV-AT-497-19F-DECOUP.10.fid
CMRV-AT-497-19F-DECOUP
-113.15
50
^{19}F NMR (376 MHz, CDCl$_3$)
f1 (ppm)

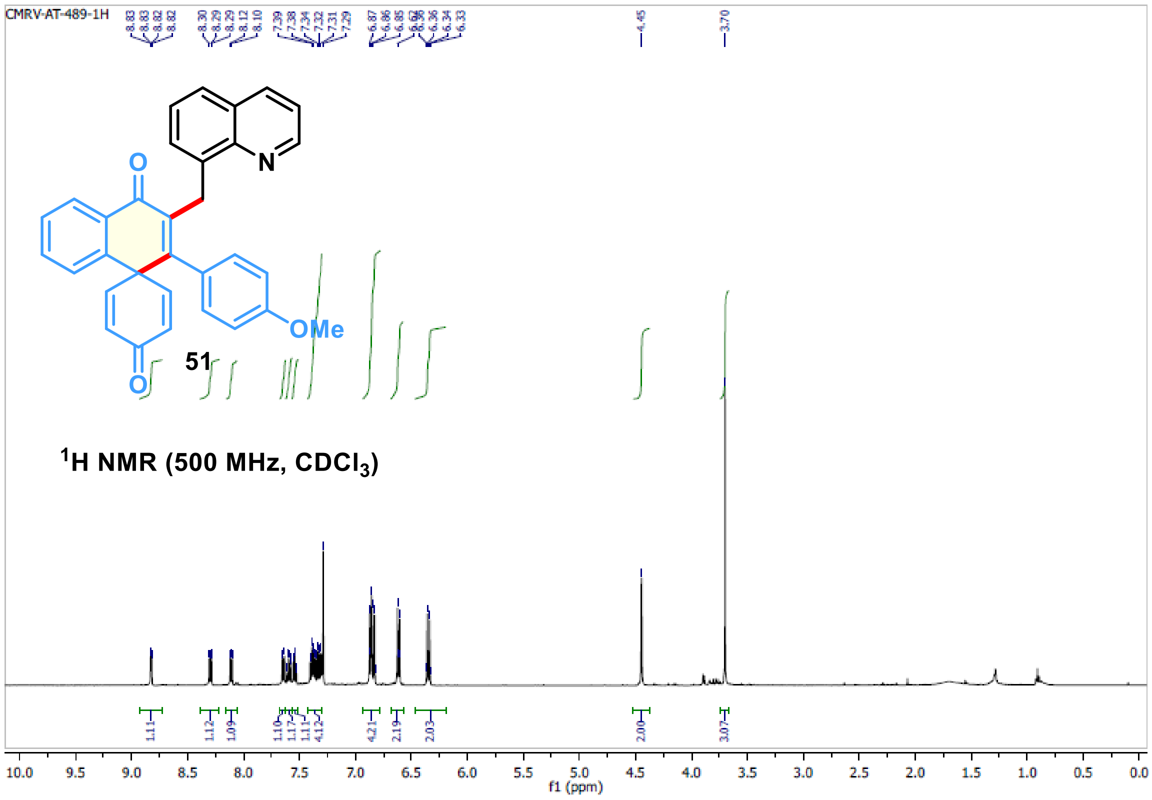
CMRV-AT-489-1H
4.45
3.70
51
^{1}H NMR (500 MHz, CDCl$_3$)
1.11
1.12
1.09
1.10
1.17
1.11
4.12
4.21
2.19
2.03
2.00
3.07
f1 (ppm)

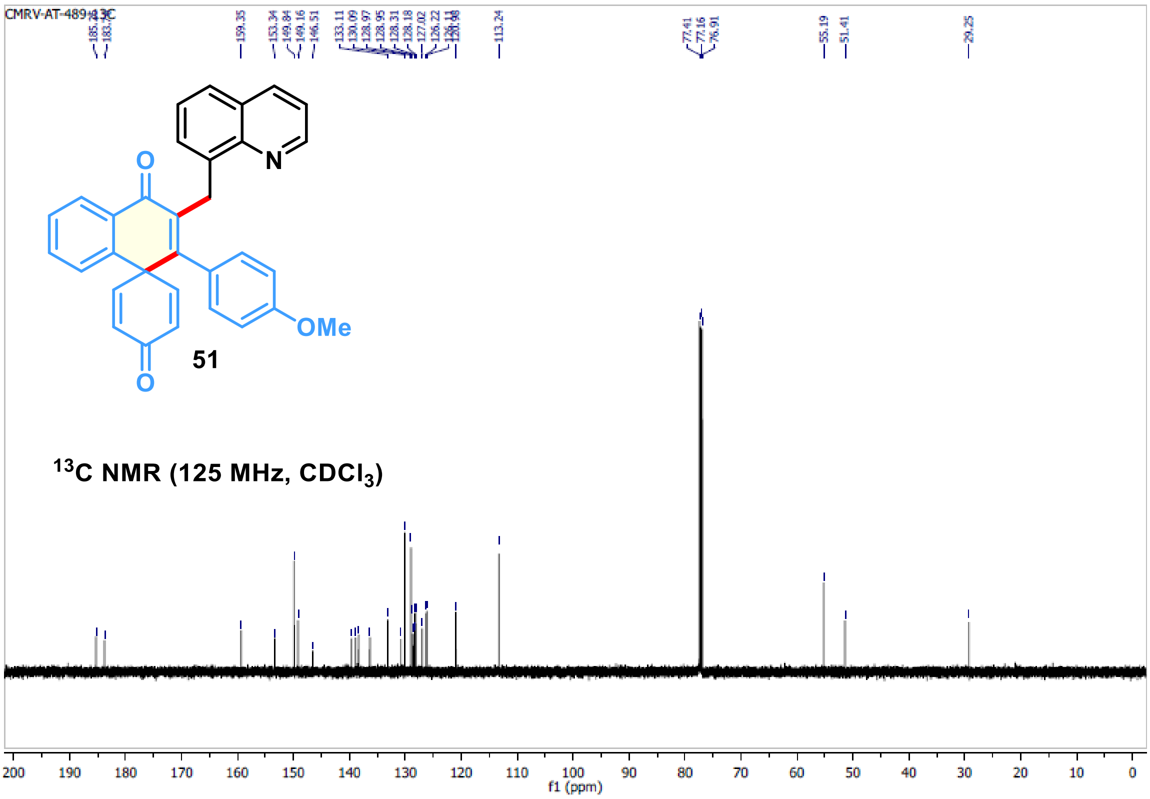

^{13}C NMR (125 MHz, $CDCl_3$)

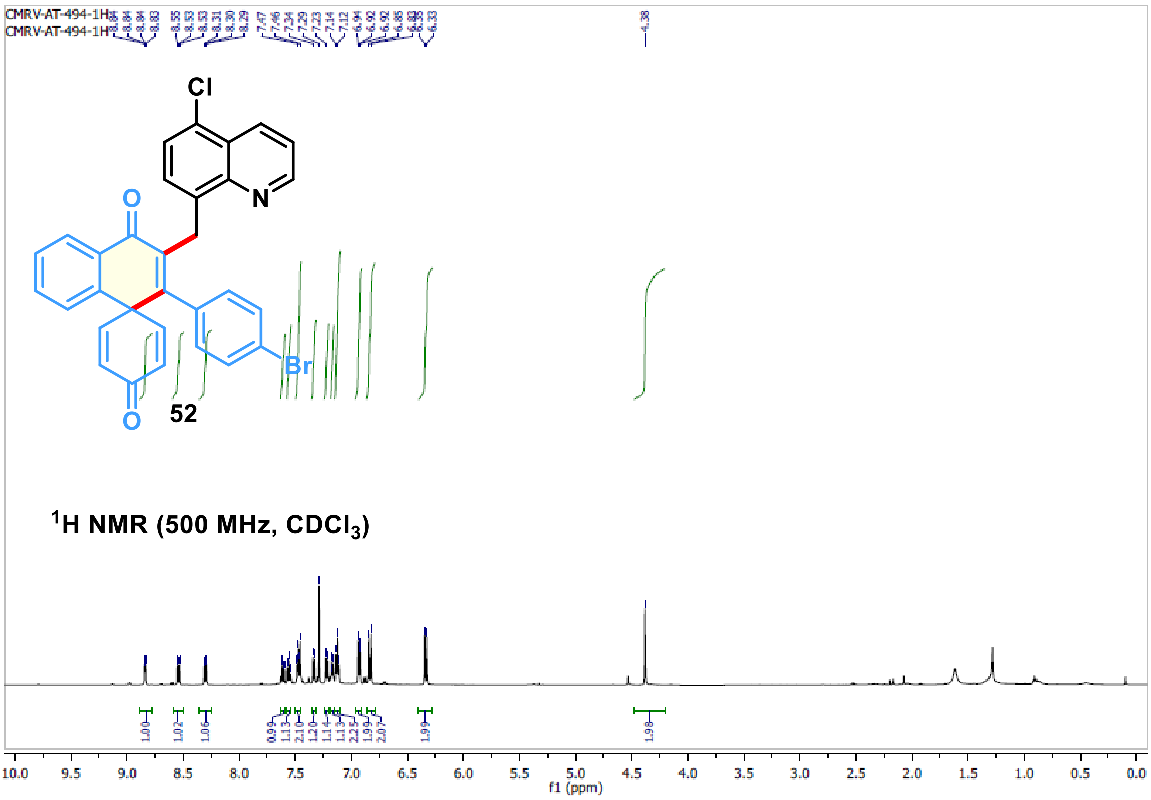

^{1}H NMR (500 MHz, $CDCl_3$)

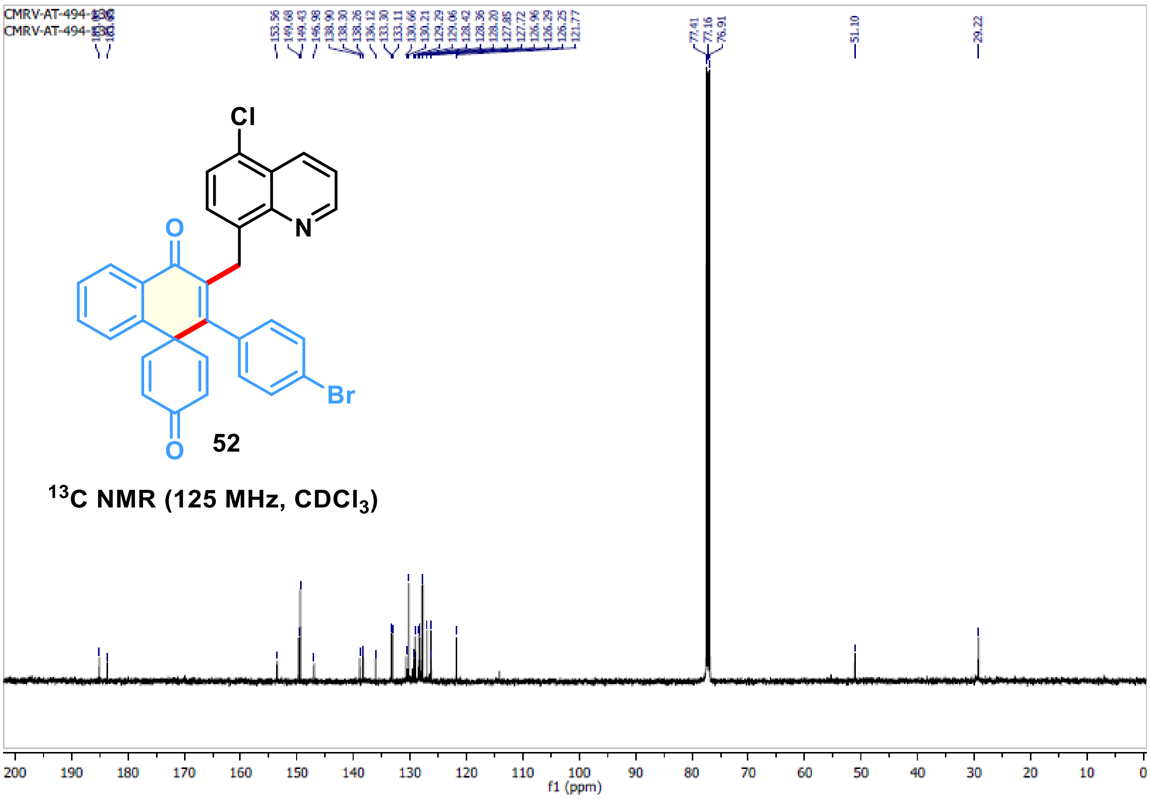
CMRV-AT-494-13C
153.56
149.68
149.43
146.98
138.90
138.30
138.26
136.12
133.30
133.11
130.66
130.21
129.29
129.06
128.42
128.36
128.20
127.85
127.72
126.96
126.29
126.25
121.77
77.41
77.16
76.91
51.10
29.22
Cl
N
O
Br
52
13C NMR (125 MHz, CDCl3)
f1 (ppm)

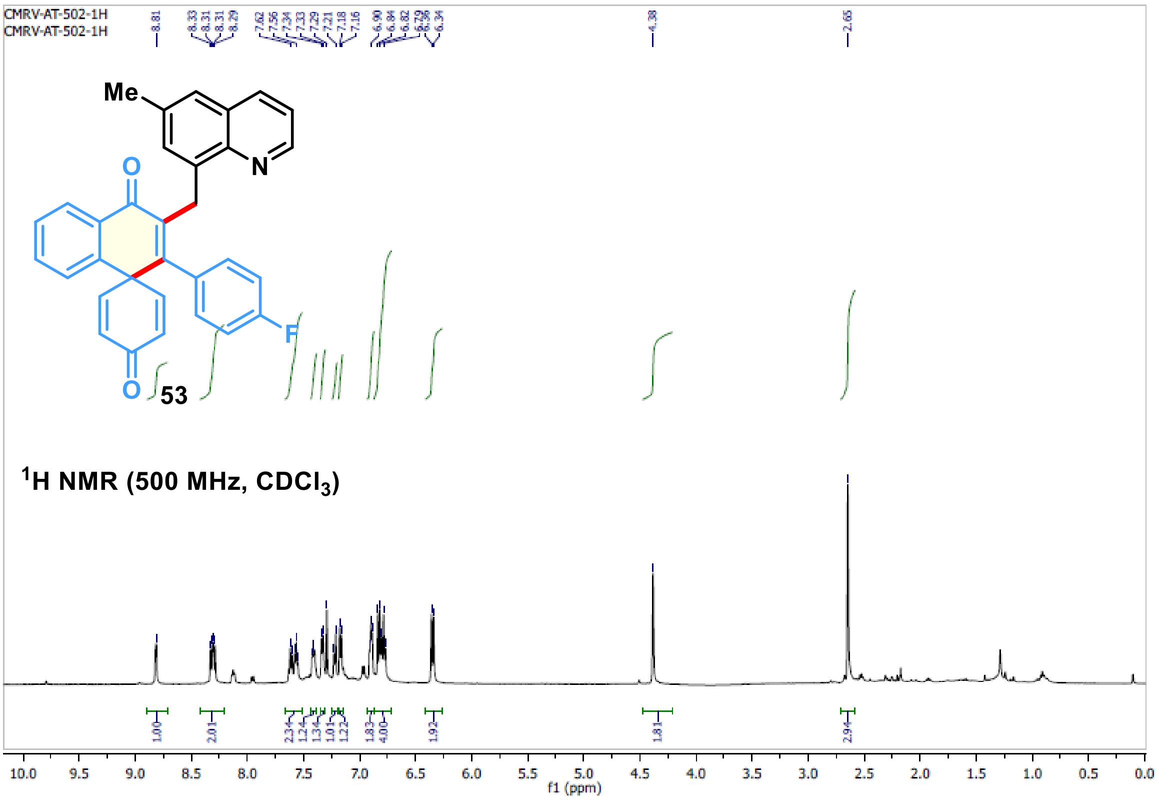
CMRV-AT-502-1H
8.81
8.33
8.31
8.31
8.29
7.62
7.56
7.34
7.33
7.29
7.21
7.18
7.16
6.90
6.84
6.82
6.79
6.36
6.34
4.38
2.65
Me
N
O
F
53
1H NMR (500 MHz, CDCl3)
1.00
2.01
2.34
1.24
1.34
1.01
1.22
1.83
4.00
1.92
1.81
2.94
f1 (ppm)

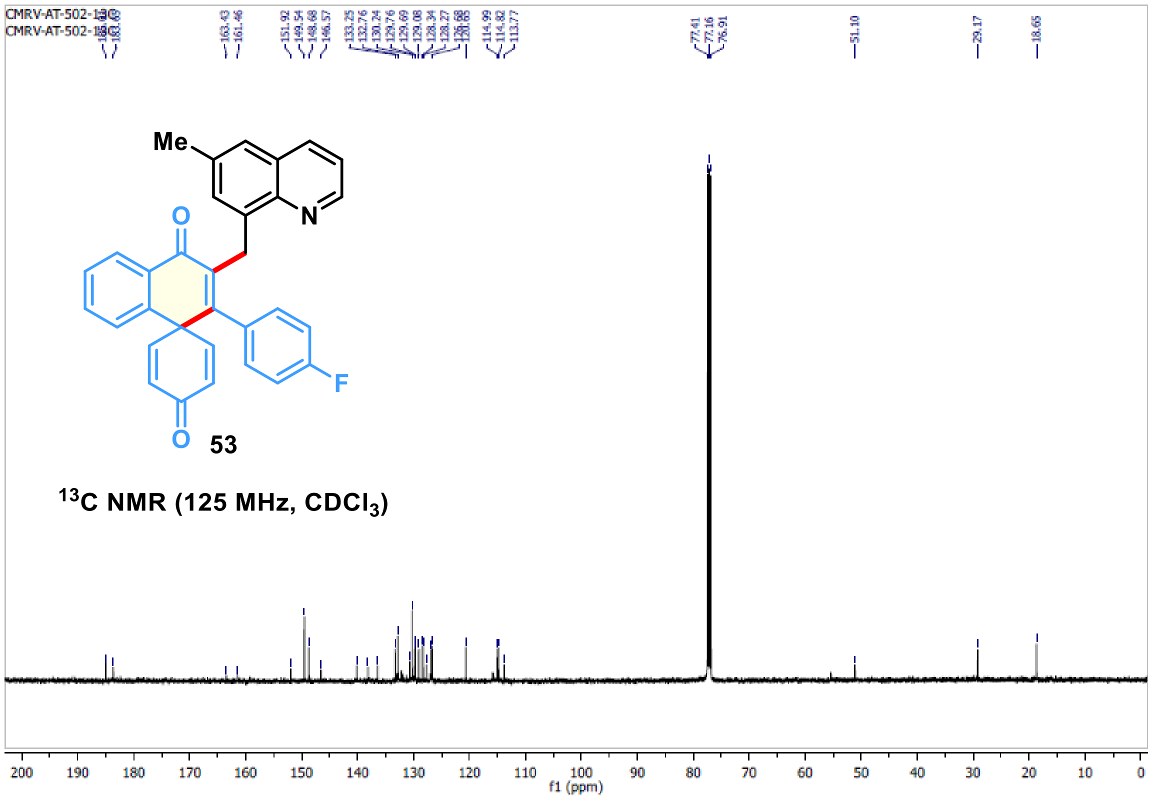

53
^{13}C NMR (125 MHz, CDCl$_3$)
163.43
161.46
151.92
149.54
148.68
146.57
133.25
132.76
130.24
129.76
129.69
129.08
128.34
128.27
114.99
114.82
113.77
77.41
77.16
76.91
51.10
29.17
18.65
f1 (ppm)

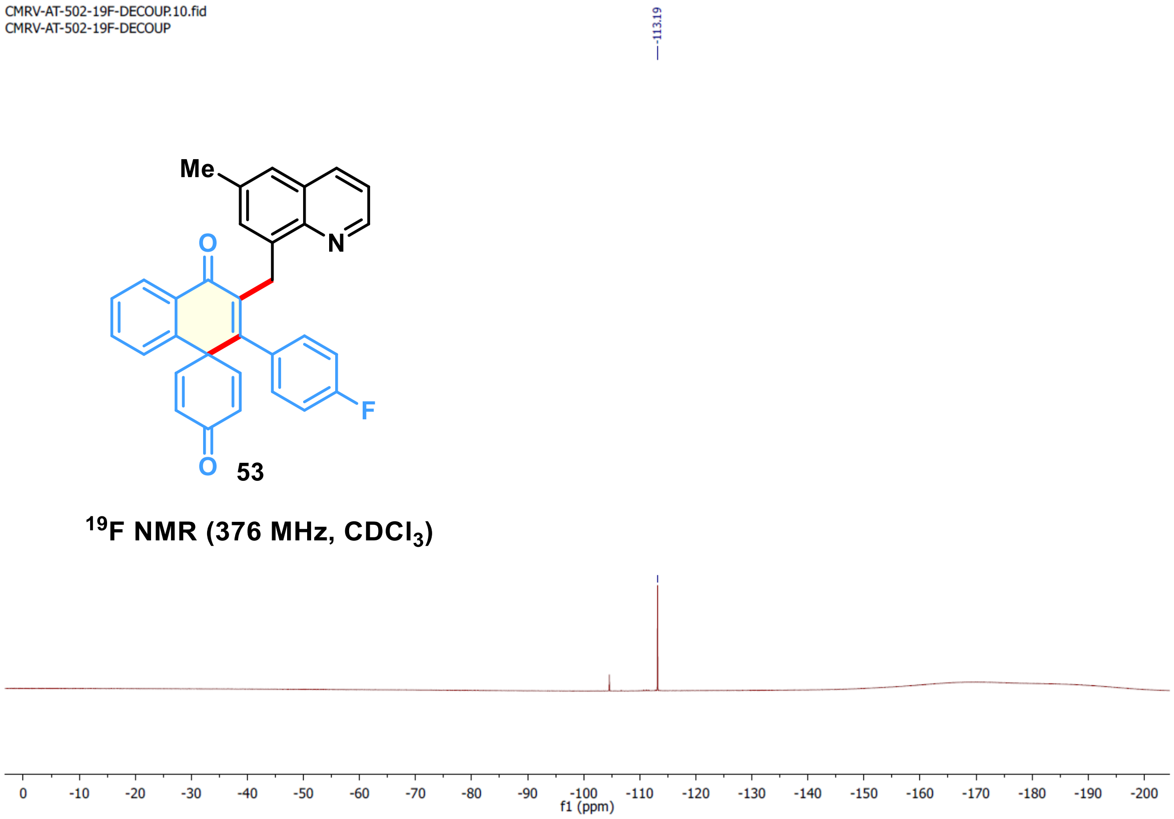

CMRV-AT-502-19F-DECOUP.10.fid
CMRV-AT-502-19F-DECOUP
-113.19
53
^{19}F NMR (376 MHz, CDCl$_3$)
f1 (ppm)

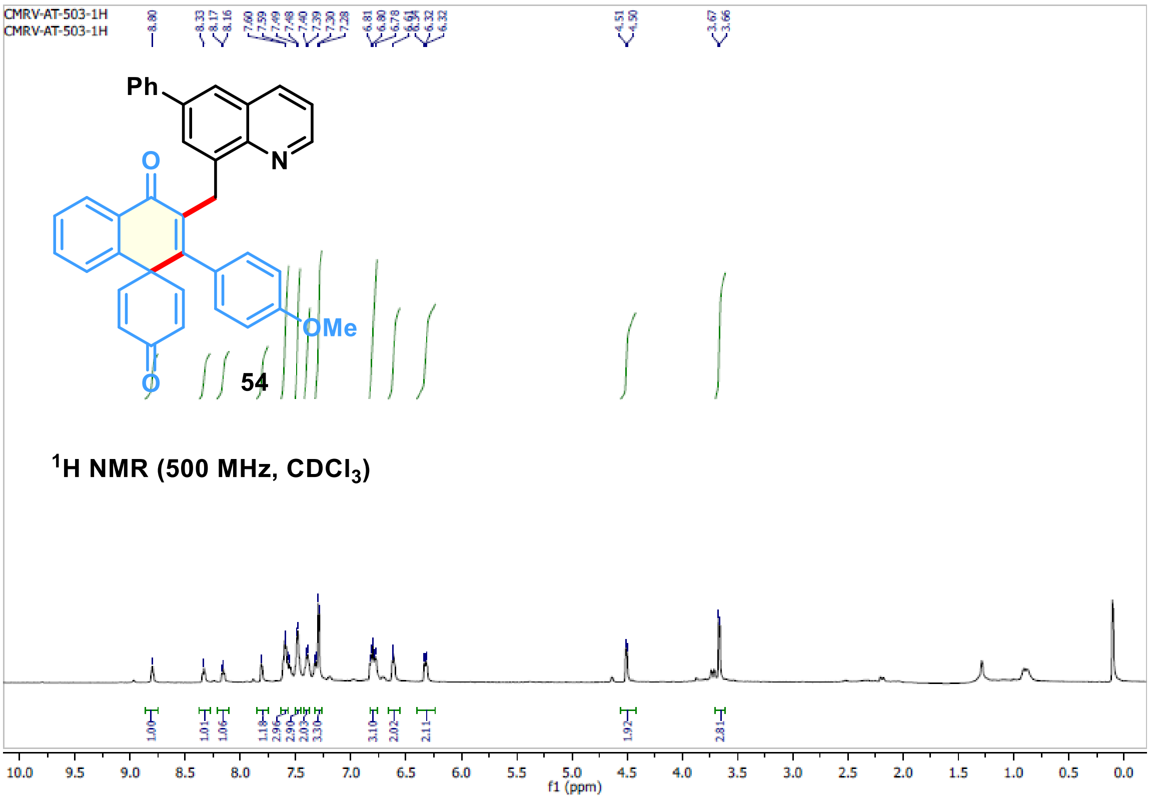

54

^{1}H NMR (500 MHz, $CDCl_3$)

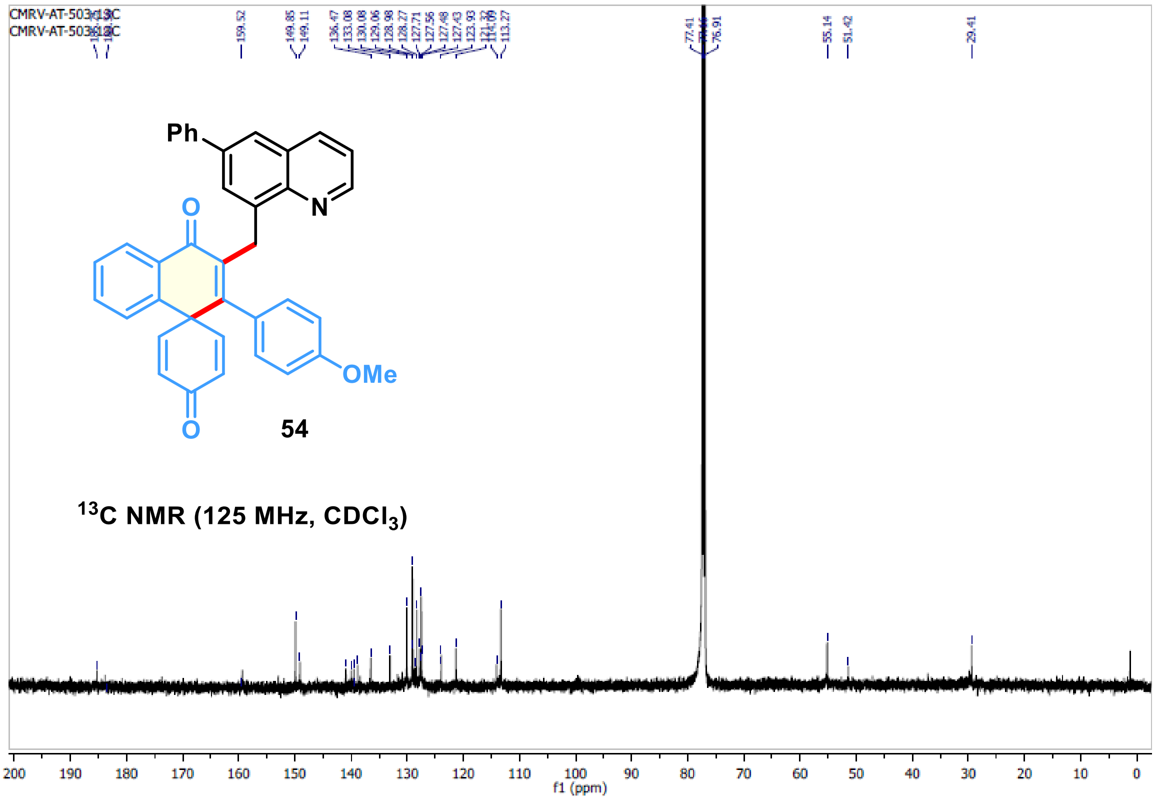

54

^{13}C NMR (125 MHz, $CDCl_3$)

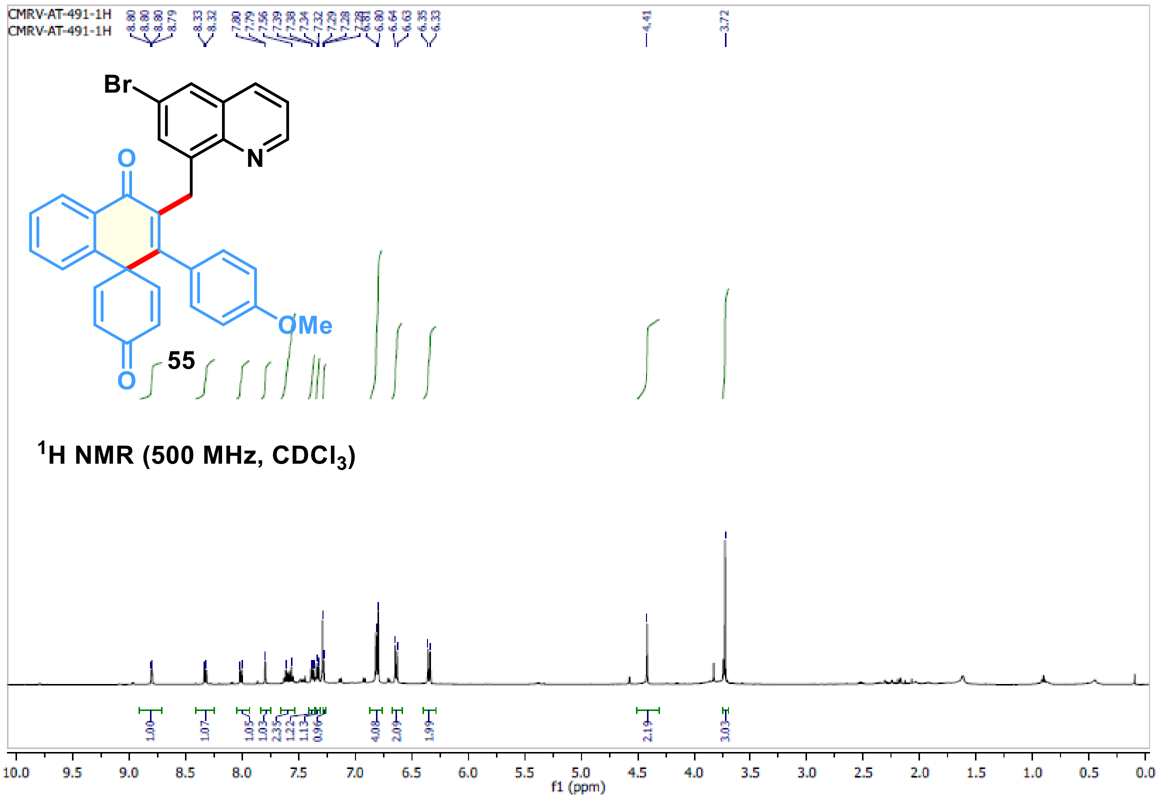
CMRV-AT-491-1H
CMRV-AT-491-1H
8.80 8.80 8.80 8.79 8.33 8.32 7.80 7.79 7.56 7.39 7.38 7.34 7.32 7.29 7.28 7.28 6.81 6.80 6.64 6.63 6.35 6.33
4.41
3.72
Br
N
O
O
OMe
55
1H NMR (500 MHz, CDCl3)
1.00 1.07 1.05 1.03 2.35 1.22 1.13 0.96 4.08 2.09 1.99 2.19 3.03
10.0 9.5 9.0 8.5 8.0 7.5 7.0 6.5 6.0 5.5 5.0 4.5 4.0 3.5 3.0 2.5 2.0 1.5 1.0 0.5 0.0
f1 (ppm)

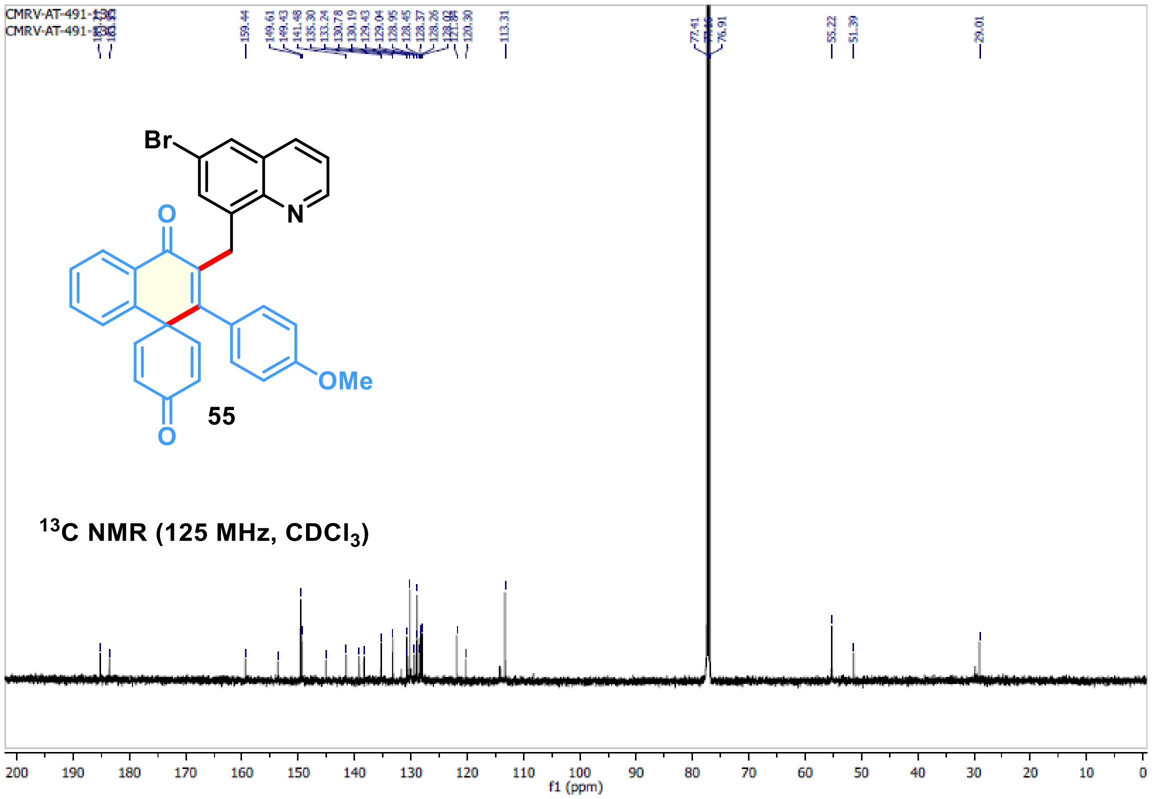
CMRV-AT-491-13C
CMRV-AT-491-13C
159.44
149.61 149.43 141.48 135.30 133.24 130.78 130.19 129.43 129.04 128.95 128.45 128.37 128.26 120.30
113.31
77.41 76.91
55.22 51.39
29.01
Br
N
O
O
OMe
55
13C NMR (125 MHz, CDCl3)
200 190 180 170 160 150 140 130 120 110 100 90 80 70 60 50 40 30 20 10 0
f1 (ppm)

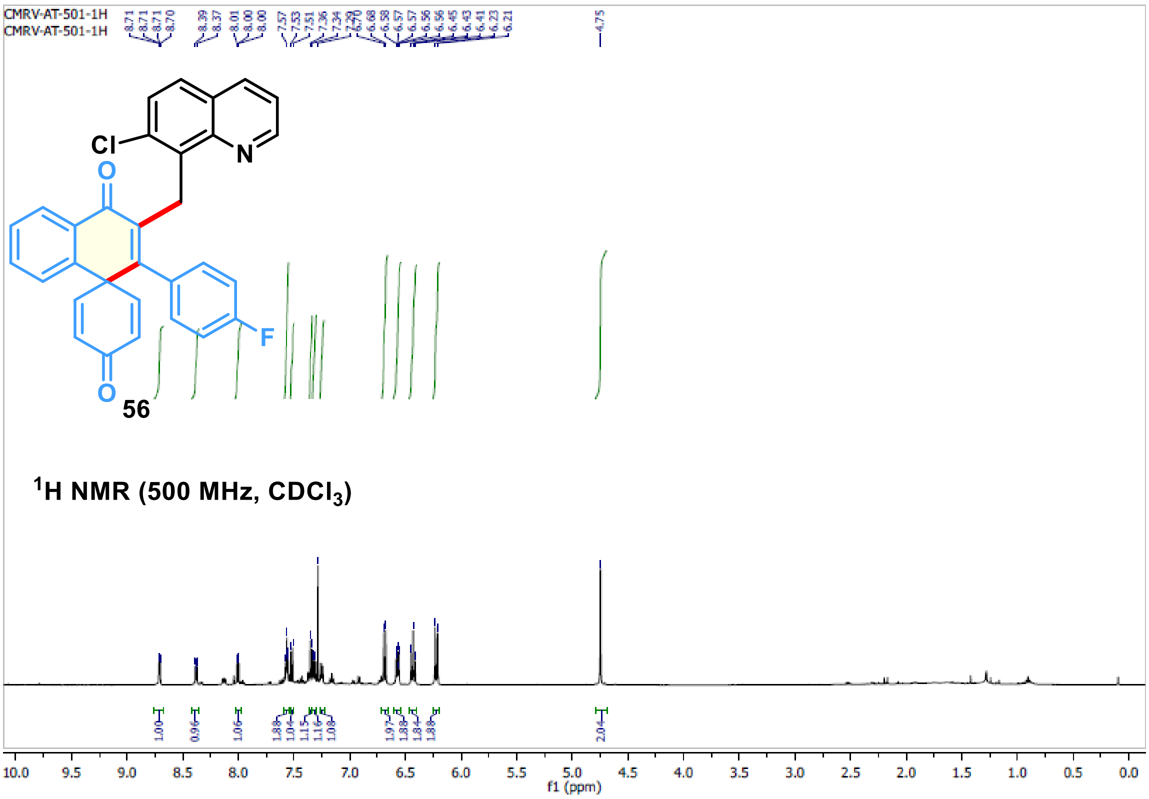
CMRV-AT-501-1H
Cl
N
O
F
56
^{1}H NMR (500 MHz, CDCl$_3$)
f1 (ppm)

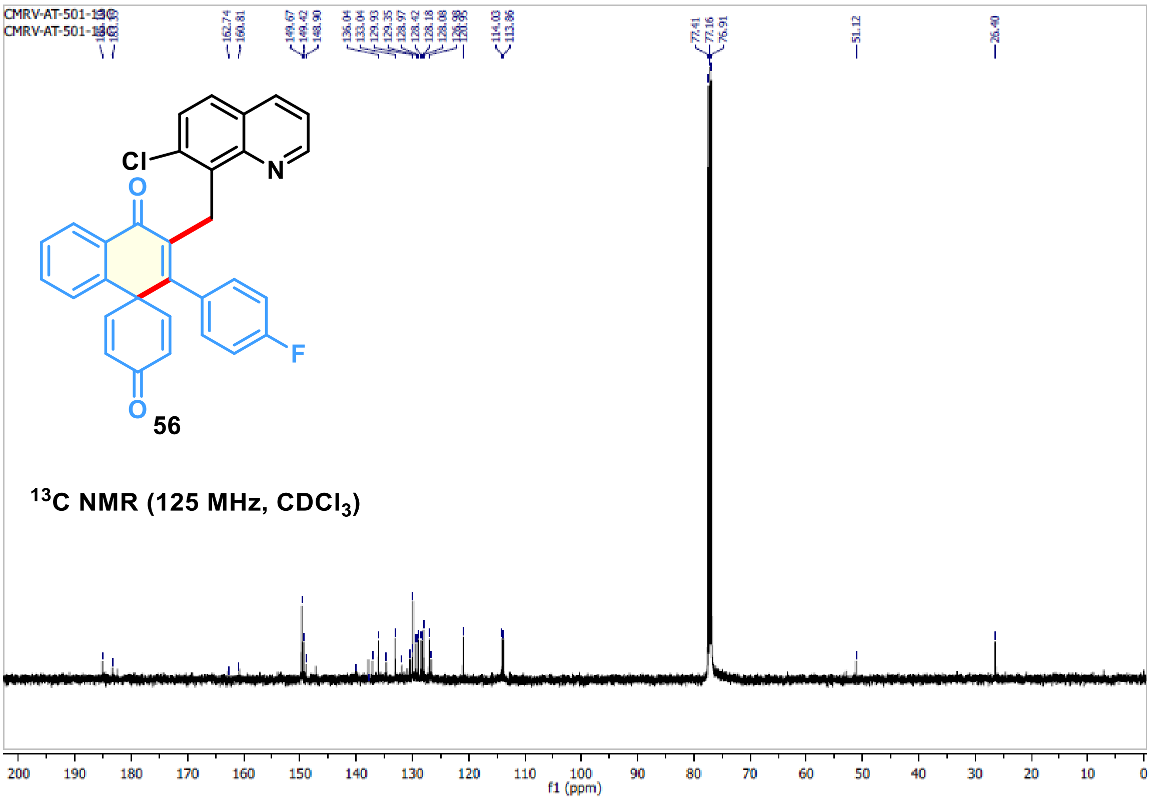
Cl
N
O
F
56
^{13}C NMR (125 MHz, CDCl$_3$)
f1 (ppm)

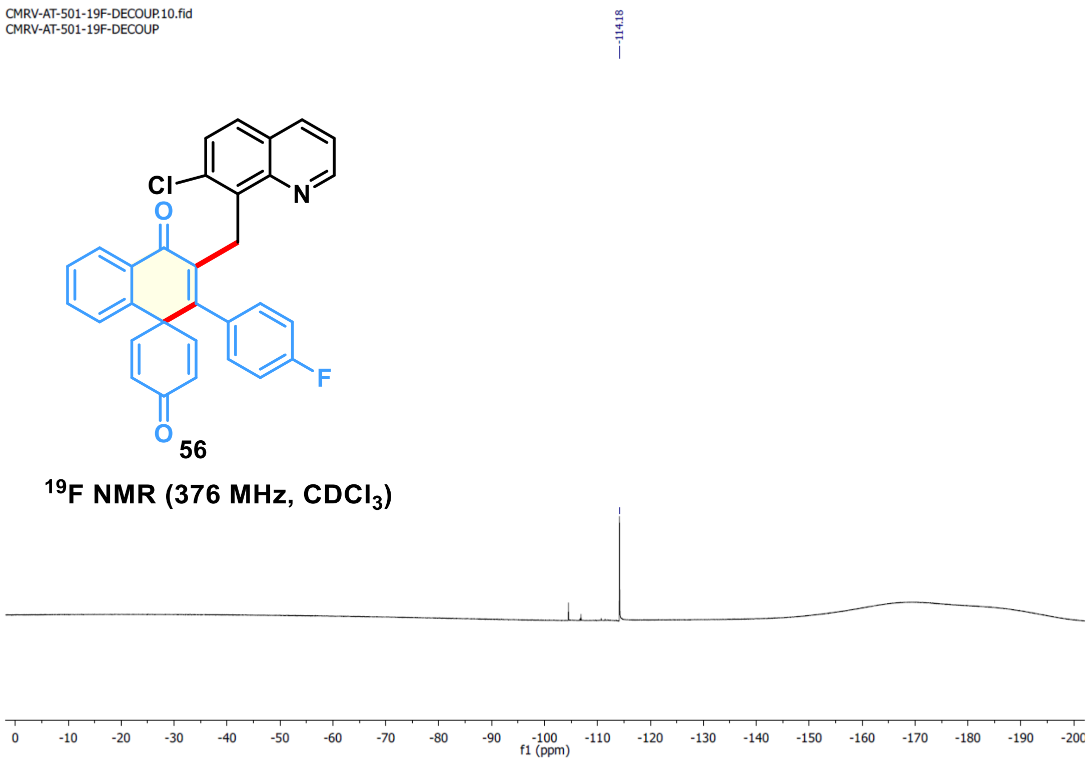
CMRV-AT-501-19F-DECOUP.10.fid
CMRV-AT-501-19F-DECOUP
-114.18
Cl
O
N
F
O
56
^{19}F NMR (376 MHz, CDCl$_3$)
f1 (ppm)

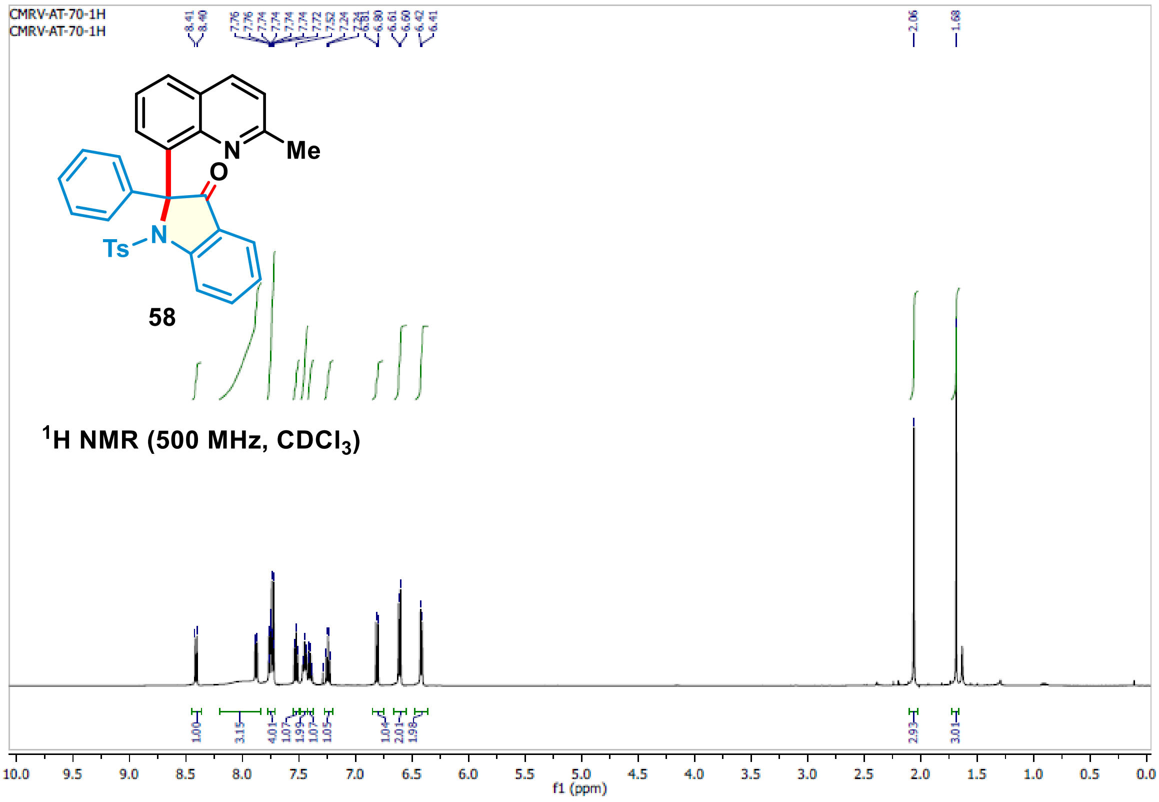
CMRV-AT-70-1H
CMRV-AT-70-1H
8.41
8.40
7.76
7.76
7.74
7.74
7.74
7.74
7.72
7.52
7.24
7.24
6.81
6.80
6.61
6.60
6.42
6.41
2.06
1.68
N
Me
O
Ts
N
58
^{1}H NMR (500 MHz, CDCl$_3$)
1.00
3.15
4.01
1.07
1.95
1.07
1.05
1.04
2.01
1.98
2.93
3.01
f1 (ppm)

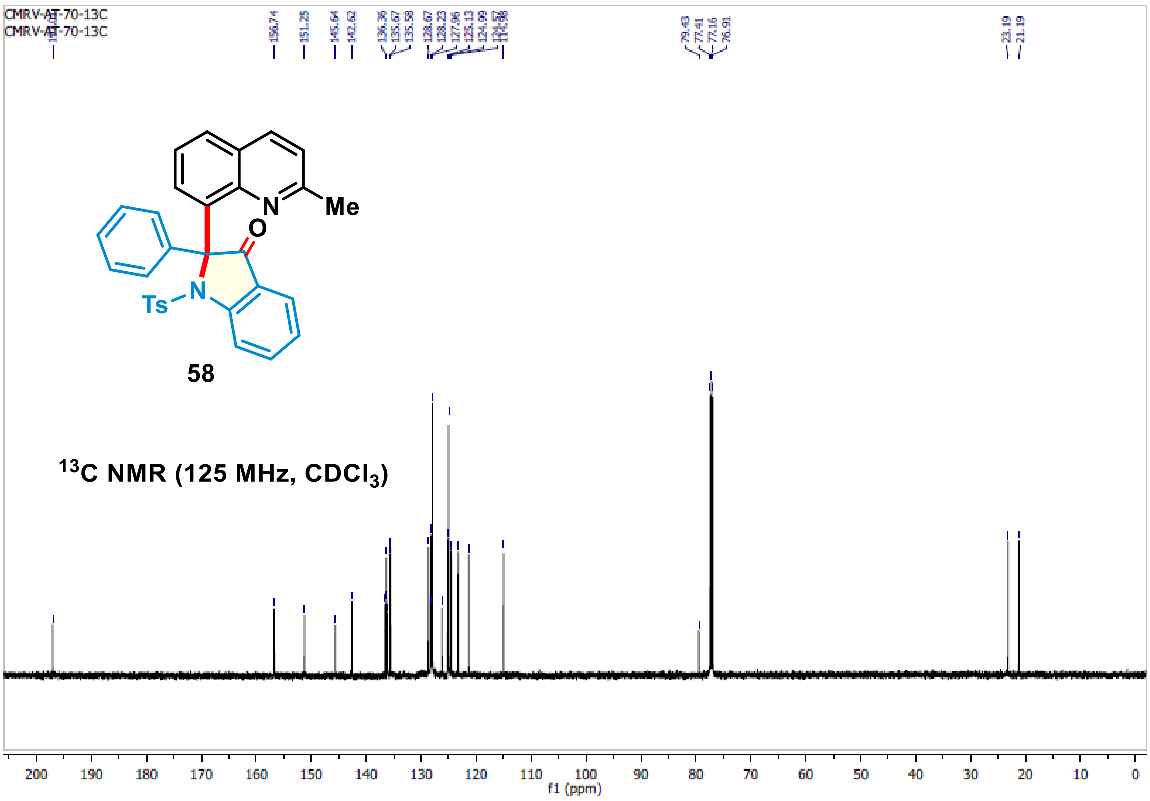
CMRV-AT-70-13C
CMRV-AT-70-13C
156.74
151.25
145.64
142.62
136.36
135.67
135.58
128.67
128.23
127.96
125.13
124.99
79.43
77.41
77.16
76.91
23.19
21.19
N
Me
O
Ts
N
58
^{13}C NMR (125 MHz, CDCl$_3$)
f1 (ppm)

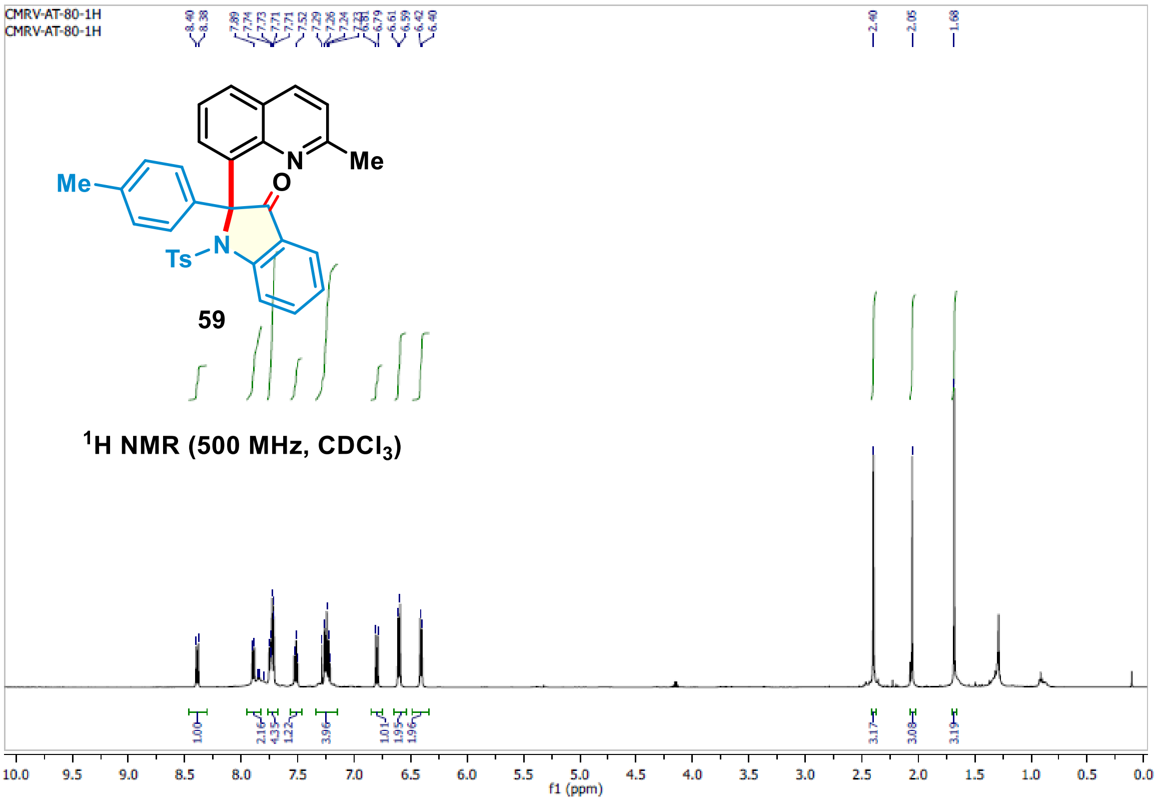
CMRV-AT-80-1H
CMRV-AT-80-1H
8.40
8.38
7.89
7.74
7.73
7.71
7.71
7.52
7.29
7.26
7.24
7.23
6.81
6.79
6.61
6.59
6.42
6.40
2.40
2.05
1.68
N
Me
Me
O
Ts
N
59
^{1}H NMR (500 MHz, CDCl$_3$)
1.00
2.16
4.35
1.22
3.96
1.01
1.95
1.96
3.17
3.08
3.19
f1 (ppm)

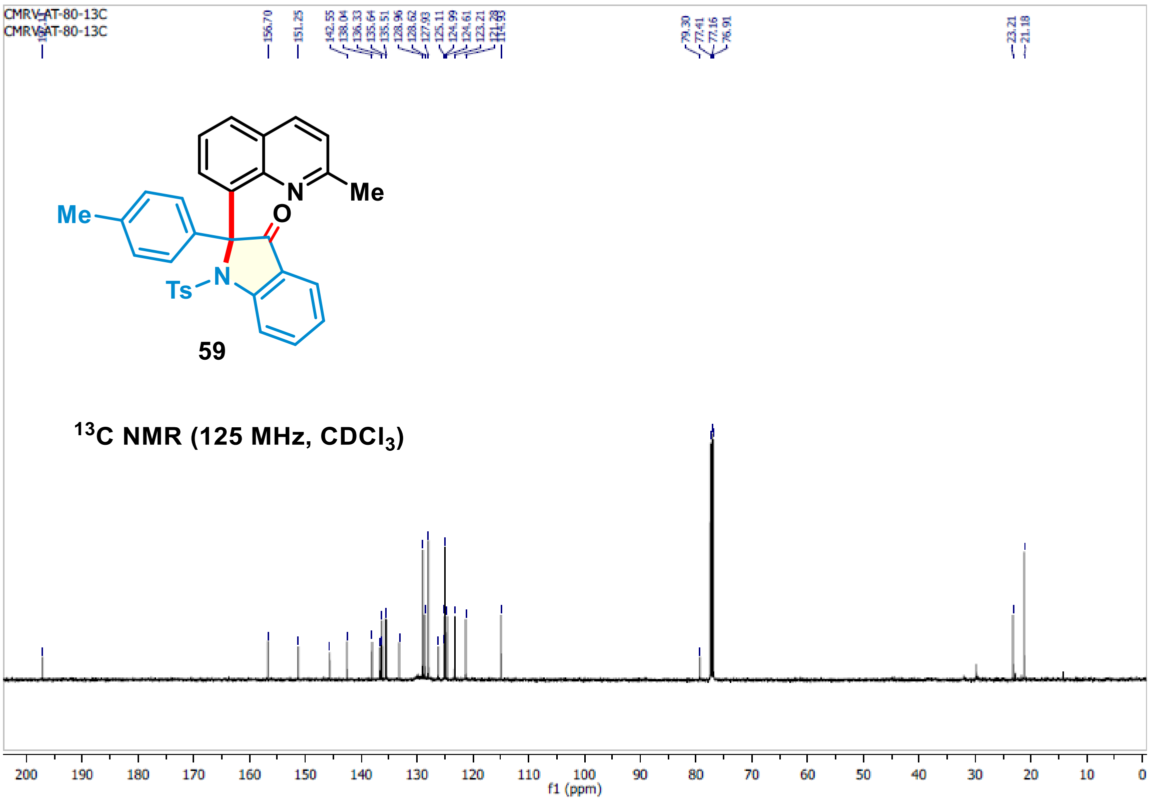
CMRV-AT-80-13C
156.70
151.25
142.55
138.04
136.33
135.64
135.51
128.96
128.62
127.93
125.11
124.99
124.61
123.21
79.30
77.41
77.16
76.91
23.21
21.18
Me
N
Me
O
Ts
N
59
13C NMR (125 MHz, CDCl3)
f1 (ppm)

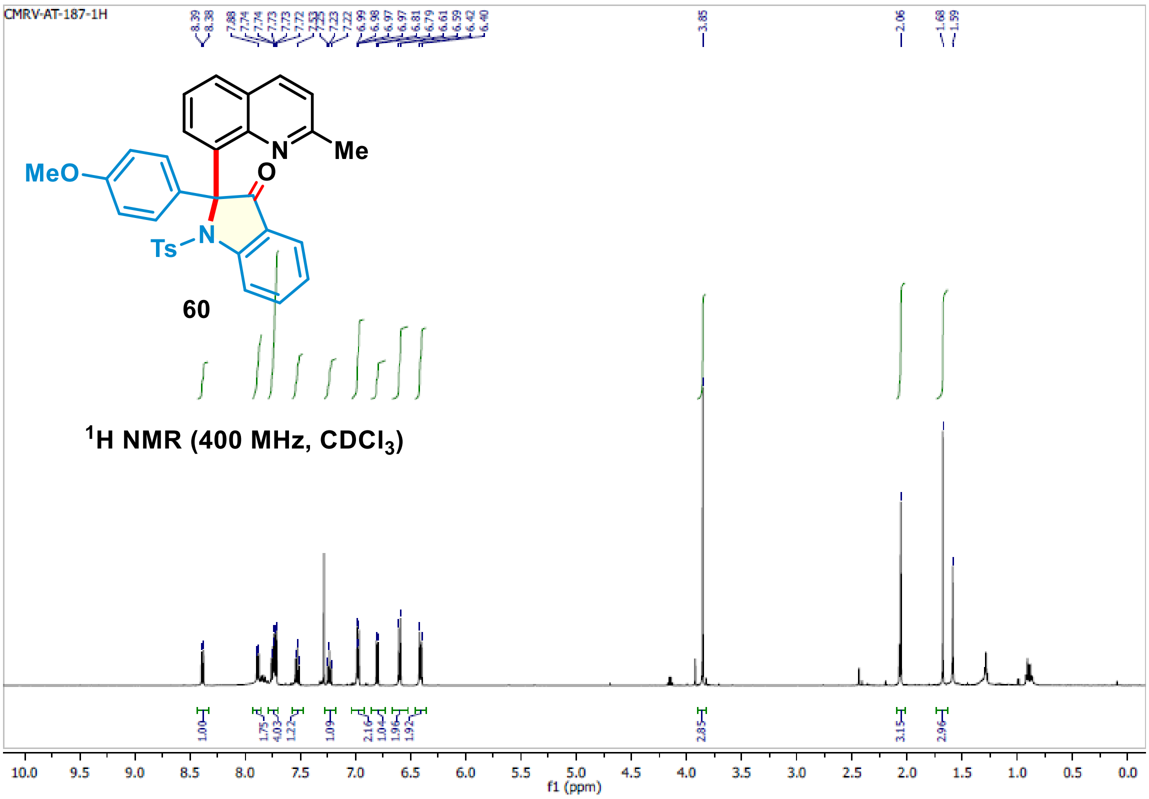
CMRV-AT-187-1H
8.39
8.38
7.88
7.74
7.74
7.73
7.73
7.72
7.53
7.23
7.22
6.99
6.98
6.97
6.97
6.81
6.79
6.61
6.59
6.42
6.40
3.85
2.06
1.68
1.59
N
Me
MeO
O
Ts
N
60
1H NMR (400 MHz, CDCl3)
1.00
1.75
4.03
1.22
1.09
2.16
1.04
1.96
1.92
2.85
3.15
2.96
f1 (ppm)

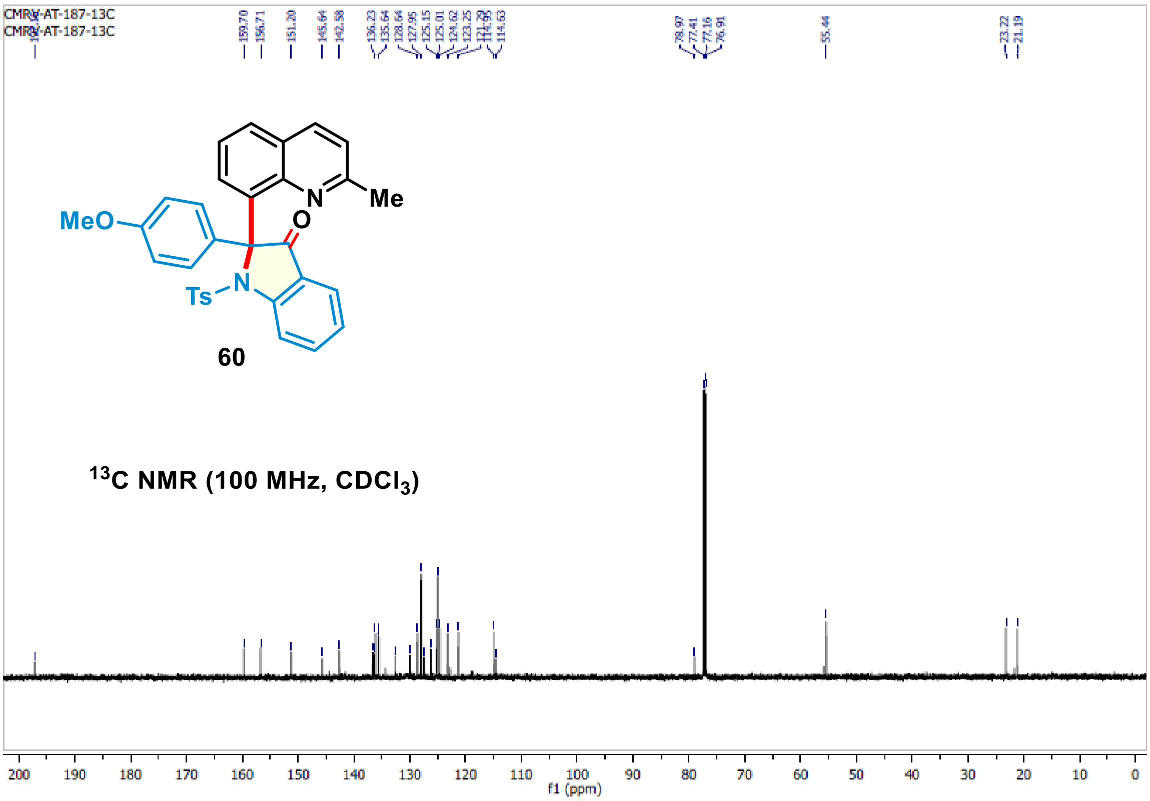
CMRV-AT-187-13C
MeO
N
Me
O
Ts
N
60
13C NMR (100 MHz, CDCl3)
159.70
156.71
151.20
145.64
142.58
136.23
135.64
128.64
127.95
125.15
125.01
124.62
123.25
114.63
78.97
77.41
77.16
76.91
55.44
23.22
21.19
f1 (ppm)

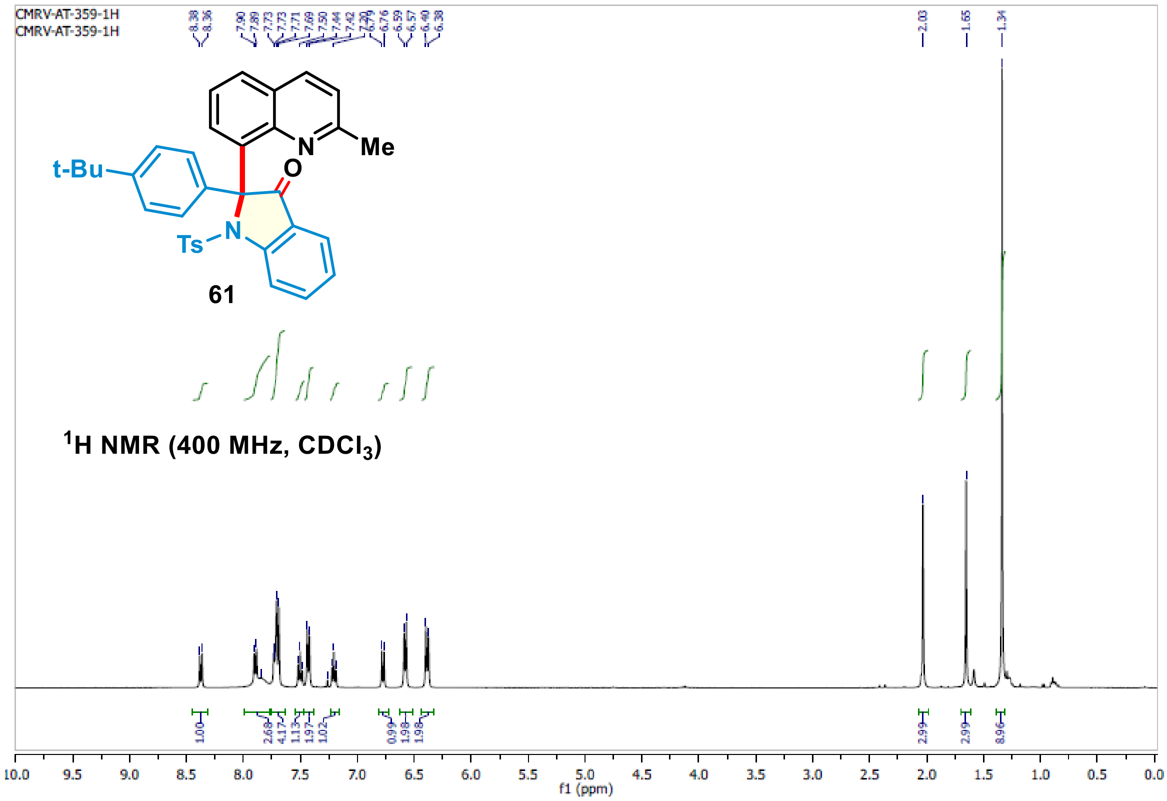
CMRV-AT-359-1H
t-Bu
N
Me
O
Ts
N
61
1H NMR (400 MHz, CDCl3)
8.38
8.36
7.90
7.89
7.73
7.71
7.69
7.50
7.44
7.42
6.79
6.76
6.59
6.57
6.40
6.38
2.03
1.65
1.34
1.00
2.68
4.17
1.13
1.97
1.02
0.99
1.98
1.98
2.99
2.99
8.96
f1 (ppm)

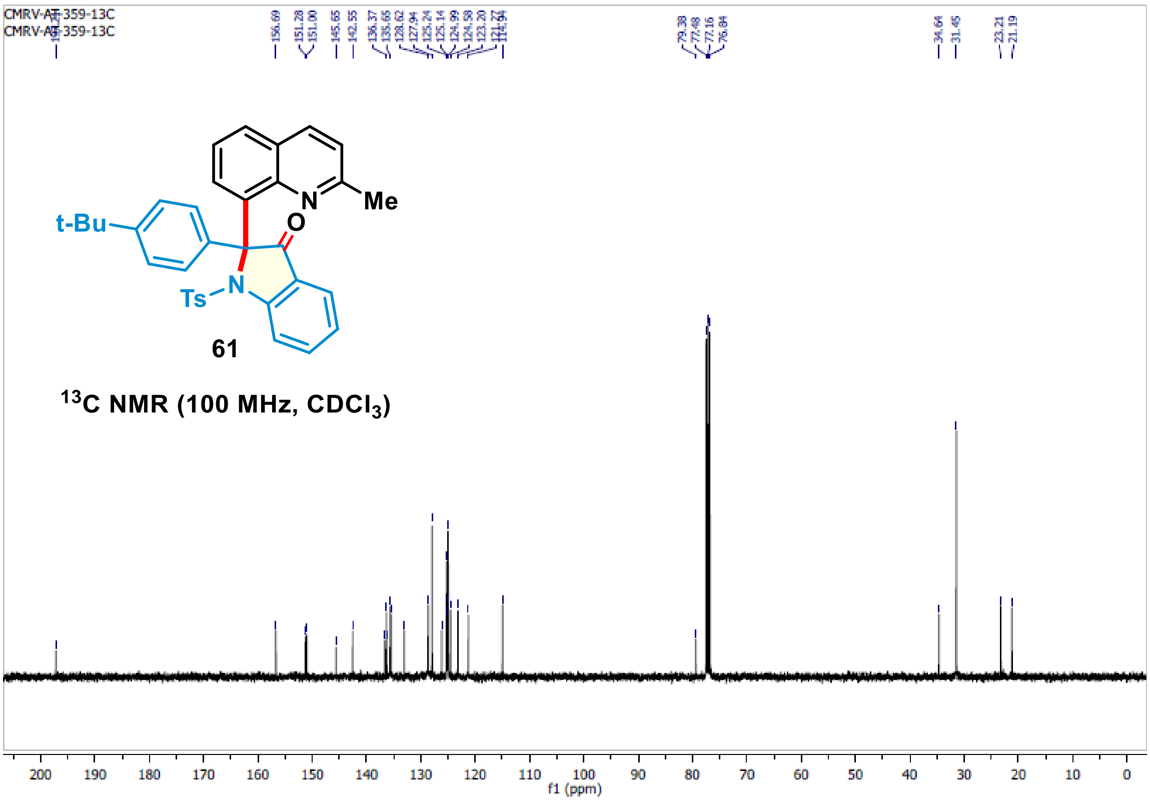
CMRV-AT-359-13C
t-Bu
Ts
N
O
N
Me
61
13C NMR (100 MHz, CDCl3)
f1 (ppm)

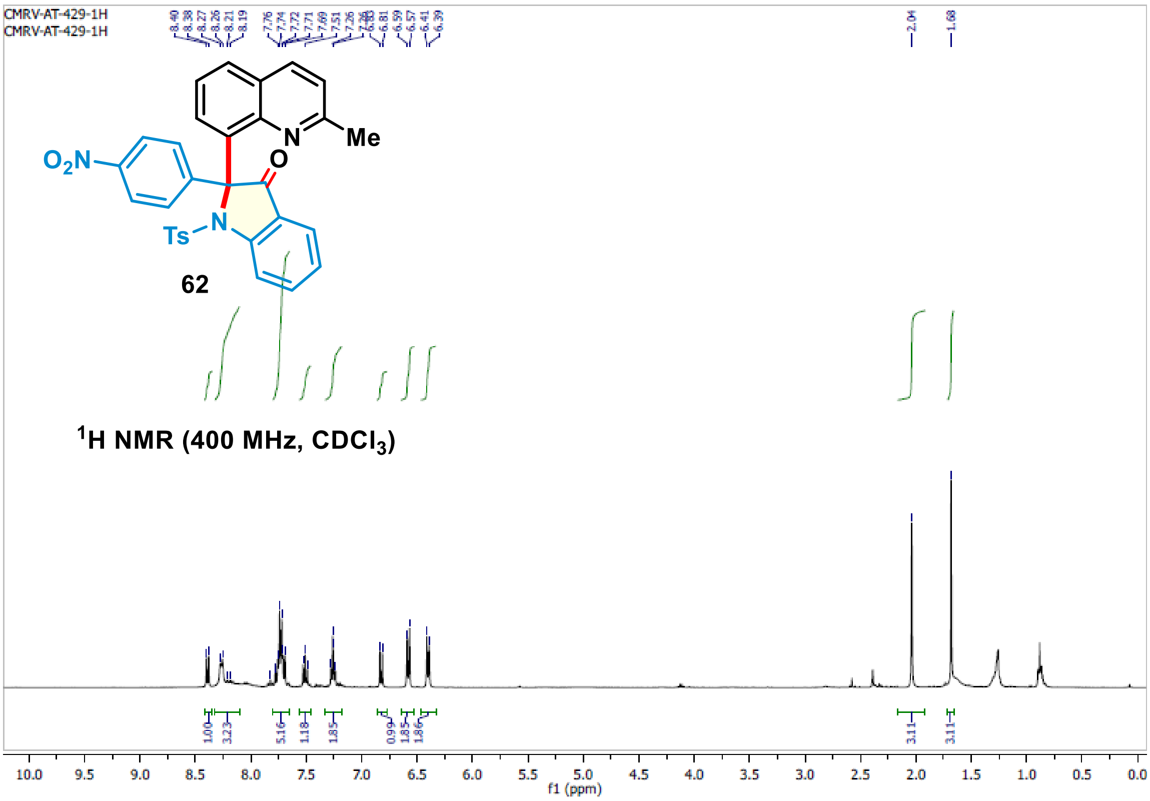
CMRV-AT-429-1H
O2N
Ts
N
O
N
Me
62
1H NMR (400 MHz, CDCl3)
f1 (ppm)

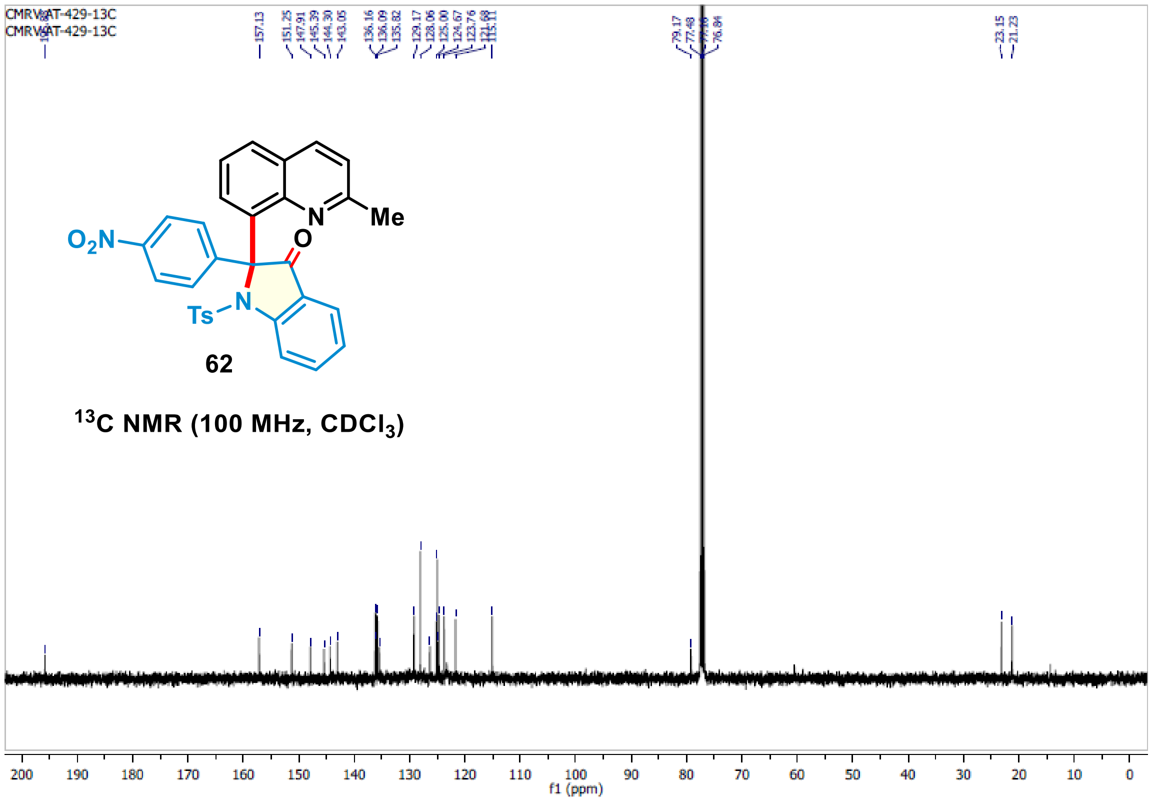
CMRV-AT-429-13C
CMRV-AT-429-13C
O2N
N
Me
O
Ts
N
62
13C NMR (100 MHz, CDCl3)
200 190 180 170 160 150 140 130 120 110 100 90 80 70 60 50 40 30 20 10 0
f1 (ppm)

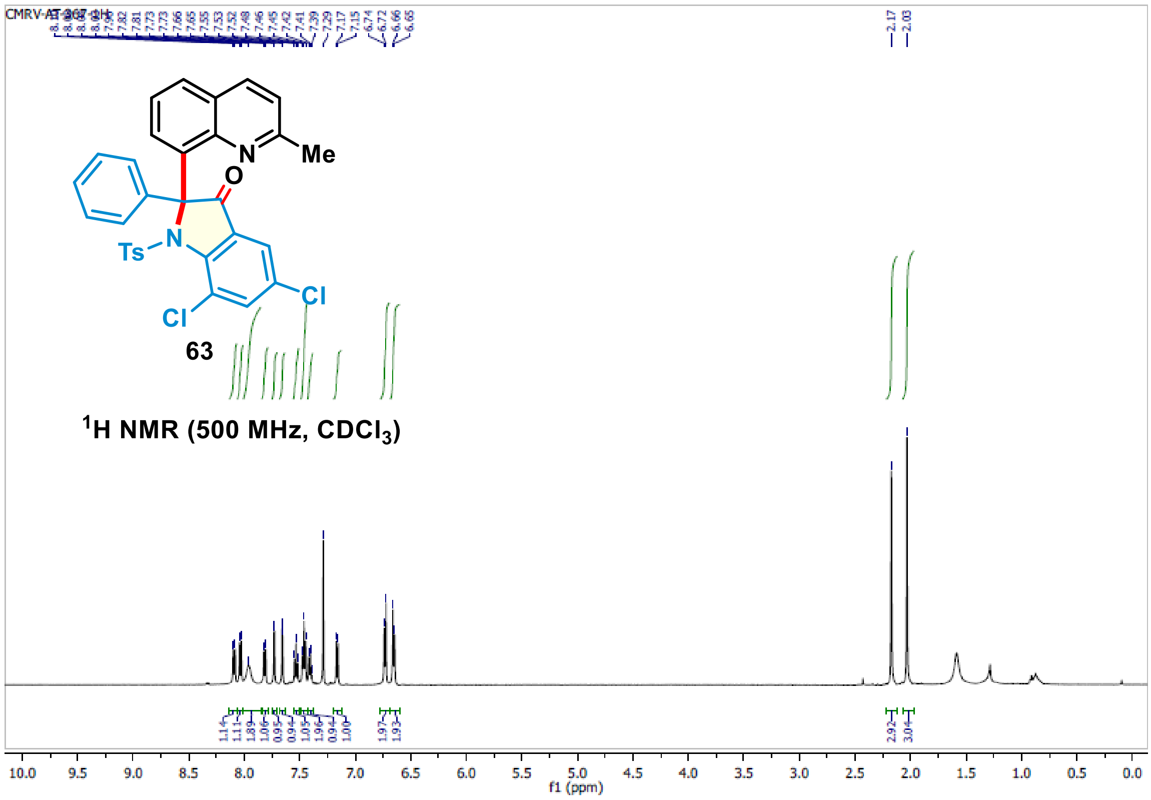
N
Me
O
Ts
N
Cl
Cl
63
1H NMR (500 MHz, CDCl3)
10.0 9.5 9.0 8.5 8.0 7.5 7.0 6.5 6.0 5.5 5.0 4.5 4.0 3.5 3.0 2.5 2.0 1.5 1.0 0.5 0.0
f1 (ppm)

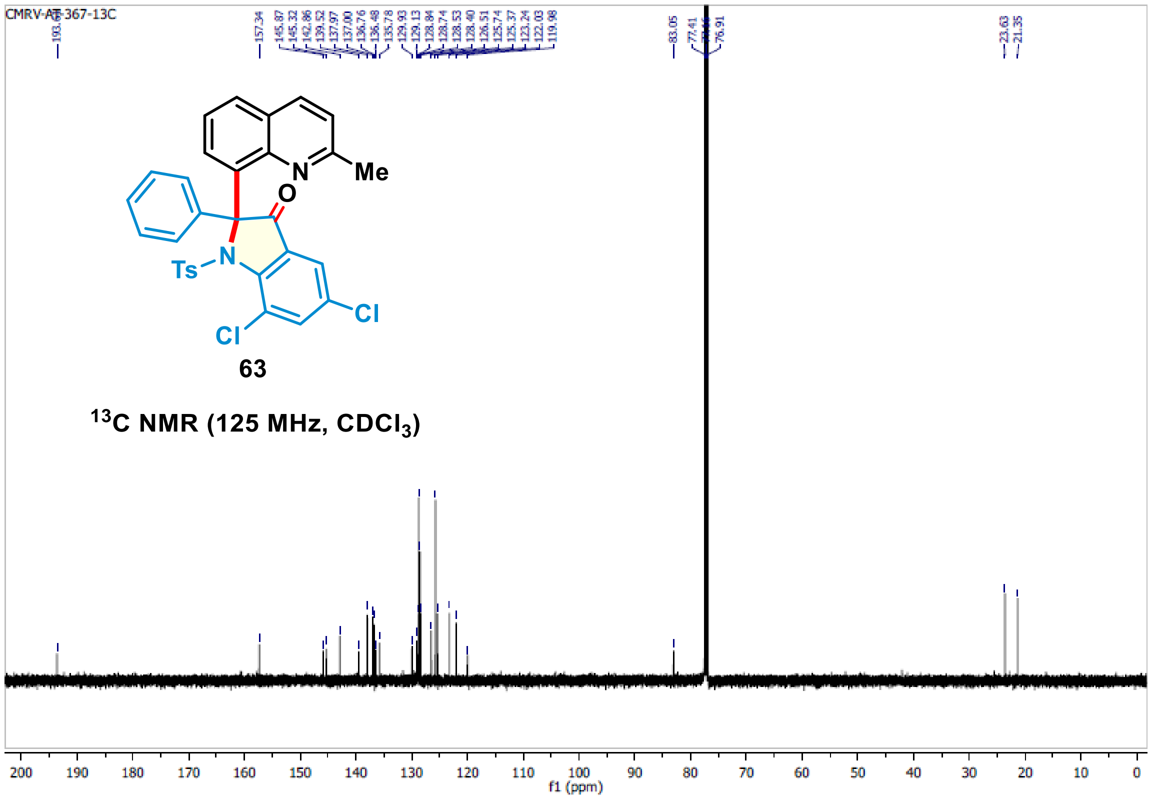
CMRV-AT-367-13C
157.34
145.87
145.32
142.86
139.52
137.97
137.00
136.76
136.48
135.78
129.93
129.13
128.84
128.74
128.53
128.40
126.51
125.74
125.37
123.24
122.03
119.98
83.05
77.41
76.91
23.63
21.35
N
Me
O
Ts
Cl
Cl
63
13C NMR (125 MHz, CDCl3)
f1 (ppm)

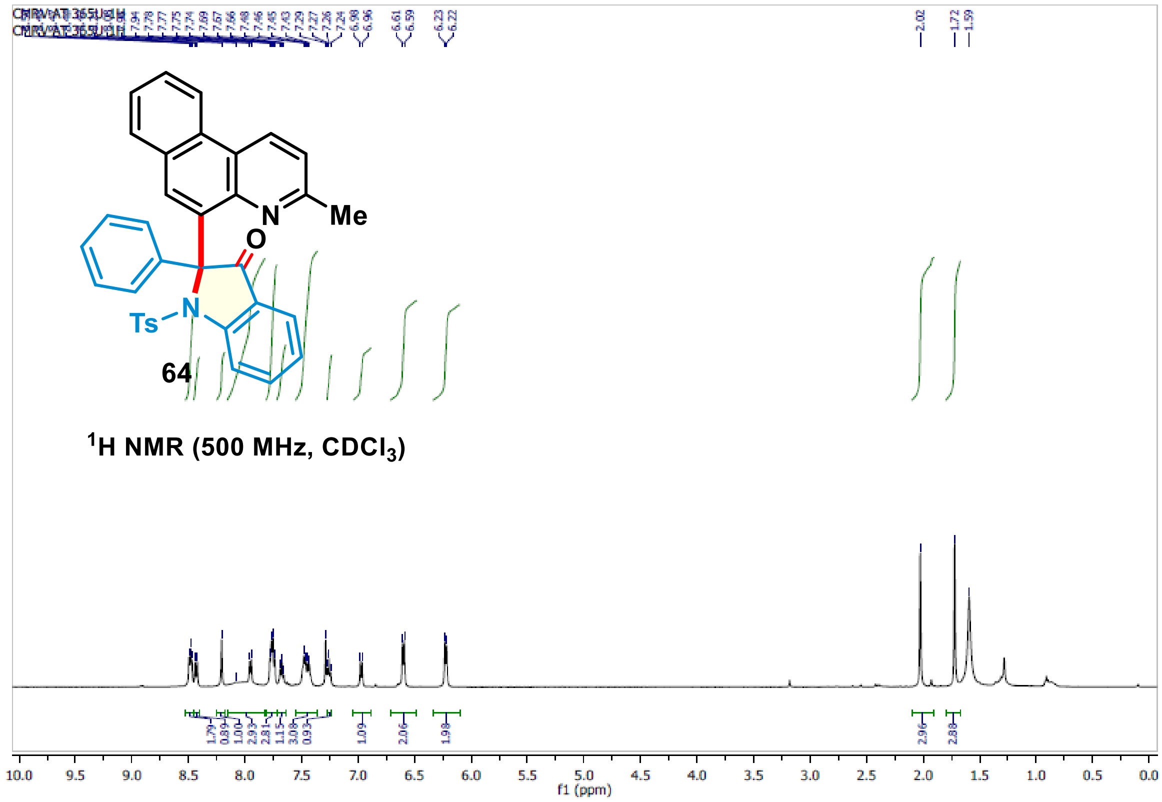
2.02
1.72
1.59
N
Me
O
Ts
N
64
1H NMR (500 MHz, CDCl3)
f1 (ppm)

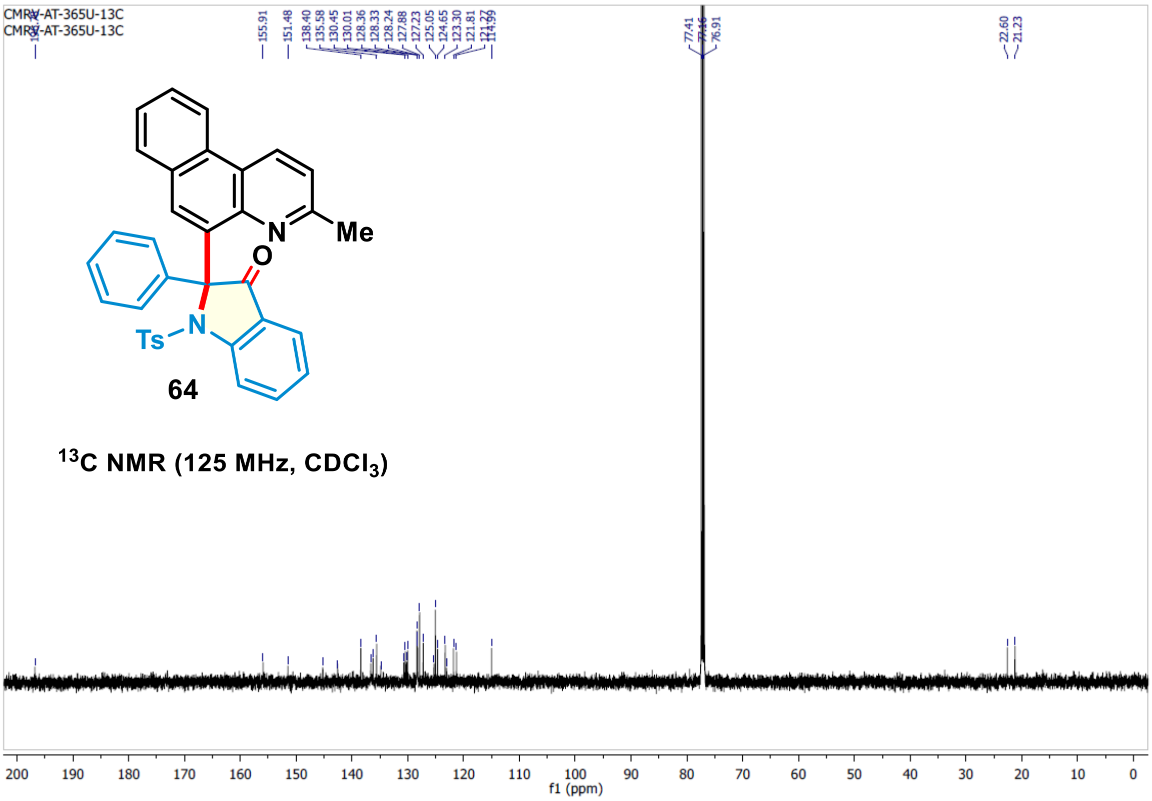

CMRV-AT-365U-13C
Me
N
O
Ts
N
64
^{13}C NMR (125 MHz, CDCl$_3$)
f1 (ppm)

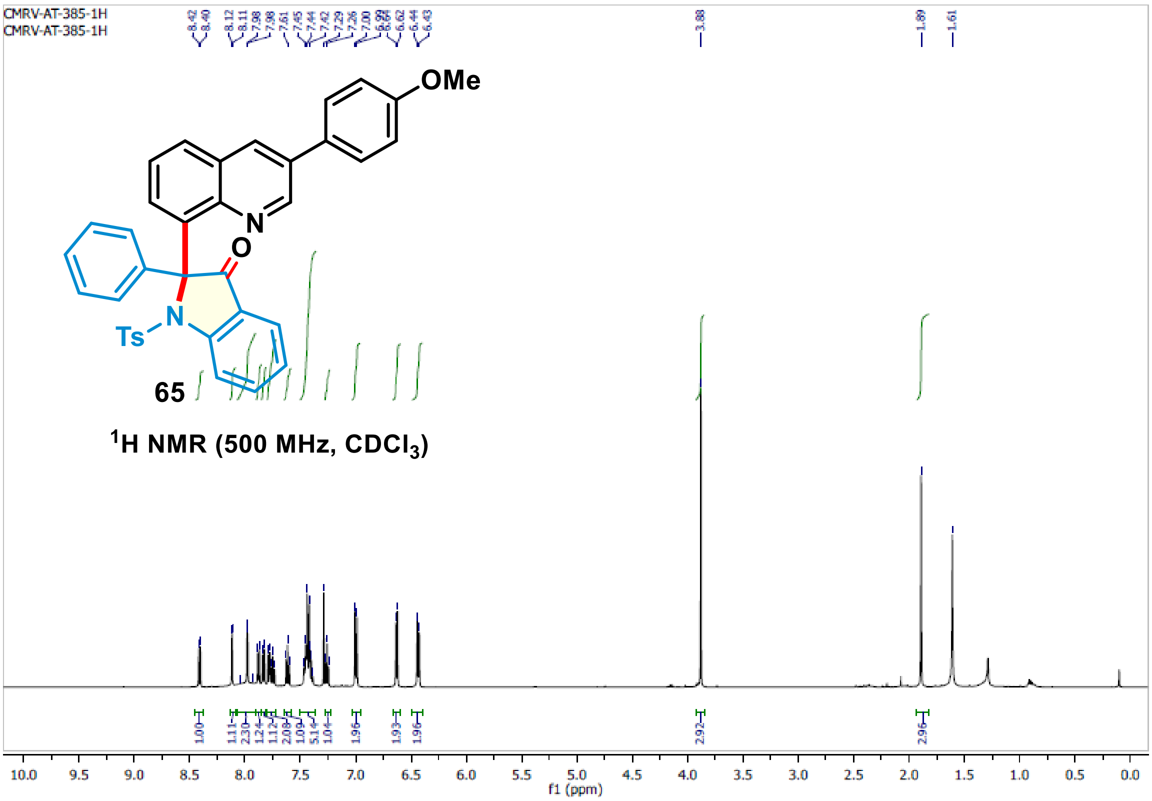

CMRV-AT-385-1H
OMe
N
O
Ts
N
65
^{1}H NMR (500 MHz, CDCl$_3$)
f1 (ppm)

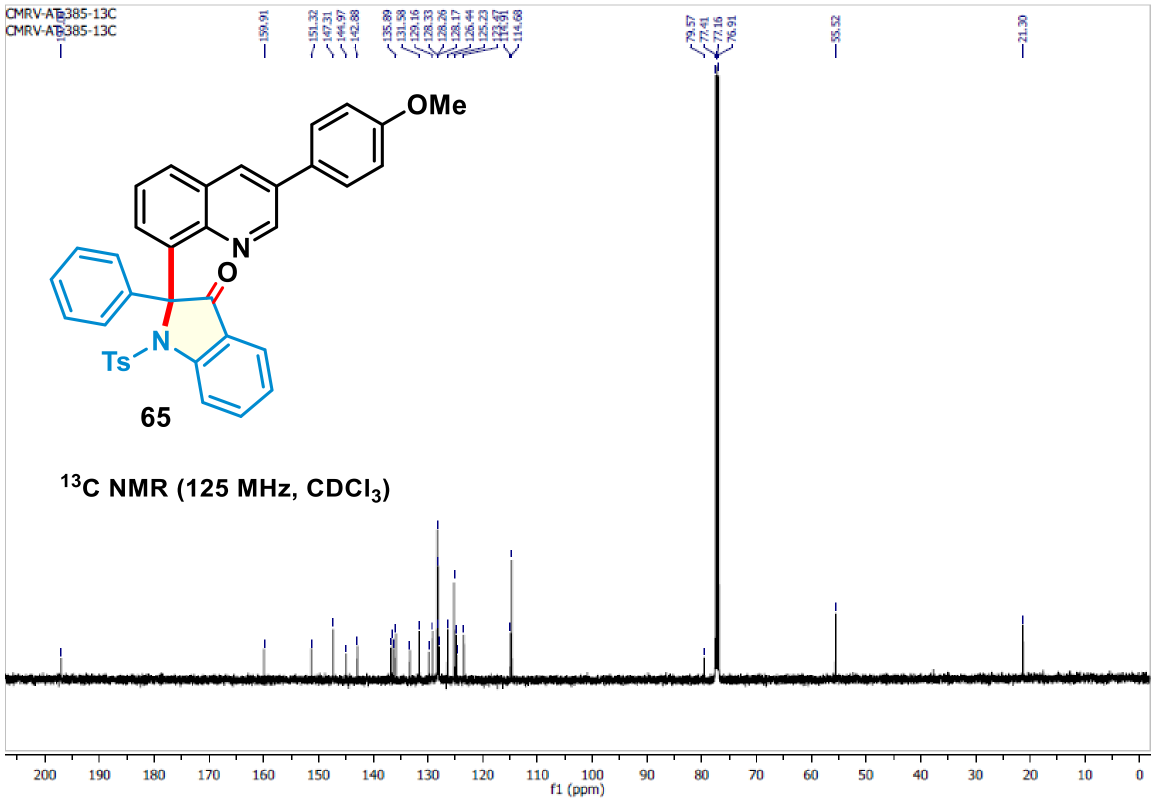

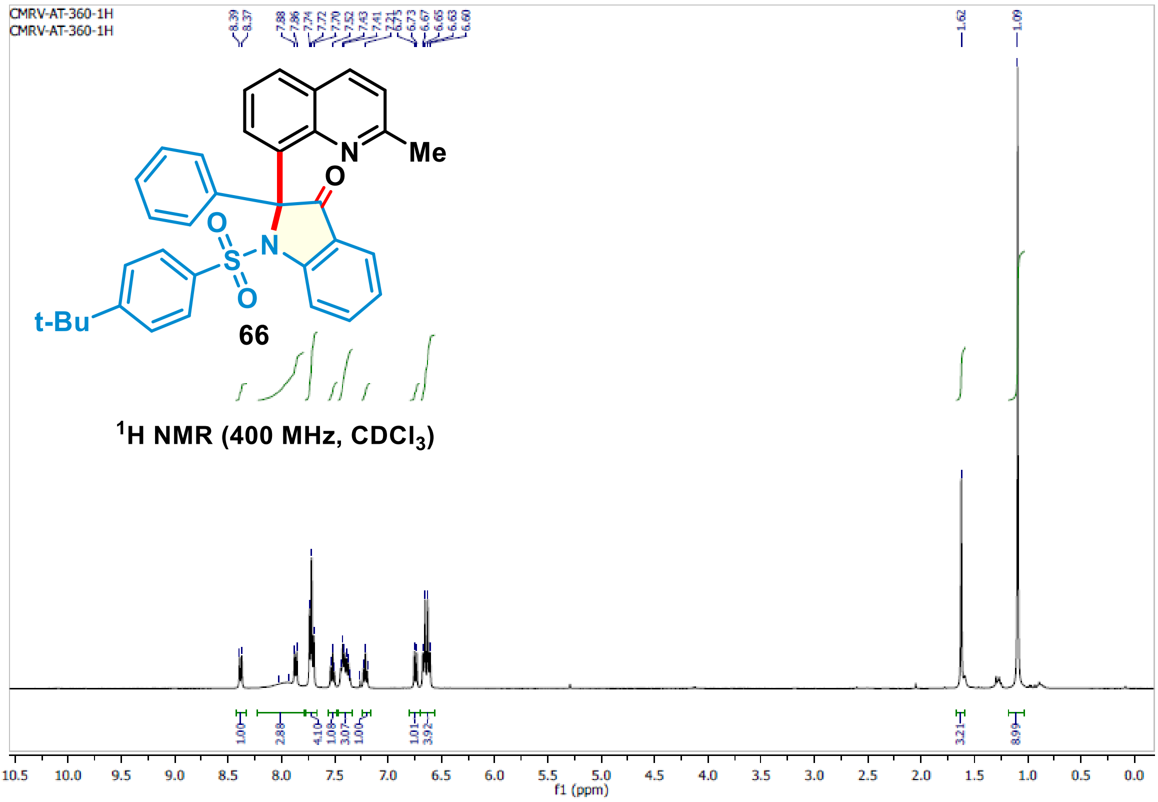

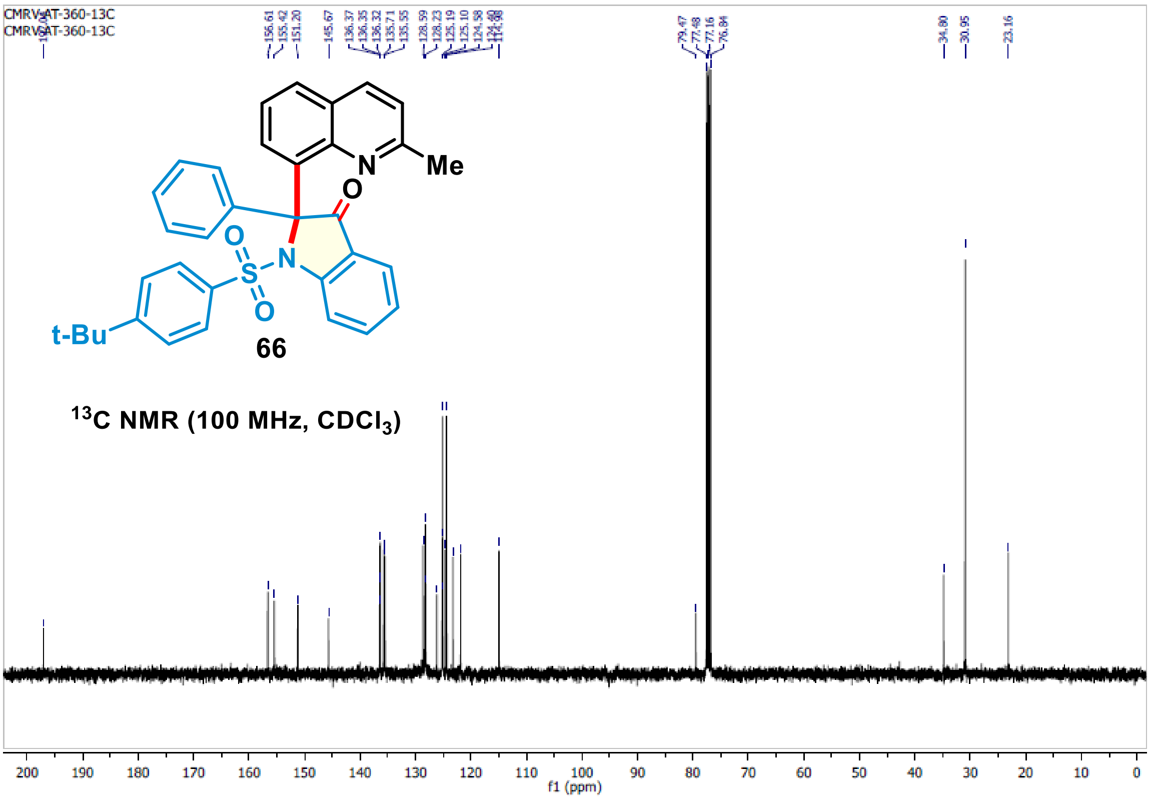

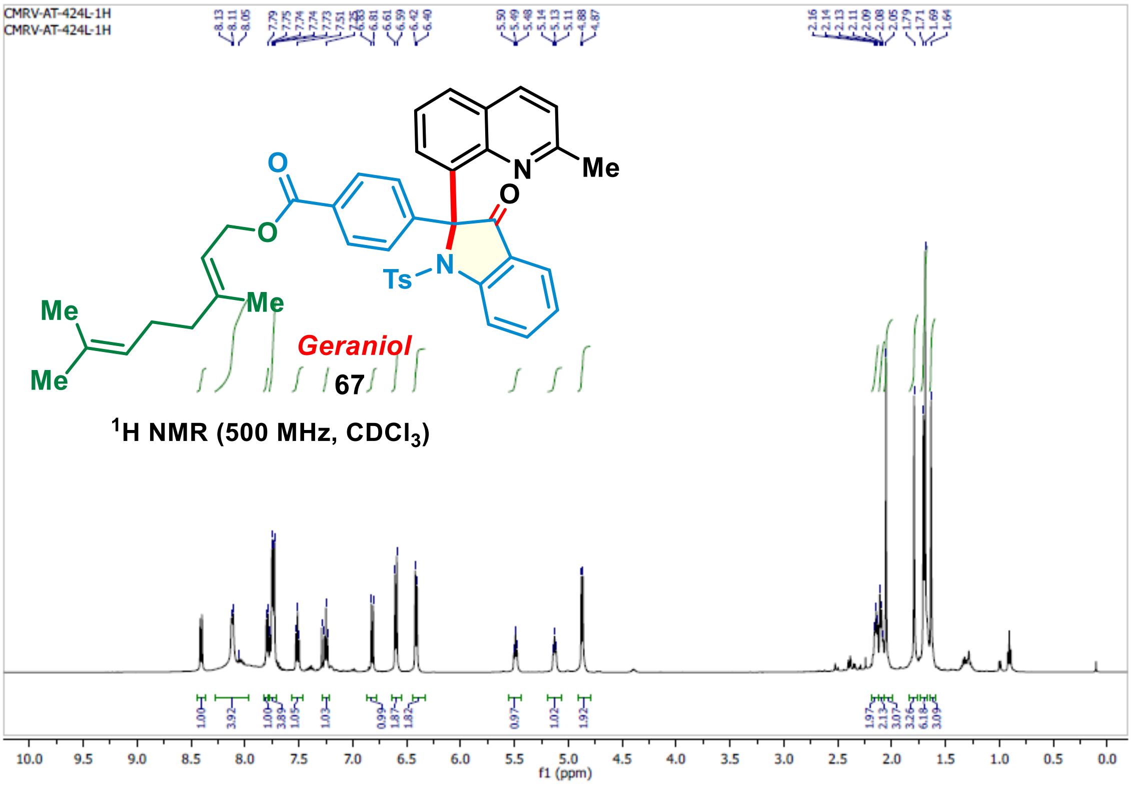

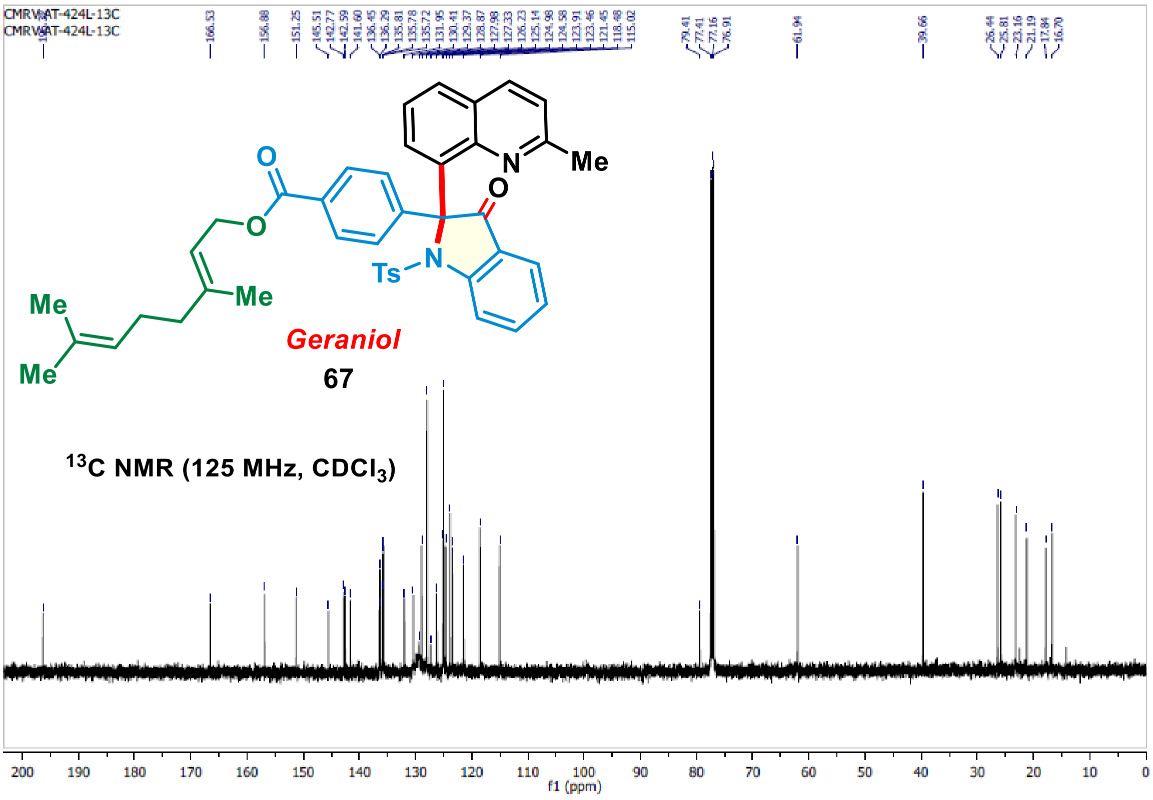
CMRV-AT-424L-13C
Geraniol
67
13C NMR (125 MHz, CDCl3)
f1 (ppm)

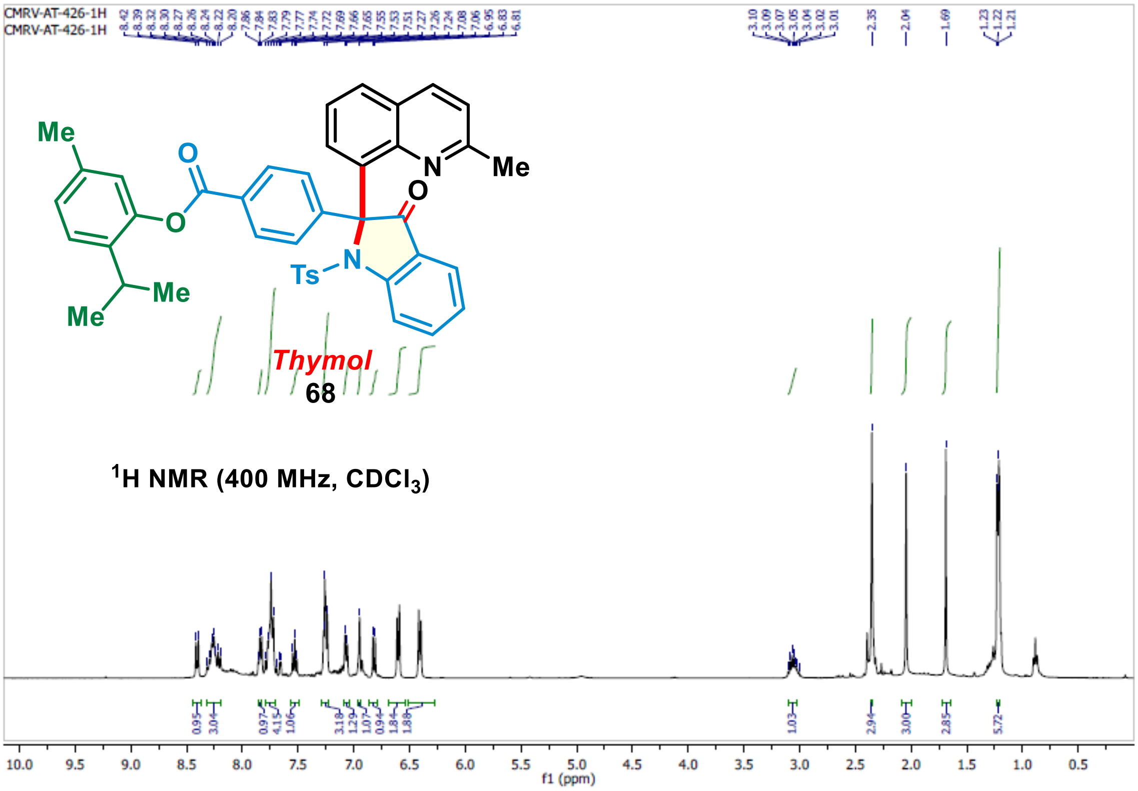
CMRV-AT-426-1H
Thymol
68
1H NMR (400 MHz, CDCl3)
f1 (ppm)

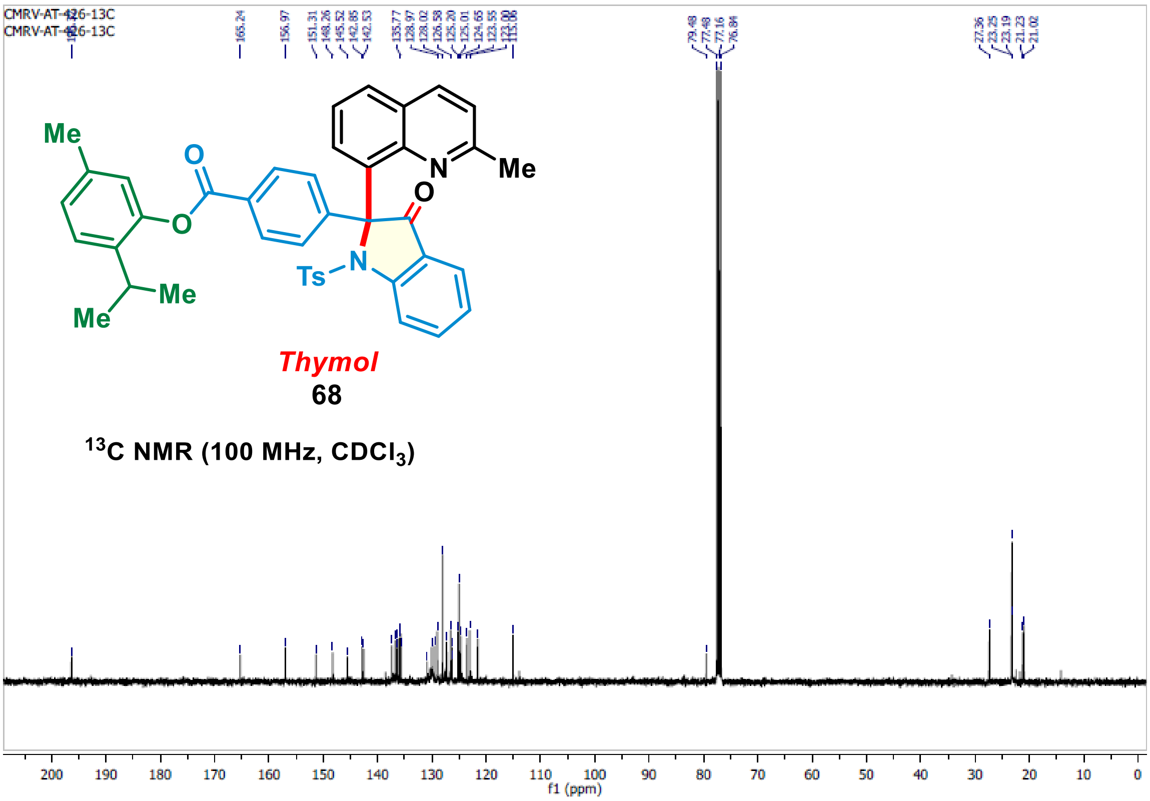
CMRV-AT-426-13C
Thymol
68
13C NMR (100 MHz, CDCl3)
f1 (ppm)

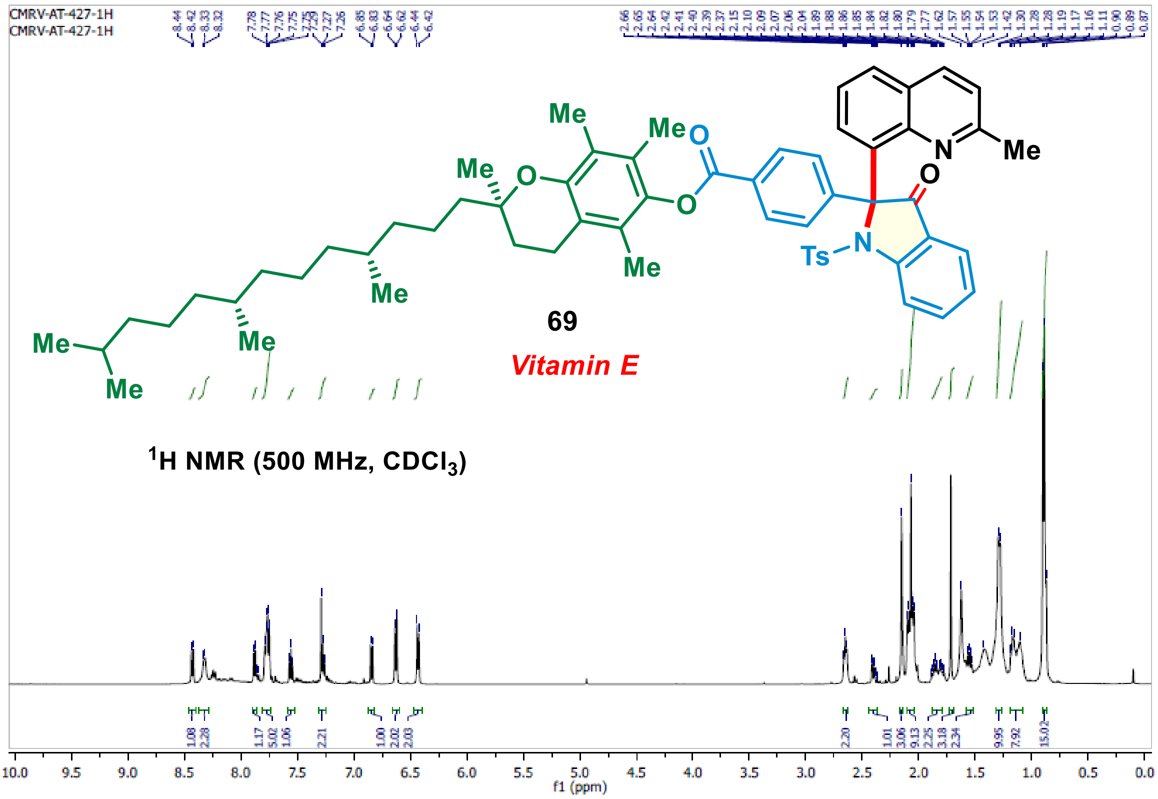
CMRV-AT-427-1H
69
Vitamin E
1H NMR (500 MHz, CDCl3)
f1 (ppm)

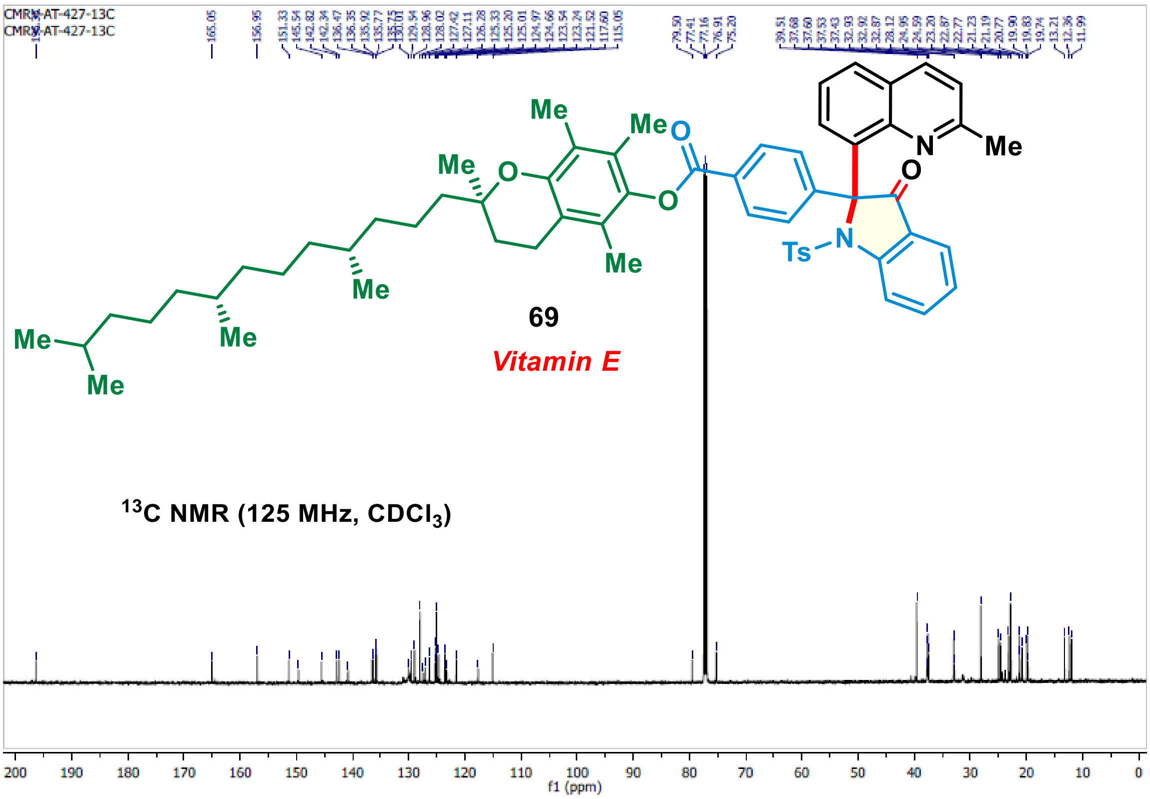
CMRV-AT-427-13C
Me
Me
Me
O
O
O
Me
Me
Me
Me
Me
N
Me
O
Ts
N
69
Vitamin E
13C NMR (125 MHz, CDCl3)
f1 (ppm)

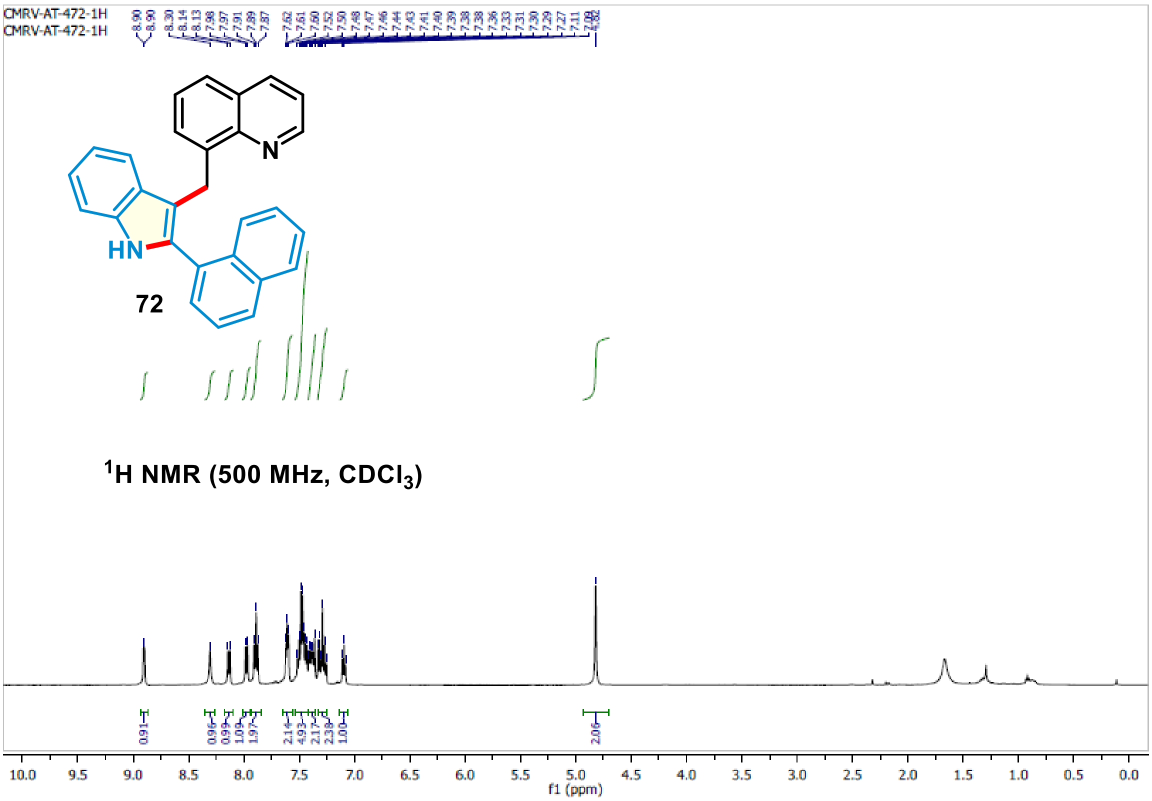
CMRV-AT-472-1H
N
HN
72
1H NMR (500 MHz, CDCl3)
f1 (ppm)

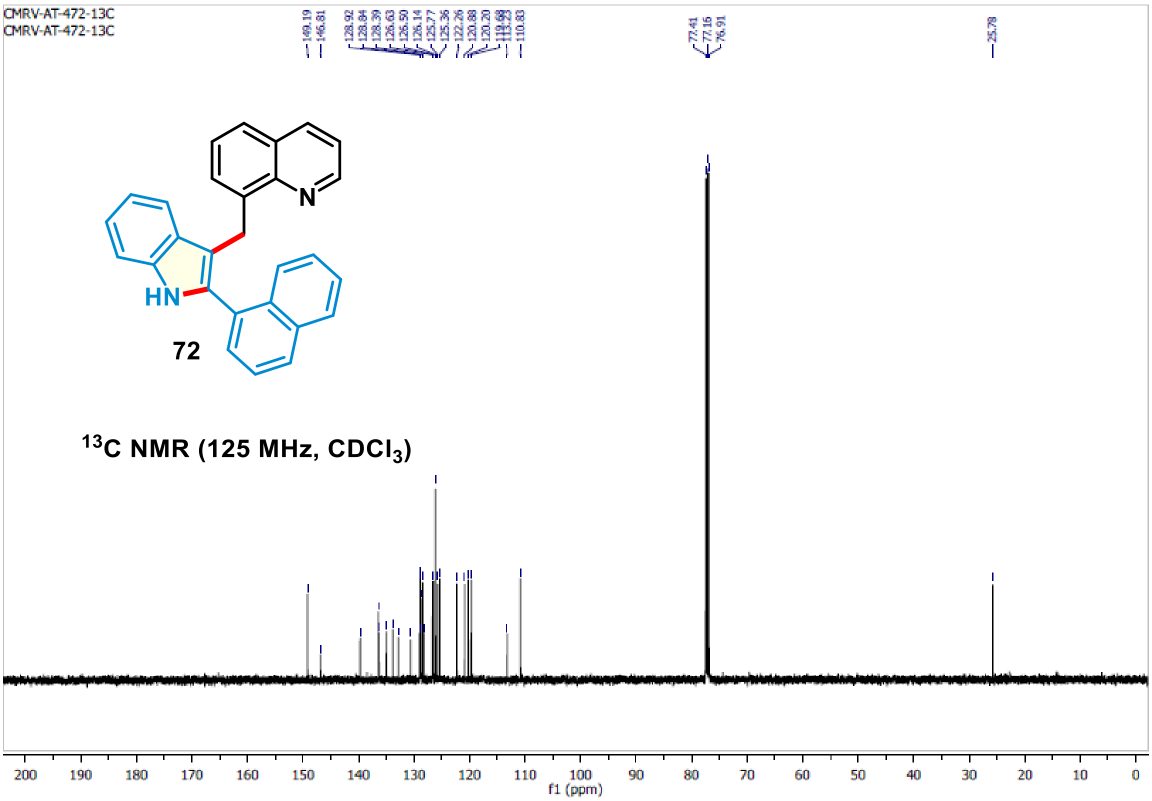

CMRV-AT-472-13C
CMRV-AT-472-13C
N
HN
72
^{13}C NMR (125 MHz, CDCl$_3$)
f1 (ppm)

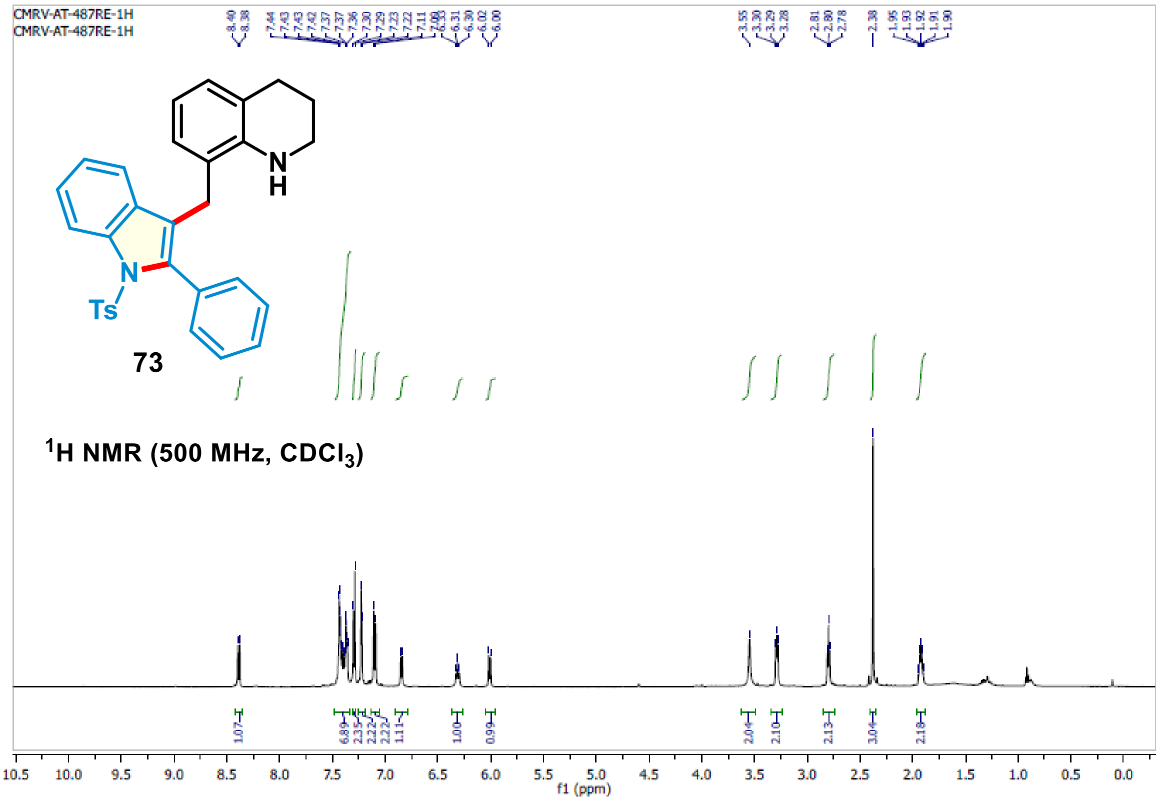

CMRV-AT-487RE-1H
CMRV-AT-487RE-1H
NH
N
Ts
73
^{1}H NMR (500 MHz, CDCl$_3$)
f1 (ppm)

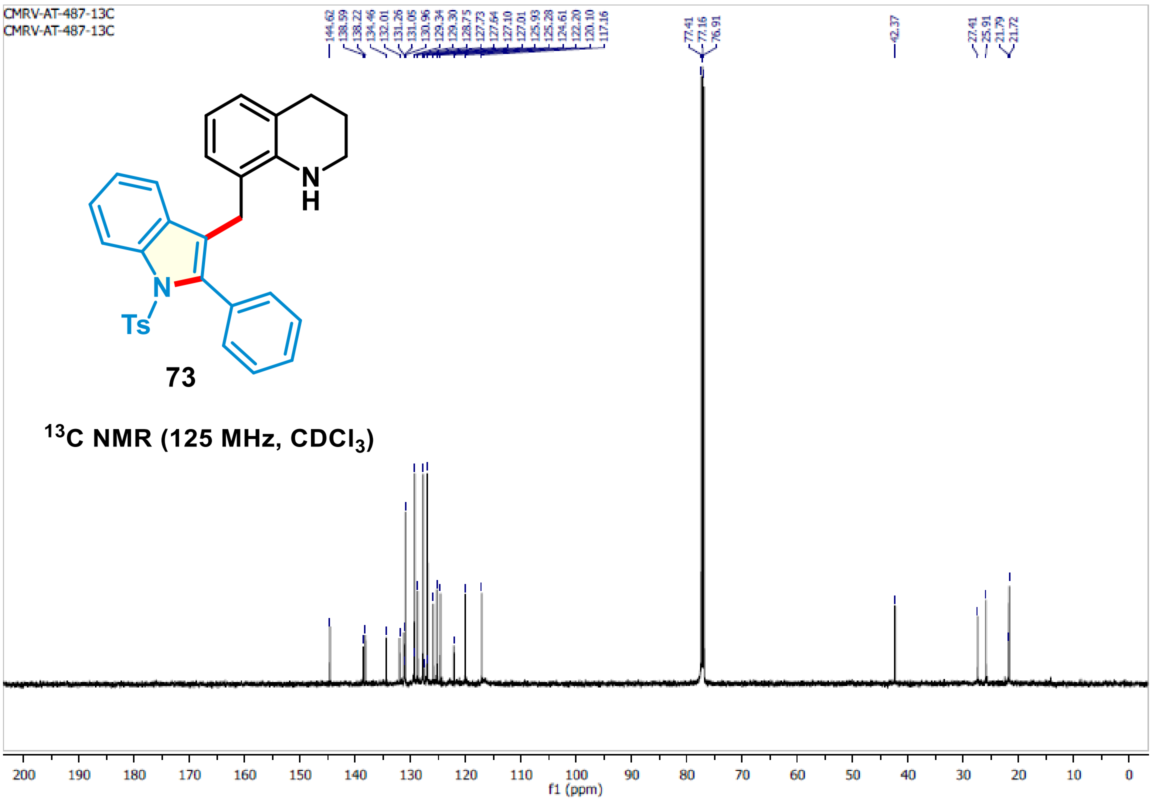
CMRV-AT-487-13C
CMRV-AT-487-13C
144.62
138.59
138.22
134.46
132.01
131.26
131.05
130.96
129.34
129.30
128.75
127.73
127.64
127.10
127.01
125.93
125.28
124.61
122.20
120.10
117.16
77.41
77.16
76.91
42.37
27.41
25.91
21.79
21.72
N
H
N
Ts
73
13C NMR (125 MHz, CDCl3)
f1 (ppm)

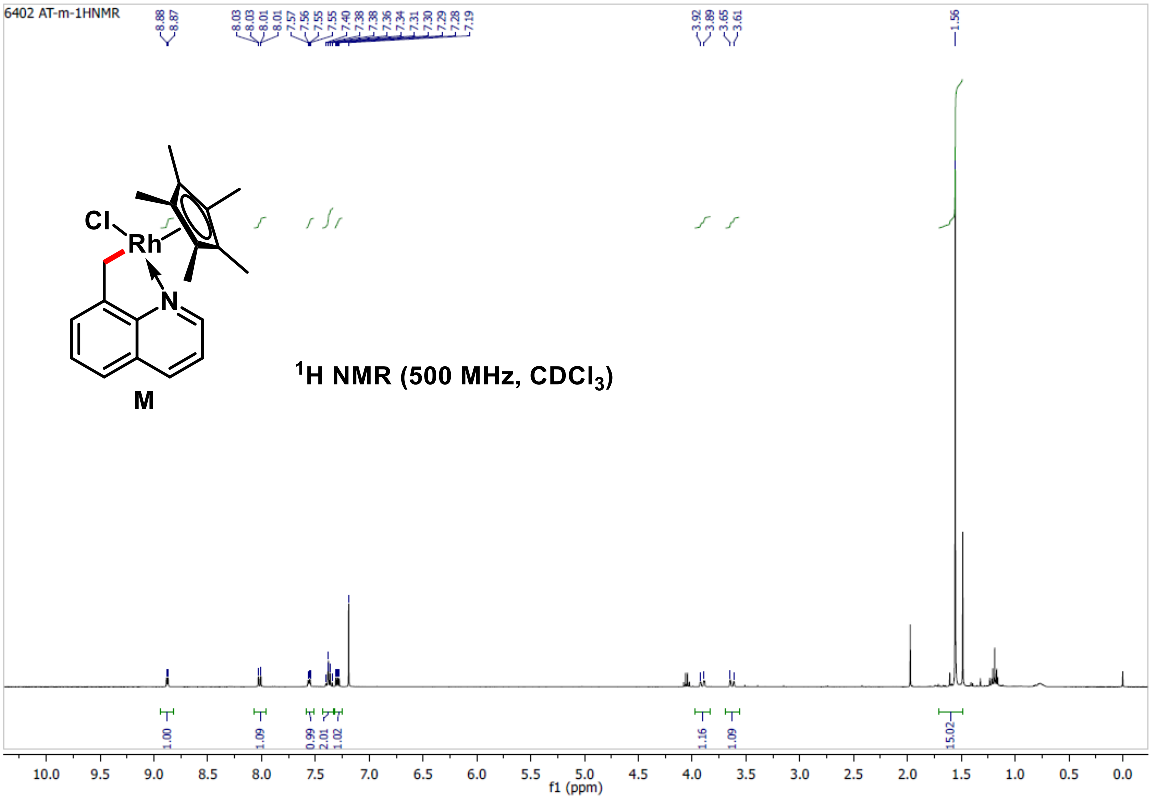
6402 AT-m-1HNMR
8.88
8.87
8.03
8.03
8.01
8.01
7.57
7.56
7.55
7.55
7.40
7.38
7.38
7.36
7.34
7.31
7.30
7.29
7.28
7.19
3.92
3.89
3.65
3.61
1.56
Cl
Rh
N
M
1H NMR (500 MHz, CDCl3)
1.00
1.09
0.99
2.01
1.02
1.16
1.09
15.02
f1 (ppm)

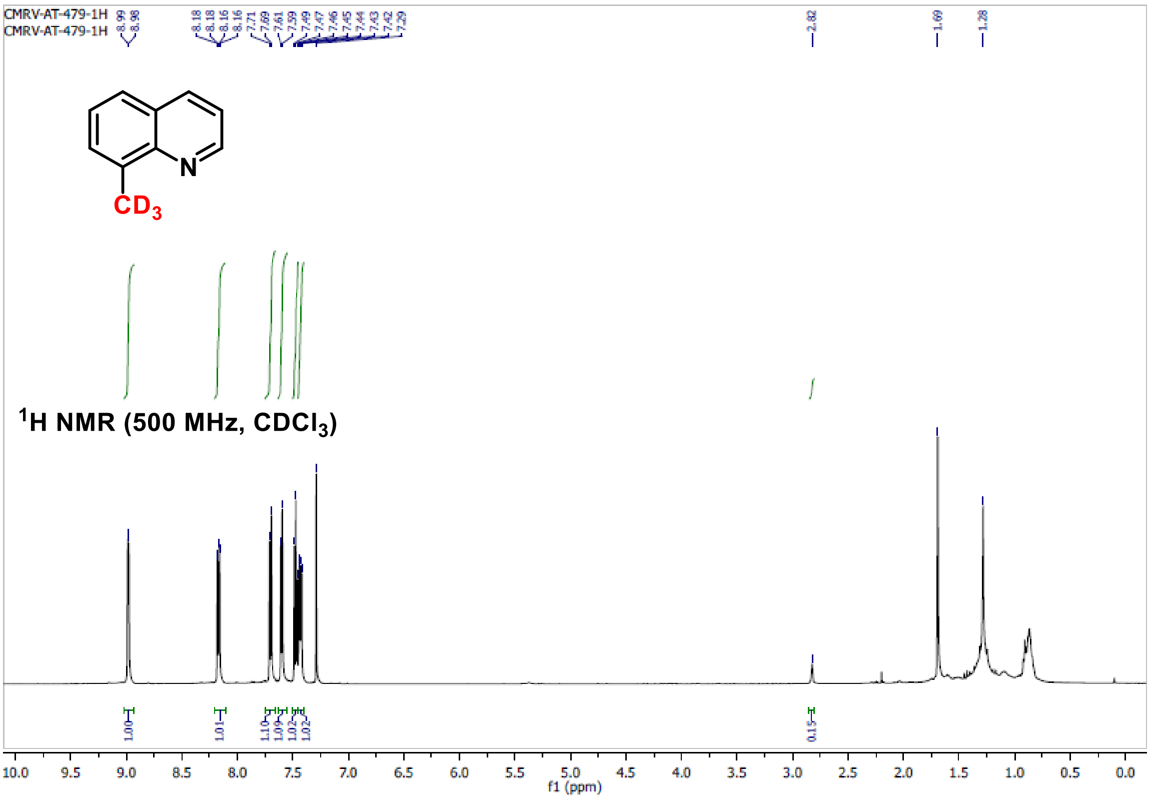
CMRV-AT-479-1H
1H NMR (500 MHz, CDCl3)
CD3
N
f1 (ppm)
